# Supplementary material for: A Global Screen for Assembly State Changes of the Mitotic Proteome by SEC-SWATH-MS
Source: Cell Syst. 2020 Feb 26;10(2):133–155.e6. doi: 10.1016/j.cels.2020.01.001 (PMC7042714; doi:10.1016/j.cels.2020.01.001)
Supplement: Document S1. Figures S1–S7 [file mmc1.pdf]

Cell Systems, Volume *10*

Supplemental Information

A Global Screen for Assembly State Changes of the Mitotic Proteome by SEC-SWATH-MS

Moritz Heusel, Max Frank, Mario Köhler, Sabine Amon, Fabian Frommelt, George Rosenberger, Isabell Bludau, Simran Aulakh, Monika I. Linder, Yansheng Liu, Ben C. Collins, Matthias Gstaiger, Ulrike Kutay, and Ruedi Aebersold

Supplemental Information

CELL-SYSTEMS-D-19-00261

Contents (this .pdf):

- Figure S1: Related to Figure 1. Validation of cell cycle arrest and inducible expression of HASt-tagged bait proteins in engineered HeLa cell lines.
- Figure S2: Related to Figures 1 & 2. Intensity normalization and correlation analysis.
- Figure S3: Related to Figure 5. Pathway enrichment of SEC- vs. thermostability-changing proteins.
- Figure S4: Related to Figures 2 & 3. Properties of multi-complex proteins.
- Figure S5: Related to Figures 6 & 7. Evaluation of complex-complex associations.
- Figure S6: Related to Figure 2, 3 & 4. Browsing dynamic complex association maps in SECexplorer-cc.
- Figure S7: Related to Figure 5 & 7. Comparison of differential scores across methods and NPC signal change.

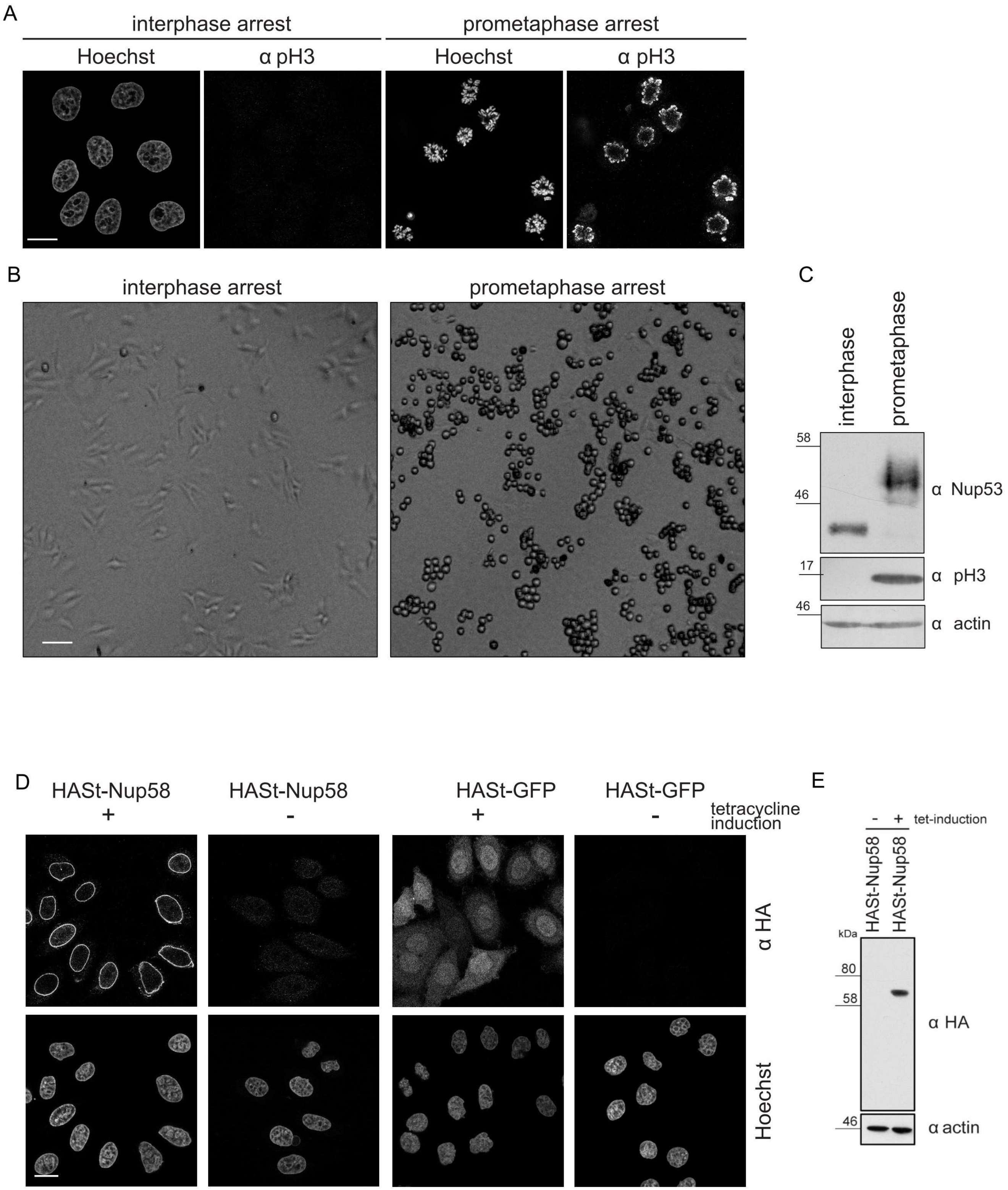

Figure S1: Related to Figure 1. Validation of cell cycle arrest and inducible expression of HASt-tagged bait proteins in engineered HeLa cell lines.

A Validation of homogenous interphase and prometaphase mitotic cell cycle arrest by visualizing chromatin structure (Hoechst staining) and detecting mitotic phosphorylation of histone H3 by immunofluorescence. Scale bar = 20 µm. **B** Phase contrast microscopy of HeLa CCL2 cells in interphase before harvest and in prometaphase before mitotic shake-off to retrieve only non-attached, rounded mitotic cells. Scale bar = 120 µm. **C** Validation of cell cycle synchronization by Western blotting and detection of mitosis-specific electrophoretic mobility shifts of hyper-phosphorylated Nup53 and Histone H3 phosphorylation. Anti-actin is included as loading control. **D** Validation of tetracycline-inducible HASt-Nup58 and control GFP bait expression in engineered HeLa cell lines by Immunofluorescence. Scale bar = 20 µm. **E** Validation of tetracycline-inducible HASt-Nup58 bait expression and size by Western blotting. Anti-actin is included as loading control.

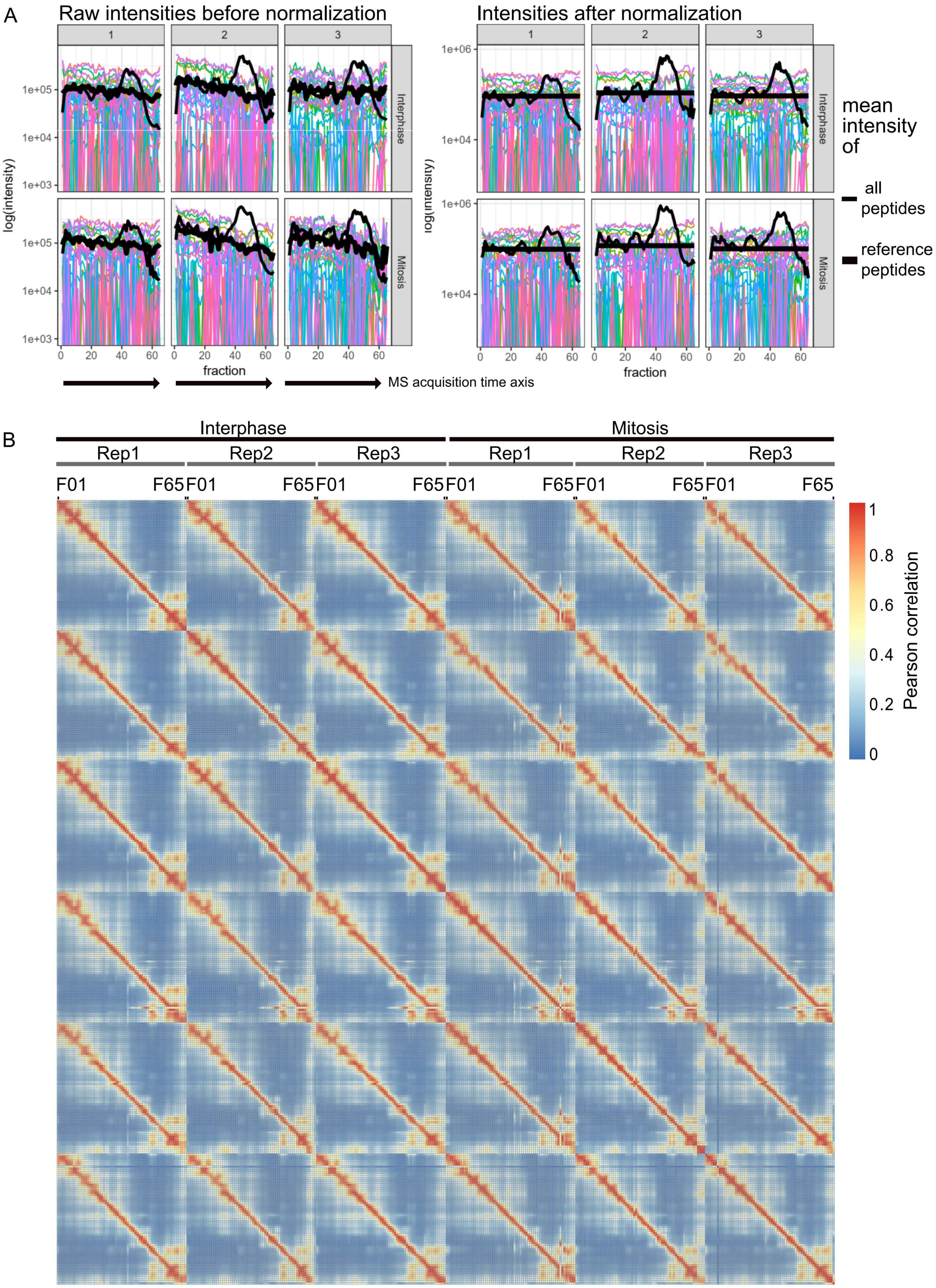

Figure S2: Related to Figures 1 & 2. Intensity normalization and correlation analysis.

A Normalization of peptide intensities based on the mean signal from a reference set of peptides spiked into each fraction prior to C-18 cleanup and MS analysis (E. coli β-galactosidase tryptic digest). The thick black line represents the mean intensity obtained from these spike-in peptides and indicates longitudinal deterioration of mass spectrometer performance in all replicates (columns) and conditions (rows) (left panels). Peptide intensities over all 390 SWATH-MS measurements were corrected by scaling to the internal standard spike-in. Impact on summed global peptide traces is given in the thin black line. Furthermore, scaling and global intensity sum smoothing along fractions was applied. For details see STAR methods. Arrows indicate the order of MS acquisition. **B** Intensity correlation analysis to assess reproducibility of protein intensities (top 2 peptide sum quantification) across replicates and conditions. Protein-level correlation analysis was performed on the global intersect set of proteins observed in all fractions. Resulting protein intensities are highly correlated across replicates and adjacent fractions with an average Pearson’s R > 0.98 between replicate fractions of the same biological condition. Labels are given on the top, sample order is identical in x- and y- directions. Sample-sample correlation further shows highest resolution ("sharpness" of diagonal) in the SEC fraction range 40-50 and lower resolution in the early 'void volume' fractions 1-5 where many analytes greater than or equal to the exclusion limit of the 500 Å diameter pores of the Sepax SRT-C SEC 500 column elute.

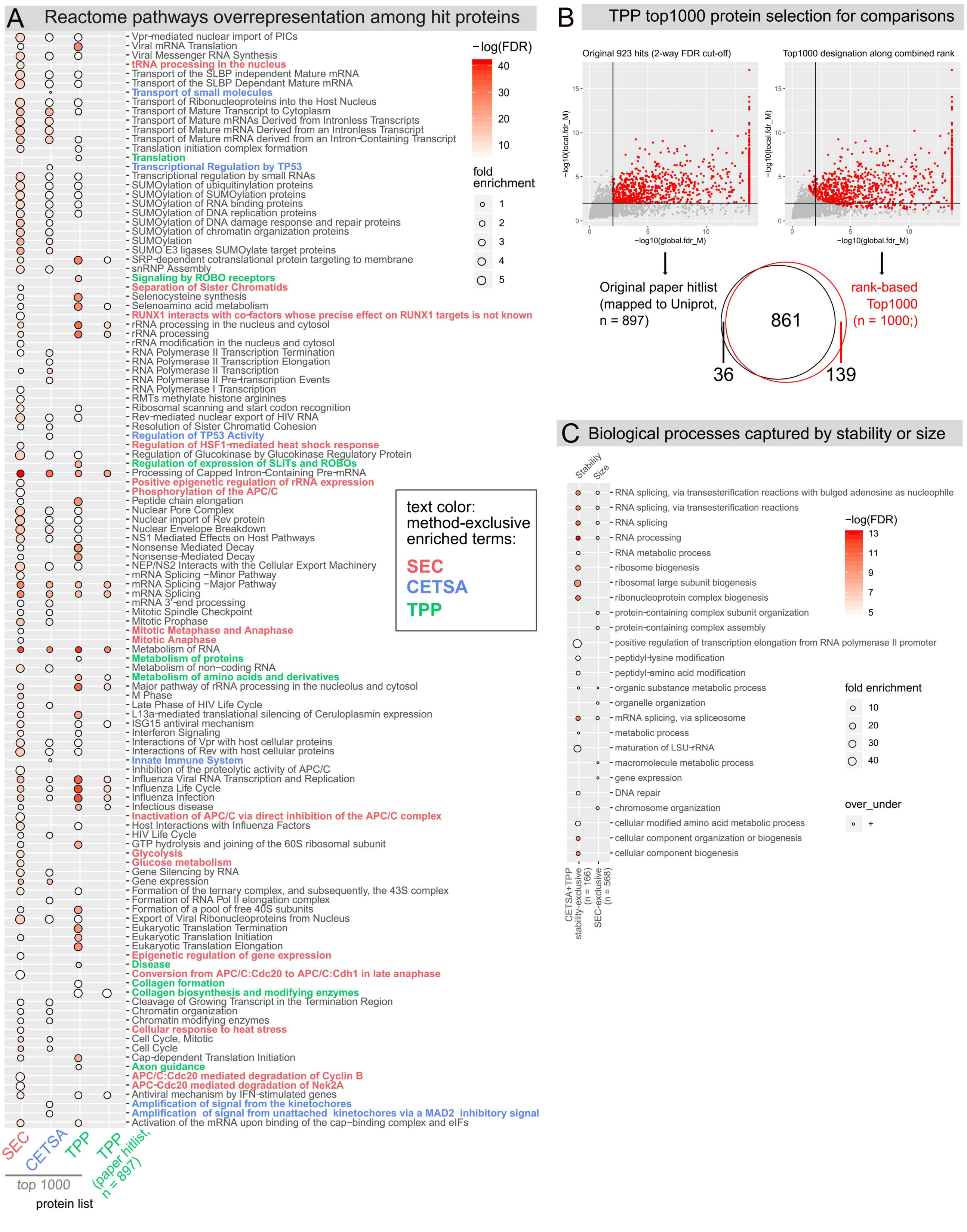

Figure S3: Related to Figure 5. Pathway enrichment of SEC- vs. thermostability-changing proteins.

A Pathway enrichment testing among the top 1000 proteins reported to change association or stability states per each method using the Panther system with Reactome pathway annotations (http://pantherdb.org/). **B** Illustration of rank-based selection of the 1000 most-regulated proteins from the TPP results (Becher et al. 2018) used for enrichment testing in panel A. **C** Biological process annotation enrichment among proteins reported exclusively by SEC (n = 568) and proteins reported by both thermostability-based studies (n = 166).

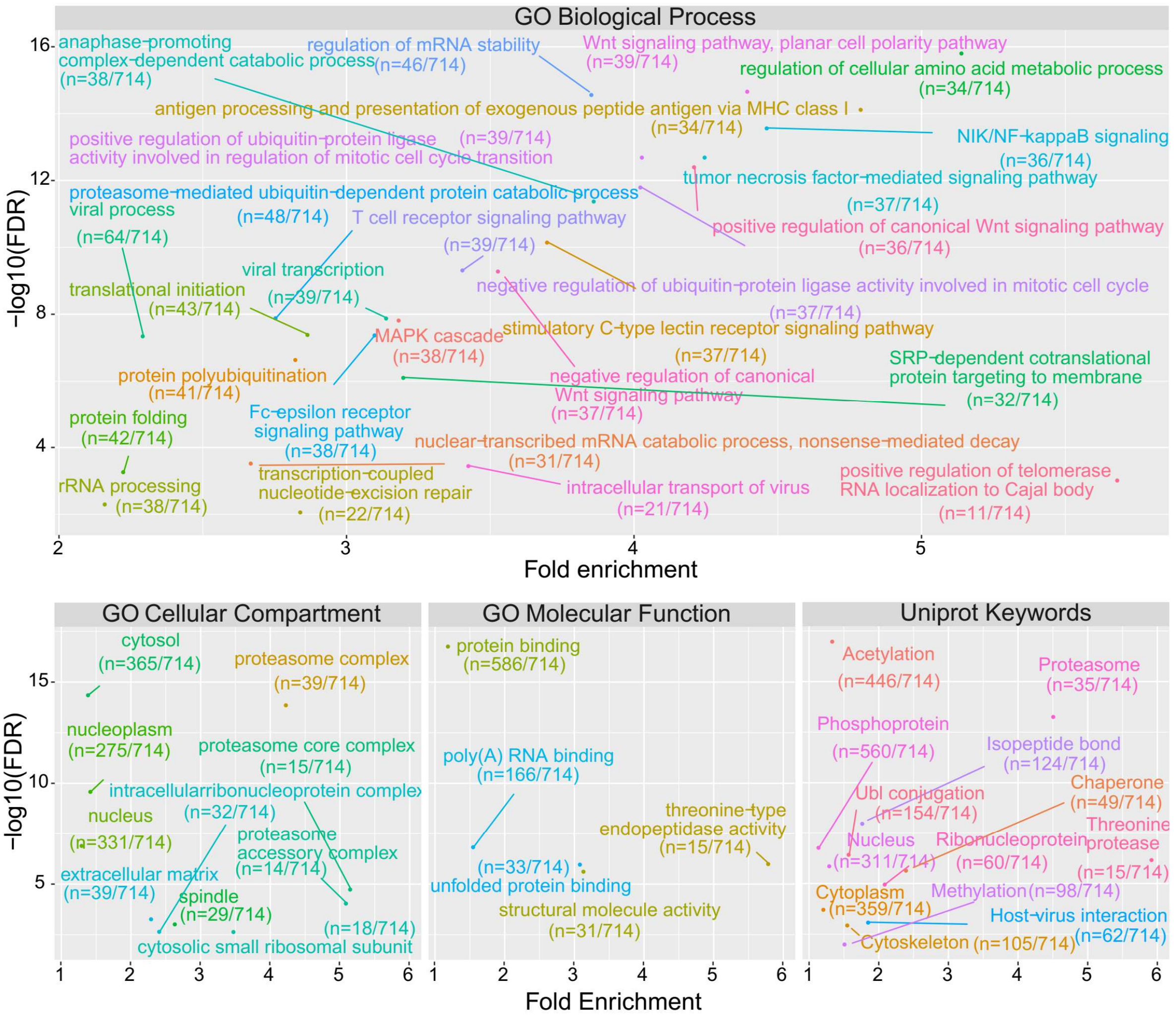

Figure S4: Related to Figures 2 & 3. Protein peak detection and properties of multi-complex signal proteins.

DAVID functional annotation enrichment testing of proteins observed in two or more distinct complex-assembled states against the background of all 4,515 proteins for which at least one elution peak was detected (performed at https://david.ncifcrf.gov/). Multi-complex proteins are enriched in signaling factors (despite global underrepresentation of signaling molecules, see **Figure S3B**) and proteins known to have binding functions, as well as proteins known to function in relation to post-translational modifications such as acetylation and phosphorylation.

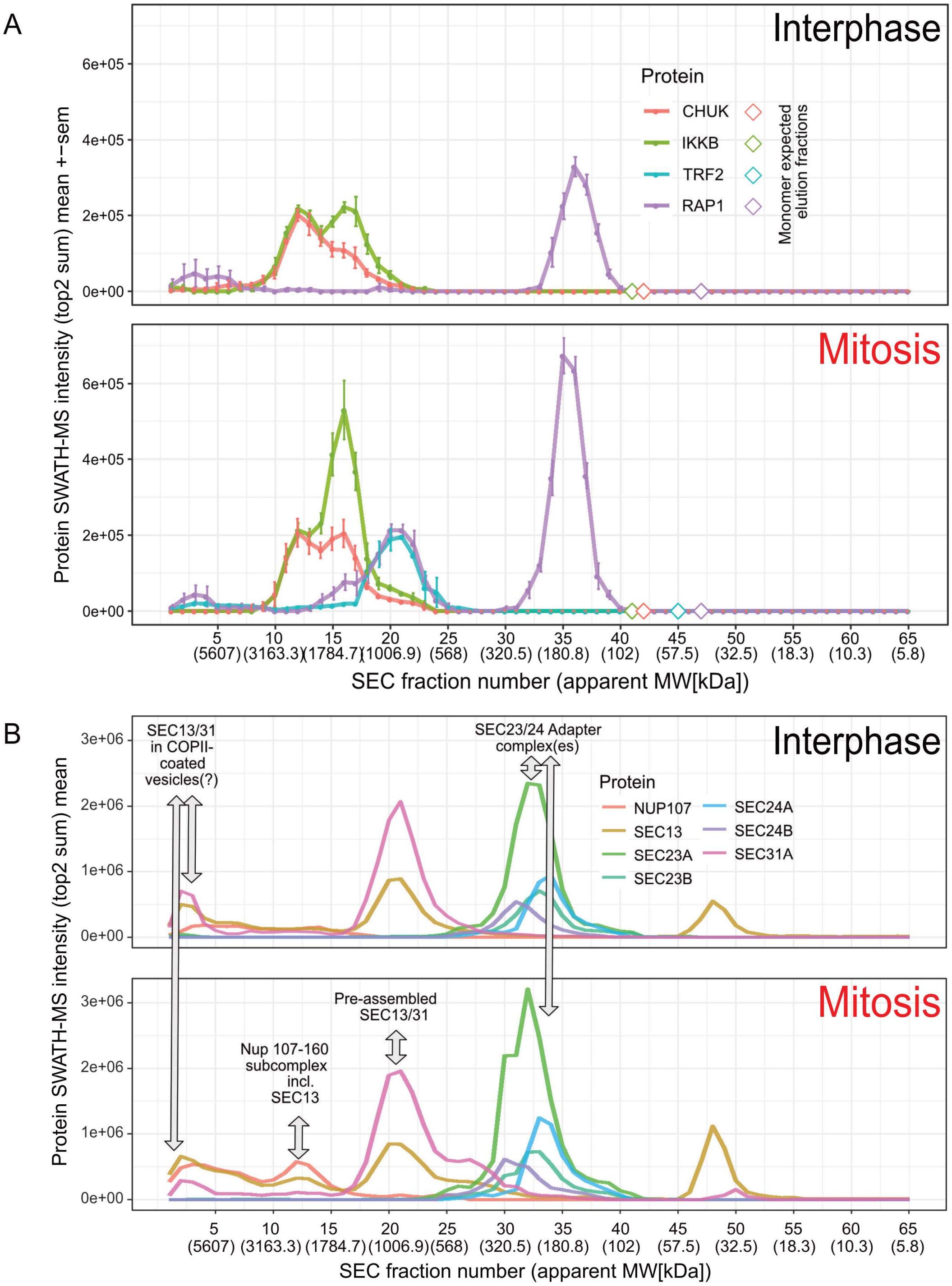

Figure S5: Related to Figures 6 & 7. Evaluation of complex-complex associations.

A Related to Figure 6F. SEC chromatogram overlay to assess complex-complex interaction between RAP1-TRF2 complex and the IKK complex (represented by subunits CHUK and IKKB). IKK complex appears in two subpopulations, one of ca. 2.5 MDa (apex fraction 12) and one of ca. 1.7 MDa (apex fraction 16), with a peak shoulder in the mitotic 950 kDa-elution signal of RAP1 in principle consistent with recruitment of a fraction of RAP1 but not TRFII to the 1.7 MDa instance of the IKK complex. Equivalent custom analyses can rapidly be performed via *SECexplorer-cc*. **B** Related to Figure 6H. Protein-level mean SEC-SWATH-MS chromatograms of Nup107-160 sub-complex subunits NUP107 and SEC13 and additional protein complex partners of SEC13 as part of COPII coatomer complexes involved in anterograde vesicle-mediated transport, SEC23(A/B), SEC24(A/B) and SEC31(A). From left to right, large to small assemblies, the profiles suggest: (i) Potential SEC13/31 interaction in and elution as part of COPII-coated vesicles in the void volume; (ii) NUP107 and SEC13 appearance as part of the Nup107-160 sub-complex eluting with apex in fraction 12/13, ca. 2.8 MDa; (iii) SEC13-SEC31 co-elution with ca. 1 MDa, apex fraction 20-21, potentially pre-assembled when not participation in coatomers and (iv) SEC23A/B and SEC24A/B co-elution in fractions 30-35, 320 - 180 kDa, likely preassembled into a mixed population of variant adaptor complexes.

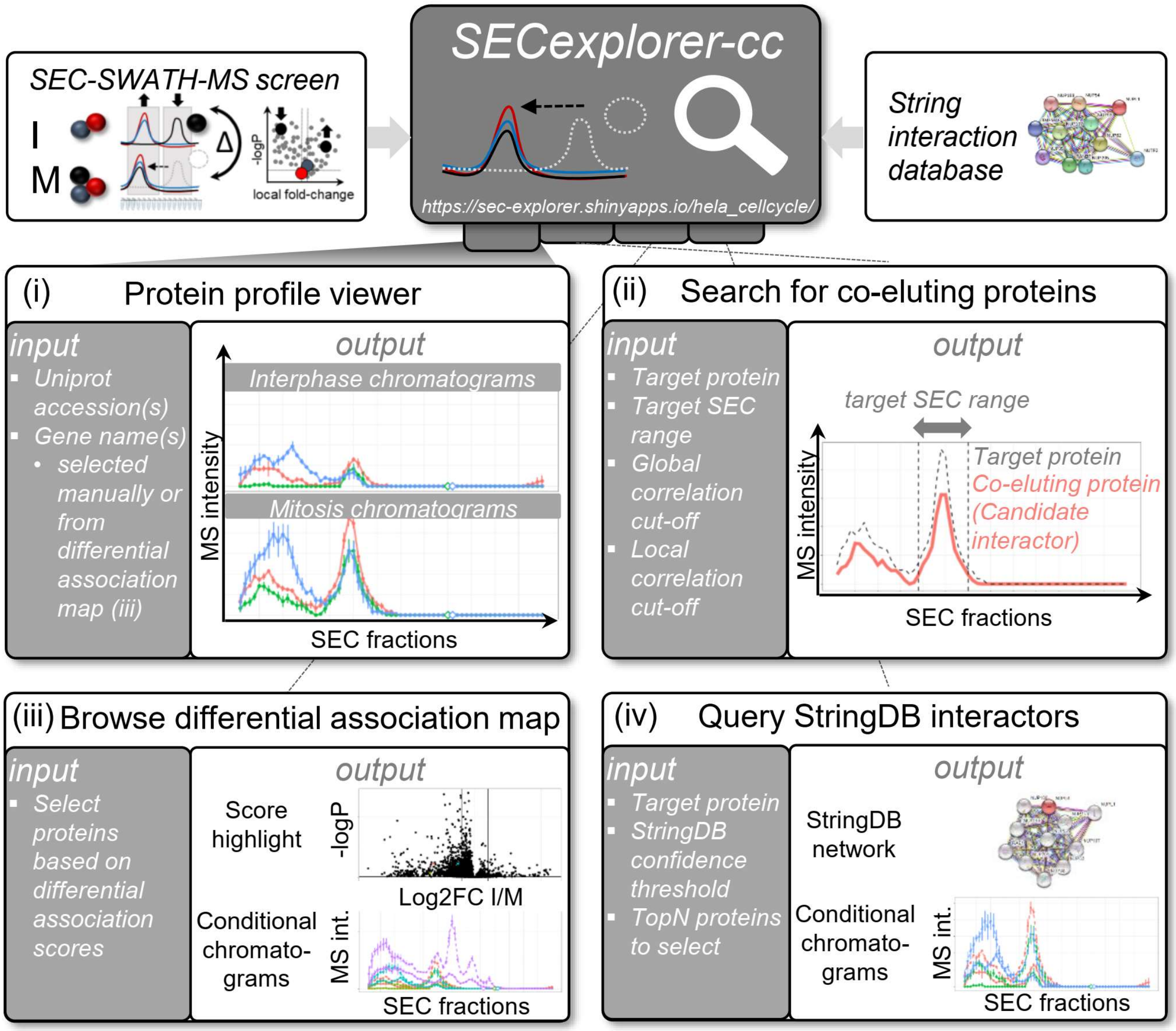

Figure S6: Related to Figure 2, 3 & 4. Browsing dynamic complex association maps in SECexplorer-cc.

Overview of SECexplorer-cc, a web tool that allows to browse and rapidly interrogate differential SEC-SWATH-MS datasets to support researches in manual review and customized hypothesis testing. SECexplorer-cc currently combines the dynamic cell-cycle proteome association map reported here and integrates it with information from the String database (Szklarczyk et al., 2017). SECexplorer-cc features four core functionalities. (i) Interactive display and hit protein selection from the statistical SEC shift score map. (ii) Semi-targeted search for locally co-eluting proteins to identify putative new binding partners showing strong co-elution within a certain range of target protein elution. (iii) Display of custom protein sets conditional fractionation profiles. (iv) Display of one or multiple hit protein's fractionation profiles in reference to its immediate functional and/or physical interaction partners obtained StringDB to discover potential physical association among functionally related proteins and to add cellular context information.

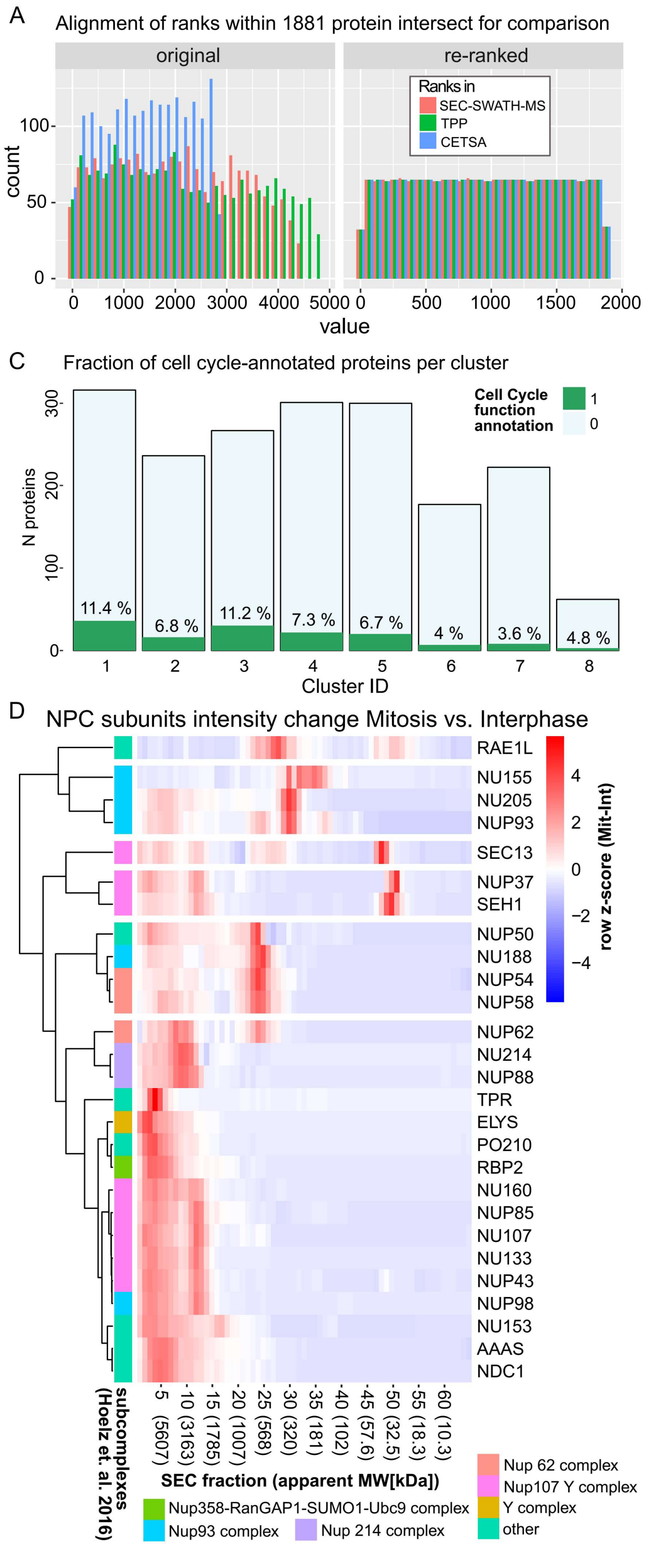

Figure S7: Related to Figure 5 & 7. Comparison of differential scores across methods and NPC signal change.

A Aligning the scales among the method-specific mitotic change ranks among the 1,881 proteins covered by all three methods by re-ranking to compare methods performance based on protein ranking patterns. **B** Comparison of protein ranking patterns and group assignment based on k-score (n = 8). C Estimation of protein cluster/group 'quality' based on the rate of observing 'true positive' proteins with annotated functions in relation to the cell cycle (UniprotKB annotation). **D** Related to Figure 7A. The protein-level pattern changes between mitosis and interphase. For visualization, protein intensity means across replicates per condition were subtracted and values scaled by row-wise by z-score transformation (R/pheatmap package).
