# Supplementary material for: A Global Screen for Assembly State Changes of the Mitotic Proteome by SEC-SWATH-MS
Source: Cell Syst. 2020 Feb 26;10(2):133–155.e6. doi: 10.1016/j.cels.2020.01.001 (PMC7042714; doi:10.1016/j.cels.2020.01.001)

A0AVT1 | UBA6_HUMAN | UBA6 MOP4 UBE1L2
Monomer MW [kDa]: 117.97 Monomer expected elution fraction: 39

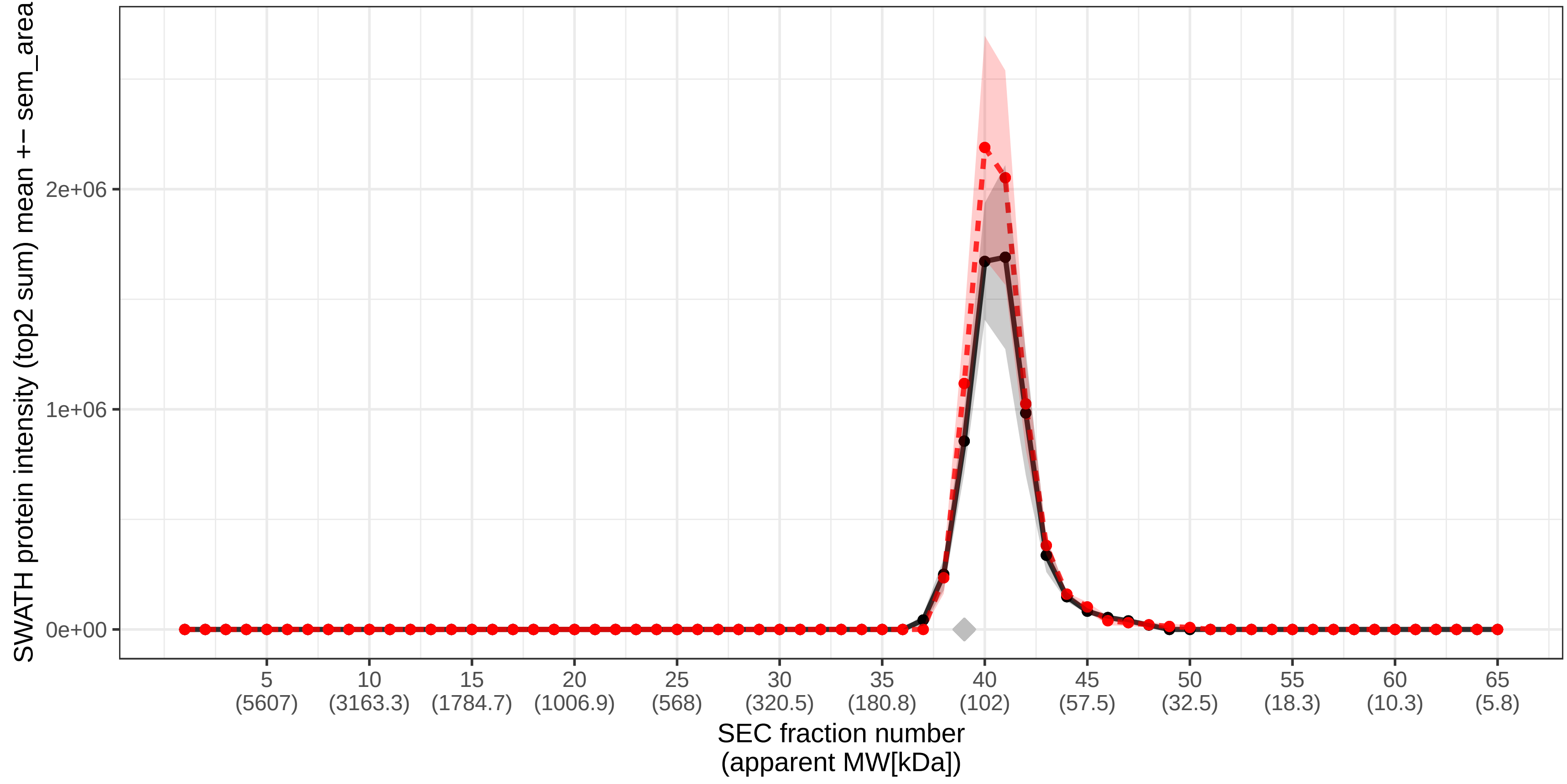

Supplement: Data S1. SEC-SWATH-MS Protein Chromatograms, Related to Figure 1 [file mmc6.zip › SECchrom_A0AVT1_UBA6_HUMAN_UBA6_MOP4_UBE1L2.pdf]
