# Supplementary material for: A Global Screen for Assembly State Changes of the Mitotic Proteome by SEC-SWATH-MS
Source: Cell Syst. 2020 Feb 26;10(2):133–155.e6. doi: 10.1016/j.cels.2020.01.001 (PMC7042714; doi:10.1016/j.cels.2020.01.001)

O00264 | PGRC1_HUMAN | PGRMC1 HPR6.6 PGRMC
Monomer MW [kDa]: 21.671 Monomer expected elution fraction: 54

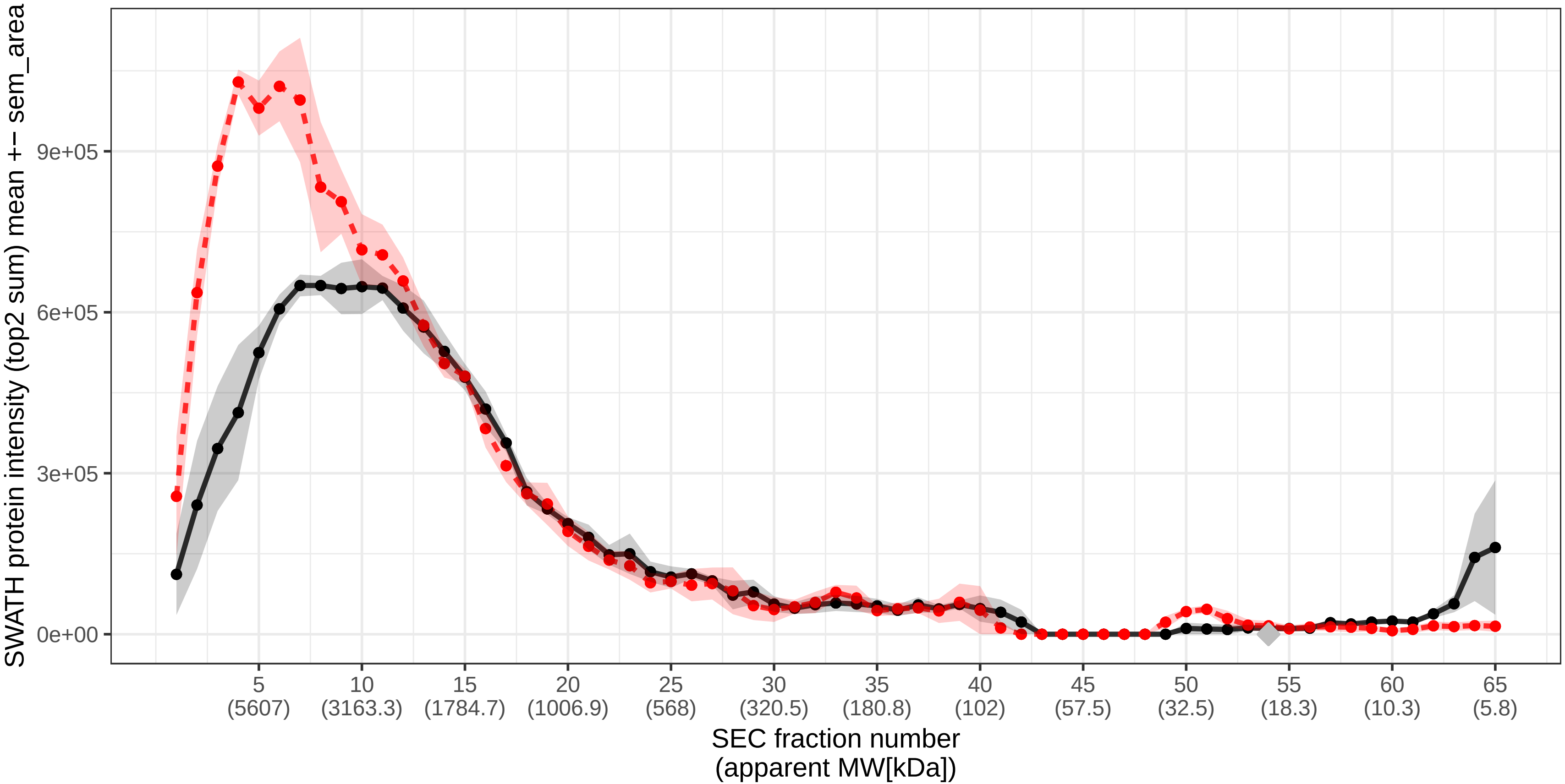

Supplement: Data S1. SEC-SWATH-MS Protein Chromatograms, Related to Figure 1 [file mmc6.zip › SECchrom_O00264_PGRC1_HUMAN_PGRMC1_HPR6.6_PGRMC.pdf]
