# Supplementary material for: A Global Screen for Assembly State Changes of the Mitotic Proteome by SEC-SWATH-MS
Source: Cell Syst. 2020 Feb 26;10(2):133–155.e6. doi: 10.1016/j.cels.2020.01.001 (PMC7042714; doi:10.1016/j.cels.2020.01.001)
Supplement: Data S1. SEC-SWATH-MS Protein Chromatograms, Related to Figure 1 [file mmc6.zip › SECchrom_O00410_IPO5_HUMAN_IPO5_KPNB3_RANBP5.pdf]

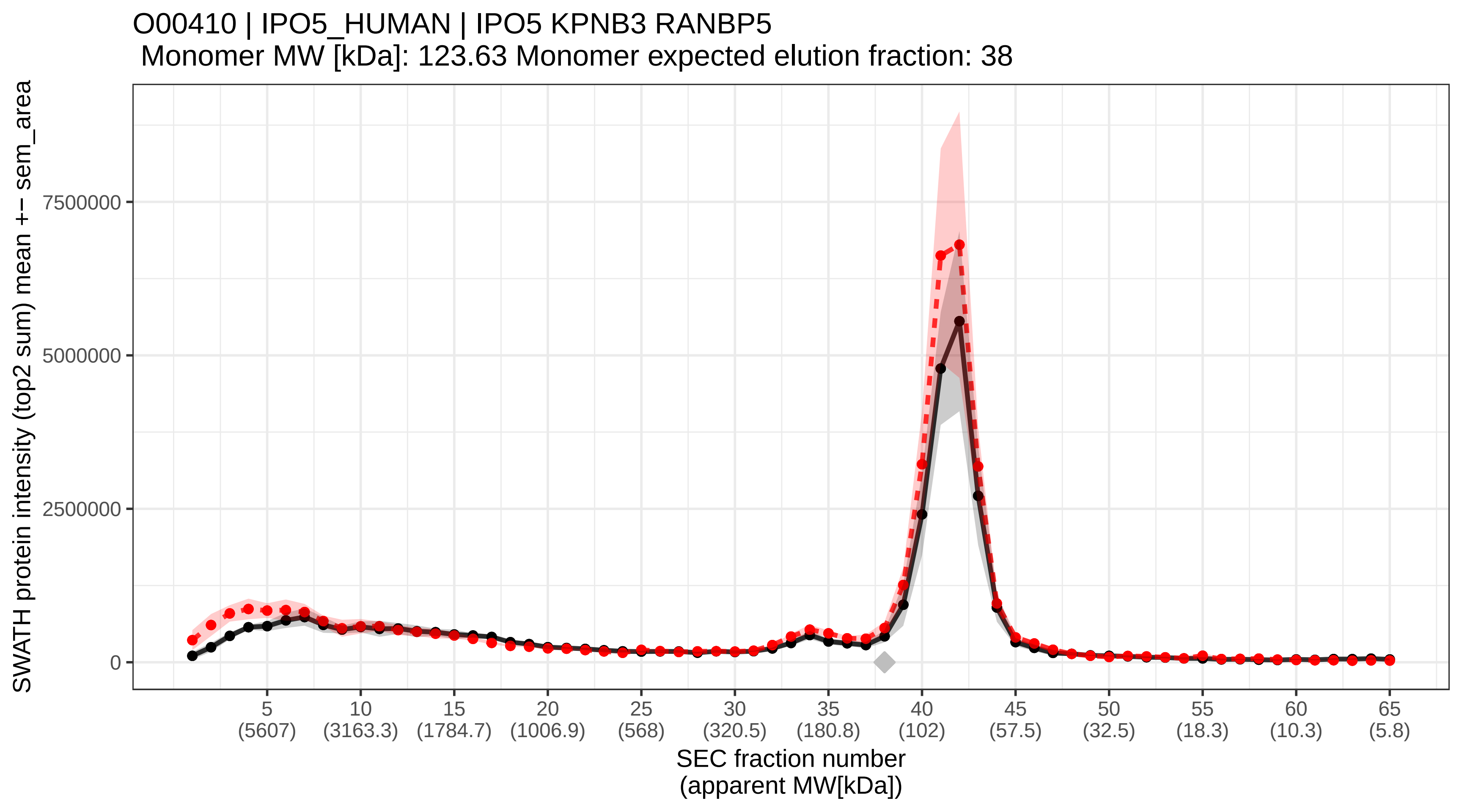
O00410 | IPO5_HUMAN | IPO5 KPNB3 RANBP5
Monomer MW [kDa]: 123.63 Monomer expected elution fraction: 38
SWATH protein intensity (top2 sum) mean +− sem_area
7500000
5000000
2500000
0
5 (5607)
10 (3163.3)
15 (1784.7)
20 (1006.9)
25 (568)
30 (320.5)
35 (180.8)
40 (102)
45 (57.5)
50 (32.5)
55 (18.3)
60 (10.3)
65 (5.8)
SEC fraction number
(apparent MW[kDa])
