# Supplementary material for: A Global Screen for Assembly State Changes of the Mitotic Proteome by SEC-SWATH-MS
Source: Cell Syst. 2020 Feb 26;10(2):133–155.e6. doi: 10.1016/j.cels.2020.01.001 (PMC7042714; doi:10.1016/j.cels.2020.01.001)

O00622 | CYR61_HUMAN | CYR61 CCN1 GIG1 IGFBP10
Monomer MW [kDa]: 42.027 Monomer expected elution fraction: 48

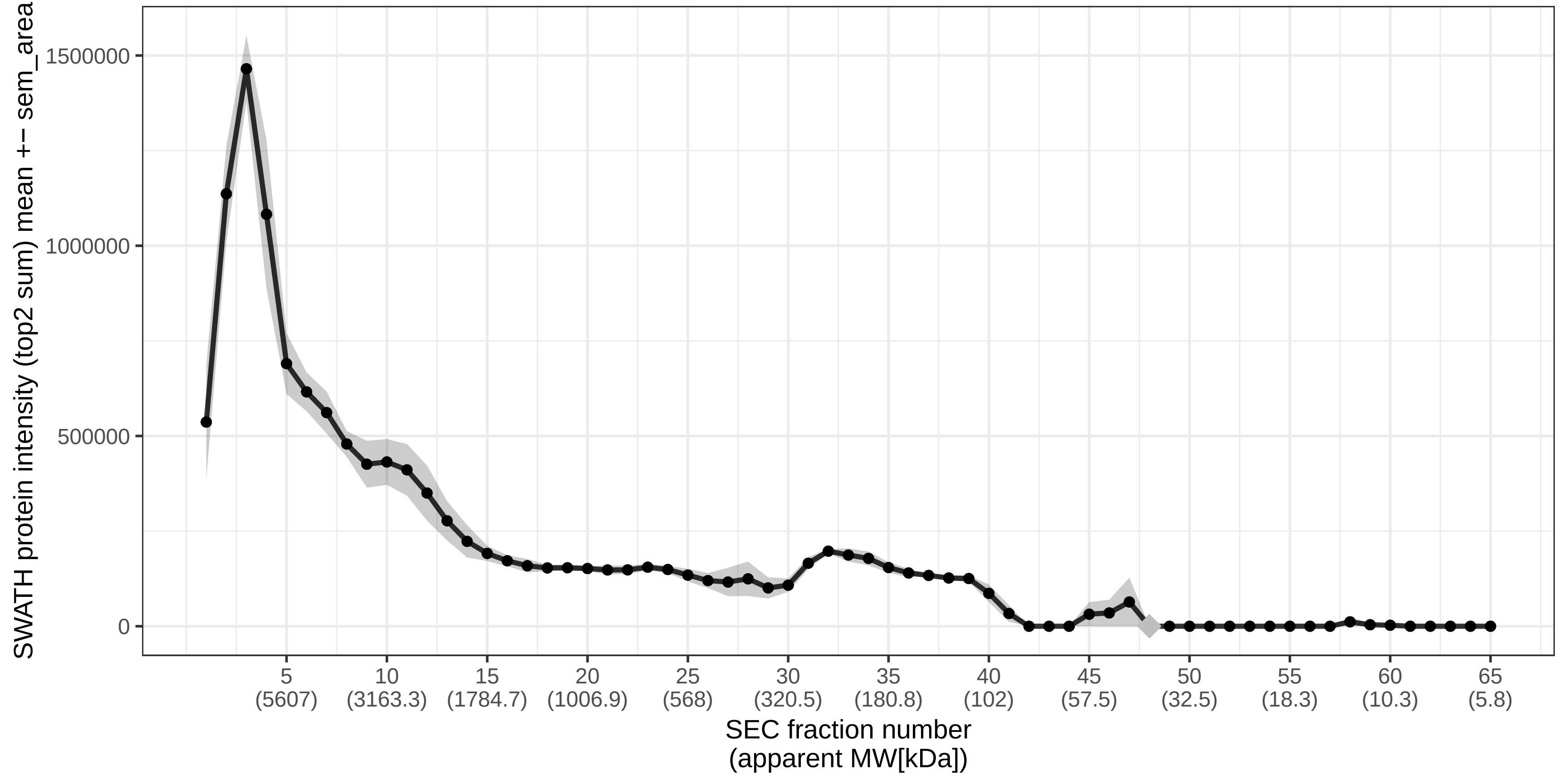

Supplement: Data S1. SEC-SWATH-MS Protein Chromatograms, Related to Figure 1 [file mmc6.zip › SECchrom_O00622_CYR61_HUMAN_CYR61_CCN1_GIG1_IGFBP10.pdf]
