# Supplementary material for: A Global Screen for Assembly State Changes of the Mitotic Proteome by SEC-SWATH-MS
Source: Cell Syst. 2020 Feb 26;10(2):133–155.e6. doi: 10.1016/j.cels.2020.01.001 (PMC7042714; doi:10.1016/j.cels.2020.01.001)

O14733 | MP2K7_HUMAN | MAP2K7 JNKK2 MEK7 MKK7 PRKMK7 SKK4
Monomer MW [kDa]: 47.485 Monomer expected elution fraction: 47

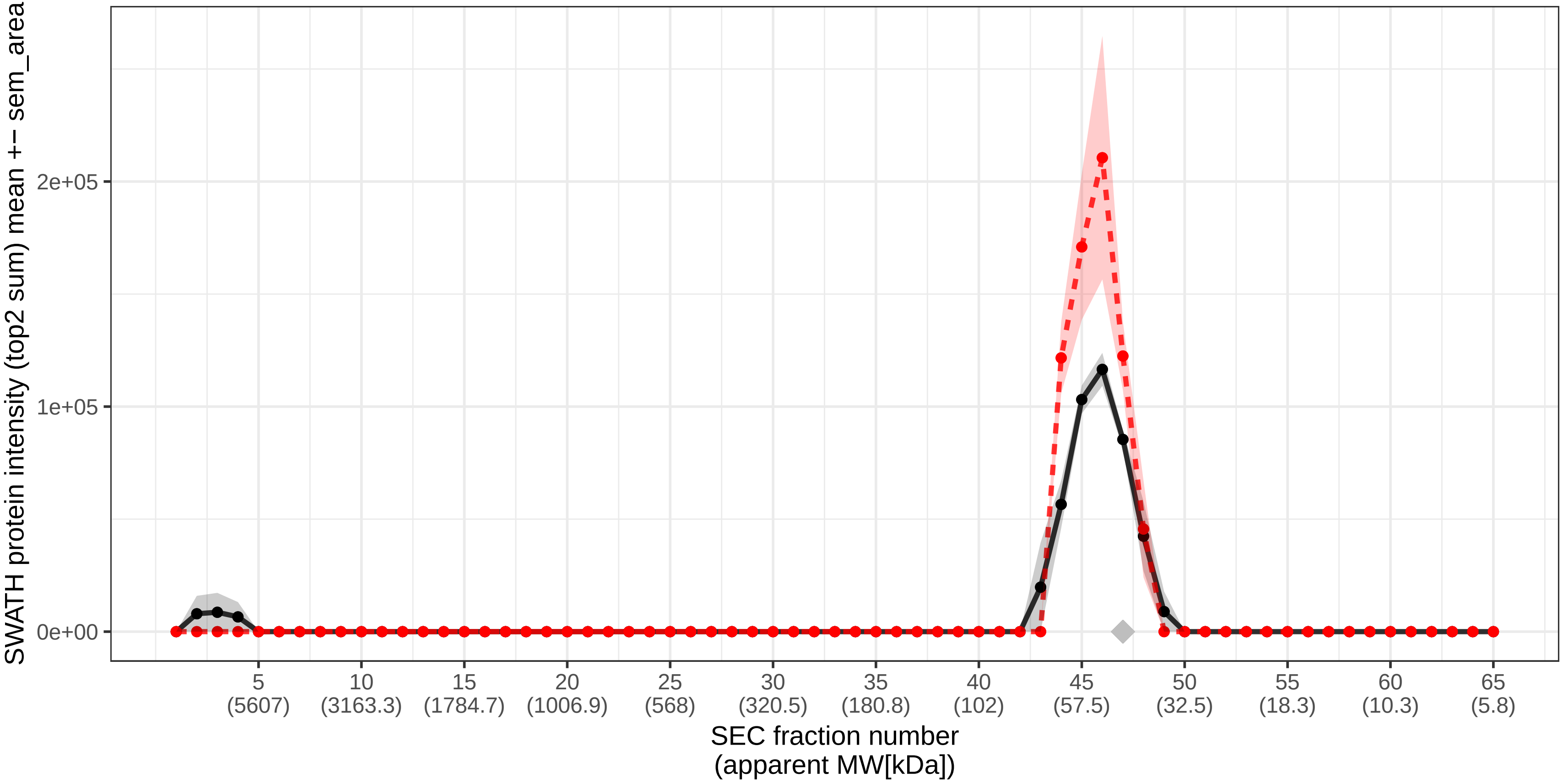

Supplement: Data S1. SEC-SWATH-MS Protein Chromatograms, Related to Figure 1 [file mmc6.zip › SECchrom_O14733_MP2K7_HUMAN_MAP2K7_JNKK2_MEK7_MKK7_PRKMK7_S.pdf]
