# Supplementary material for: A Global Screen for Assembly State Changes of the Mitotic Proteome by SEC-SWATH-MS
Source: Cell Syst. 2020 Feb 26;10(2):133–155.e6. doi: 10.1016/j.cels.2020.01.001 (PMC7042714; doi:10.1016/j.cels.2020.01.001)

O14979 | HNRDL_HUMAN | HNRNPDL HNRPDL JKTBP
Monomer MW [kDa]: 46.438 Monomer expected elution fraction: 47

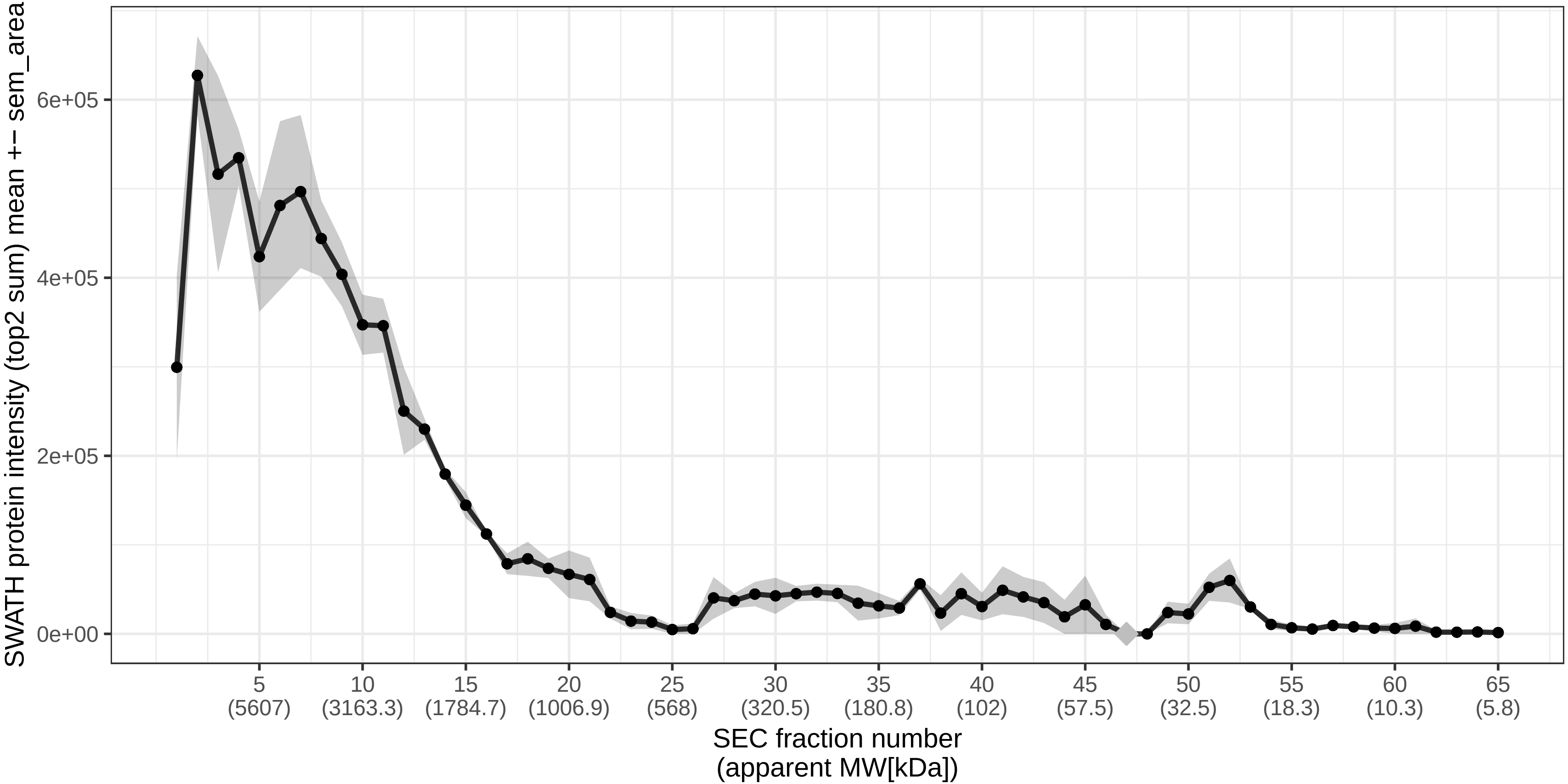

Supplement: Data S1. SEC-SWATH-MS Protein Chromatograms, Related to Figure 1 [file mmc6.zip › SECchrom_O14979_HNRDL_HUMAN_HNRNPDL_HNRPDL_JKTBP.pdf]
