# Supplementary material for: A Global Screen for Assembly State Changes of the Mitotic Proteome by SEC-SWATH-MS
Source: Cell Syst. 2020 Feb 26;10(2):133–155.e6. doi: 10.1016/j.cels.2020.01.001 (PMC7042714; doi:10.1016/j.cels.2020.01.001)

O15047 | SET1A_HUMAN | SETD1A KIAA0339 KMT2F SET1 SET1A
Monomer MW [kDa]: 186.034 Monomer expected elution fraction: 35

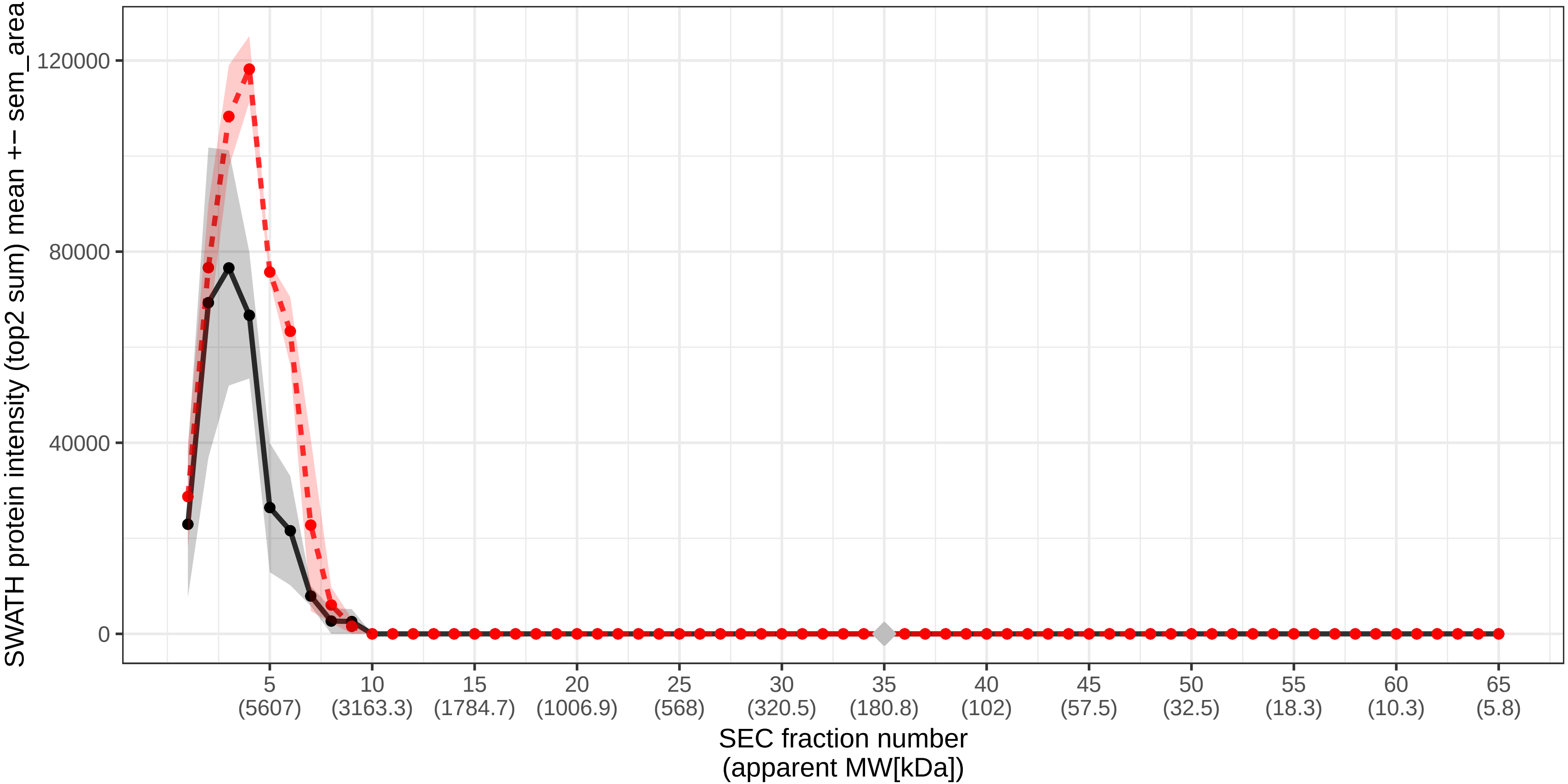

Supplement: Data S1. SEC-SWATH-MS Protein Chromatograms, Related to Figure 1 [file mmc6.zip › SECchrom_O15047_SET1A_HUMAN_SETD1A_KIAA0339_KMT2F_SET1_SET1.pdf]
