# Supplementary figures and images for: A Global Screen for Assembly State Changes of the Mitotic Proteome by SEC-SWATH-MS (part 2 of 2)
Source: Cell Syst. 2020 Feb 26;10(2):133–155.e6. doi: 10.1016/j.cels.2020.01.001 (PMC7042714; doi:10.1016/j.cels.2020.01.001)

O15067 | PUR4\_HUMAN | PFAS KIAA0361

Monomer MW [kDa]: 144.734 Monomer expected elution fraction: 37

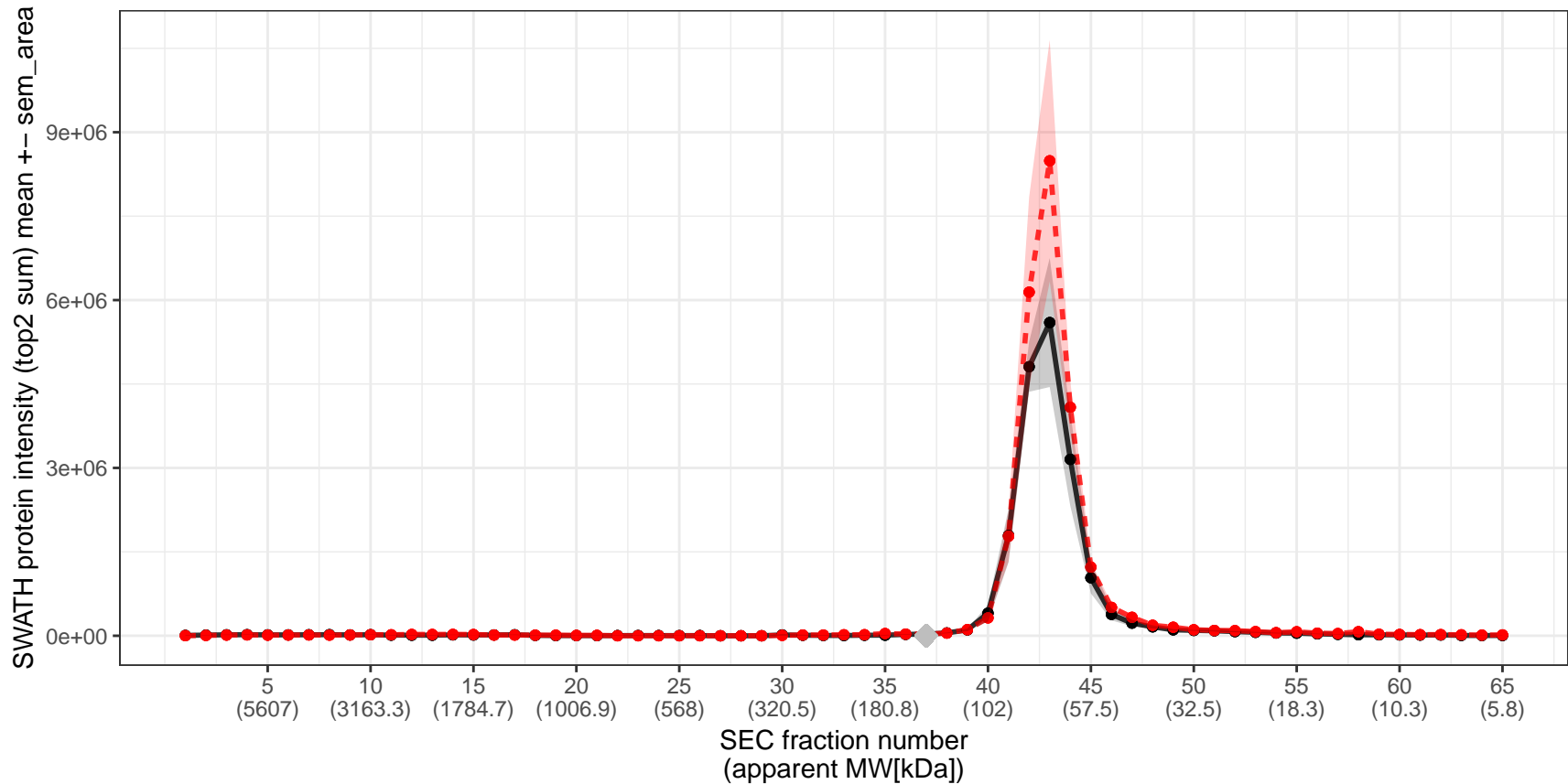

Supplement: Data S1. SEC-SWATH-MS Protein Chromatograms, Related to Figure 1 [file mmc6.zip › SECchrom_O15067_PUR4_HUMAN_PFAS_KIAA0361.pdf]

O15084 | ANR28\_HUMAN | ANKRD28 KIAA0379

Monomer MW [kDa]: 112.966 Monomer expected elution fraction: 39

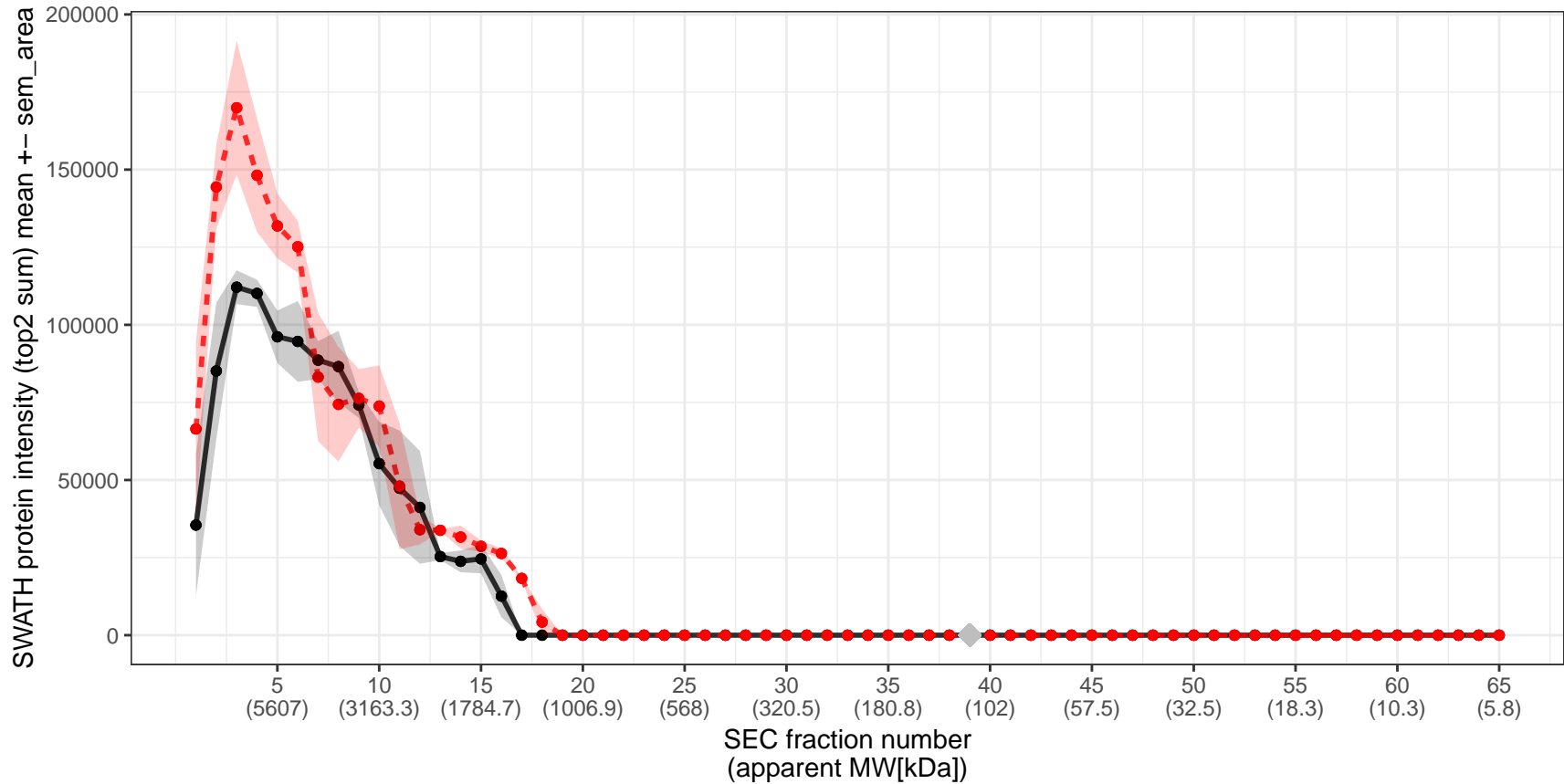

Supplement: Data S1. SEC-SWATH-MS Protein Chromatograms, Related to Figure 1 [file mmc6.zip › SECchrom_O15084_ANR28_HUMAN_ANKRD28_KIAA0379.pdf]

O15111 | IKKA\_HUMAN | CHUK IKKA TCF16

Monomer MW [kDa]: 84.64 Monomer expected elution fraction: 42

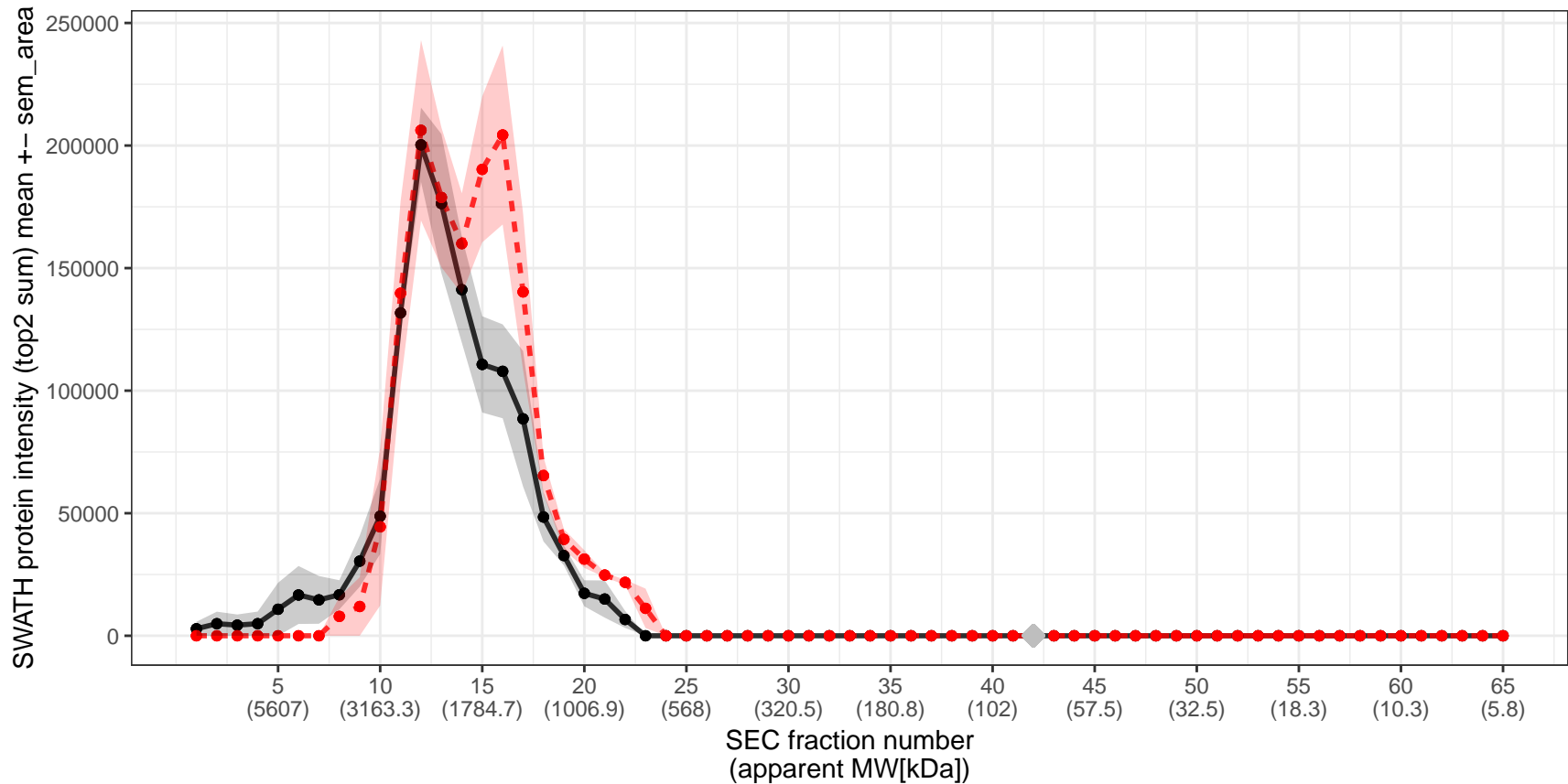

Supplement: Data S1. SEC-SWATH-MS Protein Chromatograms, Related to Figure 1 [file mmc6.zip › SECchrom_O15111_IKKA_HUMAN_CHUK_IKKA_TCF16.pdf]

O15118 | NPC1\_HUMAN | NPC1

Monomer MW [kDa]: 142.167 Monomer expected elution fraction: 37

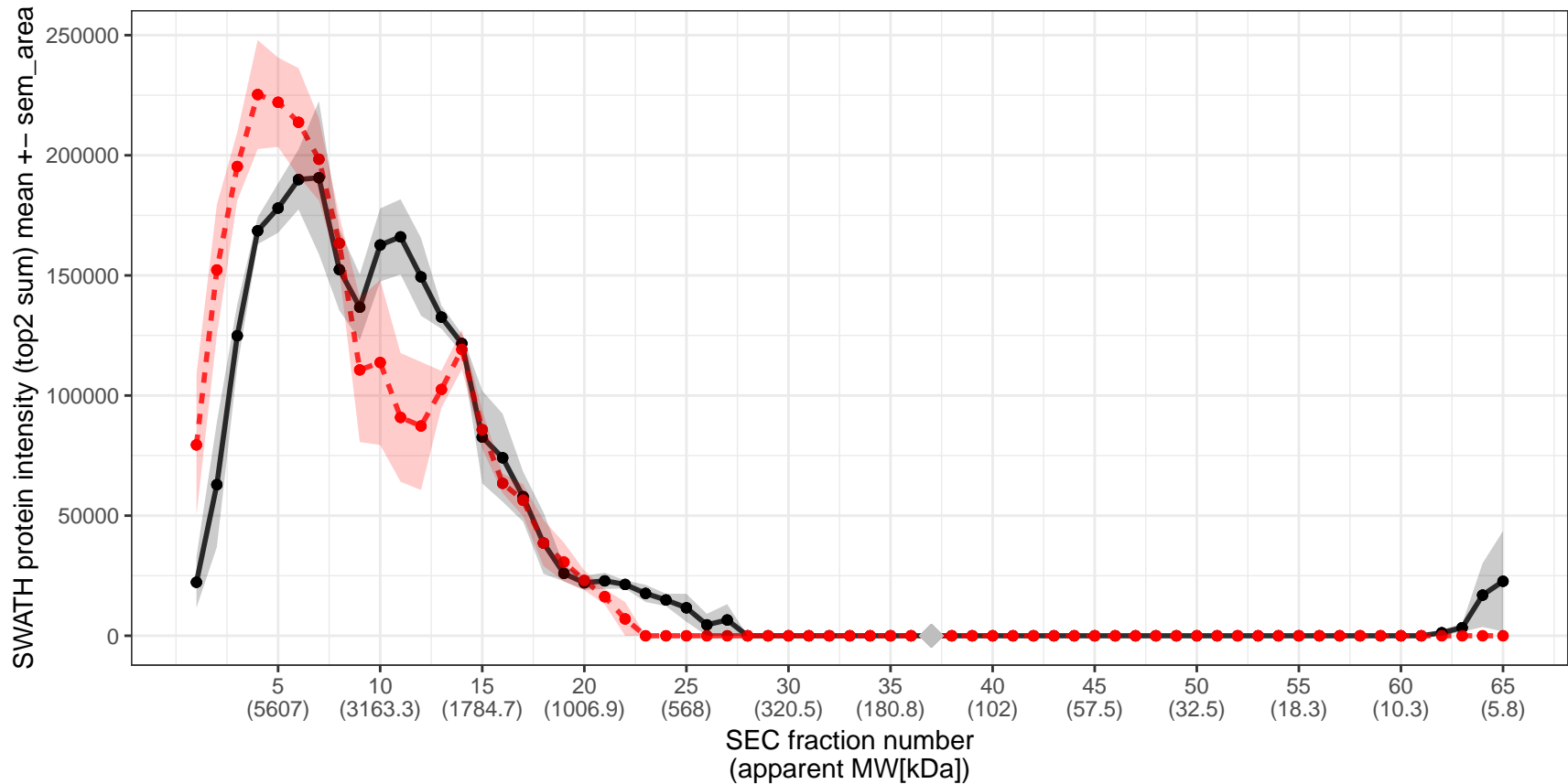

Supplement: Data S1. SEC-SWATH-MS Protein Chromatograms, Related to Figure 1 [file mmc6.zip › SECchrom_O15118_NPC1_HUMAN_NPC1.pdf]

O15126 | SCAM1\_HUMAN | SCAMP1 SCAMP

Monomer MW [kDa]: 37.92 Monomer expected elution fraction: 49

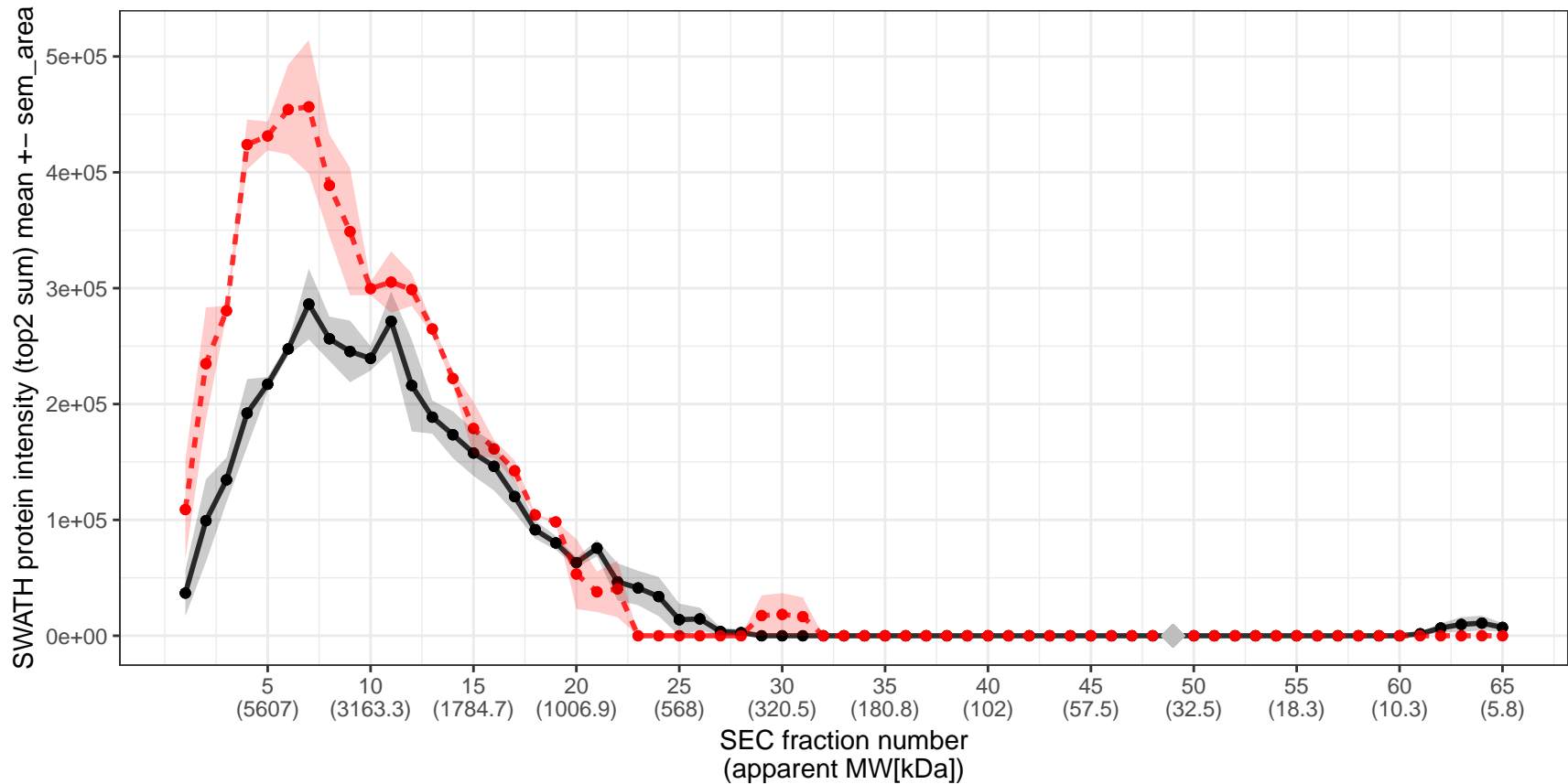

Supplement: Data S1. SEC-SWATH-MS Protein Chromatograms, Related to Figure 1 [file mmc6.zip › SECchrom_O15126_SCAM1_HUMAN_SCAMP1_SCAMP.pdf]

O15145 | ARPC3\_HUMAN | ARPC3 ARC21

Monomer MW [kDa]: 20.547 Monomer expected elution fraction: 54

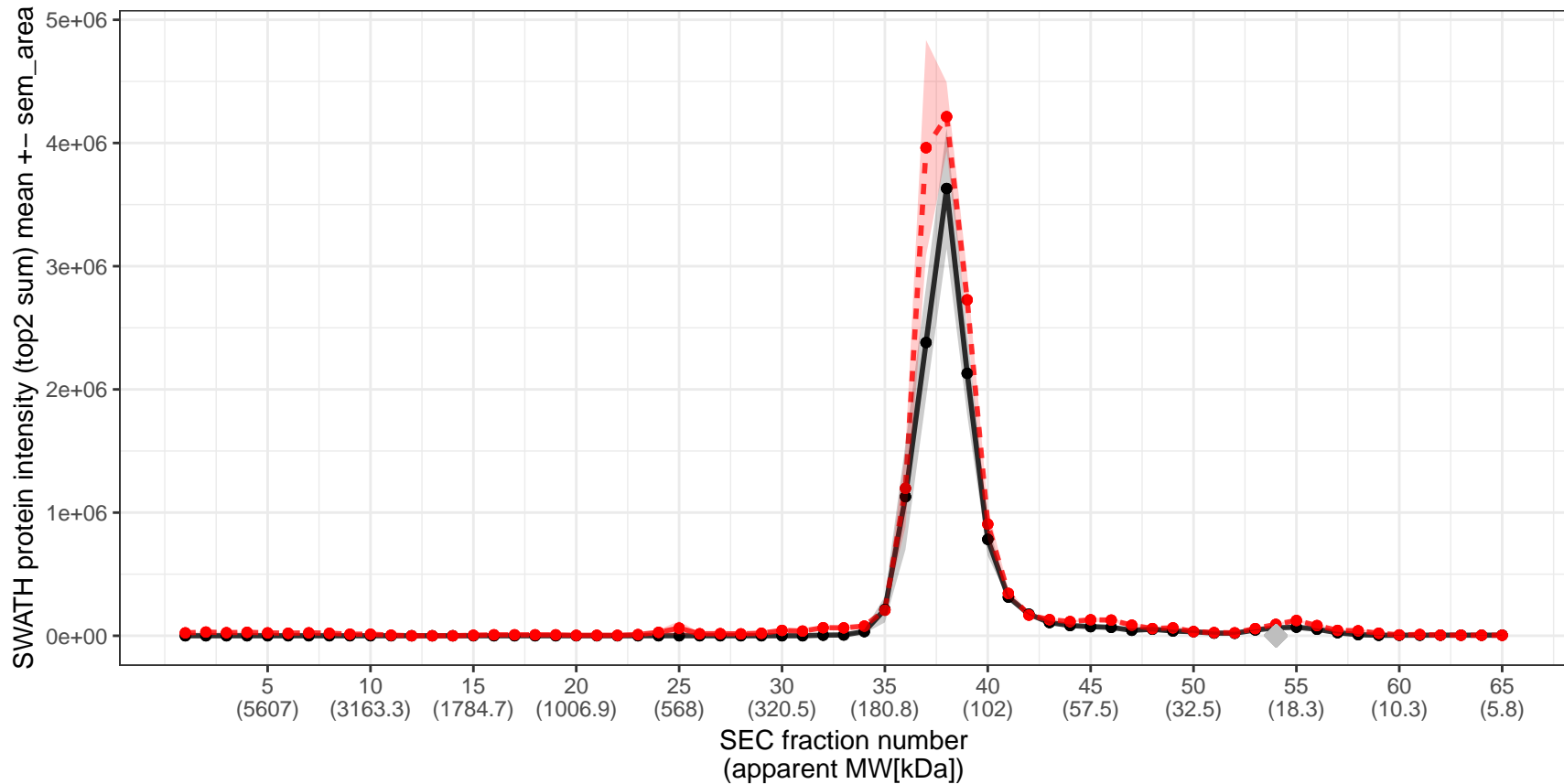

Supplement: Data S1. SEC-SWATH-MS Protein Chromatograms, Related to Figure 1 [file mmc6.zip › SECchrom_O15145_ARPC3_HUMAN_ARPC3_ARC21.pdf]

O15155 | BET1\_HUMAN | BET1

Monomer MW [kDa]: 13.289 Monomer expected elution fraction: 58

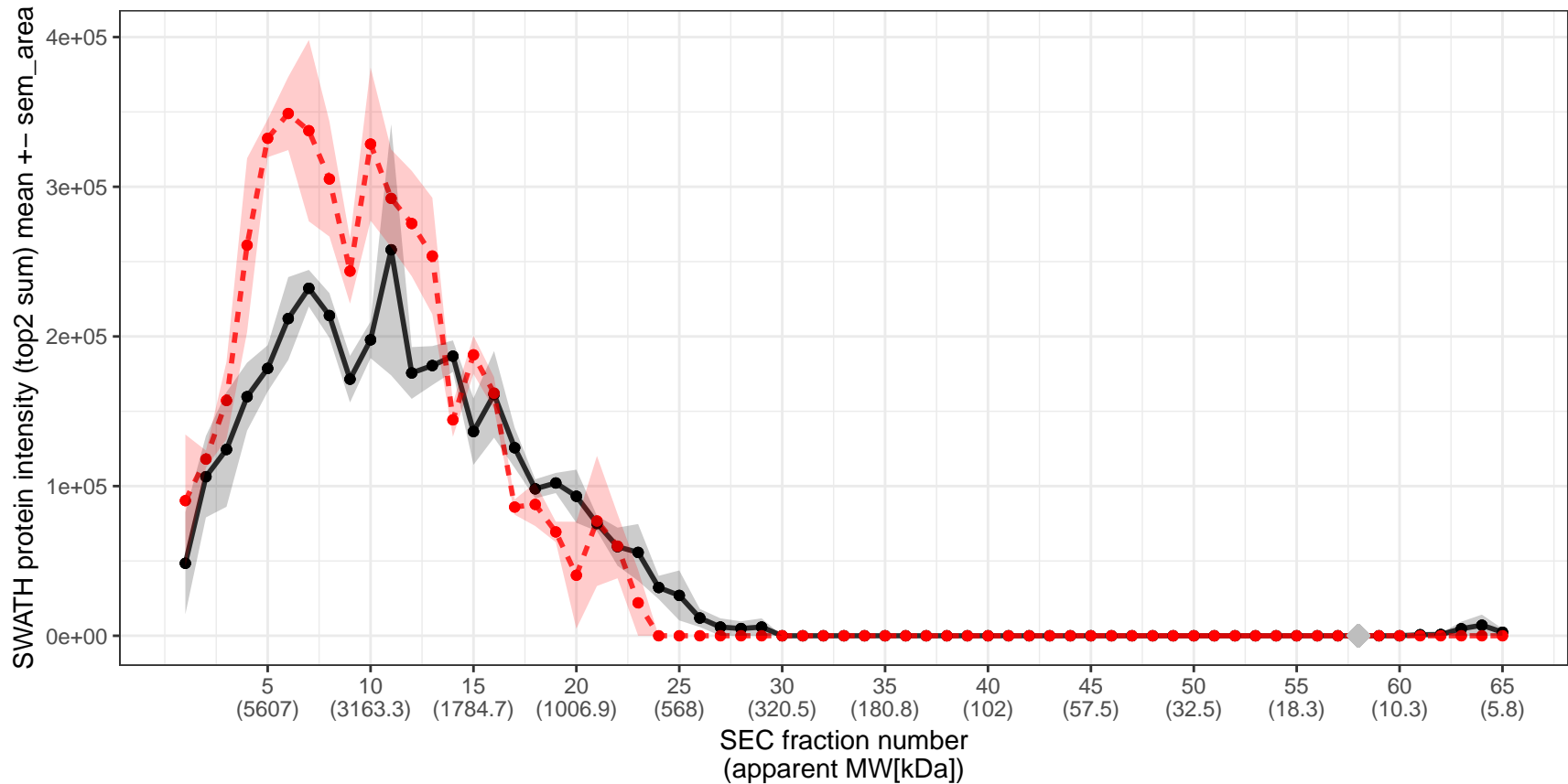

Supplement: Data S1. SEC-SWATH-MS Protein Chromatograms, Related to Figure 1 [file mmc6.zip › SECchrom_O15155_BET1_HUMAN_BET1.pdf]

O15160 | RPAC1\_HUMAN | POLR1C POLR1E

Monomer MW [kDa]: 39.25 Monomer expected elution fraction: 48

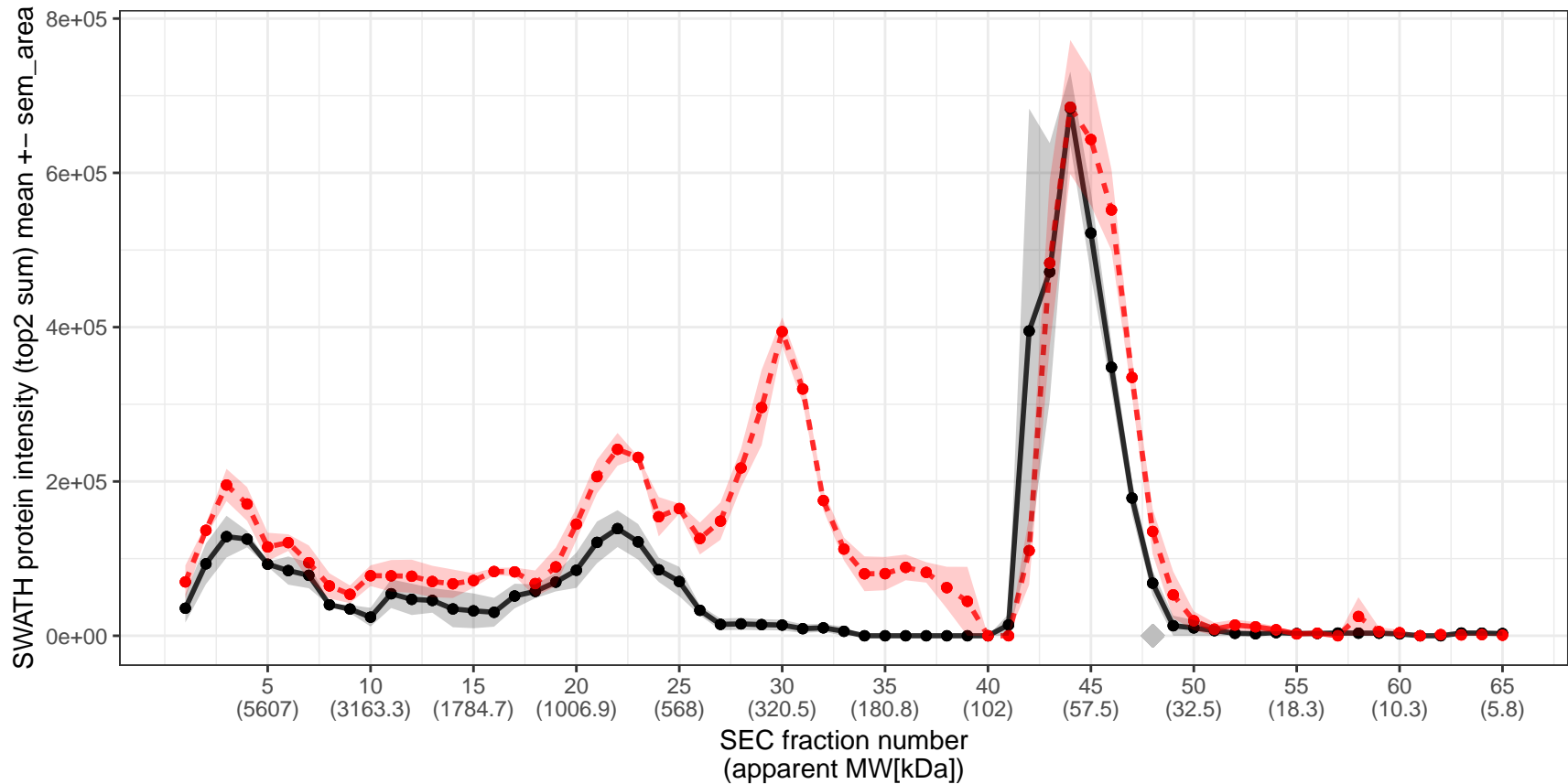

Supplement: Data S1. SEC-SWATH-MS Protein Chromatograms, Related to Figure 1 [file mmc6.zip › SECchrom_O15160_RPAC1_HUMAN_POLR1C_POLR1E.pdf]

O15173 | PGRC2\_HUMAN | PGRMC2 DG6 PMBP

Monomer MW [kDa]: 23.818 Monomer expected elution fraction: 53

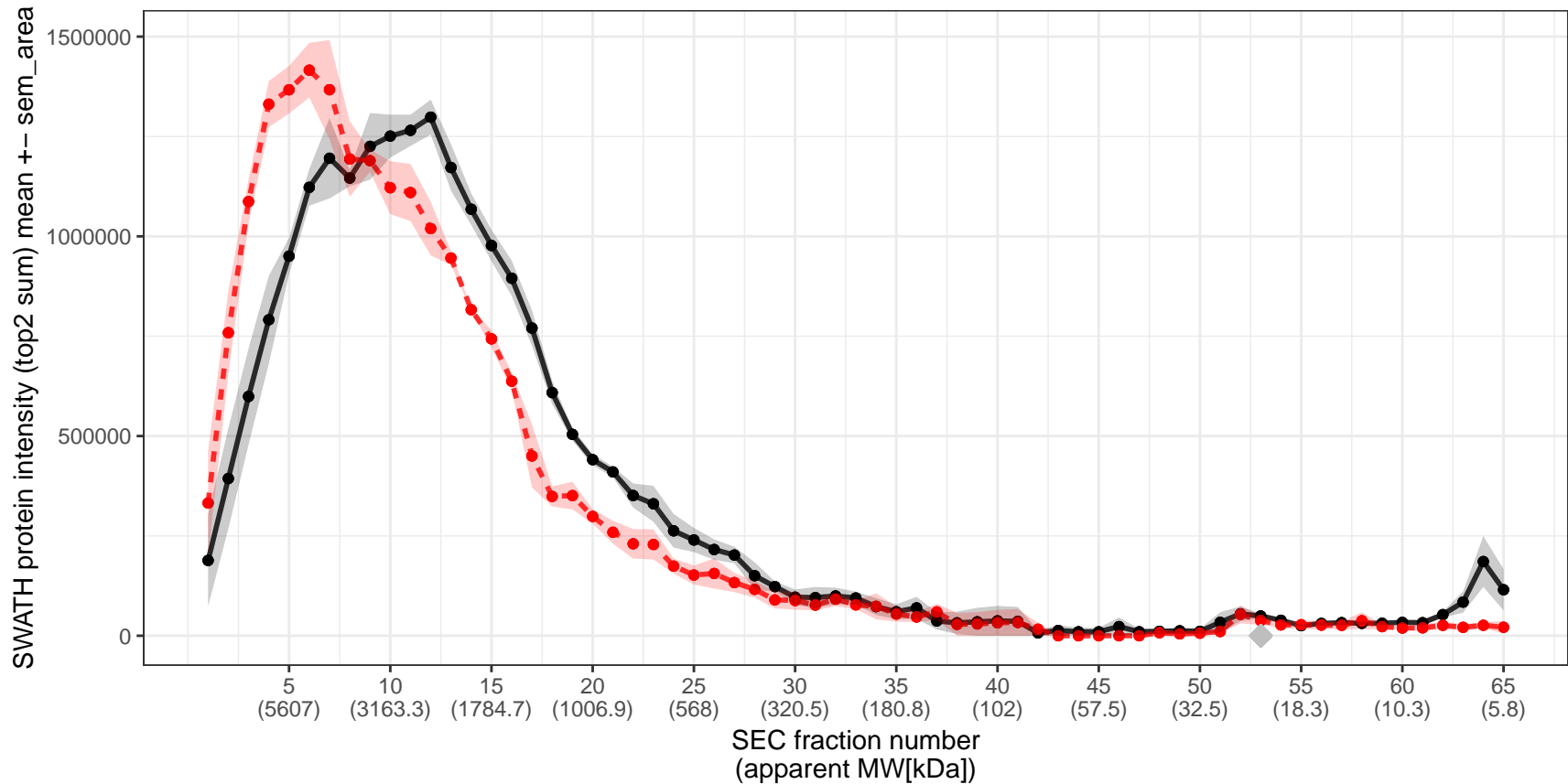

Supplement: Data S1. SEC-SWATH-MS Protein Chromatograms, Related to Figure 1 [file mmc6.zip › SECchrom_O15173_PGRC2_HUMAN_PGRMC2_DG6_PMBP.pdf]

O15212 | PFD6\_HUMAN | PFDN6 HKE2 PFD6  
Monomer MW [kDa]: 14.583 Monomer expected elution fraction: 57

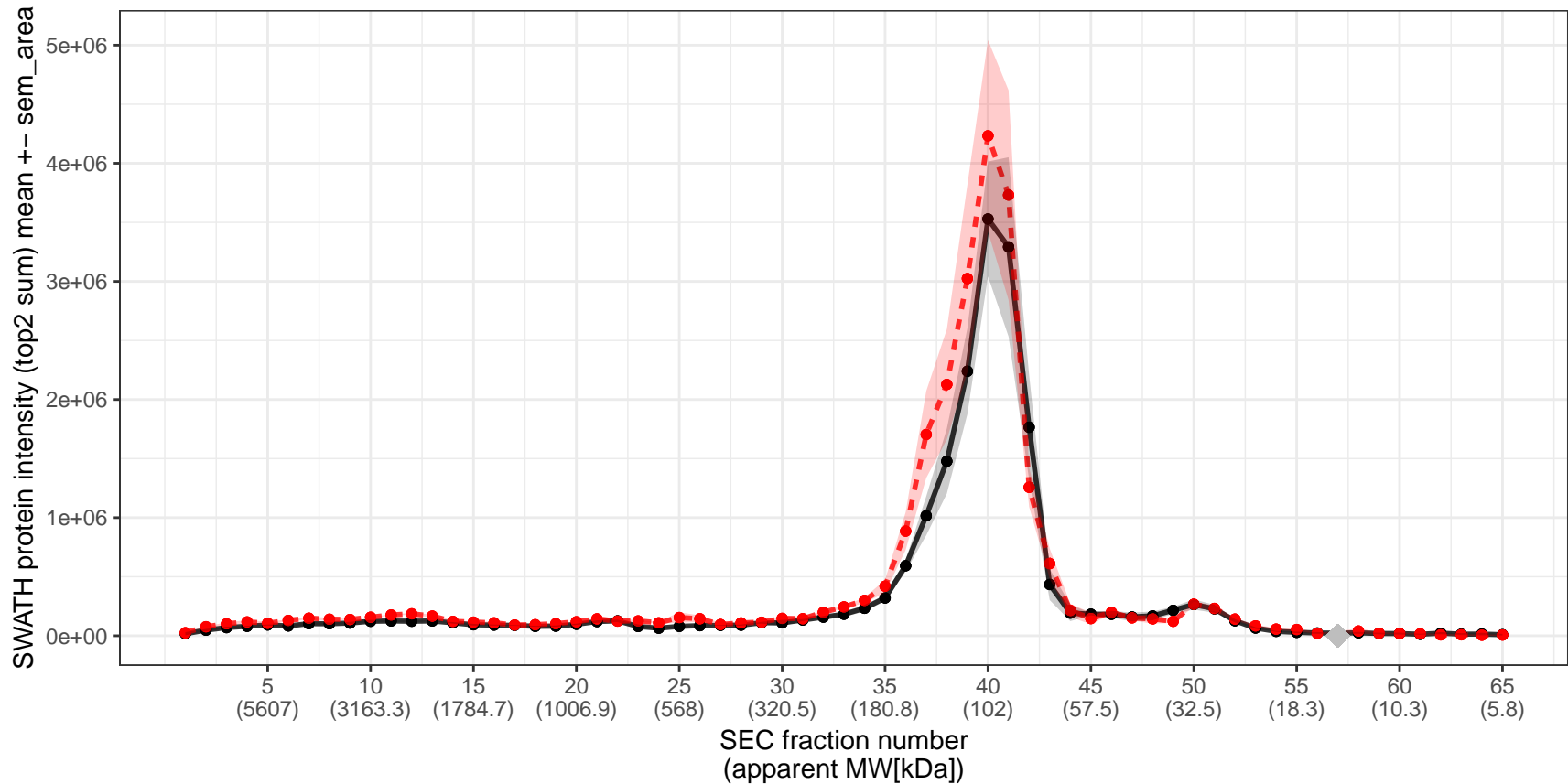

Supplement: Data S1. SEC-SWATH-MS Protein Chromatograms, Related to Figure 1 [file mmc6.zip › SECchrom_O15212_PFD6_HUMAN_PFDN6_HKE2_PFD6.pdf]

O15228 | GNPAT\_HUMAN | GNPAT DAPAT DHAPAT

Monomer MW [kDa]: 77.188 Monomer expected elution fraction: 42

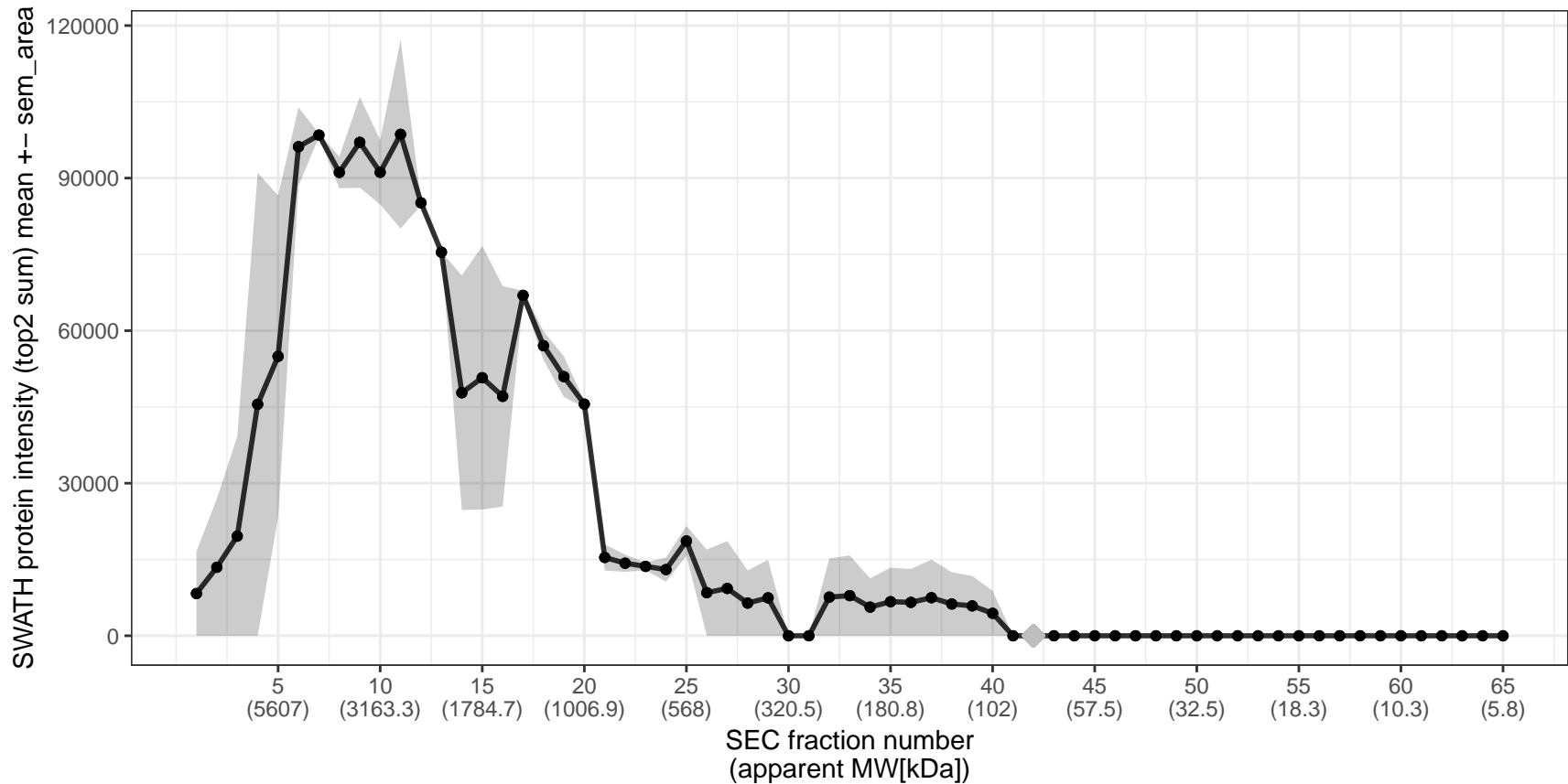

Supplement: Data S1. SEC-SWATH-MS Protein Chromatograms, Related to Figure 1 [file mmc6.zip › SECchrom_O15228_GNPAT_HUMAN_GNPAT_DAPAT_DHAPAT.pdf]

O15254 | ACOX3\_HUMAN | ACOX3 BR/COX PR/COX

Monomer MW [kDa]: 77.629 Monomer expected elution fraction: 42

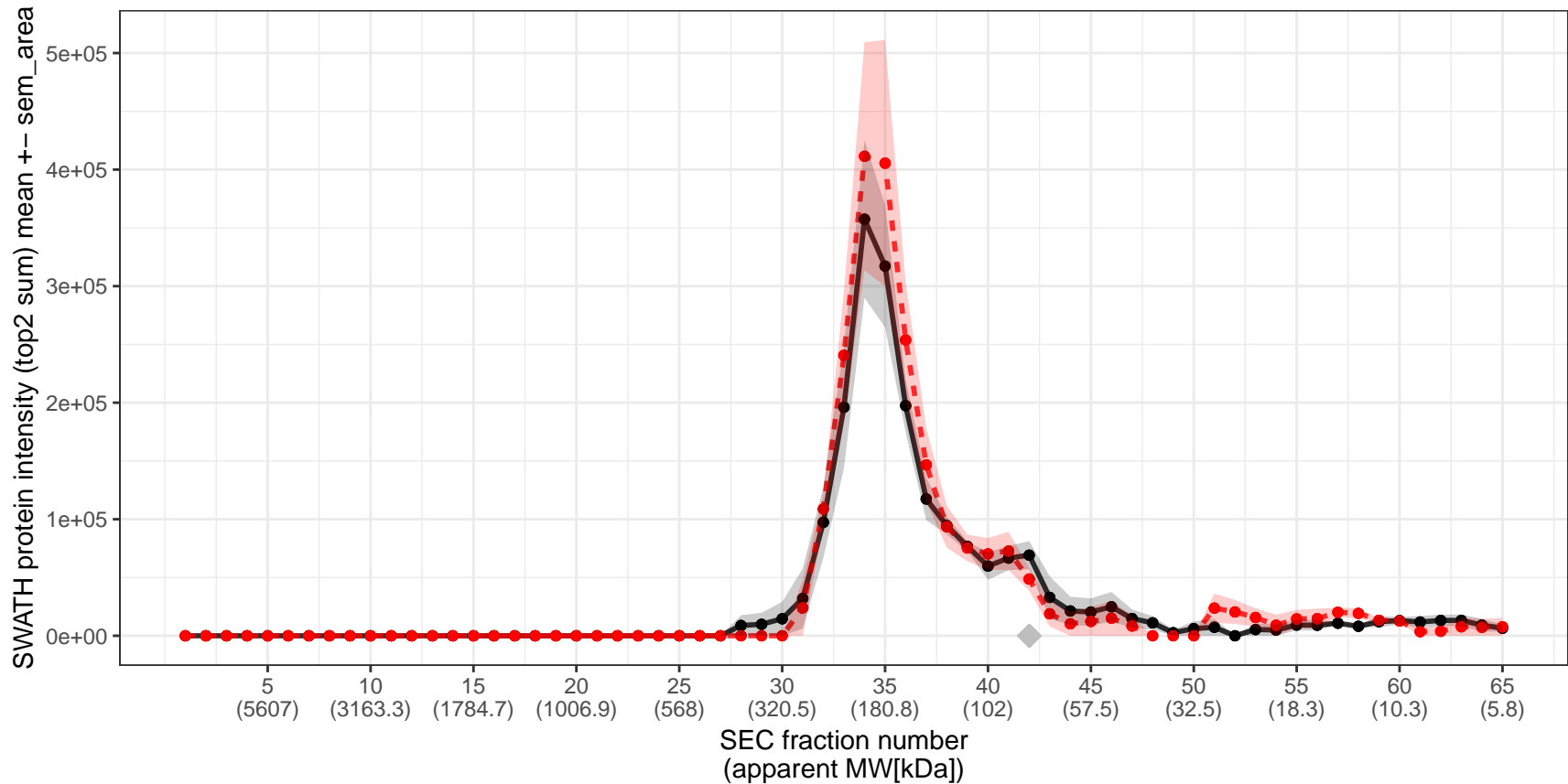

Supplement: Data S1. SEC-SWATH-MS Protein Chromatograms, Related to Figure 1 [file mmc6.zip › SECchrom_O15254_ACOX3_HUMAN_ACOX3_BRCOX_PRCOX.pdf]

O15260 | SURF4\_HUMAN | SURF4 SURF-4

Monomer MW [kDa]: 30.394 Monomer expected elution fraction: 51

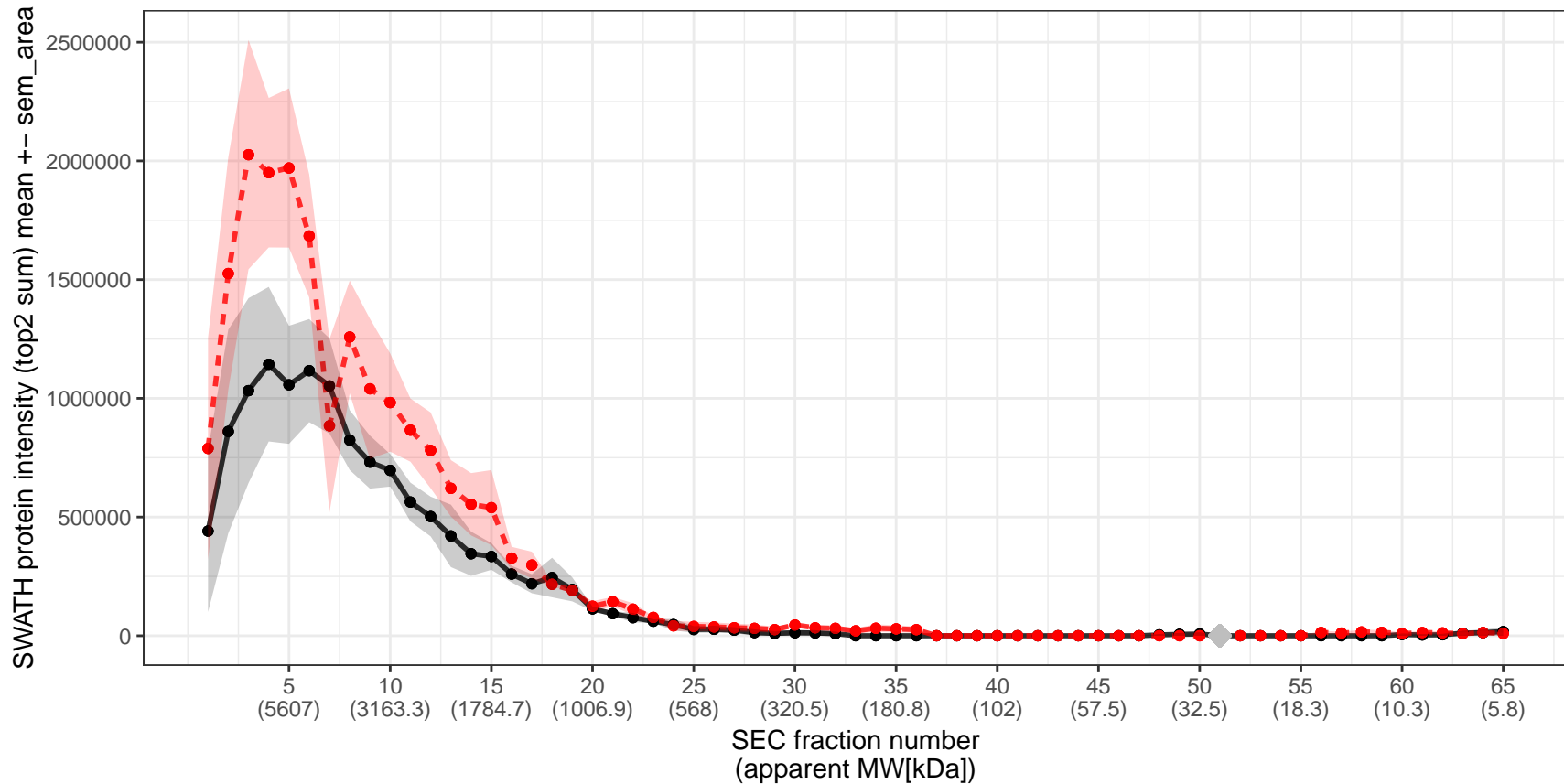

Supplement: Data S1. SEC-SWATH-MS Protein Chromatograms, Related to Figure 1 [file mmc6.zip › SECchrom_O15260_SURF4_HUMAN_SURF4_SURF-4.pdf]

O15294 | OGT1\_HUMAN | OGT

Monomer MW [kDa]: 116.925 Monomer expected elution fraction: 39

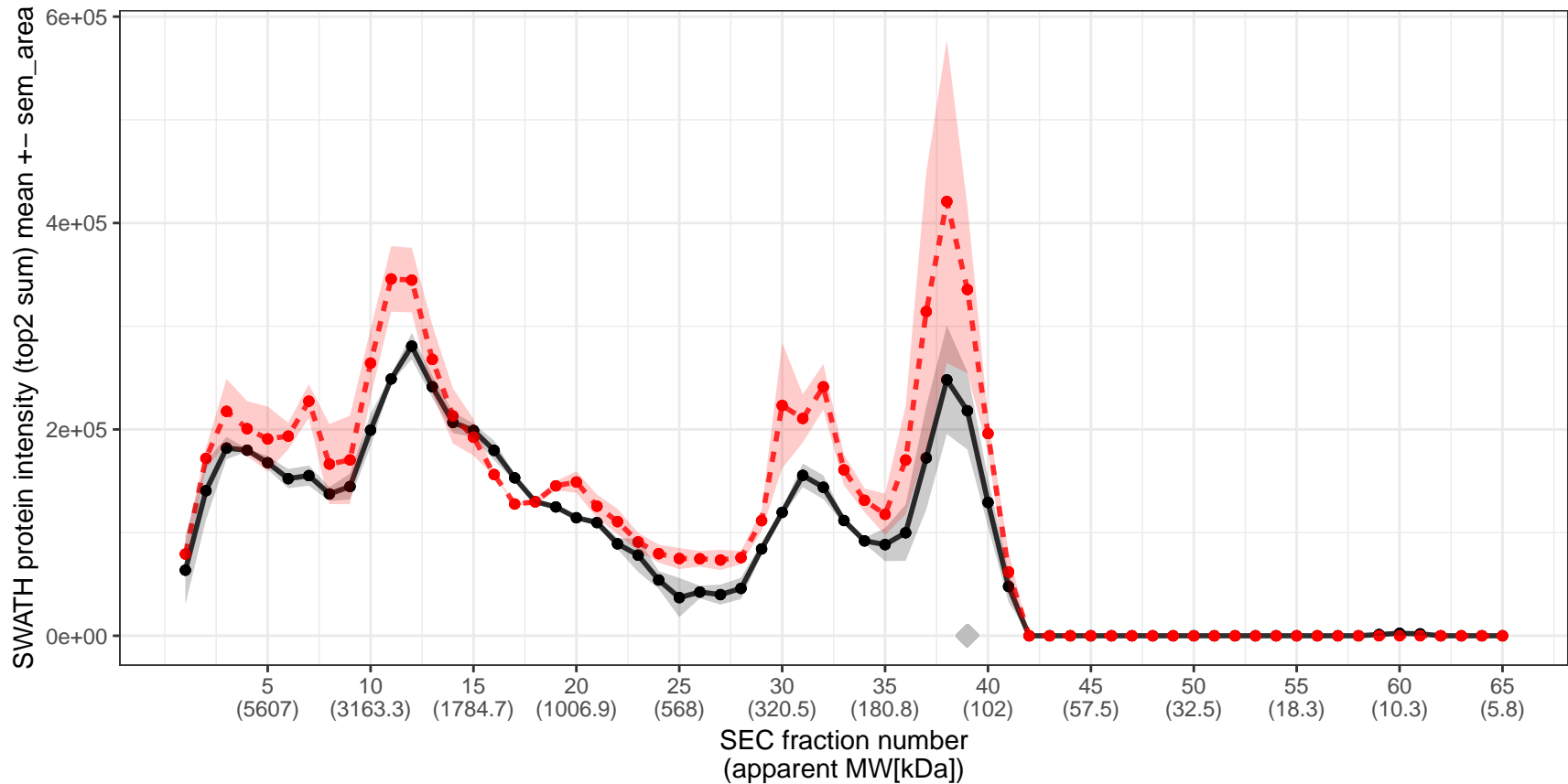

Supplement: Data S1. SEC-SWATH-MS Protein Chromatograms, Related to Figure 1 [file mmc6.zip › SECchrom_O15294_OGT1_HUMAN_OGT.pdf]

O15305 | PMM2\_HUMAN | PMM2

Monomer MW [kDa]: 28.082 Monomer expected elution fraction: 51

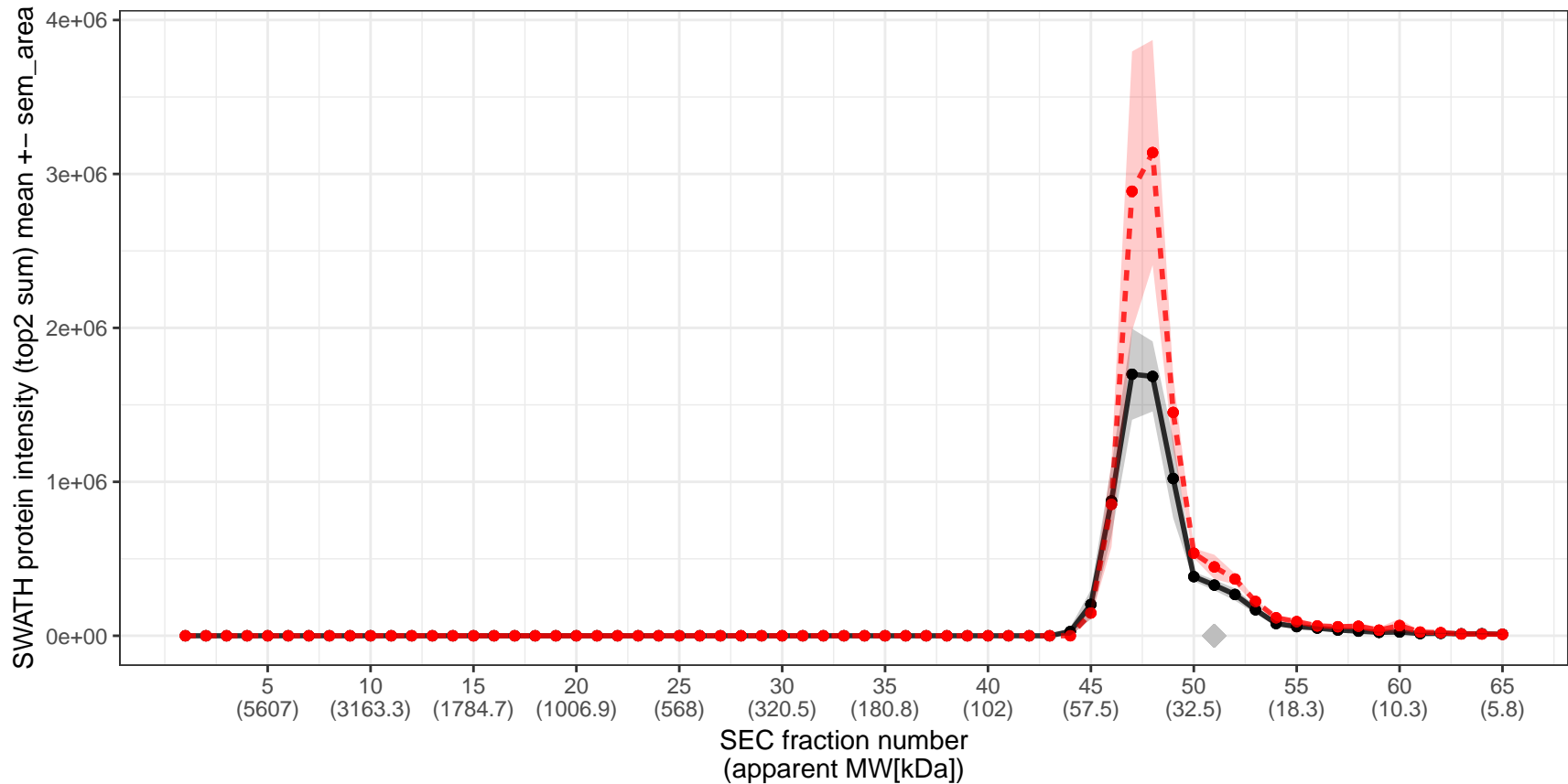

Supplement: Data S1. SEC-SWATH-MS Protein Chromatograms, Related to Figure 1 [file mmc6.zip › SECchrom_O15305_PMM2_HUMAN_PMM2.pdf]

O15318 | RPC7\_HUMAN | POLR3G

Monomer MW [kDa]: 25.914 Monomer expected elution fraction: 52

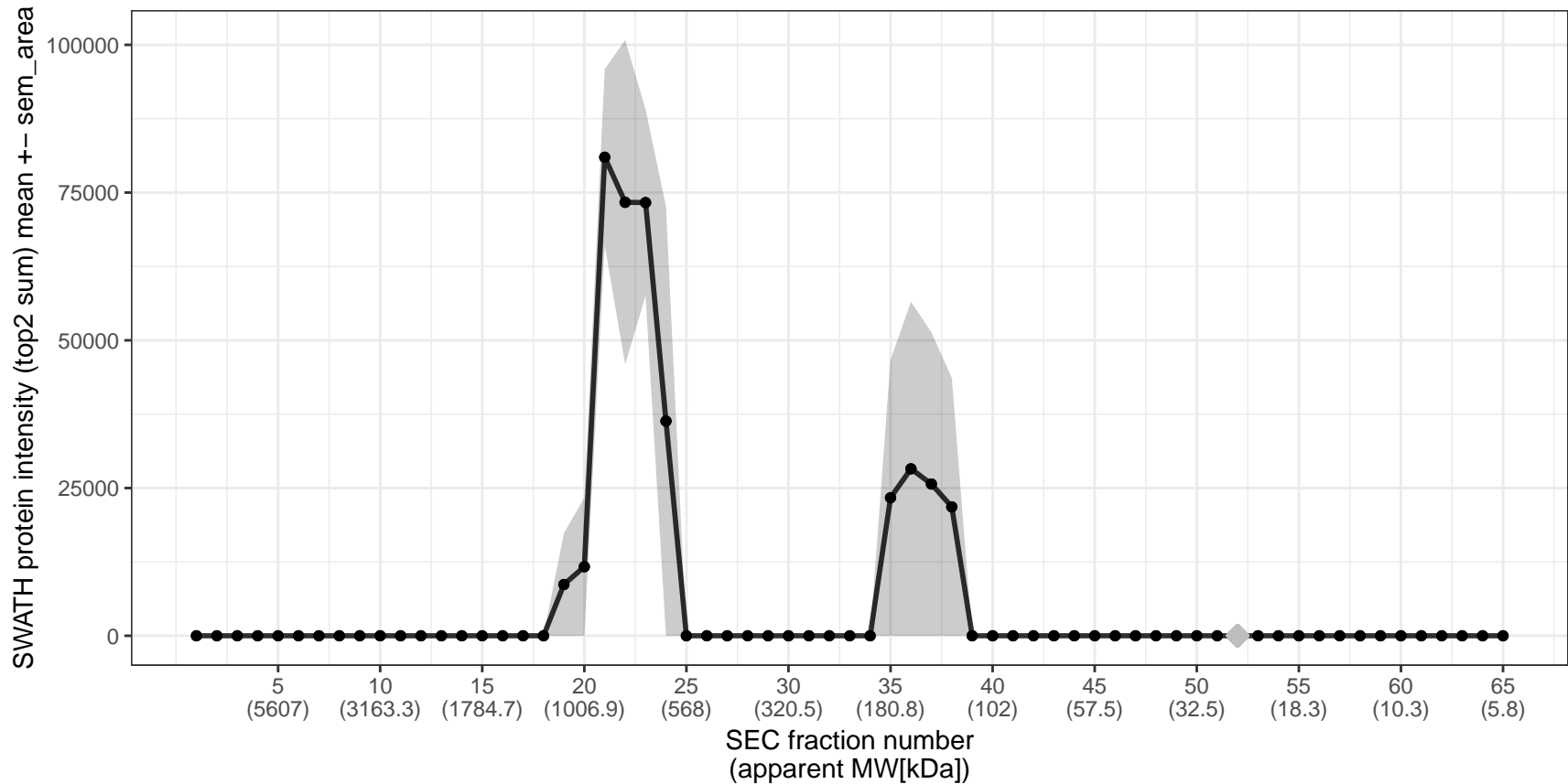

Supplement: Data S1. SEC-SWATH-MS Protein Chromatograms, Related to Figure 1 [file mmc6.zip › SECchrom_O15318_RPC7_HUMAN_POLR3G.pdf]

O15344 | TRI18\_HUMAN | MID1 FXY RNF59 TRIM18 XPRF  
Monomer MW [kDa]: 75.251 Monomer expected elution fraction: 43

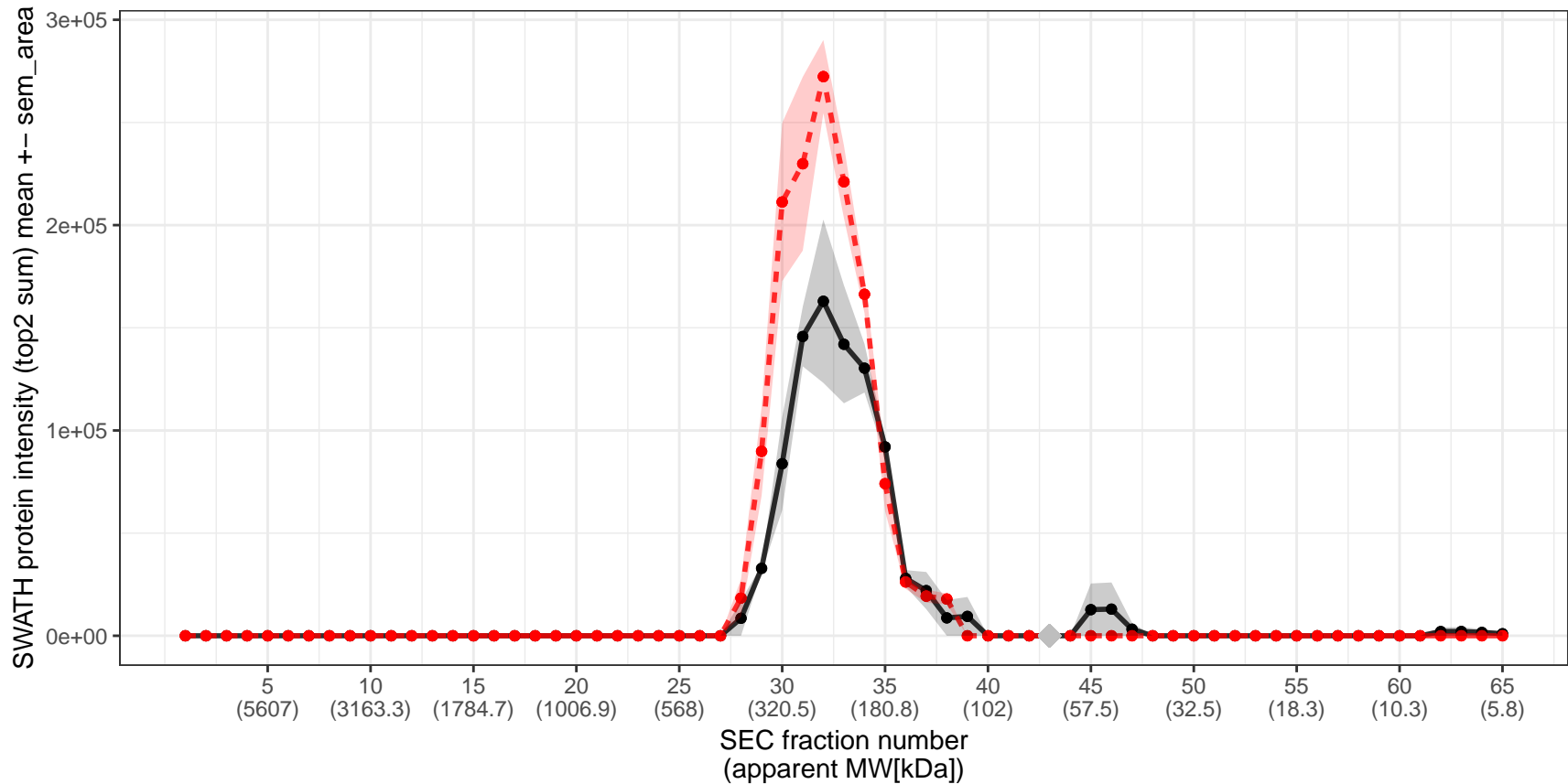

Supplement: Data S1. SEC-SWATH-MS Protein Chromatograms, Related to Figure 1 [file mmc6.zip › SECchrom_O15344_TRI18_HUMAN_MID1_FXY_RNF59_TRIM18_XPRF.pdf]

O15347 | HMGB3\_HUMAN | HMGB3 HMG2A HMG4

Monomer MW [kDa]: 22.98 Monomer expected elution fraction: 53

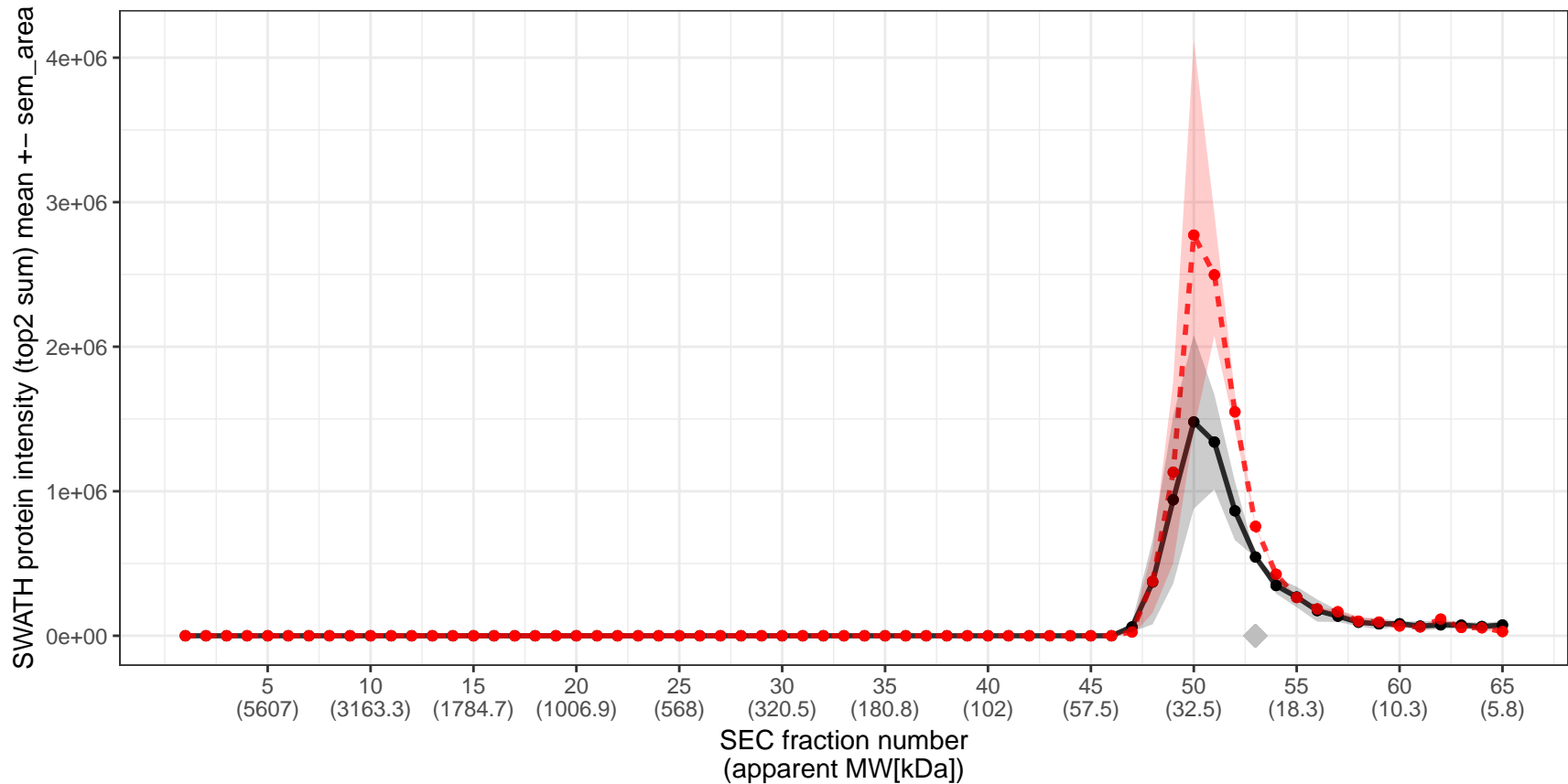

Supplement: Data S1. SEC-SWATH-MS Protein Chromatograms, Related to Figure 1 [file mmc6.zip › SECchrom_O15347_HMGB3_HUMAN_HMGB3_HMG2A_HMG4.pdf]

O15355 | PPM1G\_HUMAN | PPM1G PPM1C

Monomer MW [kDa]: 59.272 Monomer expected elution fraction: 45

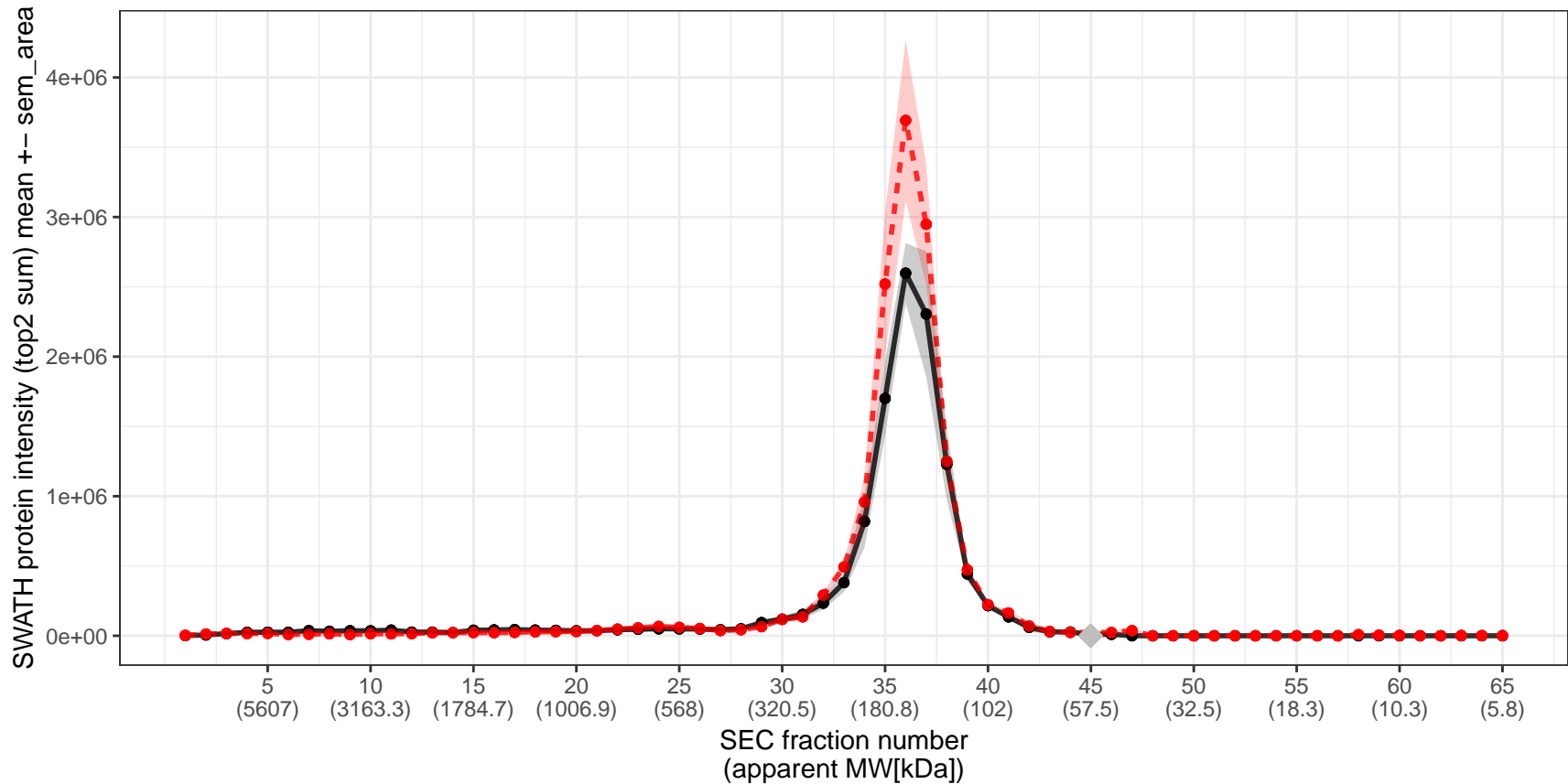

Supplement: Data S1. SEC-SWATH-MS Protein Chromatograms, Related to Figure 1 [file mmc6.zip › SECchrom_O15355_PPM1G_HUMAN_PPM1G_PPM1C.pdf]

O15357 | SHIP2\_HUMAN | INPPL1 SHIP2

Monomer MW [kDa]: 138.599 Monomer expected elution fraction: 37

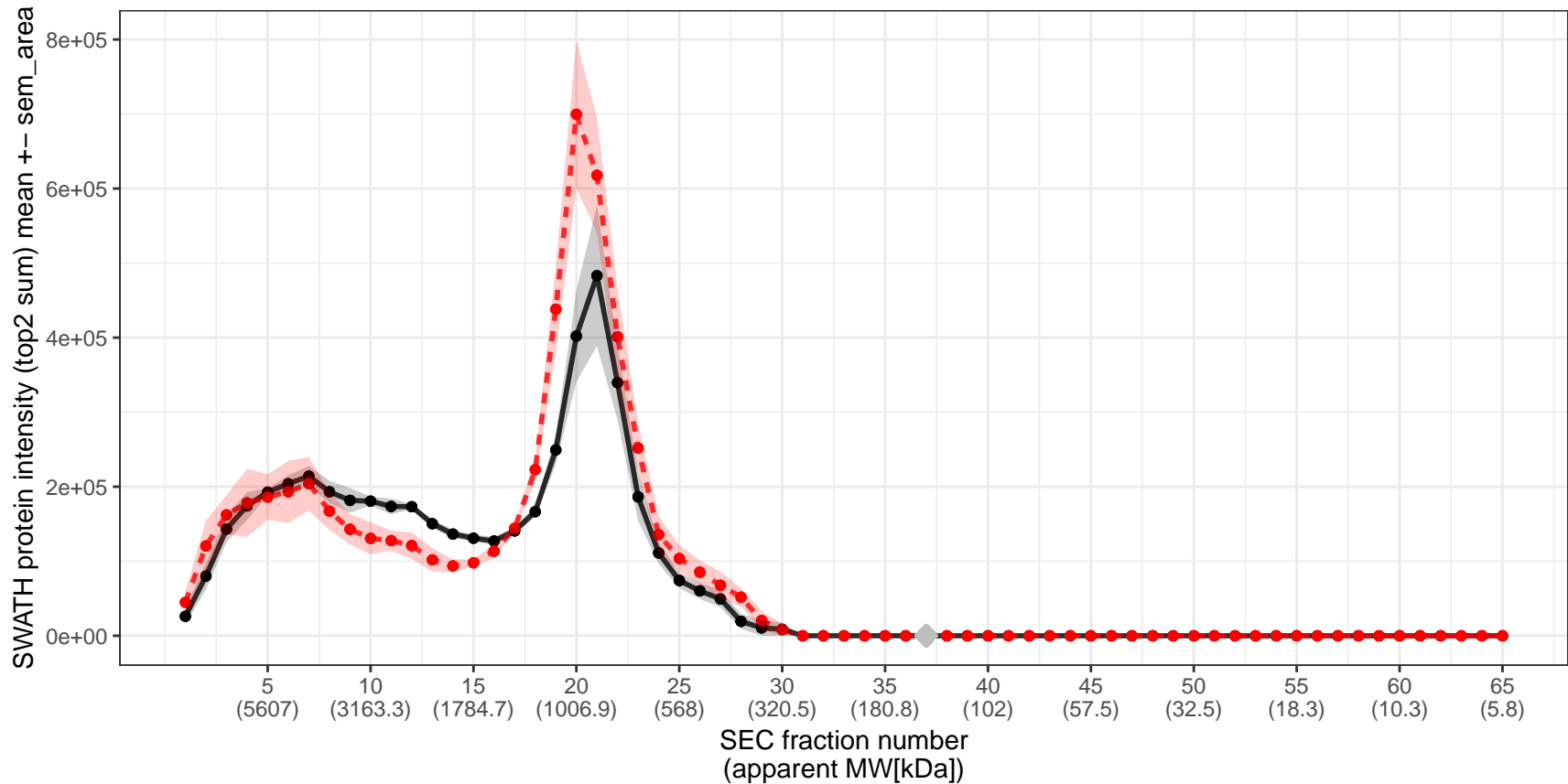

Supplement: Data S1. SEC-SWATH-MS Protein Chromatograms, Related to Figure 1 [file mmc6.zip › SECchrom_O15357_SHIP2_HUMAN_INPPL1_SHIP2.pdf]
