# Supplementary figures and images for: A Global Screen for Assembly State Changes of the Mitotic Proteome by SEC-SWATH-MS (part 1 of 2)
Source: Cell Syst. 2020 Feb 26;10(2):133–155.e6. doi: 10.1016/j.cels.2020.01.001 (PMC7042714; doi:10.1016/j.cels.2020.01.001)

A0FGR8 | ESYT2\_HUMAN | ESYT2 FAM62B KIAA1228

Monomer MW [kDa]: 102.357 Monomer expected elution fraction: 40

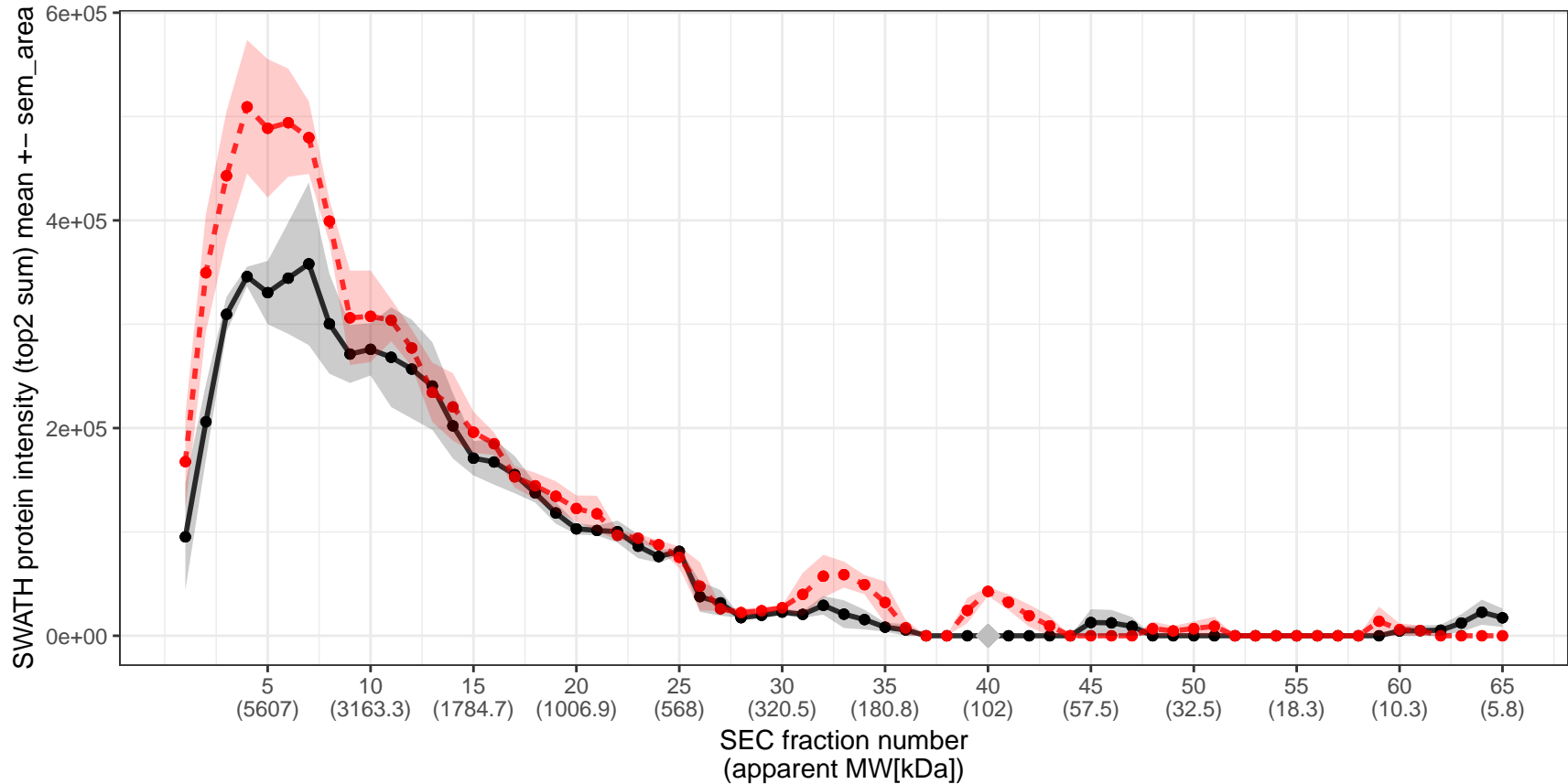

Supplement: Data S1. SEC-SWATH-MS Protein Chromatograms, Related to Figure 1 [file mmc6.zip › SECchrom_A0FGR8_ESYT2_HUMAN_ESYT2_FAM62B_KIAA1228.pdf]

A0MZ66 | SHOT1\_HUMAN | SHTN1 KIAA1598  
Monomer MW [kDa]: 71.64 Monomer expected elution fraction: 43

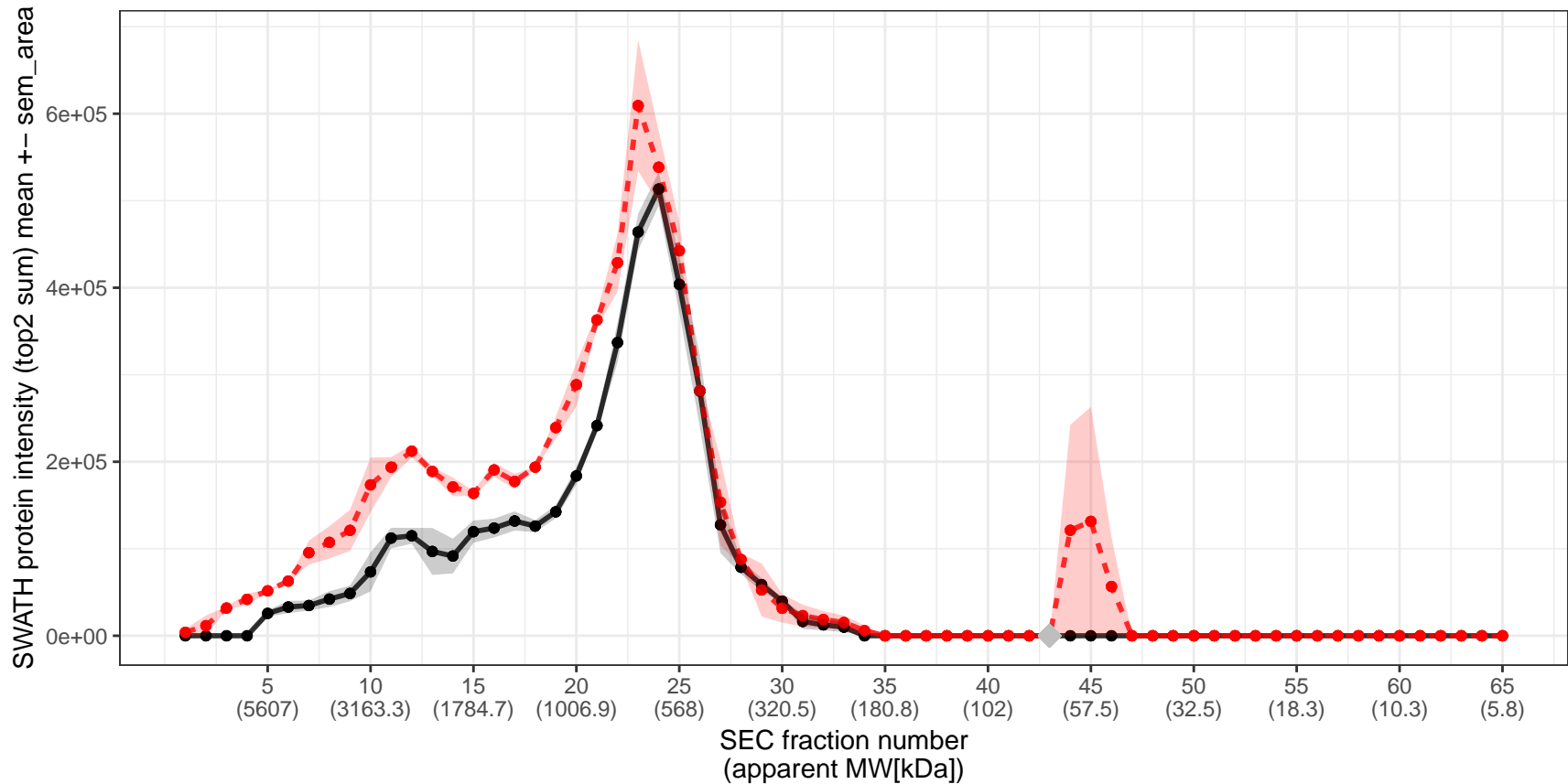

Supplement: Data S1. SEC-SWATH-MS Protein Chromatograms, Related to Figure 1 [file mmc6.zip › SECchrom_A0MZ66_SHOT1_HUMAN_SHTN1_KIAA1598.pdf]

A1L0T0 | ILVBL\_HUMAN | ILVBL AHAS

Monomer MW [kDa]: 67.868 Monomer expected elution fraction: 44

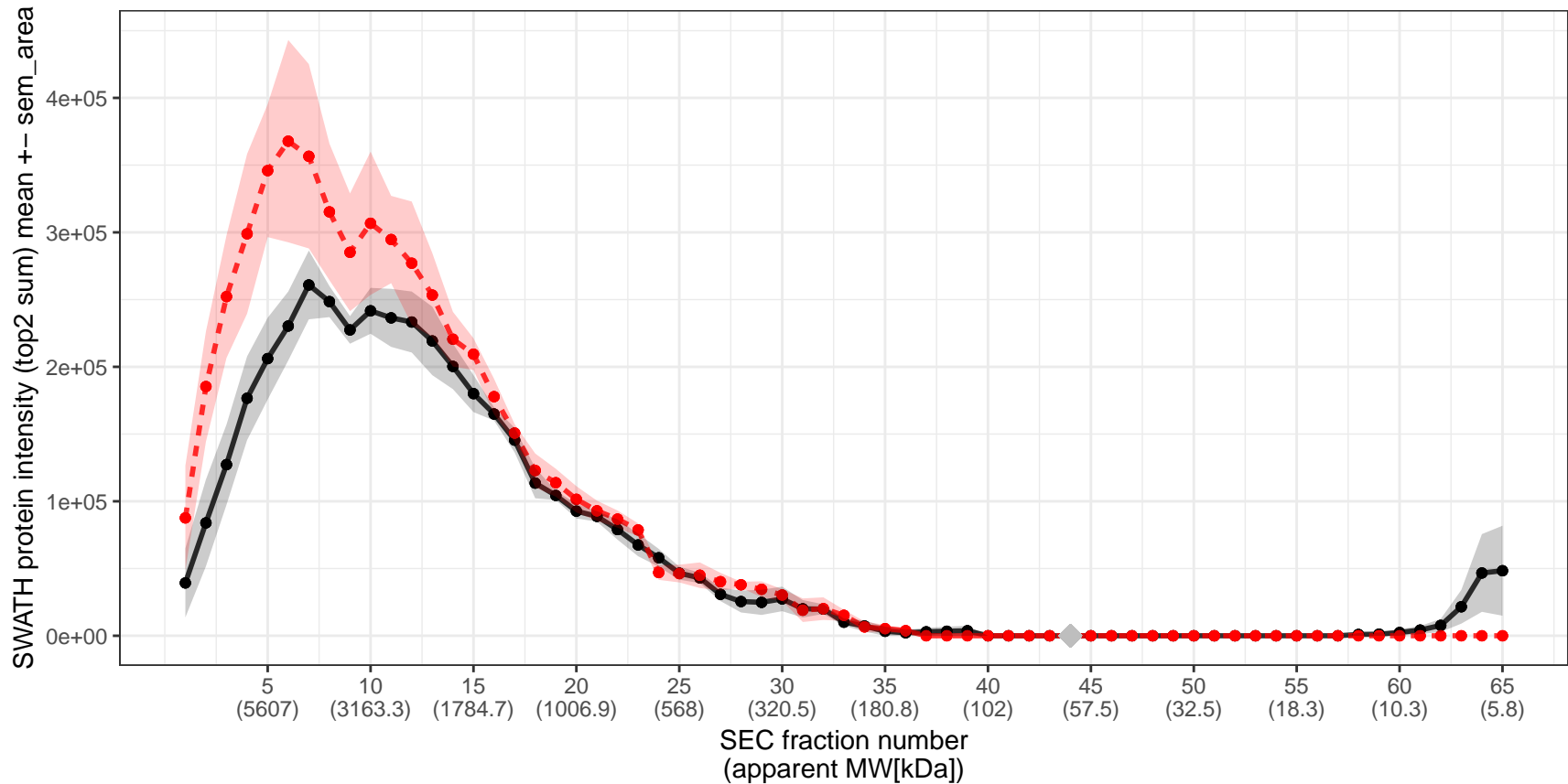

Supplement: Data S1. SEC-SWATH-MS Protein Chromatograms, Related to Figure 1 [file mmc6.zip › SECchrom_A1L0T0_ILVBL_HUMAN_ILVBL_AHAS.pdf]

A1X283 | SPD2B\_HUMAN | SH3PXD2B FAD49 KIAA1295 TKS4

Monomer MW [kDa]: 101.579 Monomer expected elution fraction: 40

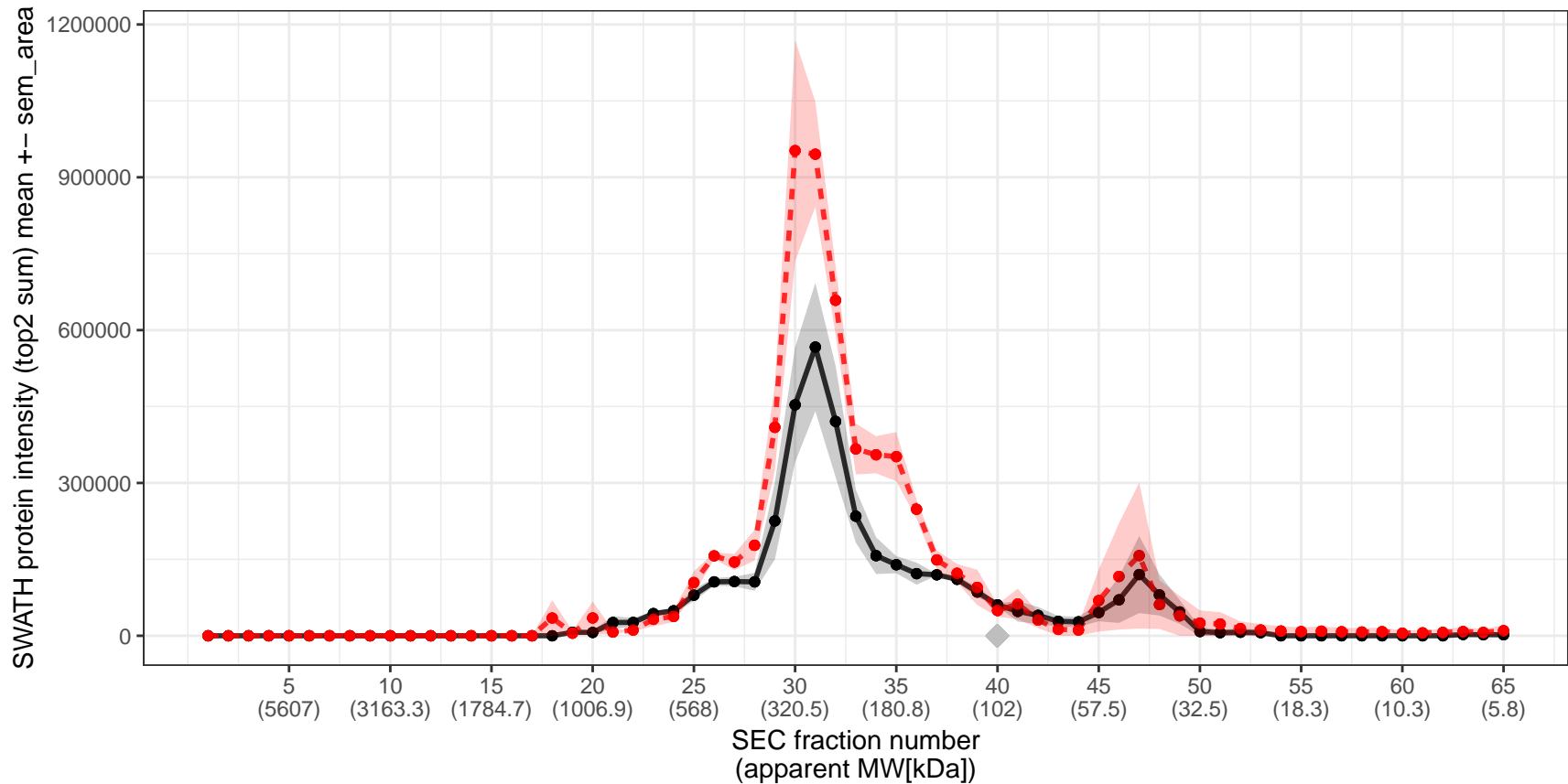

Supplement: Data S1. SEC-SWATH-MS Protein Chromatograms, Related to Figure 1 [file mmc6.zip › SECchrom_A1X283_SPD2B_HUMAN_SH3PXD2B_FAD49_KIAA1295_TKS4.pdf]

A2RU67 | F234B\_HUMAN | FAM234B KIAA1467  
Monomer MW [kDa]: 67.039 Monomer expected elution fraction: 44

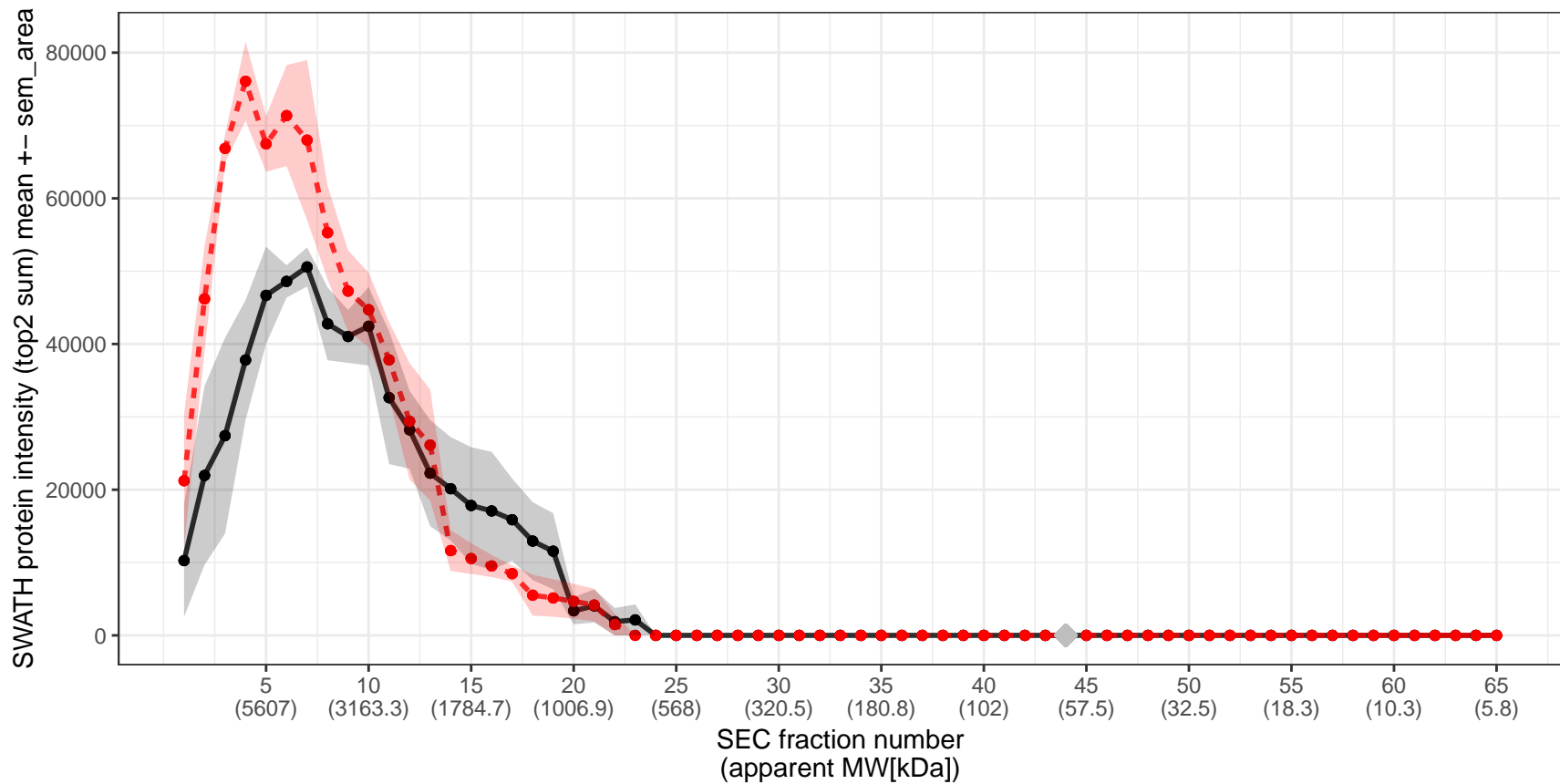

Supplement: Data S1. SEC-SWATH-MS Protein Chromatograms, Related to Figure 1 [file mmc6.zip › SECchrom_A2RU67_F234B_HUMAN_FAM234B_KIAA1467.pdf]

A2RUC4 | TYW5\_HUMAN | TYW5 C2orf60

Monomer MW [kDa]: 36.548 Monomer expected elution fraction: 49

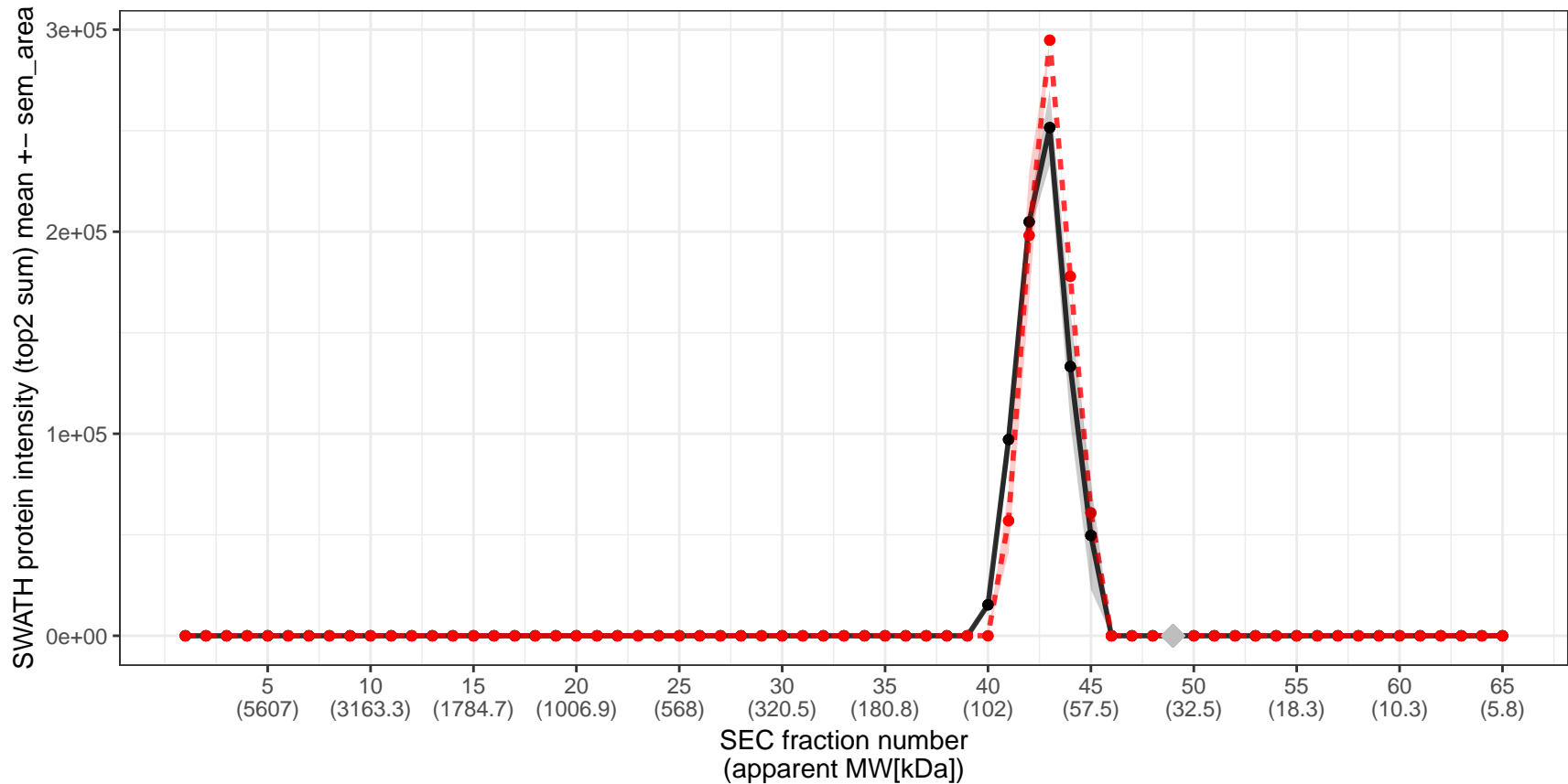

Supplement: Data S1. SEC-SWATH-MS Protein Chromatograms, Related to Figure 1 [file mmc6.zip › SECchrom_A2RUC4_TYW5_HUMAN_TYW5_C2orf60.pdf]

A3KN83 | SBNO1\_HUMAN | SBNO1 MOP3

Monomer MW [kDa]: 154.312 Monomer expected elution fraction: 36

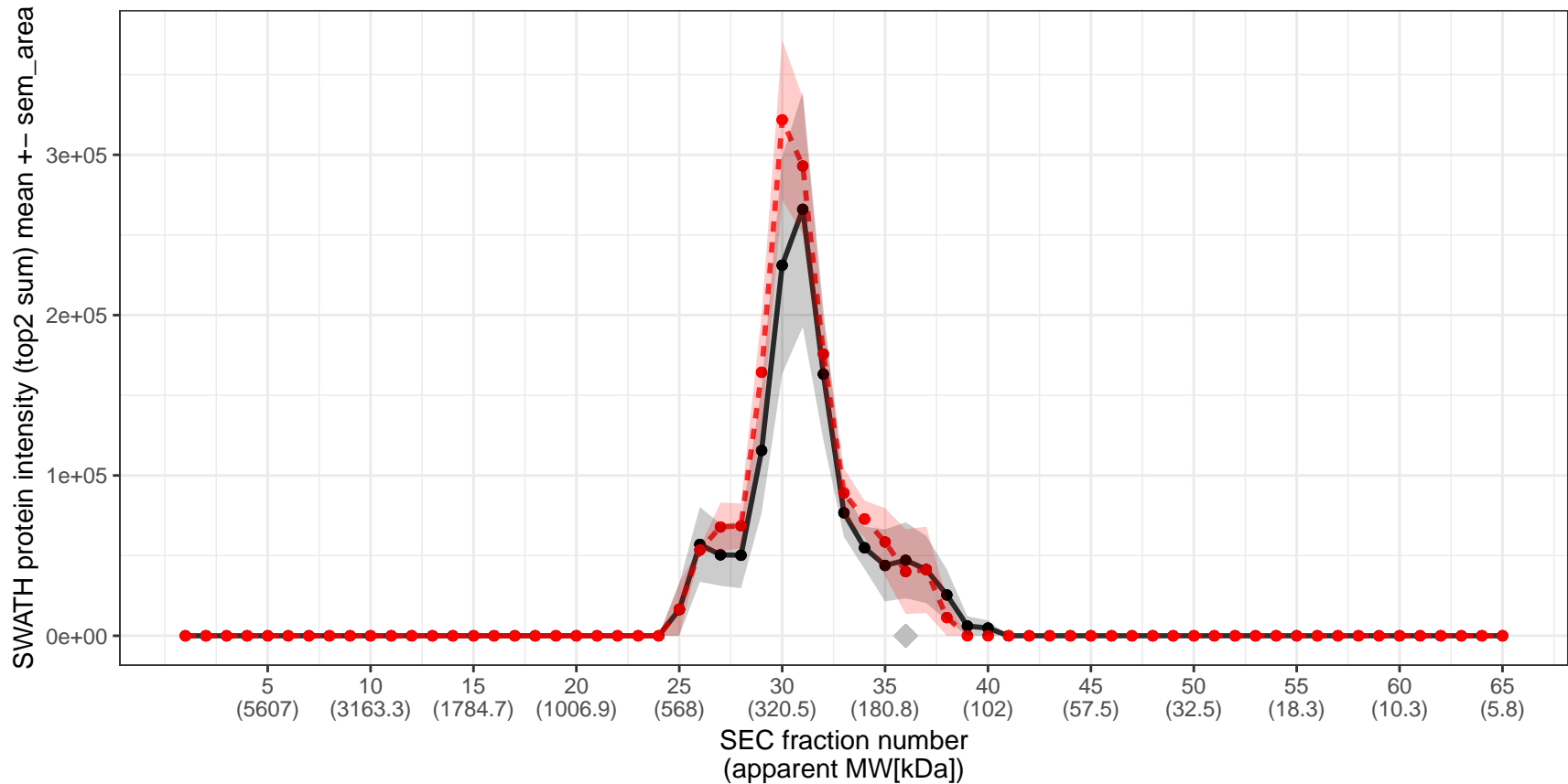

Supplement: Data S1. SEC-SWATH-MS Protein Chromatograms, Related to Figure 1 [file mmc6.zip › SECchrom_A3KN83_SBNO1_HUMAN_SBNO1_MOP3.pdf]

A4D1P6 | WDR91\_HUMAN | WDR91 HSPC049

Monomer MW [kDa]: 83.344 Monomer expected elution fraction: 42

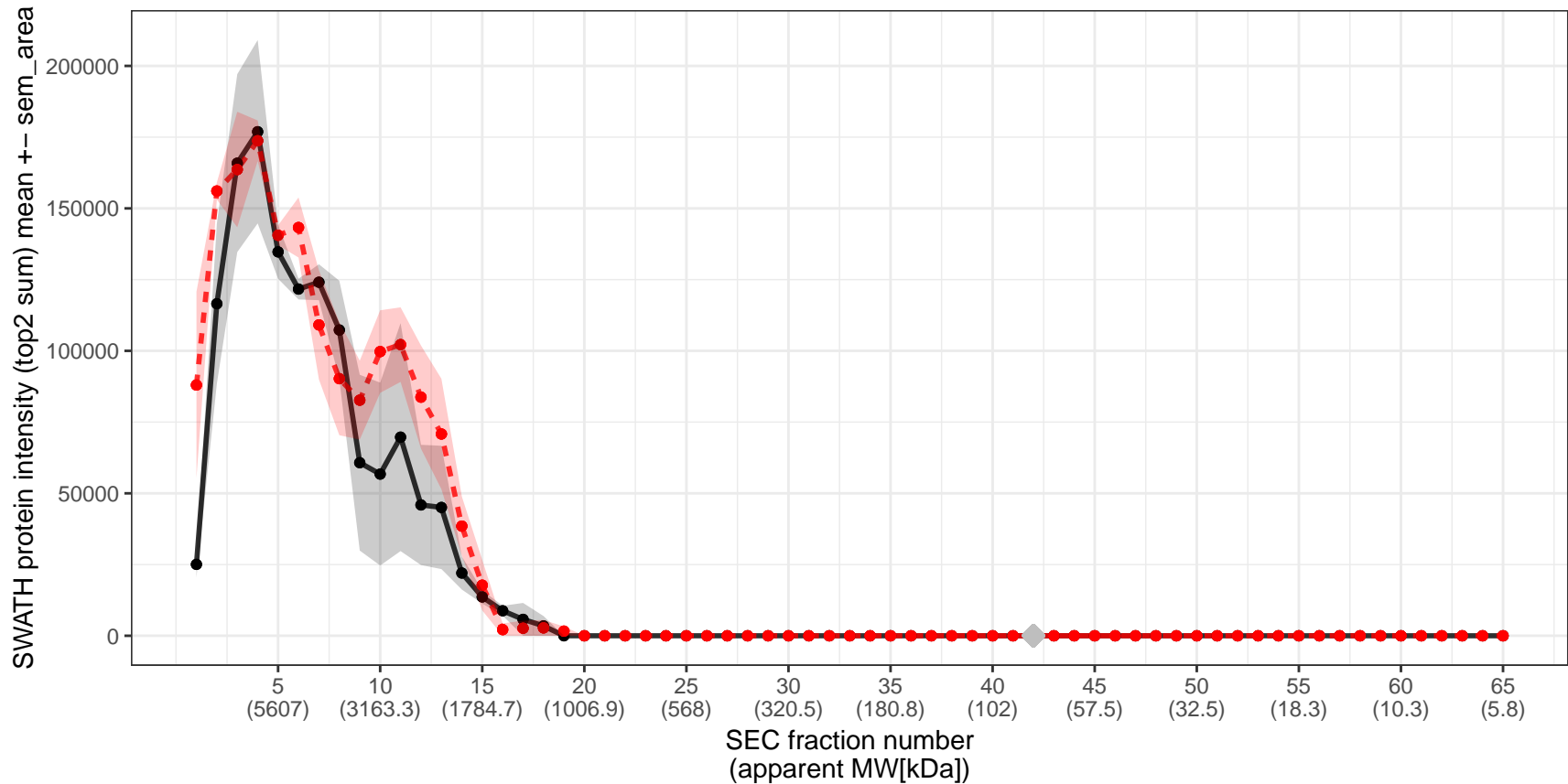

Supplement: Data S1. SEC-SWATH-MS Protein Chromatograms, Related to Figure 1 [file mmc6.zip › SECchrom_A4D1P6_WDR91_HUMAN_WDR91_HSPC049.pdf]

A5D8V6 | VP37C\_HUMAN | VPS37C PML39

Monomer MW [kDa]: 38.659 Monomer expected elution fraction: 48

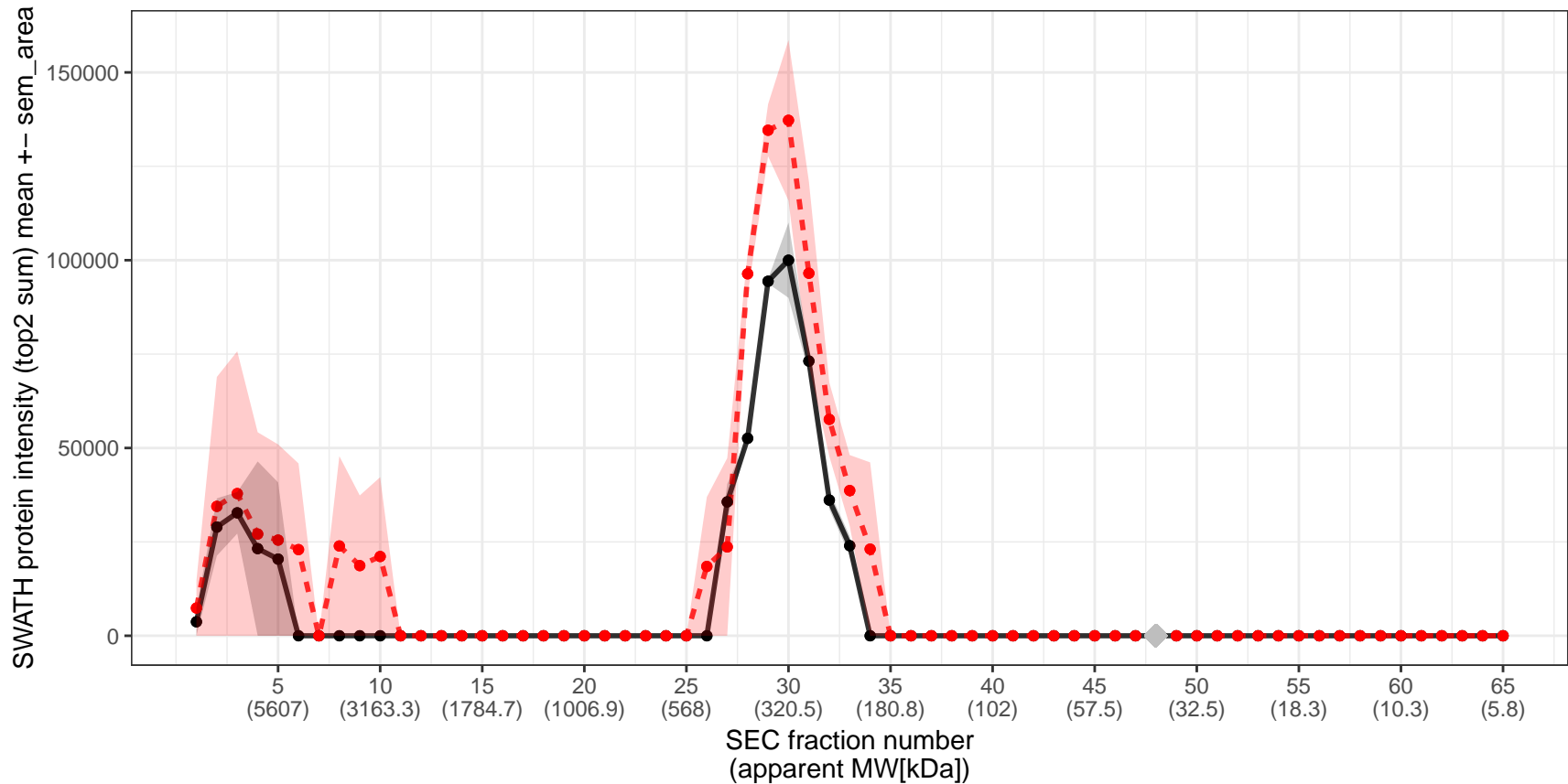

Supplement: Data S1. SEC-SWATH-MS Protein Chromatograms, Related to Figure 1 [file mmc6.zip › SECchrom_A5D8V6_VP37C_HUMAN_VPS37C_PML39.pdf]

A5PLL7 | TM189\_HUMAN | TMEM189 KUA

Monomer MW [kDa]: 31.135 Monomer expected elution fraction: 50

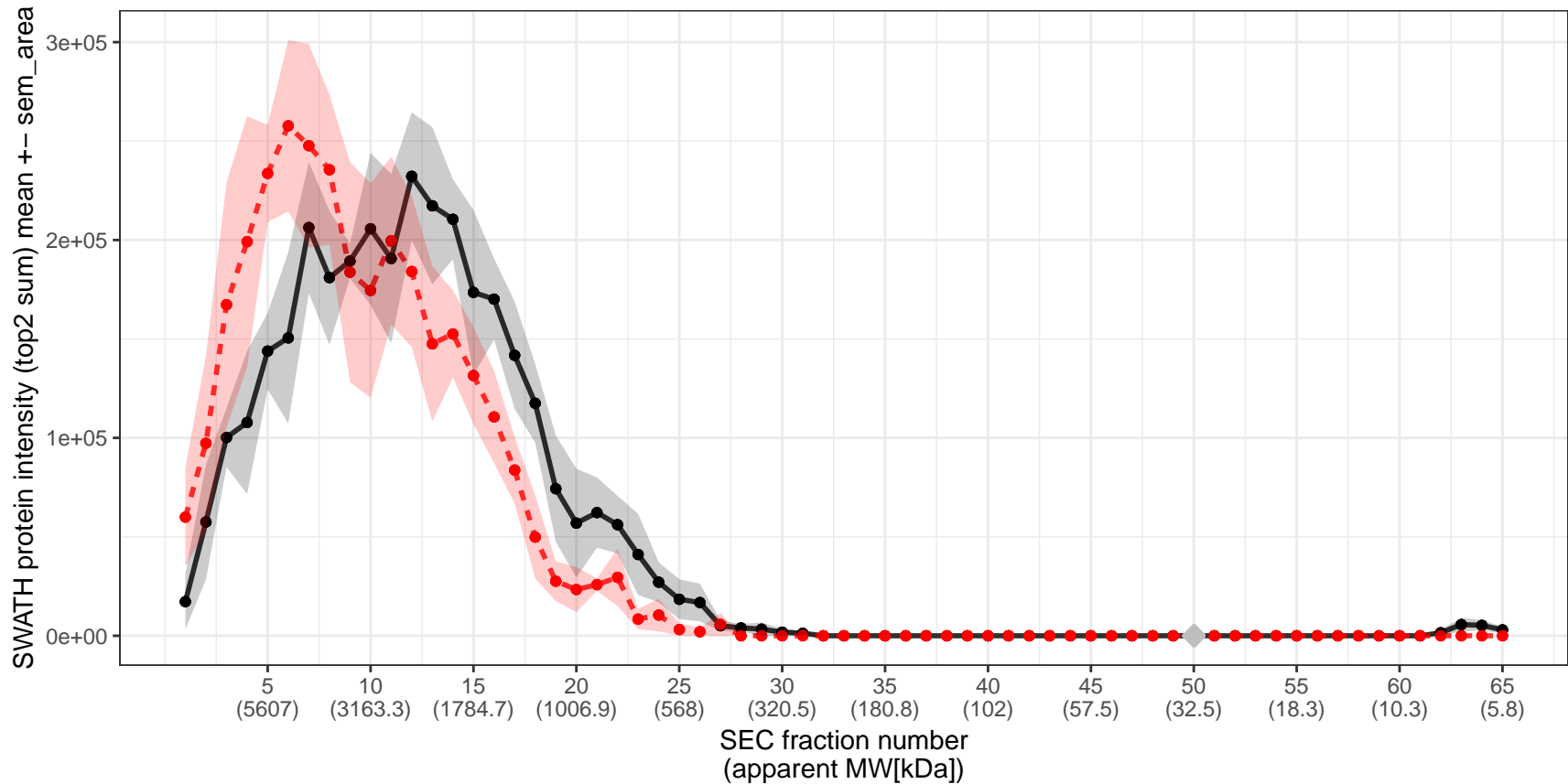

Supplement: Data S1. SEC-SWATH-MS Protein Chromatograms, Related to Figure 1 [file mmc6.zip › SECchrom_A5PLL7_TM189_HUMAN_TMEM189_KUA.pdf]

A5PLN9 | TPC13\_HUMAN | TRAPPC13 C5orf44

Monomer MW [kDa]: 46.524 Monomer expected elution fraction: 47

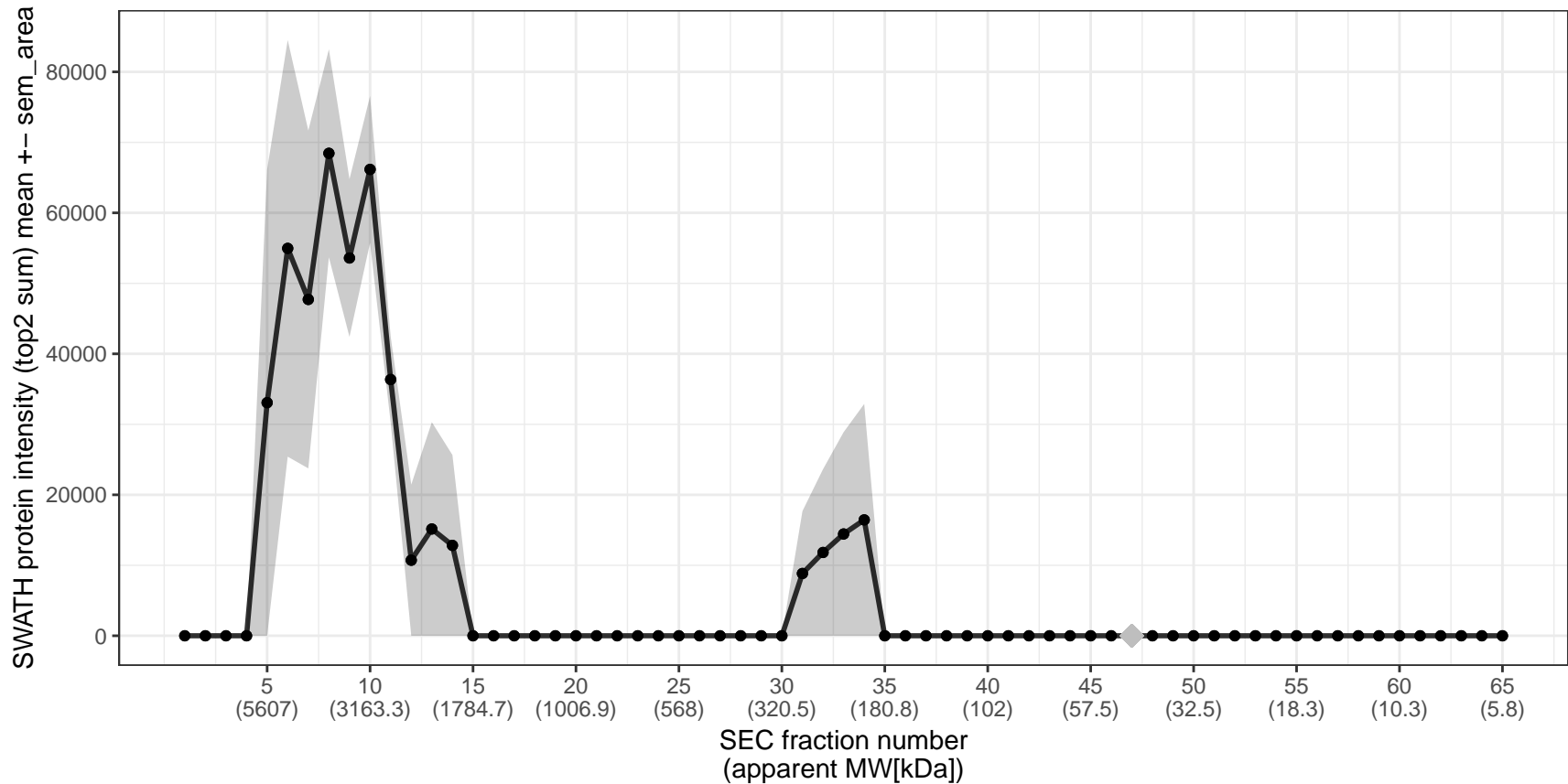

Supplement: Data S1. SEC-SWATH-MS Protein Chromatograms, Related to Figure 1 [file mmc6.zip › SECchrom_A5PLN9_TPC13_HUMAN_TRAPPC13_C5orf44.pdf]

A6NDU8 | CE051\_HUMAN | C5orf51

Monomer MW [kDa]: 33.62 Monomer expected elution fraction: 50

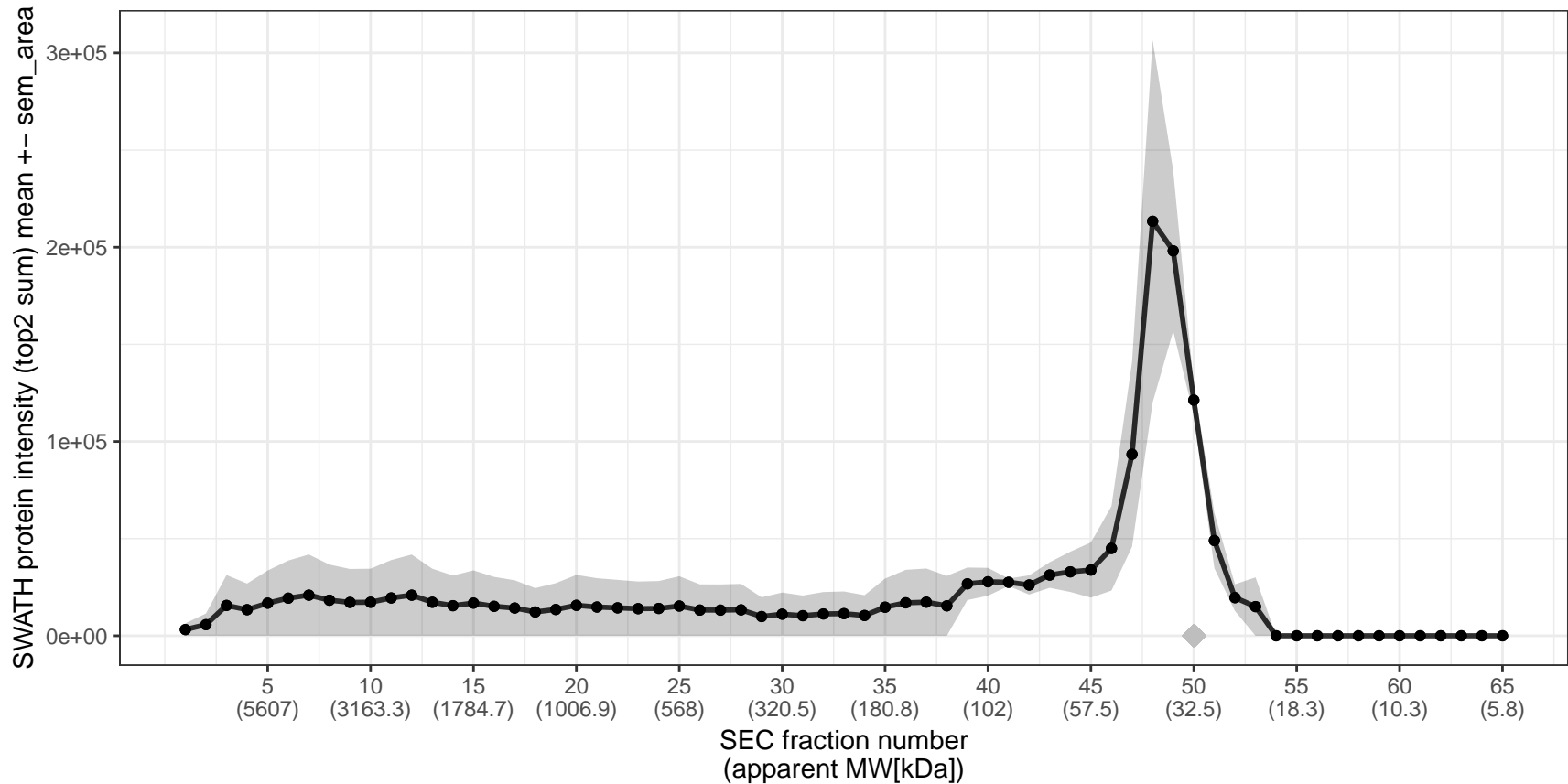

Supplement: Data S1. SEC-SWATH-MS Protein Chromatograms, Related to Figure 1 [file mmc6.zip › SECchrom_A6NDU8_CE051_HUMAN_C5orf51.pdf]

A6NED2 | RCCD1\_HUMAN | RCCD1

Monomer MW [kDa]: 40.079 Monomer expected elution fraction: 48

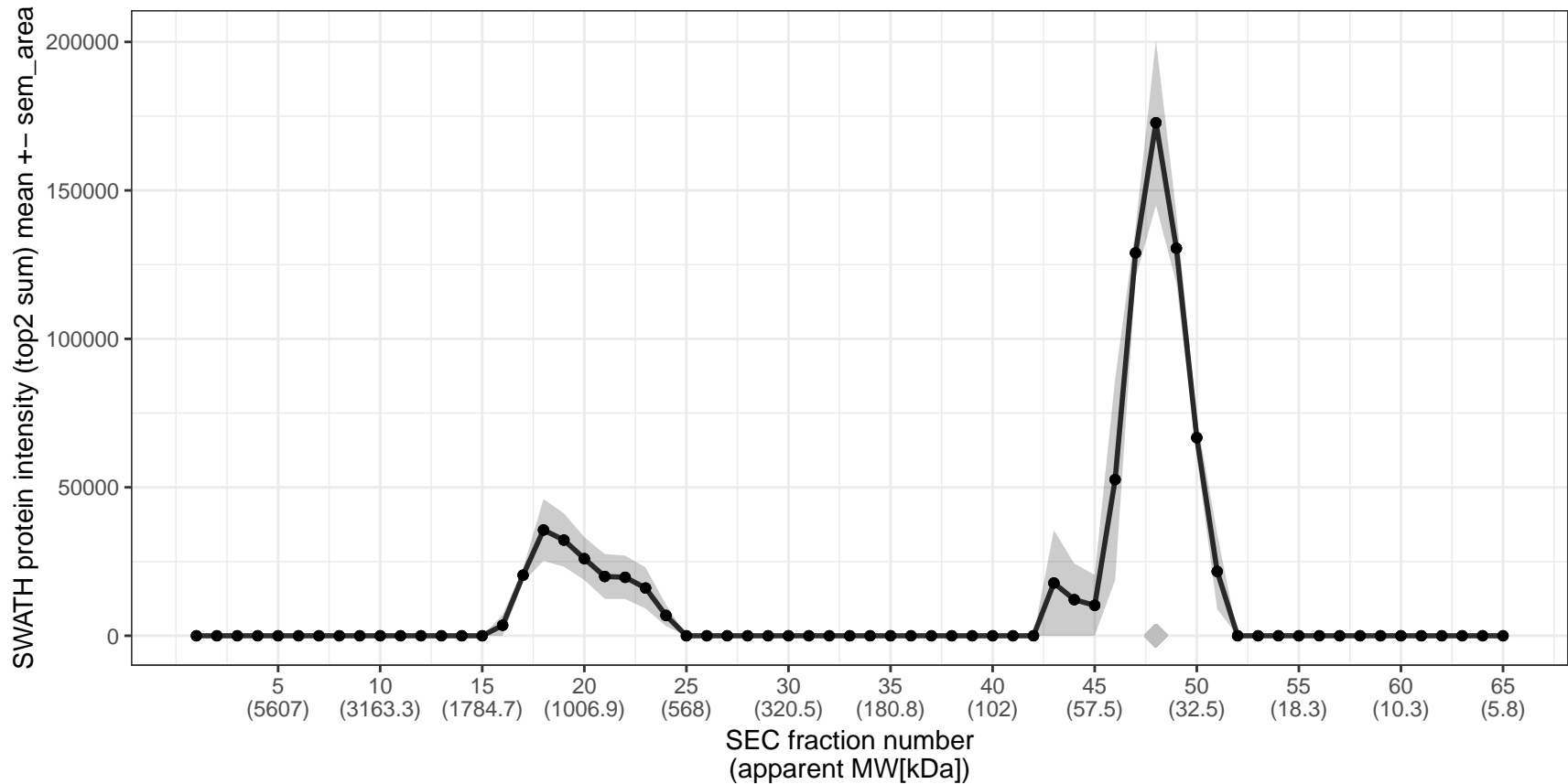

Supplement: Data S1. SEC-SWATH-MS Protein Chromatograms, Related to Figure 1 [file mmc6.zip › SECchrom_A6NED2_RCCD1_HUMAN_RCCD1.pdf]

A6NKD9 | CC85C\_HUMAN | CCDC85C

Monomer MW [kDa]: 45.21 Monomer expected elution fraction: 47

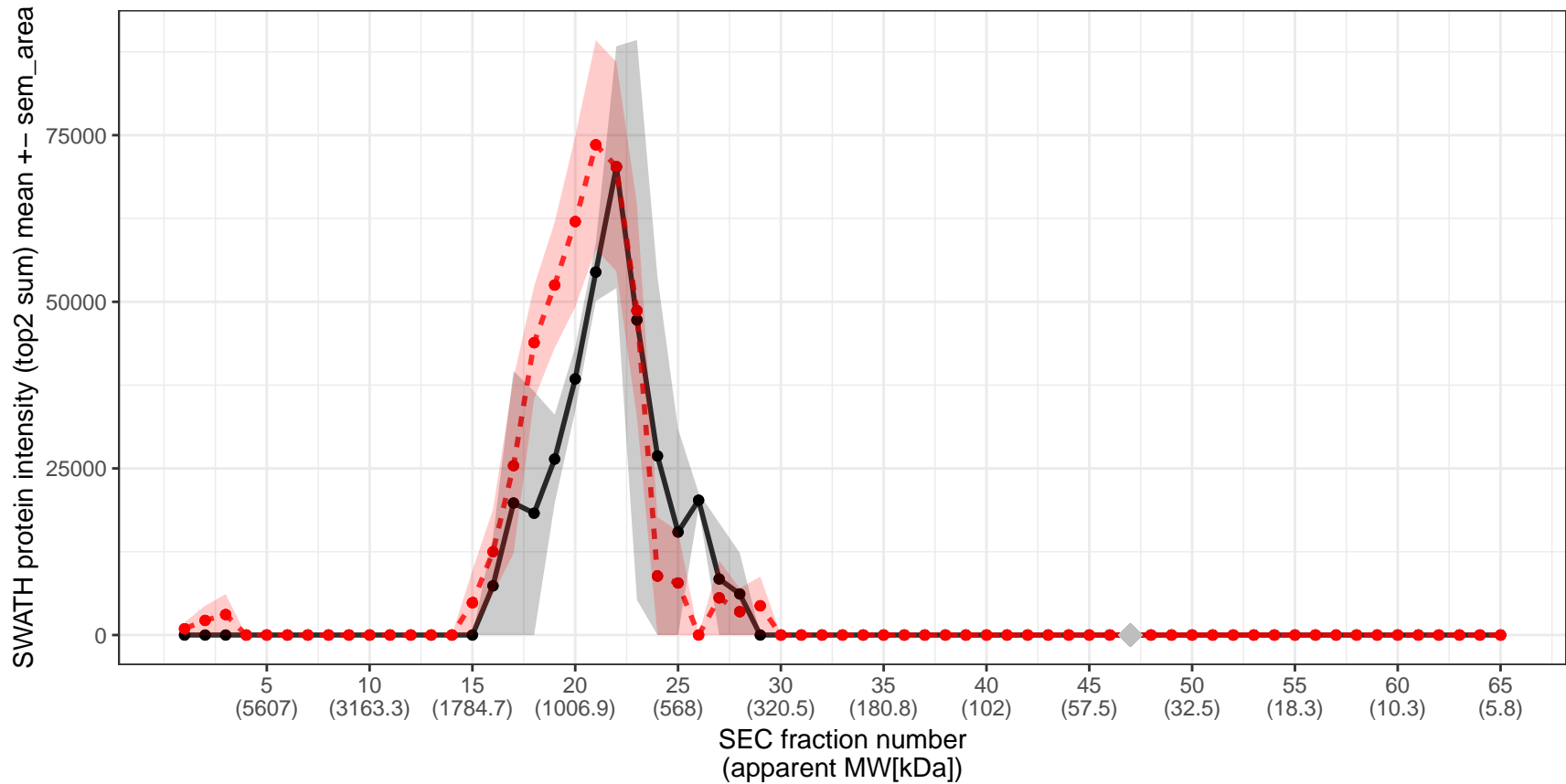

Supplement: Data S1. SEC-SWATH-MS Protein Chromatograms, Related to Figure 1 [file mmc6.zip › SECchrom_A6NKD9_CC85C_HUMAN_CCDC85C.pdf]

C9JLW8 | MCRI1\_HUMAN | MCRIP1 FAM195B GRAN2

Monomer MW [kDa]: 10.92 Monomer expected elution fraction: 60

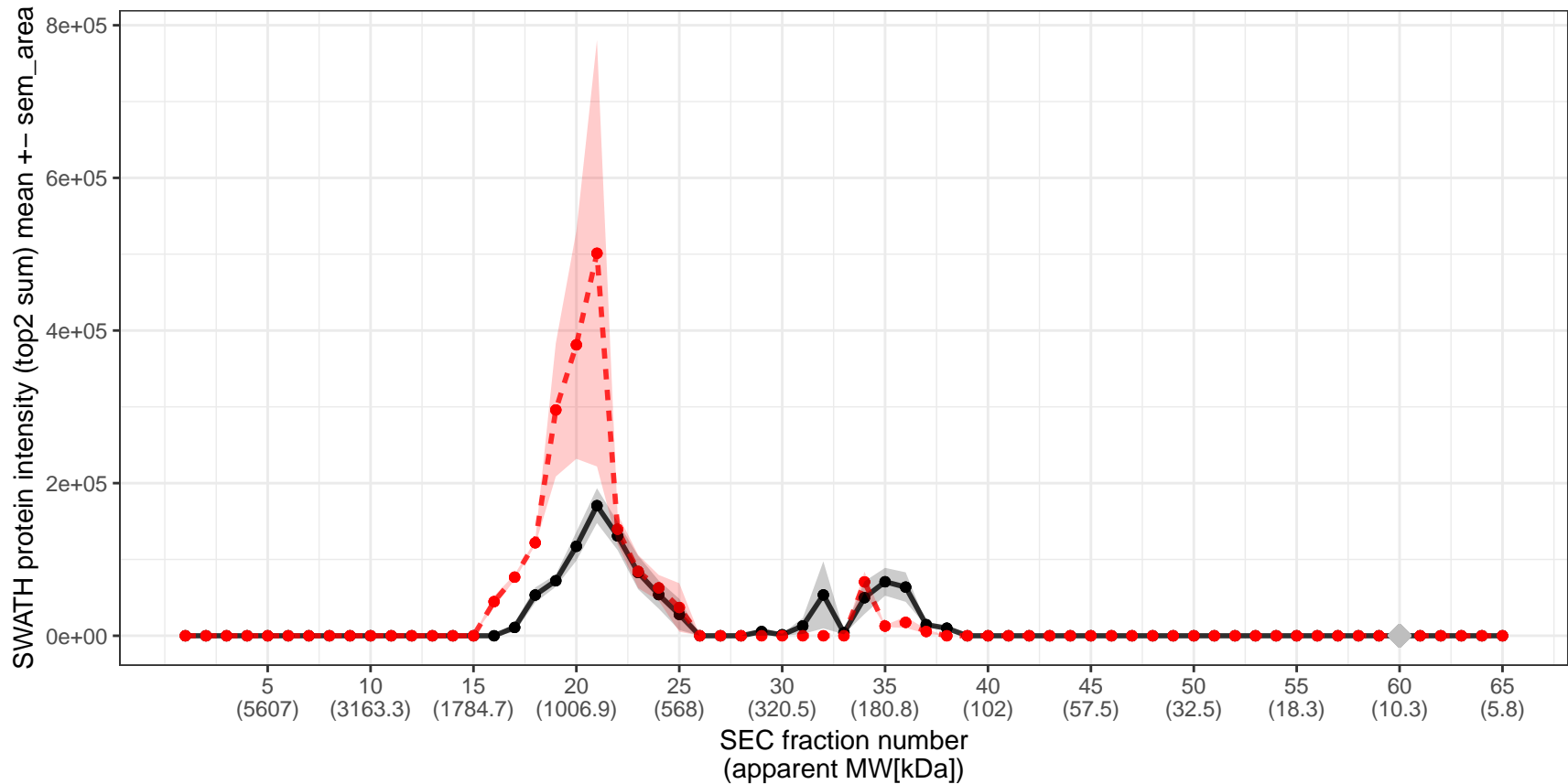

Supplement: Data S1. SEC-SWATH-MS Protein Chromatograms, Related to Figure 1 [file mmc6.zip › SECchrom_C9JLW8_MCRI1_HUMAN_MCRIP1_FAM195B_GRAN2.pdf]

cRAP\_ALBU\_BOVIN | NA | NA

Monomer MW [kDa]: NA Monomer expected elution fraction: NA

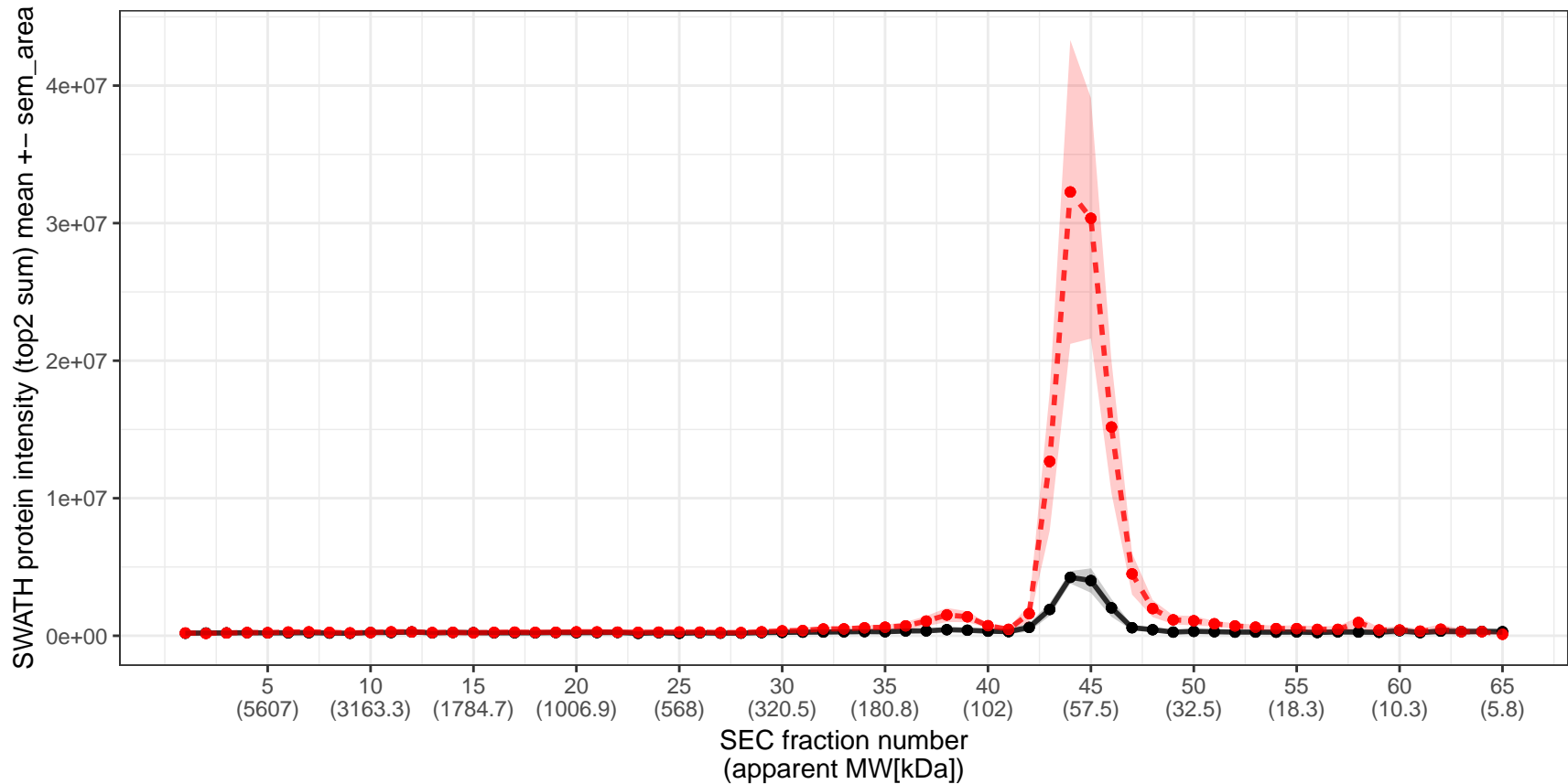

Supplement: Data S1. SEC-SWATH-MS Protein Chromatograms, Related to Figure 1 [file mmc6.zip › SECchrom_cRAP_ALBU_BOVIN_NA_NA.pdf]

cRAP\_MYG\_HORSE | NA | NA

Monomer MW [kDa]: NA Monomer expected elution fraction: NA

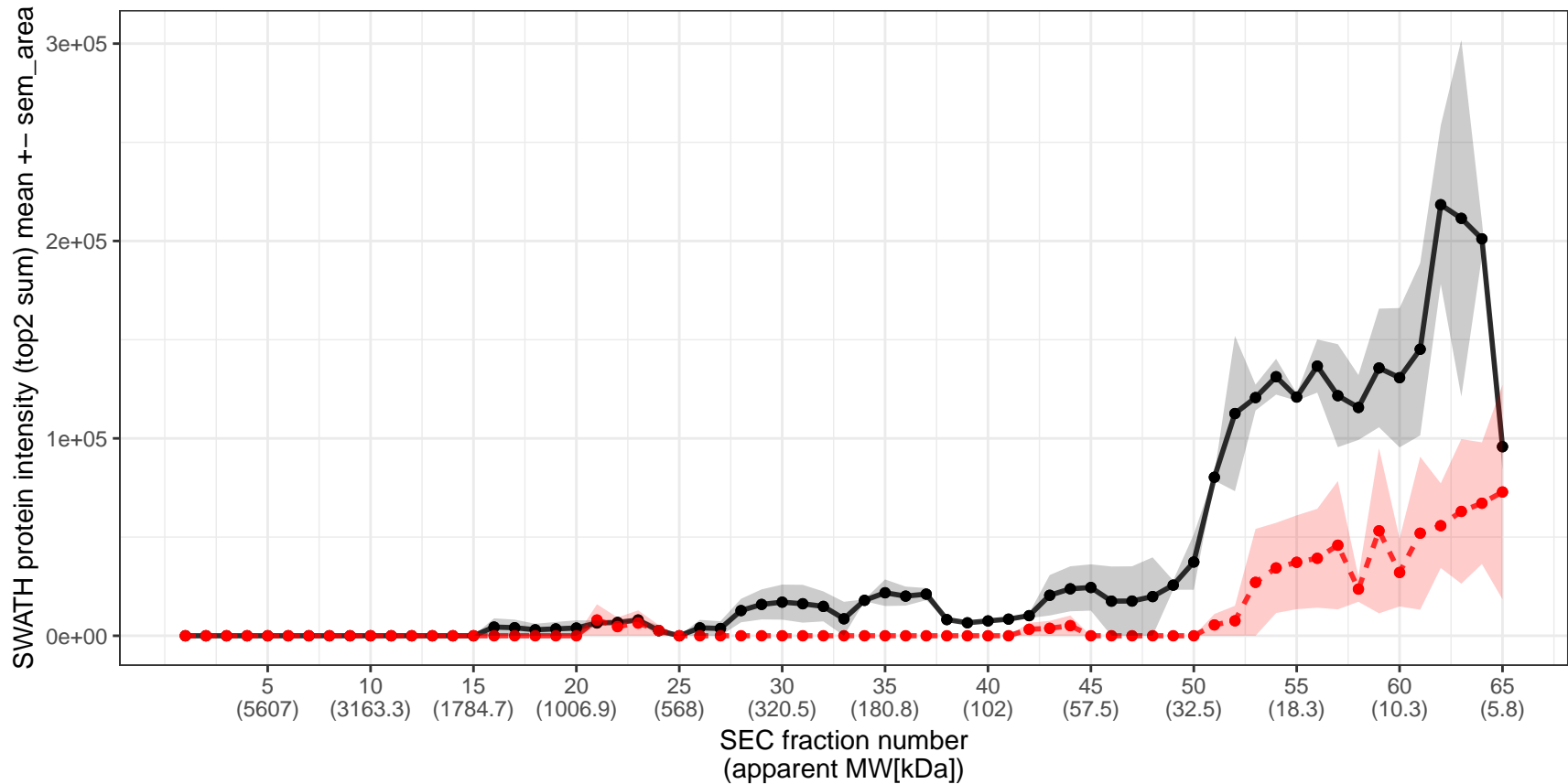

Supplement: Data S1. SEC-SWATH-MS Protein Chromatograms, Related to Figure 1 [file mmc6.zip › SECchrom_cRAP_MYG_HORSE_NA_NA.pdf]

cRAP\_OVAL\_CHICK | NA | NA

Monomer MW [kDa]: NA Monomer expected elution fraction: NA

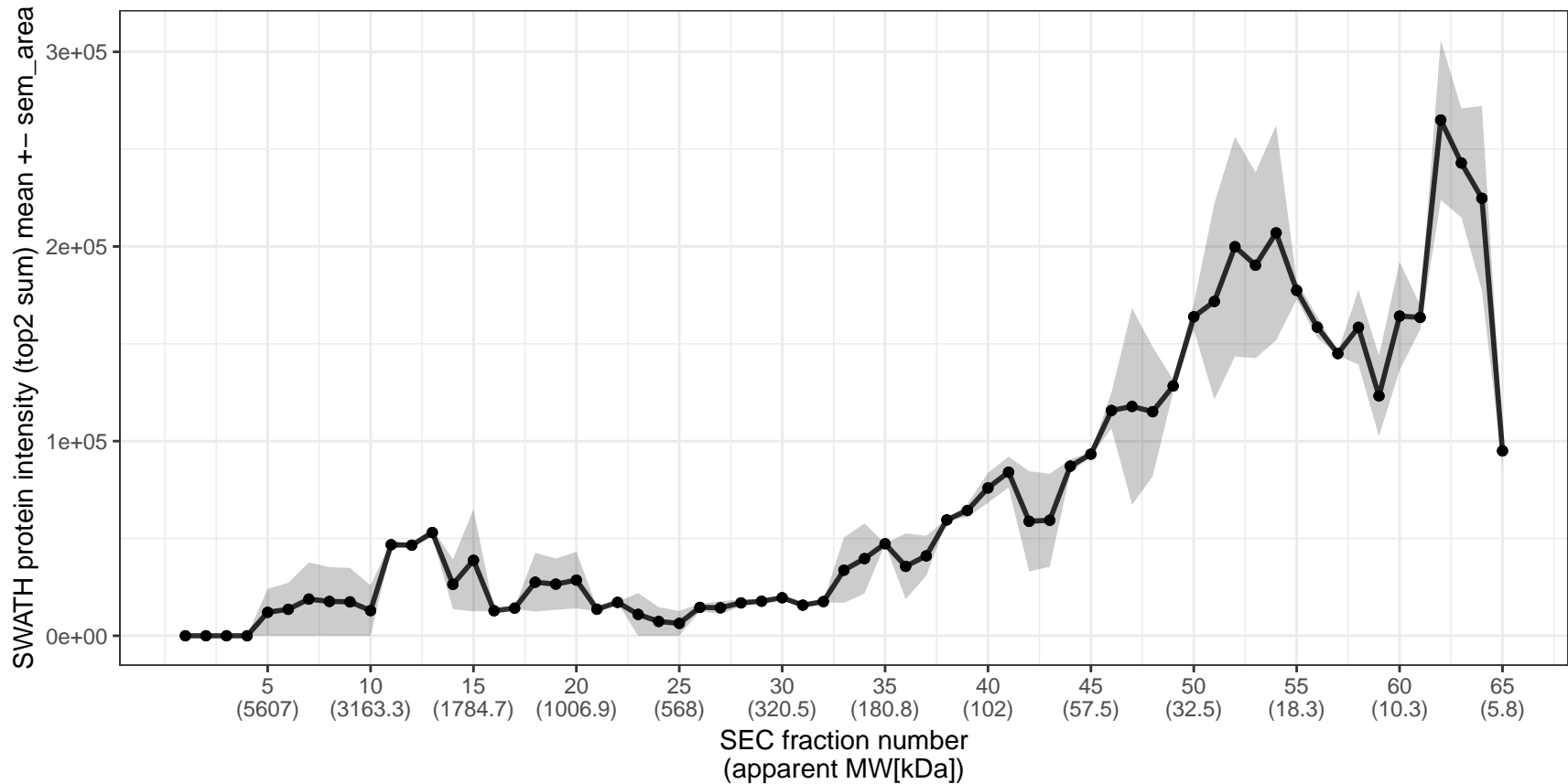

Supplement: Data S1. SEC-SWATH-MS Protein Chromatograms, Related to Figure 1 [file mmc6.zip › SECchrom_cRAP_OVAL_CHICK_NA_NA.pdf]

iRT\_protein | NA | NA

Monomer MW [kDa]: NA Monomer expected elution fraction: NA

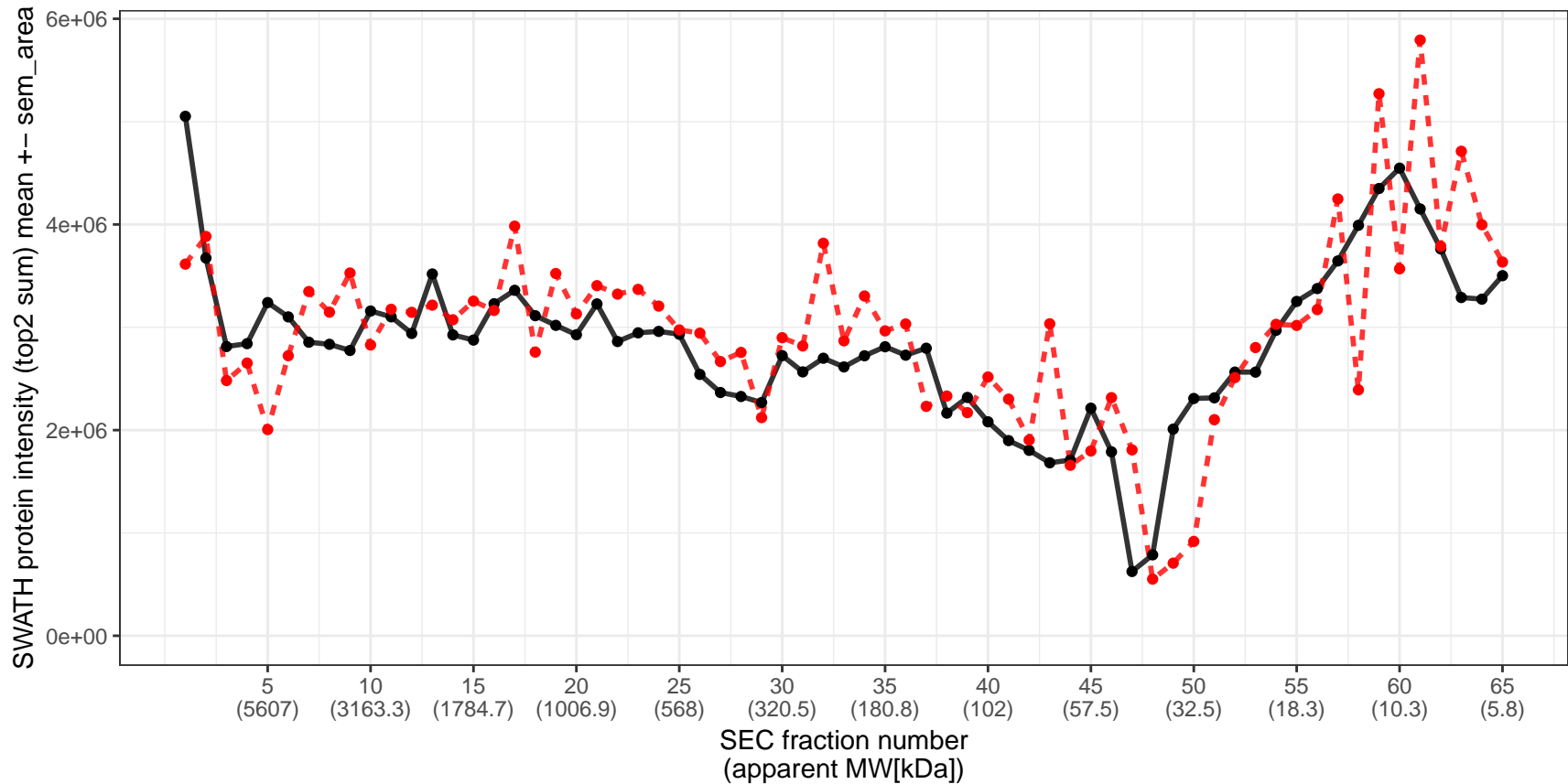

Supplement: Data S1. SEC-SWATH-MS Protein Chromatograms, Related to Figure 1 [file mmc6.zip › SECchrom_iRT_protein_NA_NA.pdf]

O00116 | ADAS\_HUMAN | AGPS AAG5

Monomer MW [kDa]: 72.912 Monomer expected elution fraction: 43

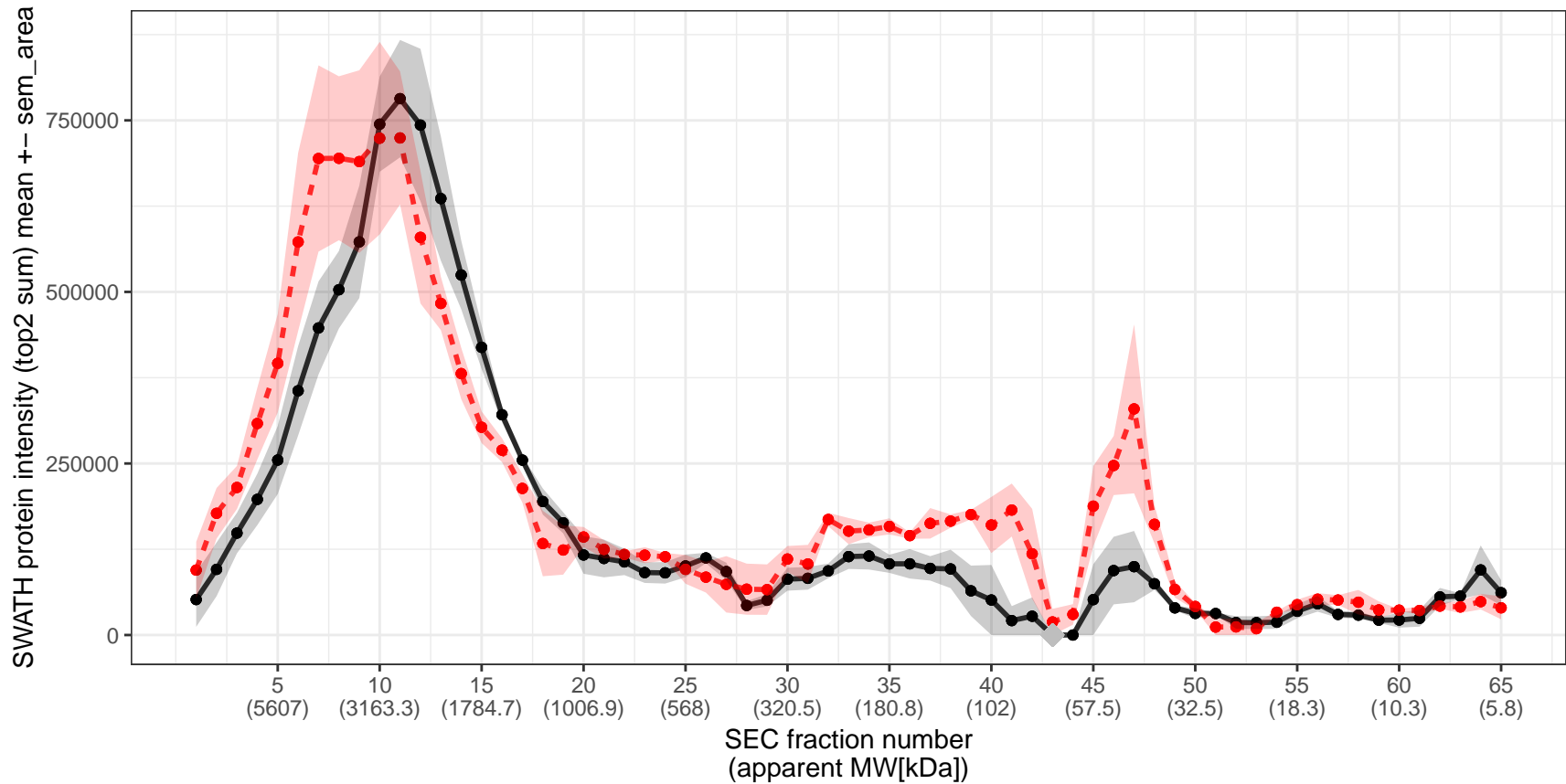

Supplement: Data S1. SEC-SWATH-MS Protein Chromatograms, Related to Figure 1 [file mmc6.zip › SECchrom_O00116_ADAS_HUMAN_AGPS_AAG5.pdf]

O00139 | KIF2A\_HUMAN | KIF2A KIF2 KNS2

Monomer MW [kDa]: 79.955 Monomer expected elution fraction: 42

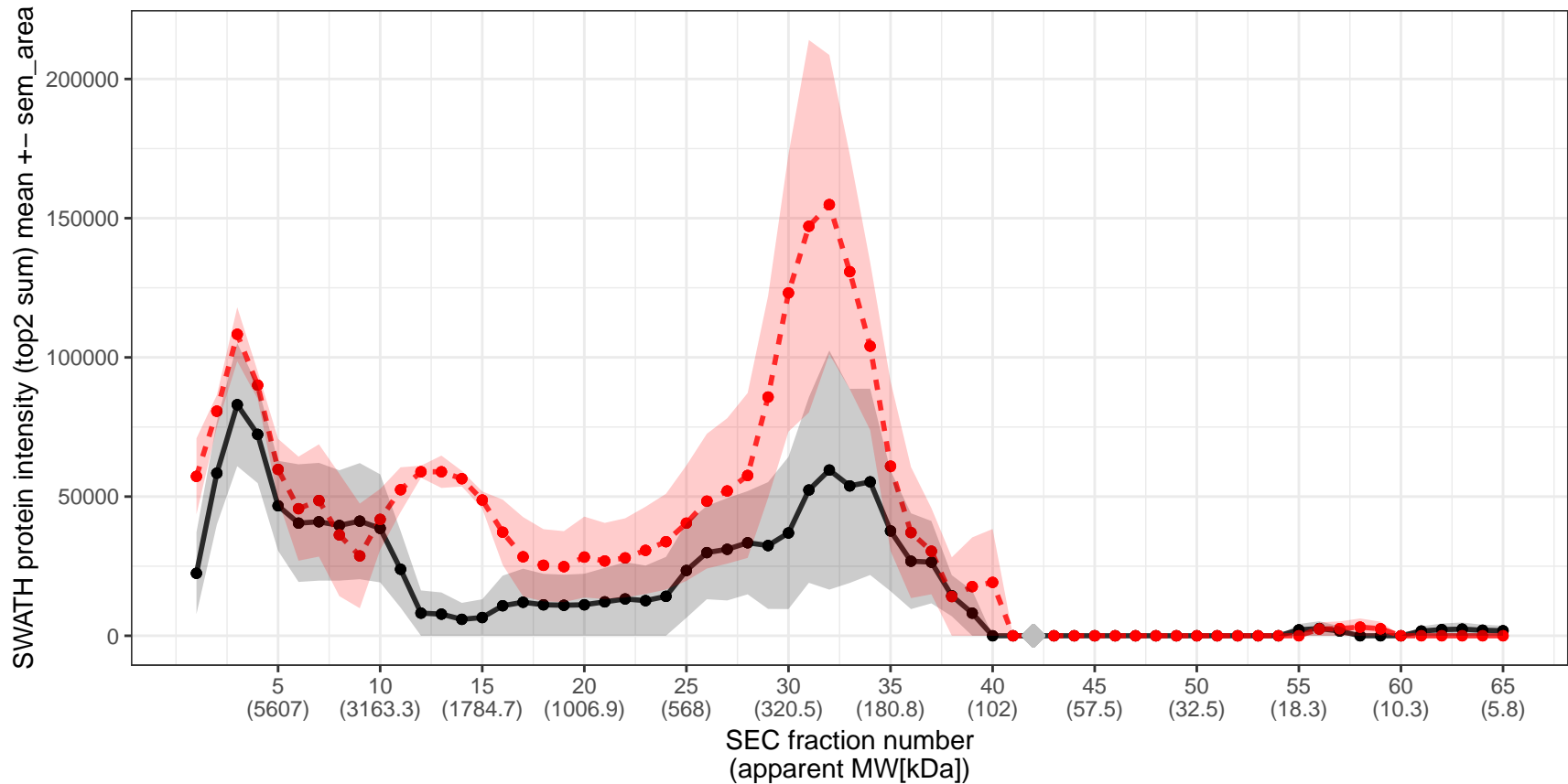

Supplement: Data S1. SEC-SWATH-MS Protein Chromatograms, Related to Figure 1 [file mmc6.zip › SECchrom_O00139_KIF2A_HUMAN_KIF2A_KIF2_KNS2.pdf]

O00151 | PDLI1\_HUMAN | PDLIM1 CLIM1 CLP36

Monomer MW [kDa]: 36.072 Monomer expected elution fraction: 49

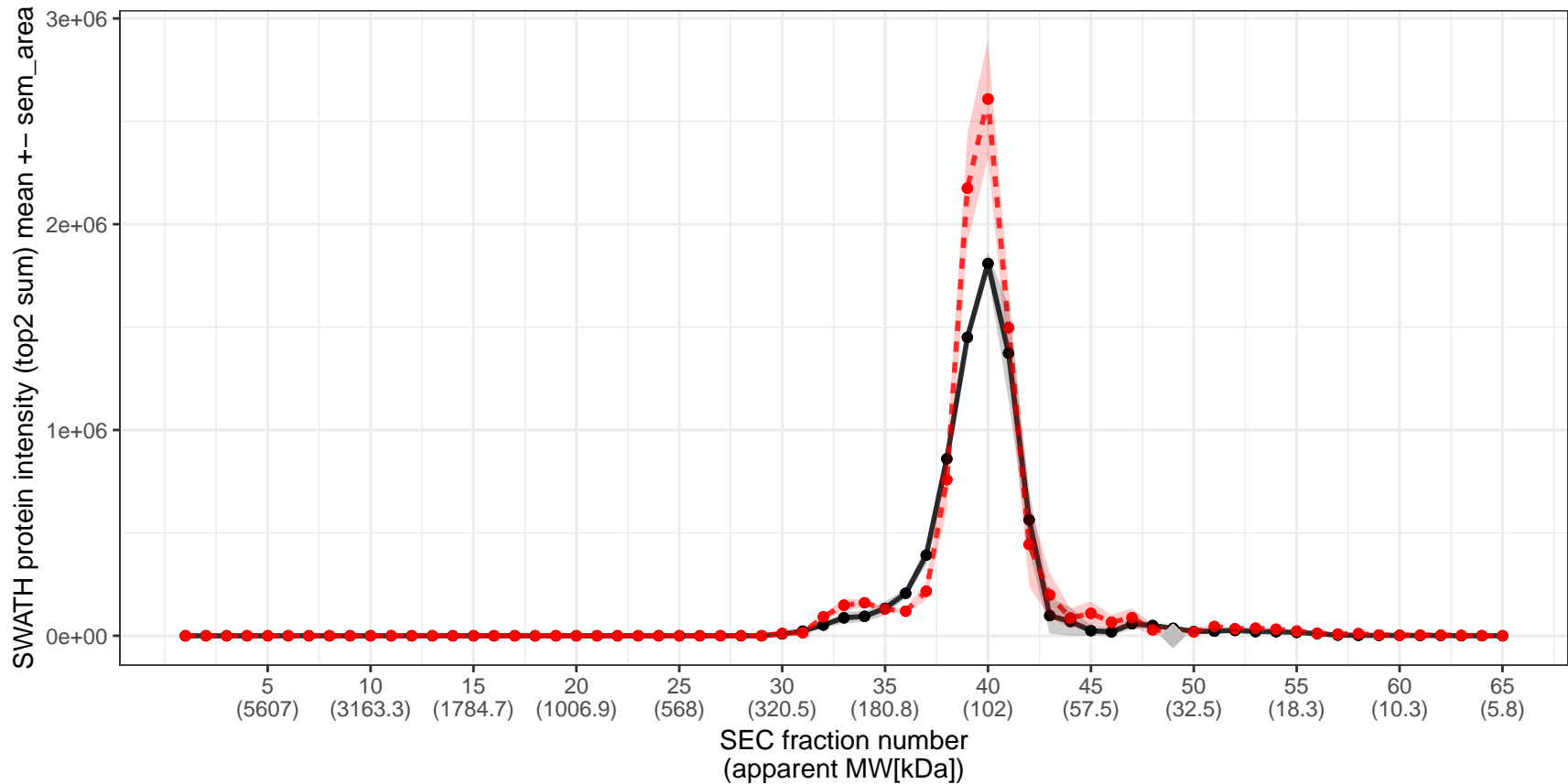

Supplement: Data S1. SEC-SWATH-MS Protein Chromatograms, Related to Figure 1 [file mmc6.zip › SECchrom_O00151_PDLI1_HUMAN_PDLIM1_CLIM1_CLP36.pdf]

O00154 | BACH\_HUMAN | ACOT7 BACH

Monomer MW [kDa]: 41.796 Monomer expected elution fraction: 48

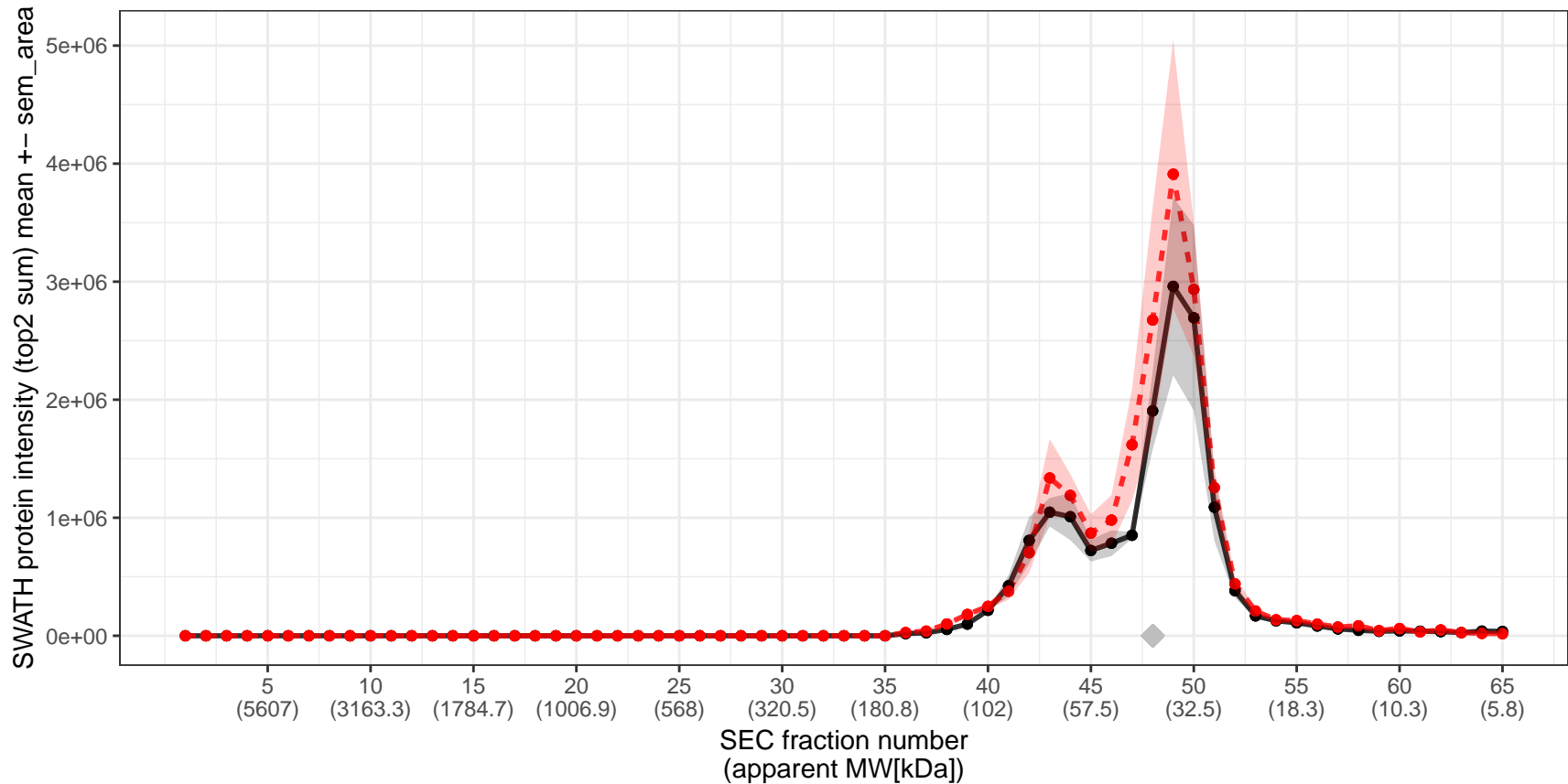

Supplement: Data S1. SEC-SWATH-MS Protein Chromatograms, Related to Figure 1 [file mmc6.zip › SECchrom_O00154_BACH_HUMAN_ACOT7_BACH.pdf]

O00159 | MYO1C\_HUMAN | MYO1C

Monomer MW [kDa]: 121.682 Monomer expected elution fraction: 38

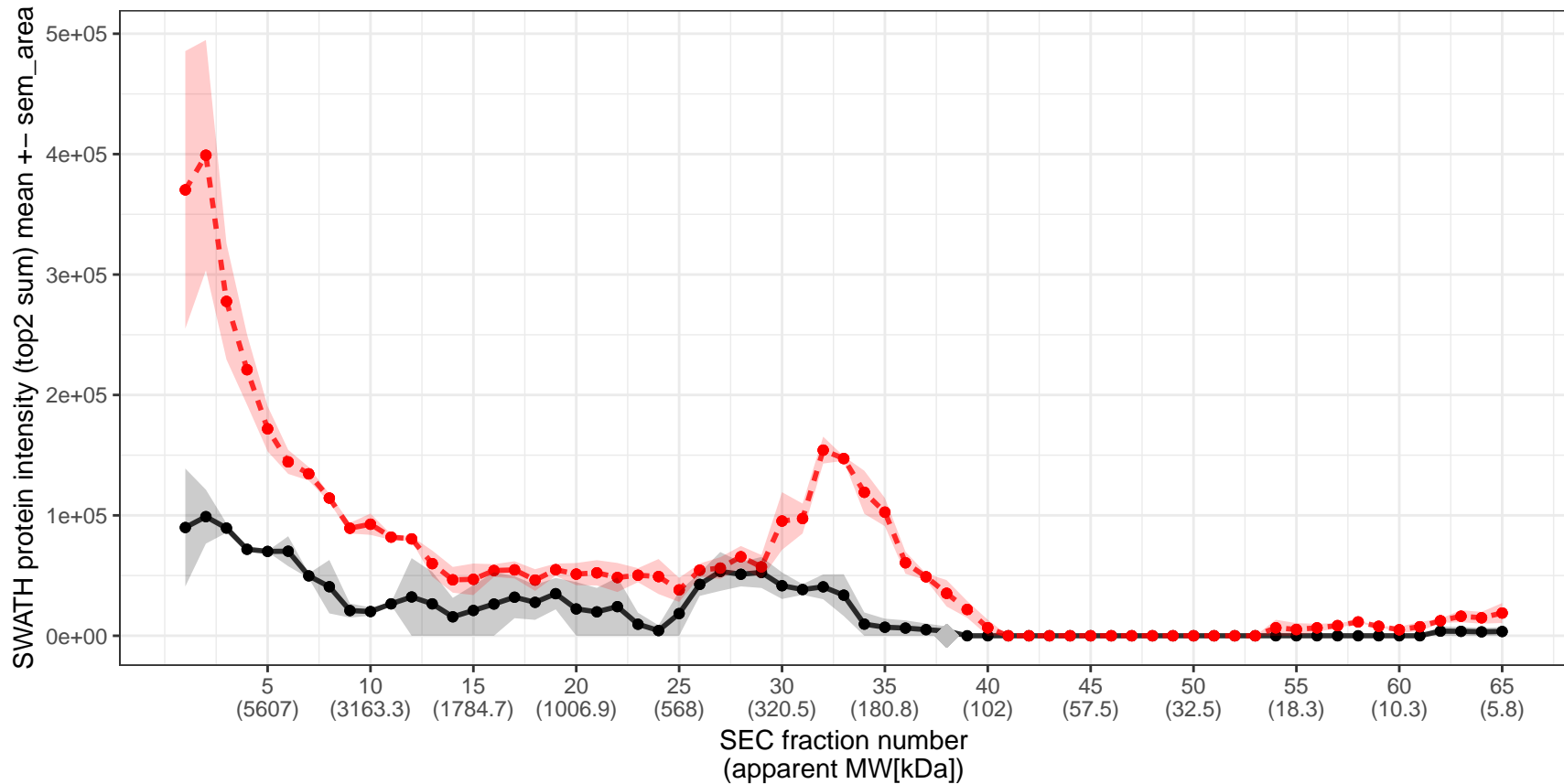

Supplement: Data S1. SEC-SWATH-MS Protein Chromatograms, Related to Figure 1 [file mmc6.zip › SECchrom_O00159_MYO1C_HUMAN_MYO1C.pdf]

O00165 | HAX1\_HUMAN | HAX1 HS1BP1

Monomer MW [kDa]: 31.621 Monomer expected elution fraction: 50

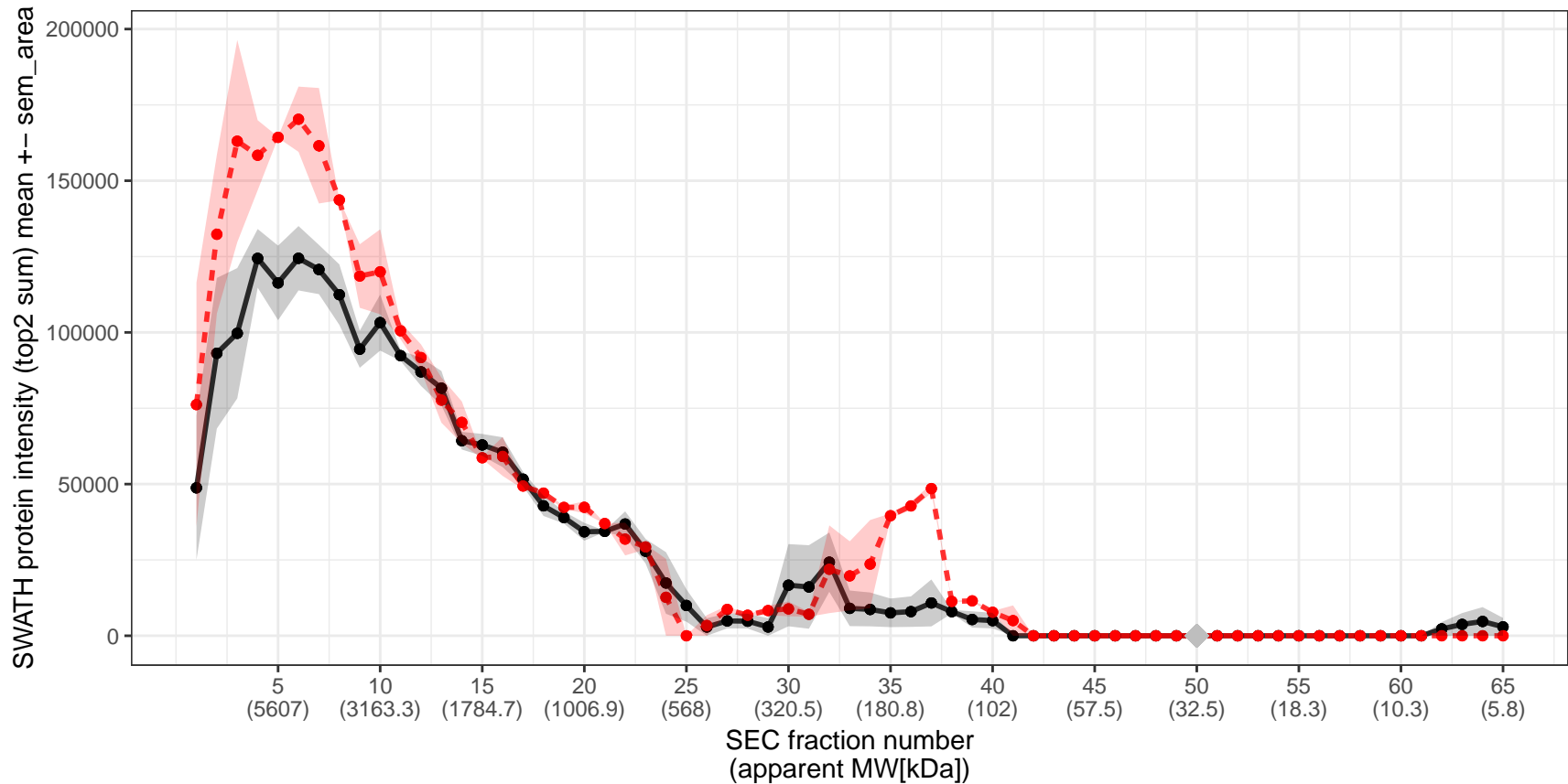

Supplement: Data S1. SEC-SWATH-MS Protein Chromatograms, Related to Figure 1 [file mmc6.zip › SECchrom_O00165_HAX1_HUMAN_HAX1_HS1BP1.pdf]

O00178 | GTPB1\_HUMAN | GTPBP1

Monomer MW [kDa]: 72.454 Monomer expected elution fraction: 43

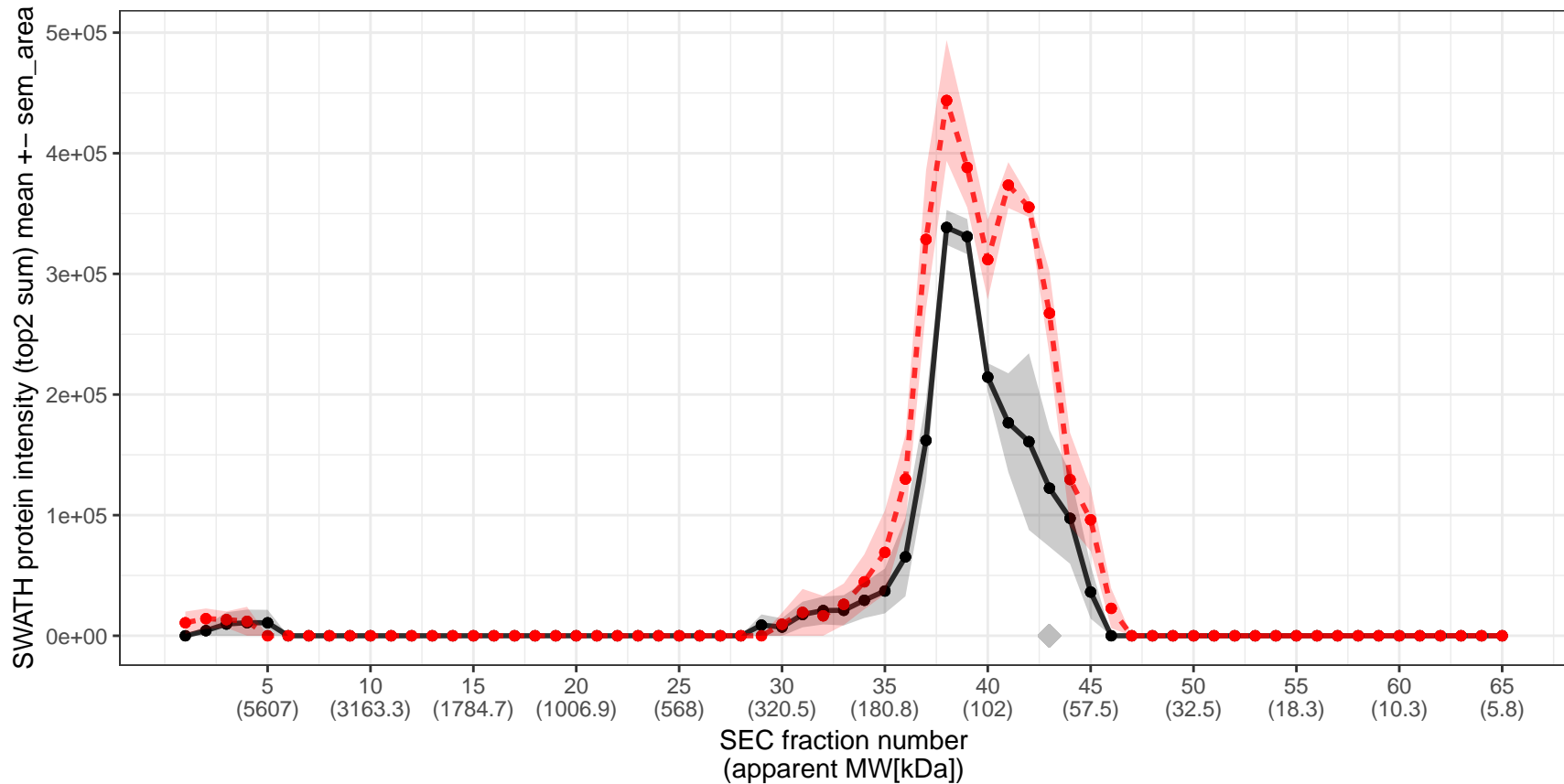

Supplement: Data S1. SEC-SWATH-MS Protein Chromatograms, Related to Figure 1 [file mmc6.zip › SECchrom_O00178_GTPB1_HUMAN_GTPBP1.pdf]

O00186 | STXB3\_HUMAN | STXBP3

Monomer MW [kDa]: 67.764 Monomer expected elution fraction: 44

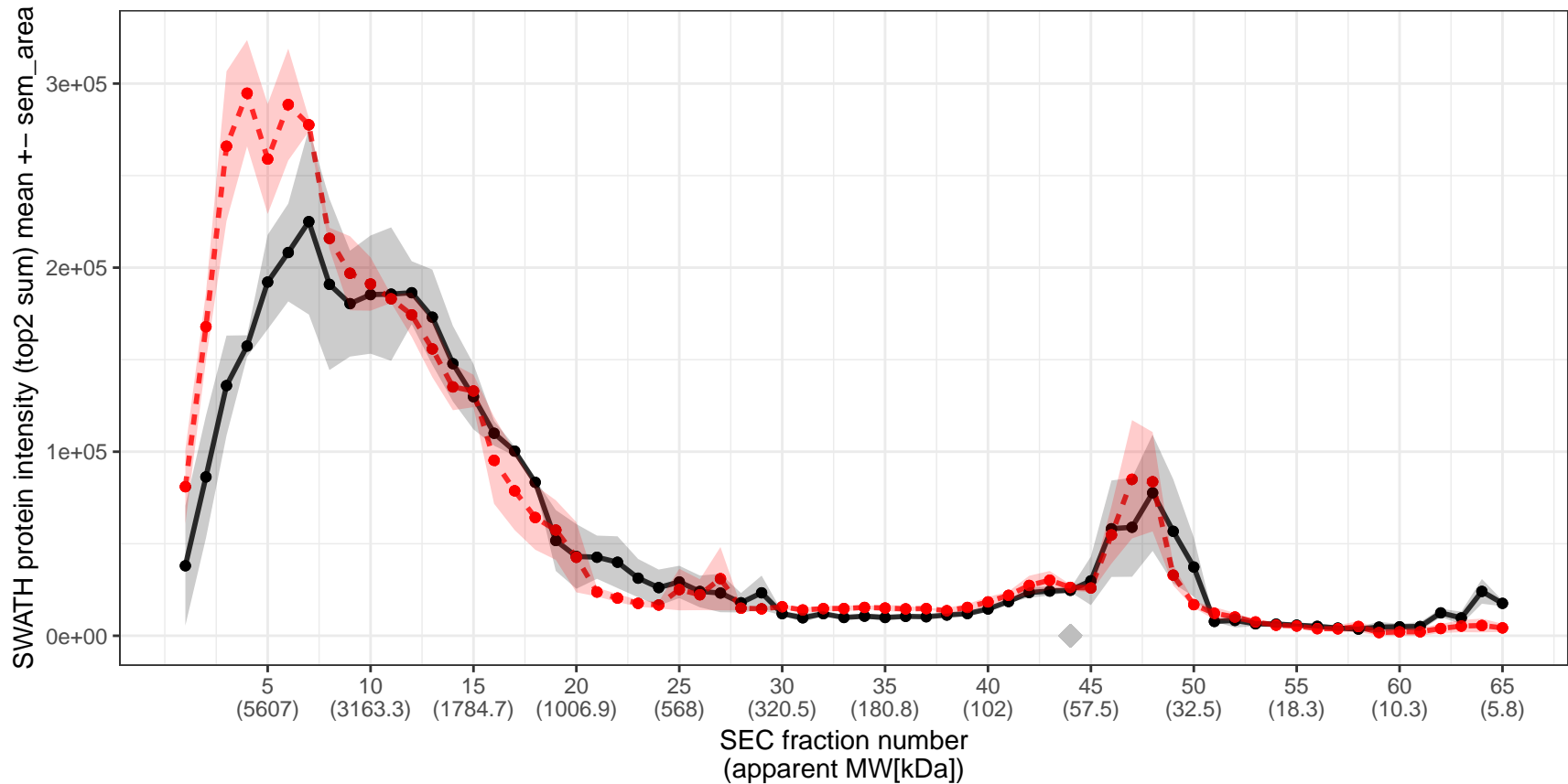

Supplement: Data S1. SEC-SWATH-MS Protein Chromatograms, Related to Figure 1 [file mmc6.zip › SECchrom_O00186_STXB3_HUMAN_STXBP3.pdf]

O00189 | AP4M1\_HUMAN | AP4M1 MUARP2

Monomer MW [kDa]: 49.977 Monomer expected elution fraction: 46

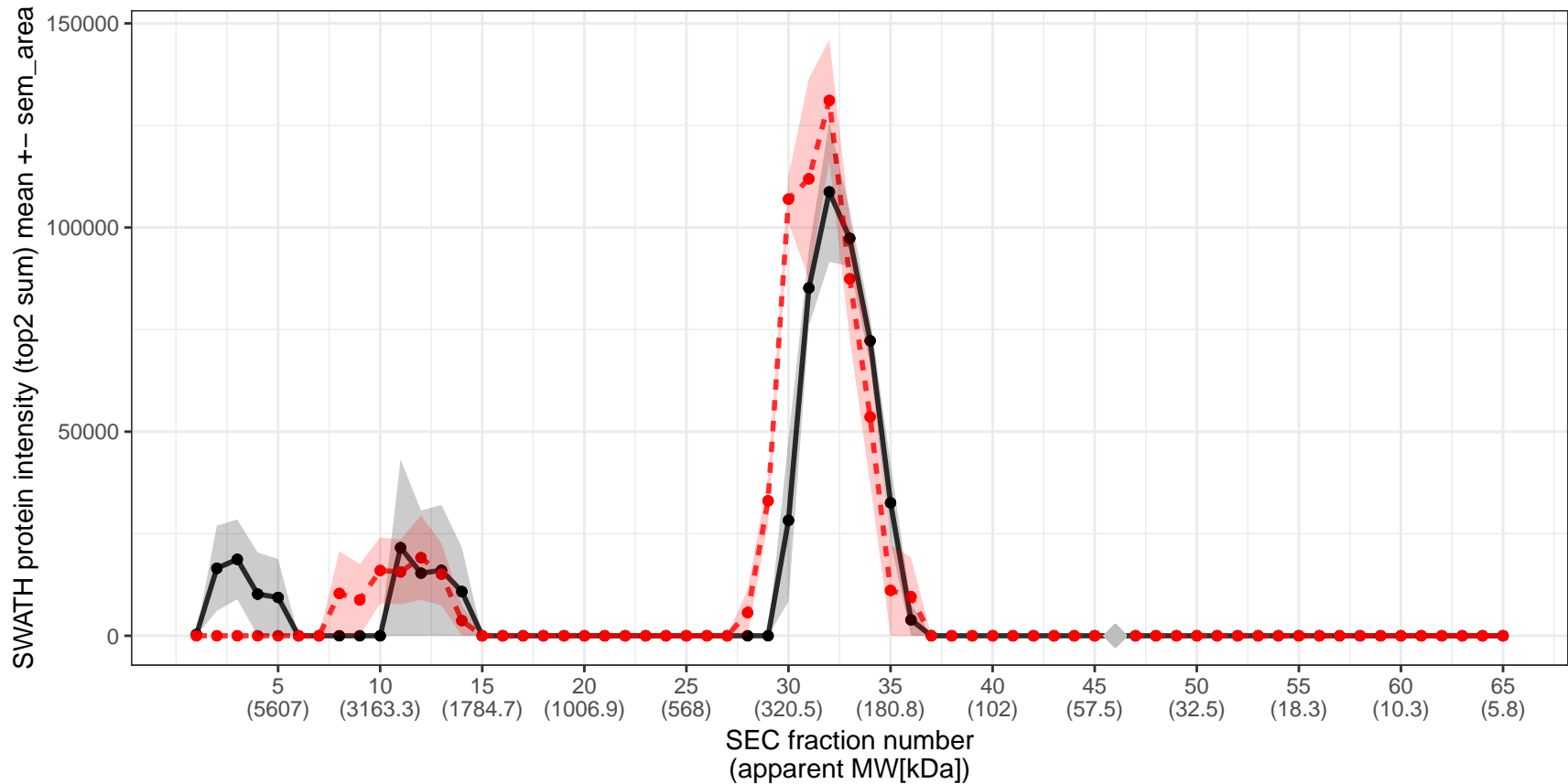

Supplement: Data S1. SEC-SWATH-MS Protein Chromatograms, Related to Figure 1 [file mmc6.zip › SECchrom_O00189_AP4M1_HUMAN_AP4M1_MUARP2.pdf]

O00193 | SMAP\_HUMAN | SMAP C11orf58

Monomer MW [kDa]: 20.333 Monomer expected elution fraction: 54

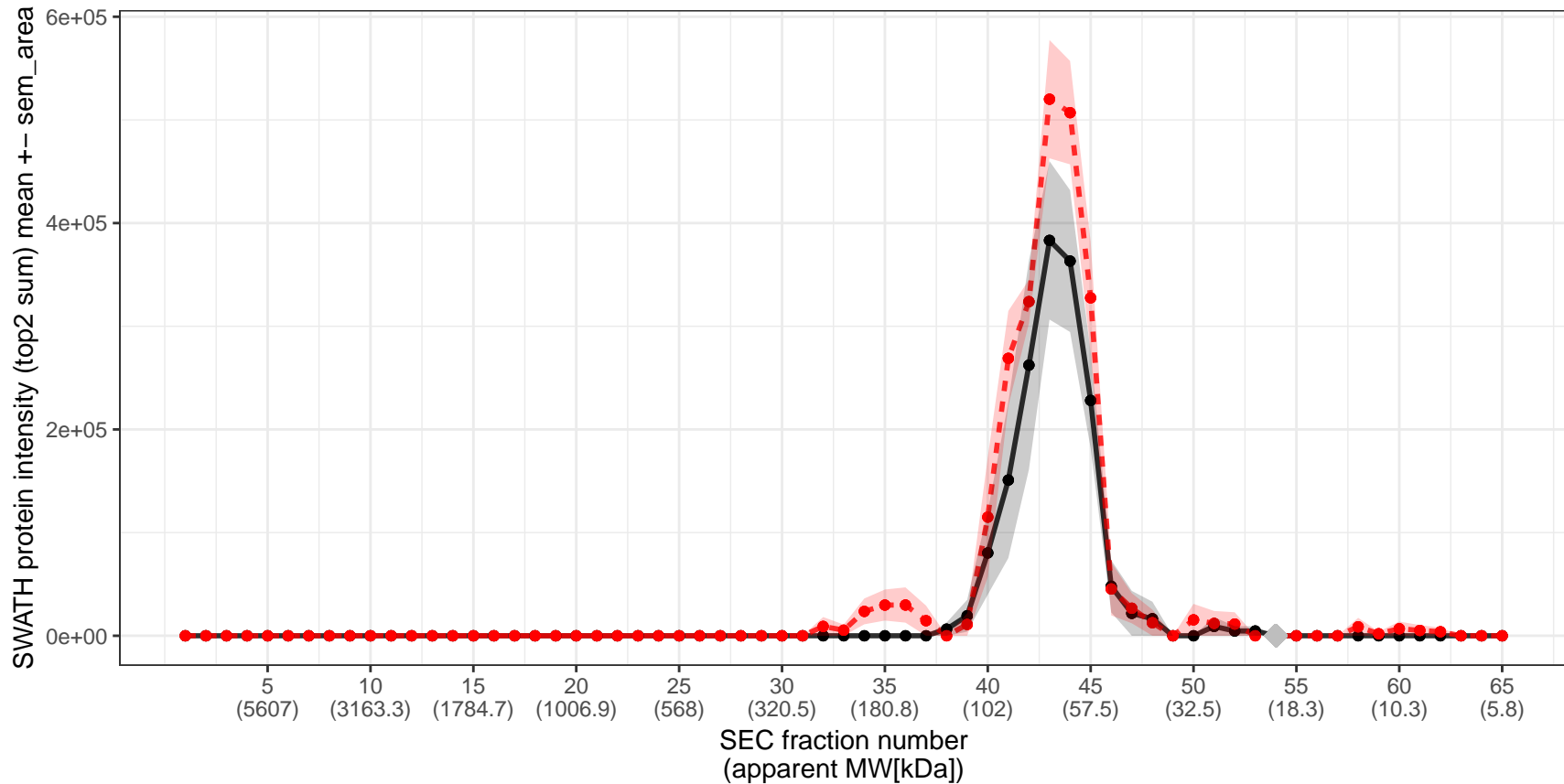

Supplement: Data S1. SEC-SWATH-MS Protein Chromatograms, Related to Figure 1 [file mmc6.zip › SECchrom_O00193_SMAP_HUMAN_SMAP_C11orf58.pdf]

O00203 | AP3B1\_HUMAN | AP3B1 ADTB3A

Monomer MW [kDa]: 121.32 Monomer expected elution fraction: 38

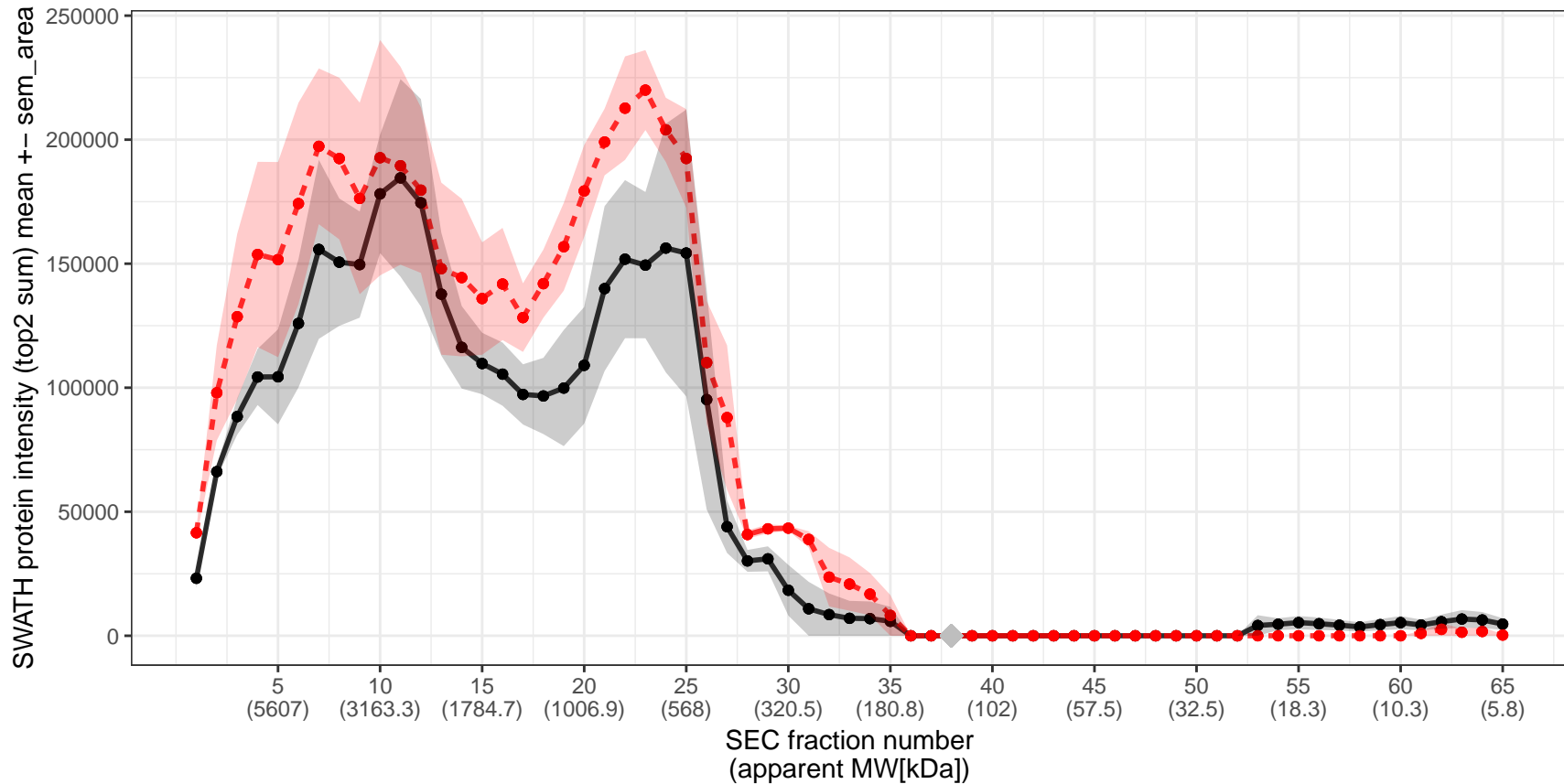

Supplement: Data S1. SEC-SWATH-MS Protein Chromatograms, Related to Figure 1 [file mmc6.zip › SECchrom_O00203_AP3B1_HUMAN_AP3B1_ADTB3A.pdf]

O00213 | APBB1\_HUMAN | APBB1 FE65 RIR

Monomer MW [kDa]: 77.244 Monomer expected elution fraction: 42

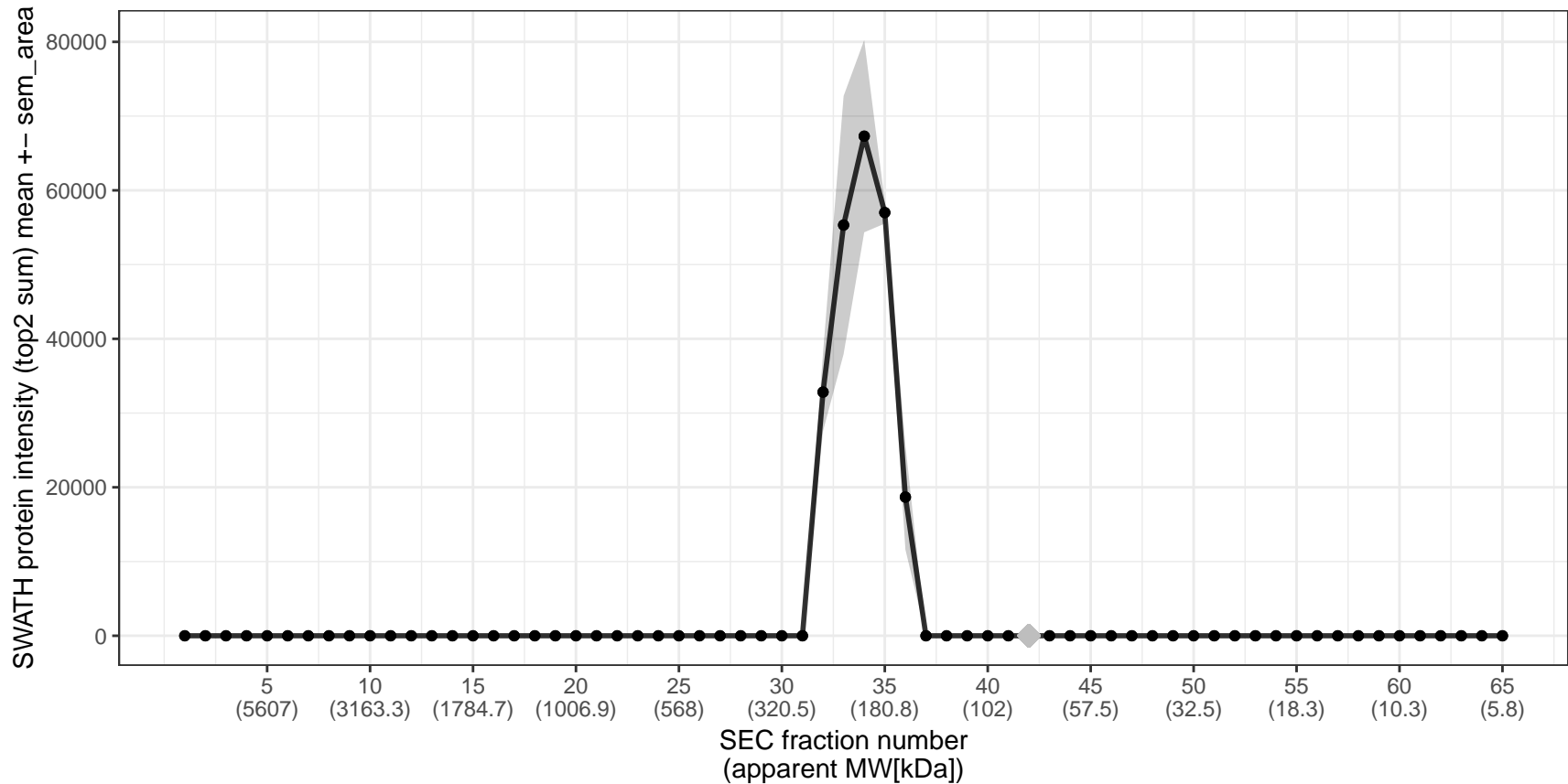

Supplement: Data S1. SEC-SWATH-MS Protein Chromatograms, Related to Figure 1 [file mmc6.zip › SECchrom_O00213_APBB1_HUMAN_APBB1_FE65_RIR.pdf]

O00217 | NDUS8\_HUMAN | NDUFS8

Monomer MW [kDa]: 23.705 Monomer expected elution fraction: 53

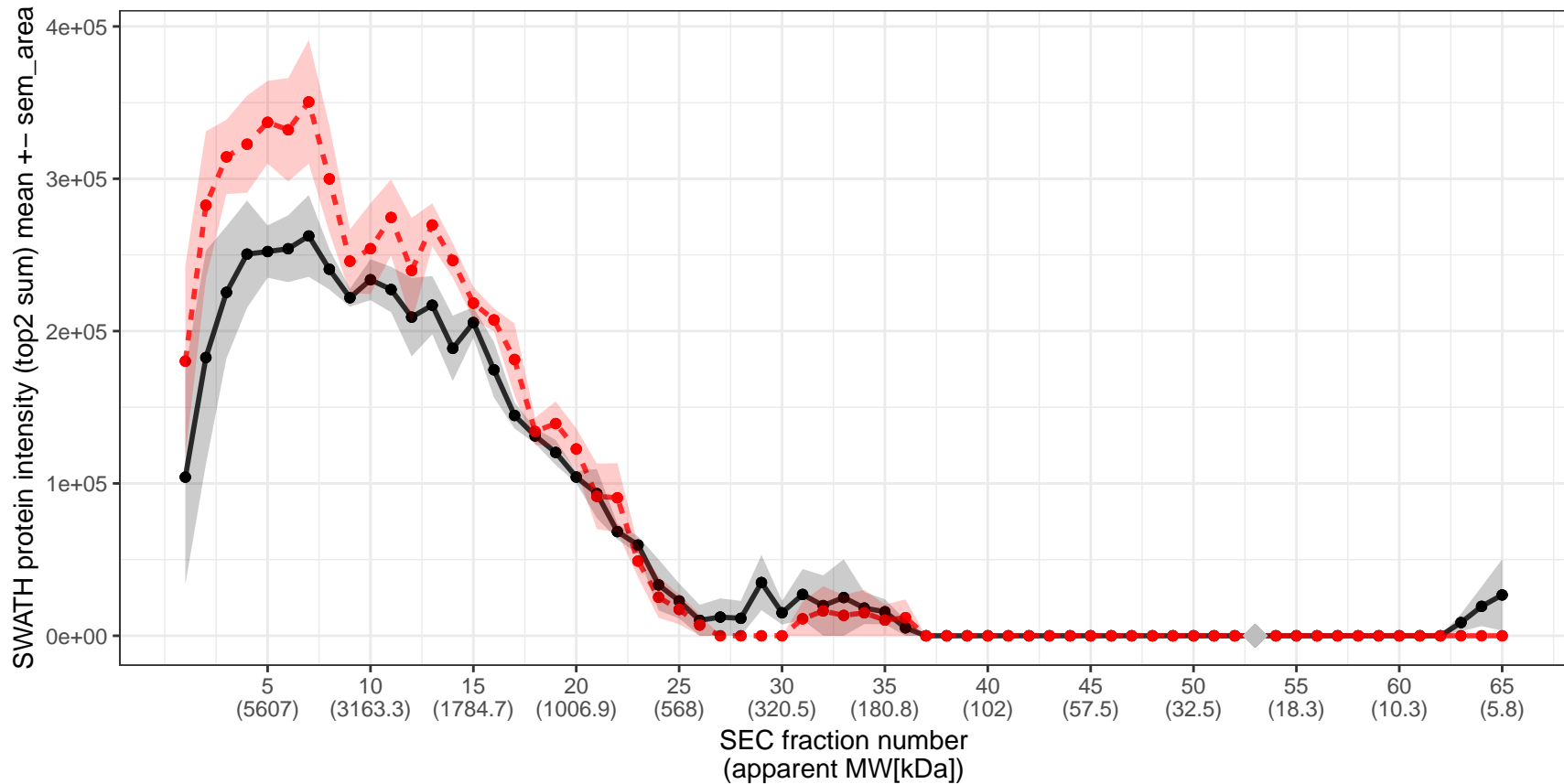

Supplement: Data S1. SEC-SWATH-MS Protein Chromatograms, Related to Figure 1 [file mmc6.zip › SECchrom_O00217_NDUS8_HUMAN_NDUFS8.pdf]

O00220 | TR10A\_HUMAN | TNFRSF10A APO2 DR4 TRAILR1  
Monomer MW [kDa]: 50.089 Monomer expected elution fraction: 46

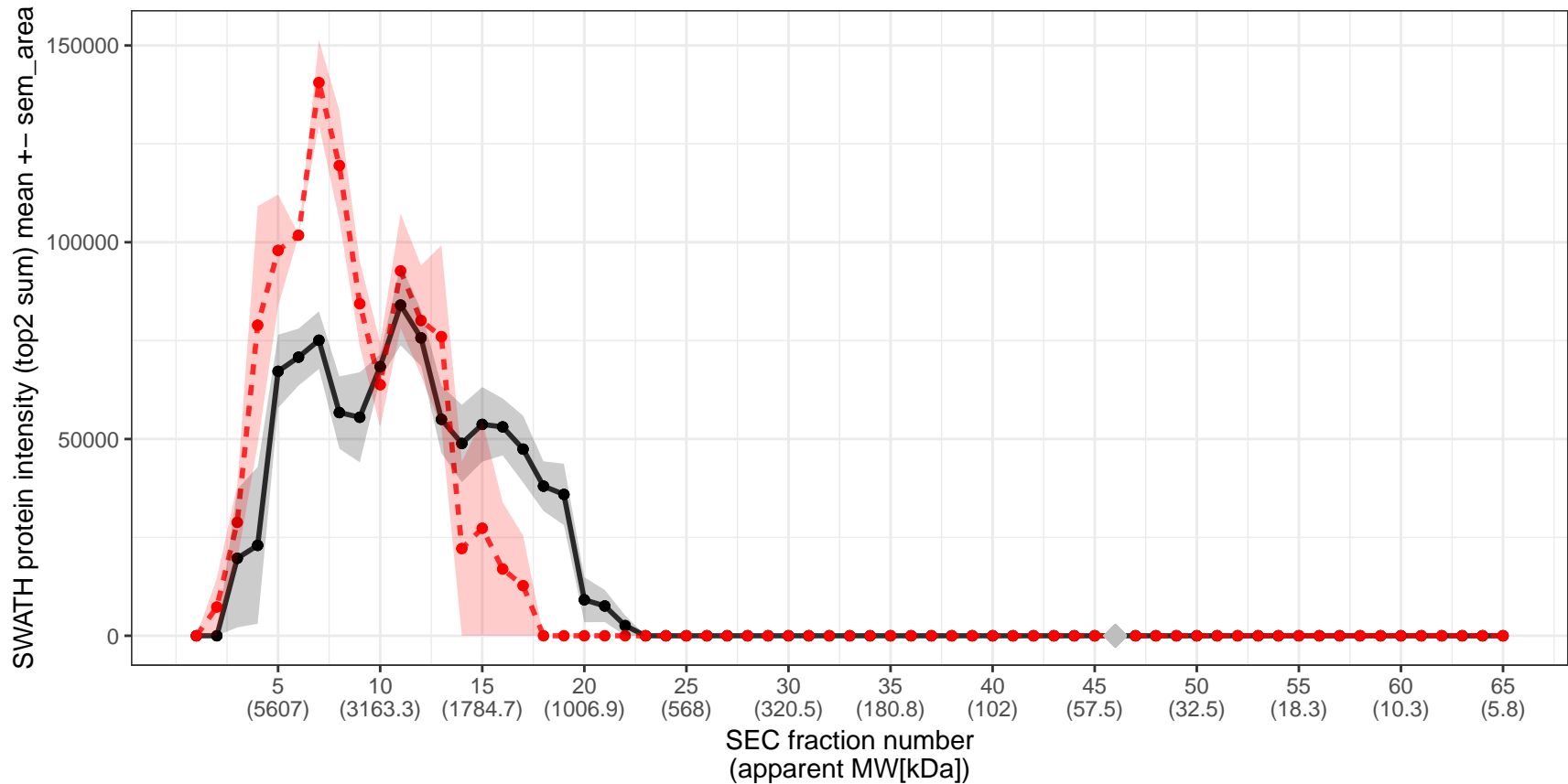

Supplement: Data S1. SEC-SWATH-MS Protein Chromatograms, Related to Figure 1 [file mmc6.zip › SECchrom_O00220_TR10A_HUMAN_TNFRSF10A_APO2_DR4_TRAILR1.pdf]

O00221 | IKBE\_HUMAN | NFKBIE IKBE

Monomer MW [kDa]: 52.864 Monomer expected elution fraction: 46

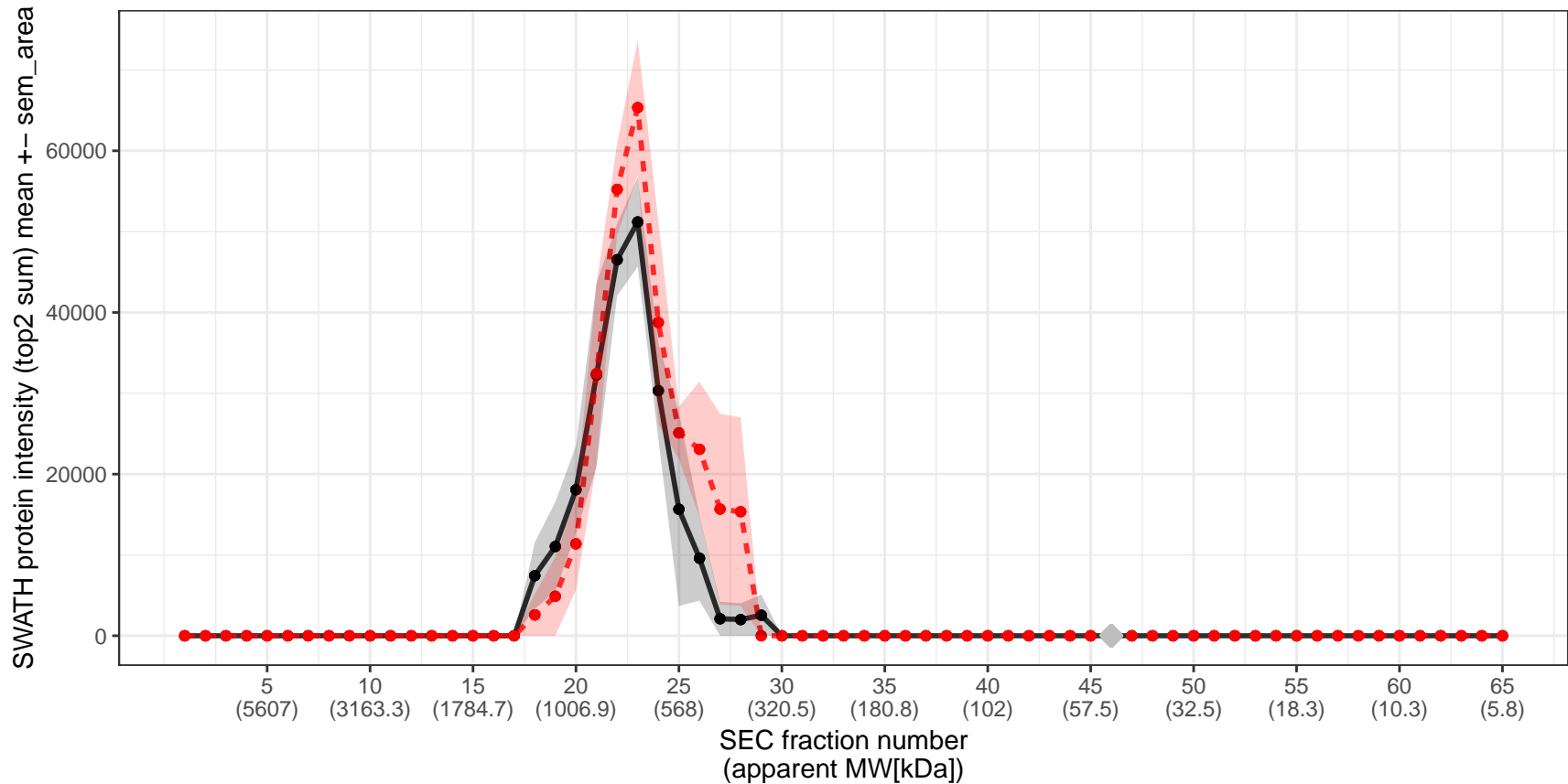

Supplement: Data S1. SEC-SWATH-MS Protein Chromatograms, Related to Figure 1 [file mmc6.zip › SECchrom_O00221_IKBE_HUMAN_NFKBIE_IKBE.pdf]

O00233 | PSMD9\_HUMAN | PSMD9

Monomer MW [kDa]: 24.682 Monomer expected elution fraction: 52

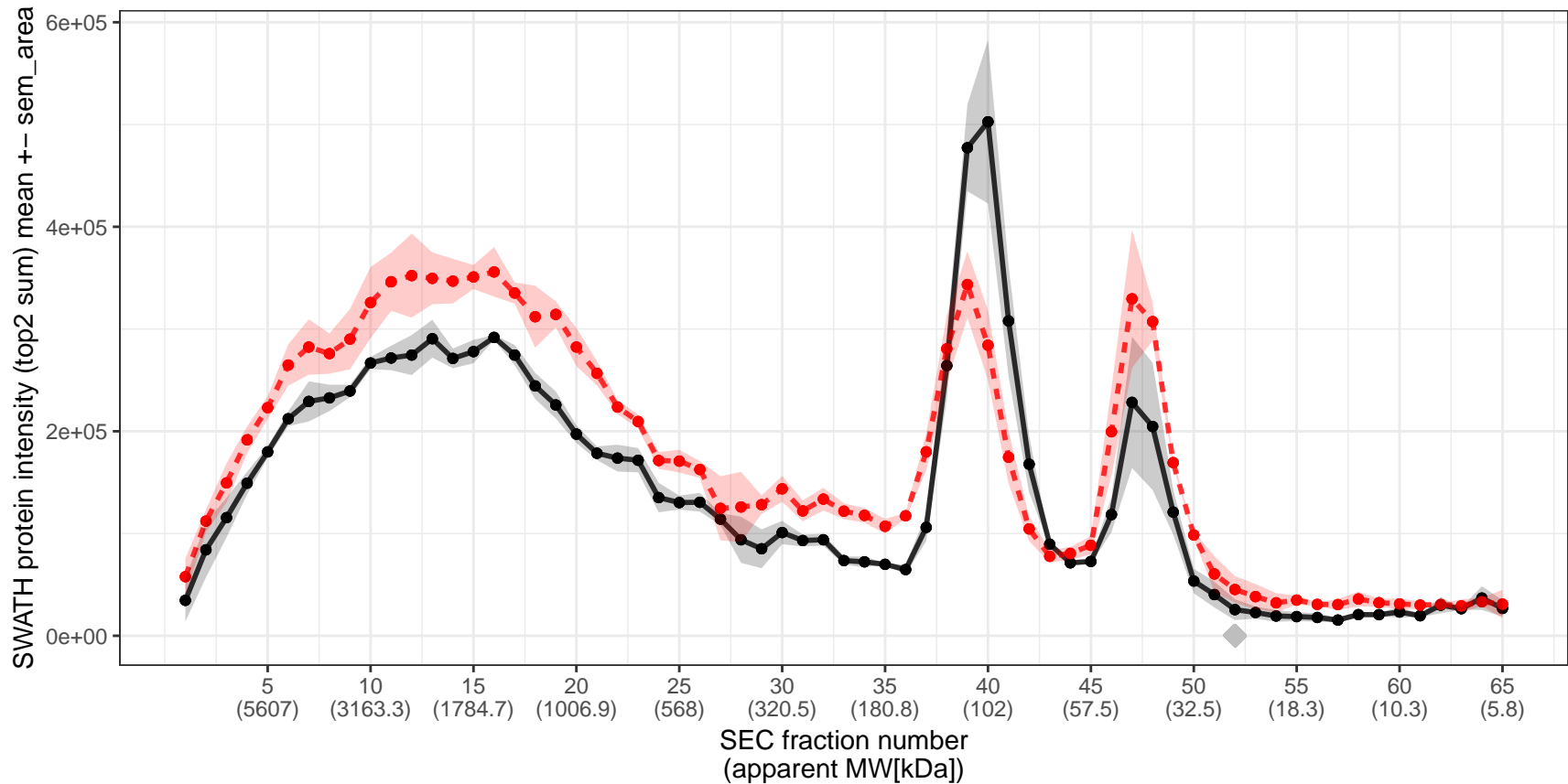

Supplement: Data S1. SEC-SWATH-MS Protein Chromatograms, Related to Figure 1 [file mmc6.zip › SECchrom_O00233_PSMD9_HUMAN_PSMD9.pdf]

O00244 | ATOX1\_HUMAN | ATOX1 HAH1

Monomer MW [kDa]: 7.402 Monomer expected elution fraction: 63

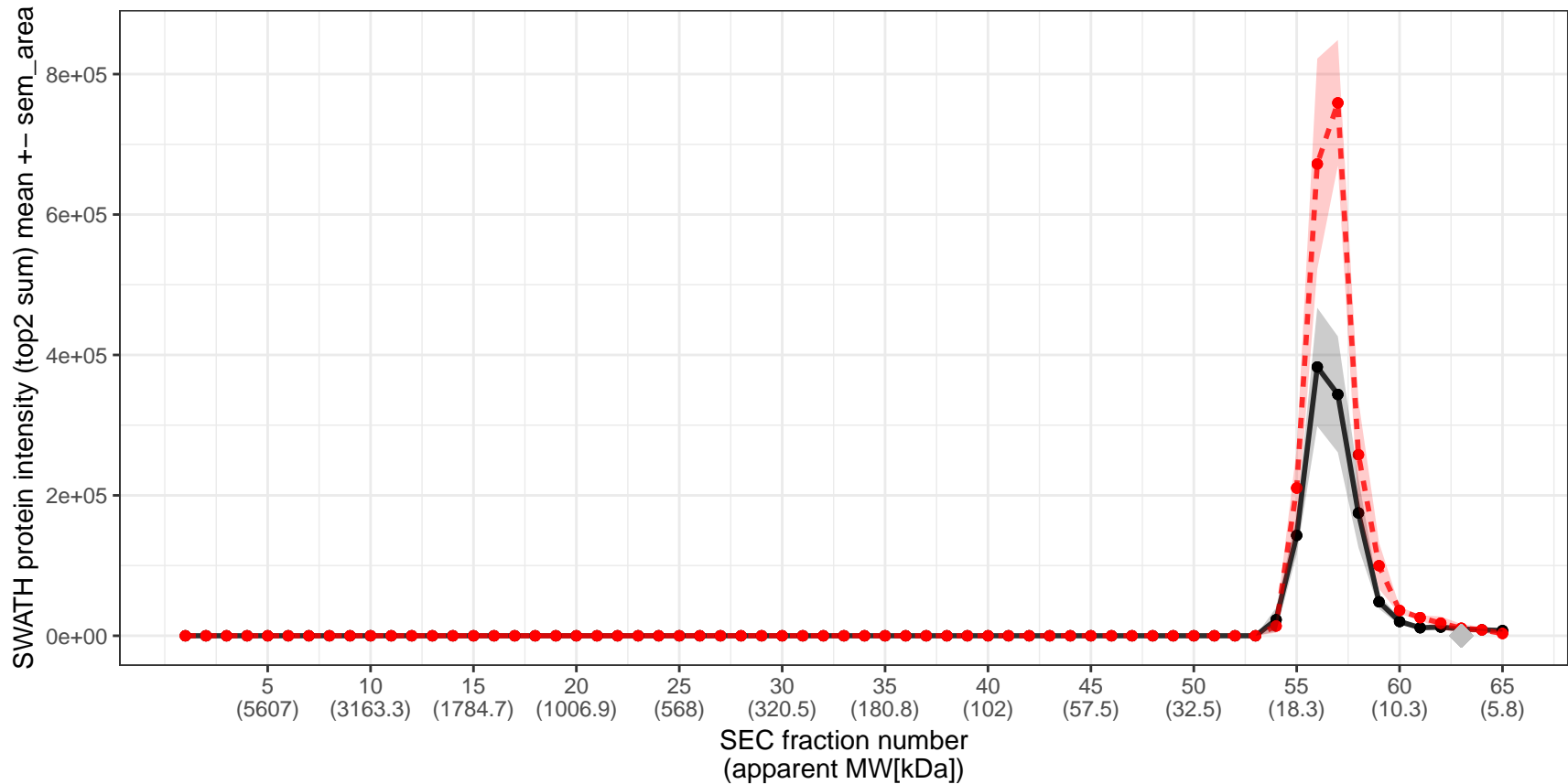

Supplement: Data S1. SEC-SWATH-MS Protein Chromatograms, Related to Figure 1 [file mmc6.zip › SECchrom_O00244_ATOX1_HUMAN_ATOX1_HAH1.pdf]

O00267 | SPT5H\_HUMAN | SUPT5H SPT5 SPT5H

Monomer MW [kDa]: 121 Monomer expected elution fraction: 39

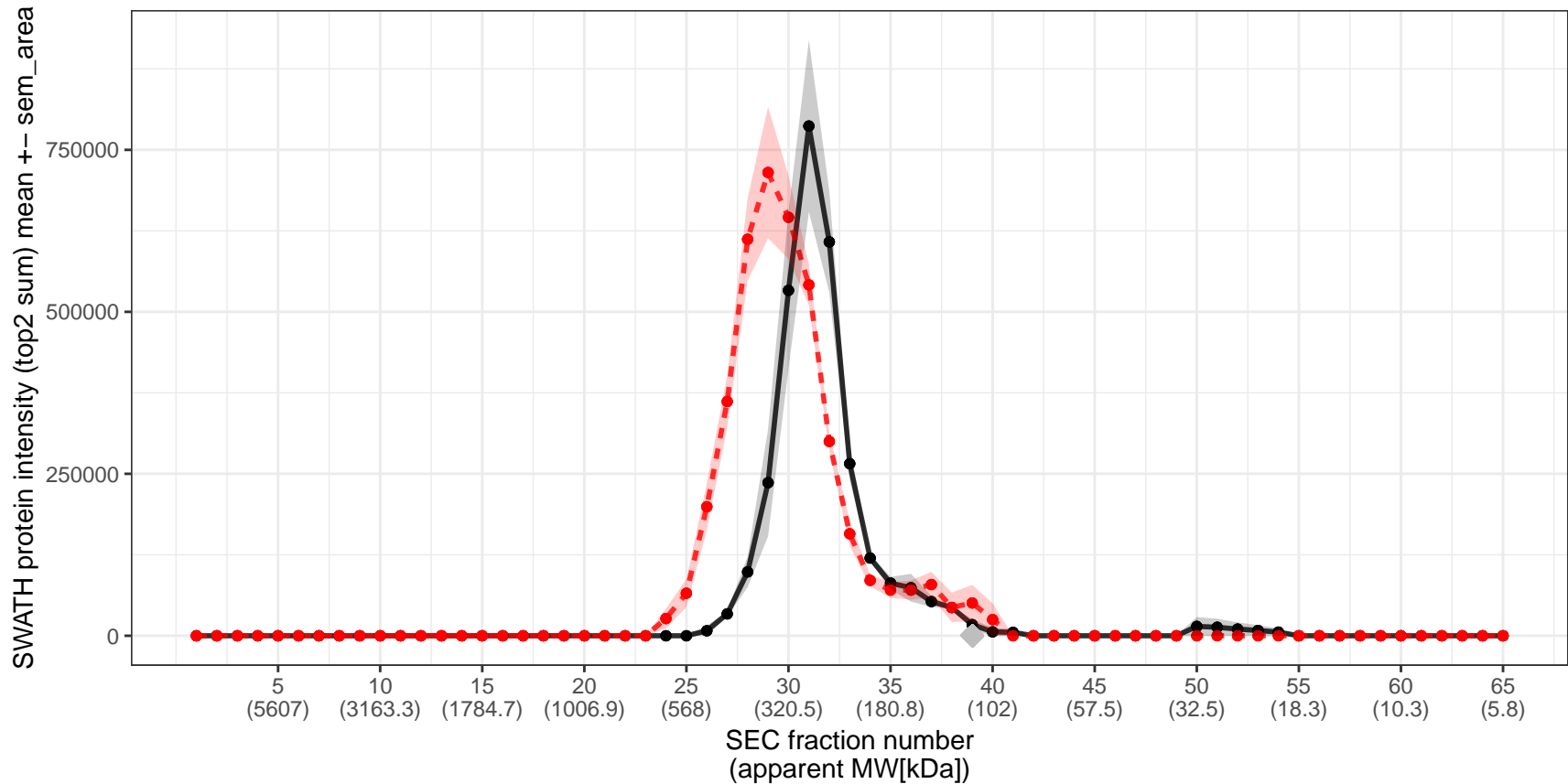

Supplement: Data S1. SEC-SWATH-MS Protein Chromatograms, Related to Figure 1 [file mmc6.zip › SECchrom_O00267_SPT5H_HUMAN_SUPT5H_SPT5_SPT5H.pdf]

O00268 | TAF4\_HUMAN | TAF4 TAF2C TAF2C1 TAF4A TAFII130 TAFII135

Monomer MW [kDa]: 110.114 Monomer expected elution fraction: 39

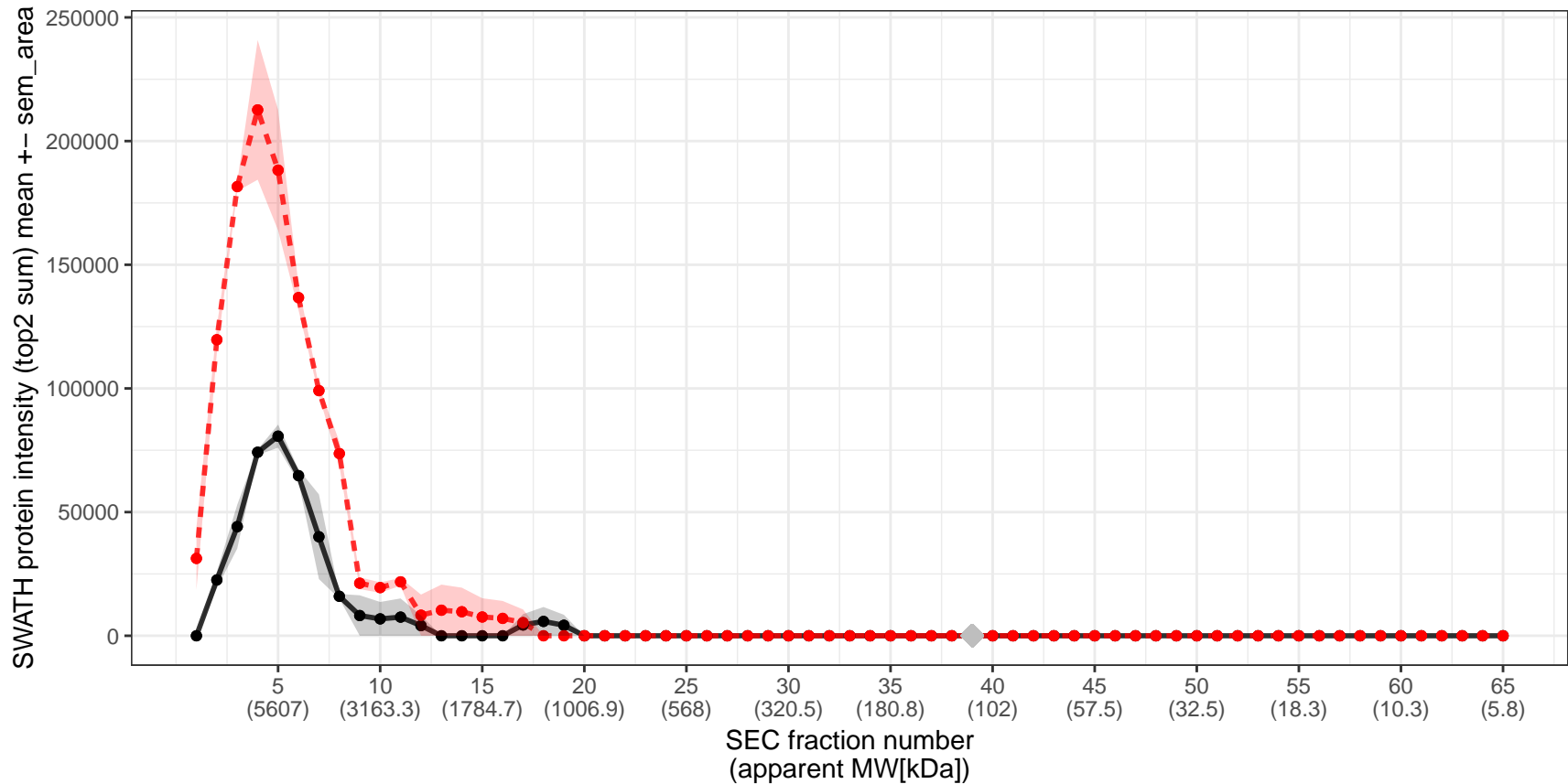

Supplement: Data S1. SEC-SWATH-MS Protein Chromatograms, Related to Figure 1 [file mmc6.zip › SECchrom_O00268_TAF4_HUMAN_TAF4_TAF2C_TAF2C1_TAF4A_TAFII130.pdf]

O00273 | DFFA\_HUMAN | DFFA DFF1 DFF45 H13

Monomer MW [kDa]: 36.522 Monomer expected elution fraction: 49

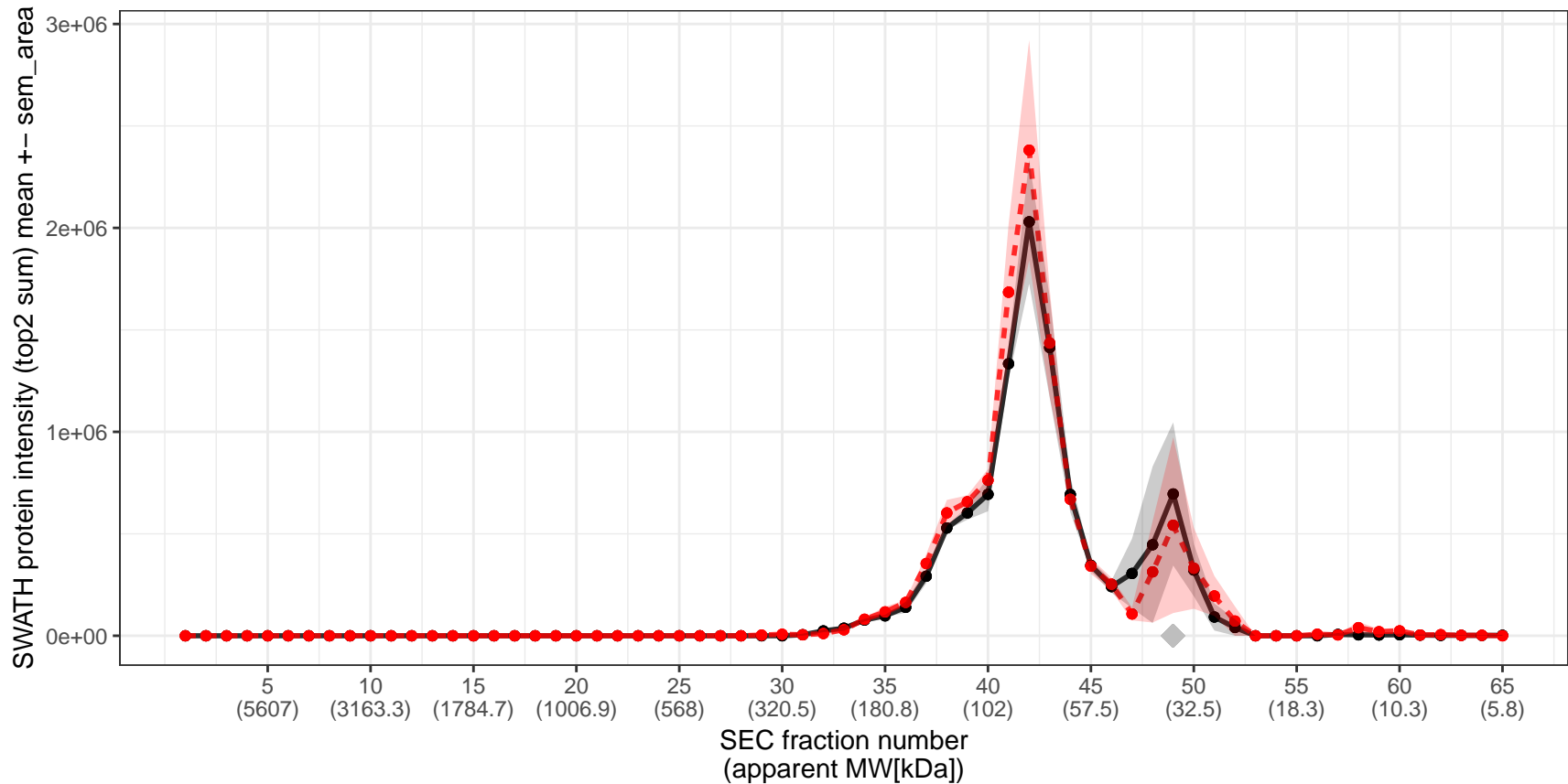

Supplement: Data S1. SEC-SWATH-MS Protein Chromatograms, Related to Figure 1 [file mmc6.zip › SECchrom_O00273_DFFA_HUMAN_DFFA_DFF1_DFF45_H13.pdf]

O00291 | HIP1\_HUMAN | HIP1

Monomer MW [kDa]: 116.221 Monomer expected elution fraction: 39

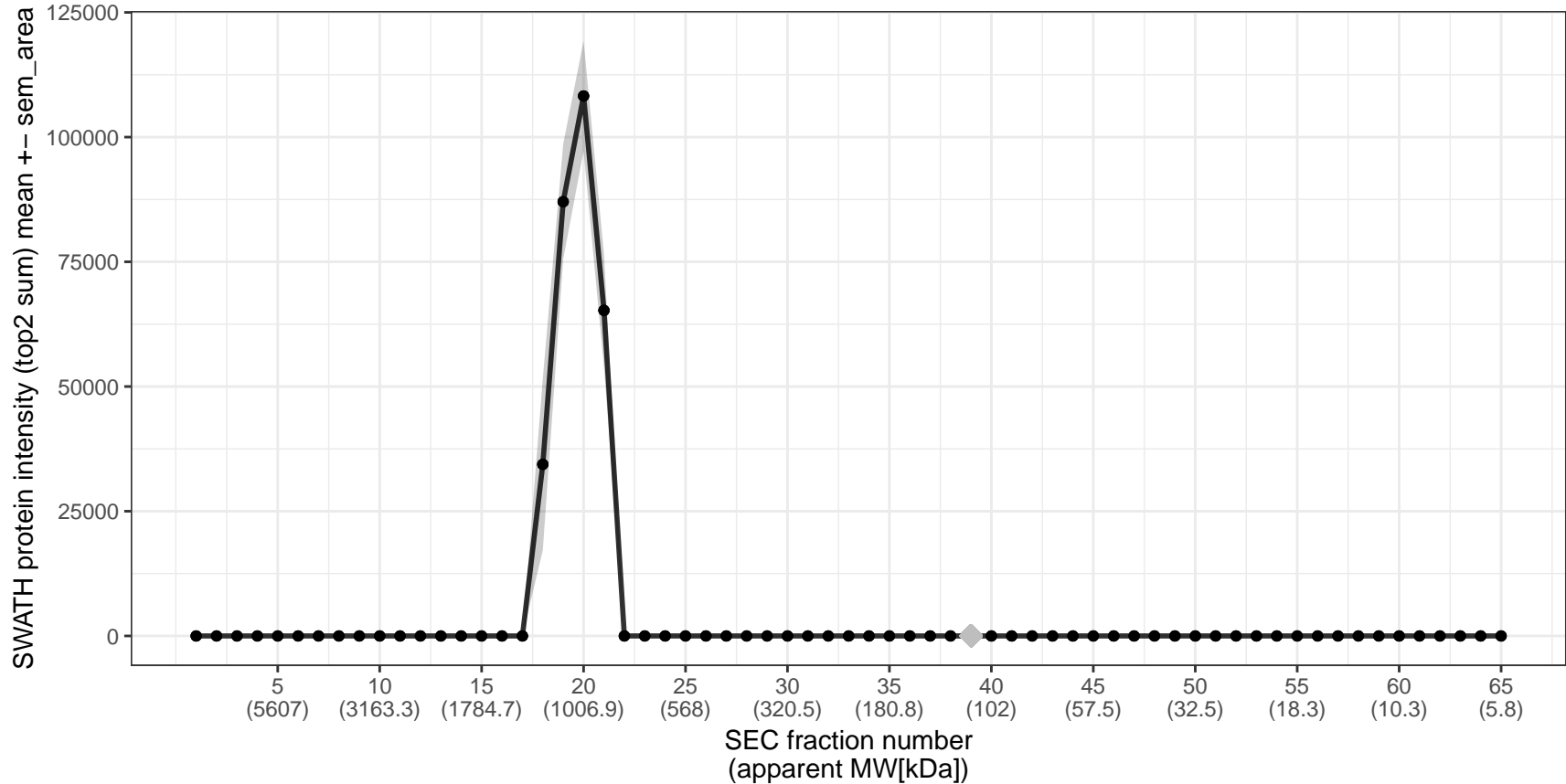

Supplement: Data S1. SEC-SWATH-MS Protein Chromatograms, Related to Figure 1 [file mmc6.zip › SECchrom_O00291_HIP1_HUMAN_HIP1.pdf]

O00299 | CLIC1\_HUMAN | CLIC1 G6 NCC27

Monomer MW [kDa]: 26.923 Monomer expected elution fraction: 52

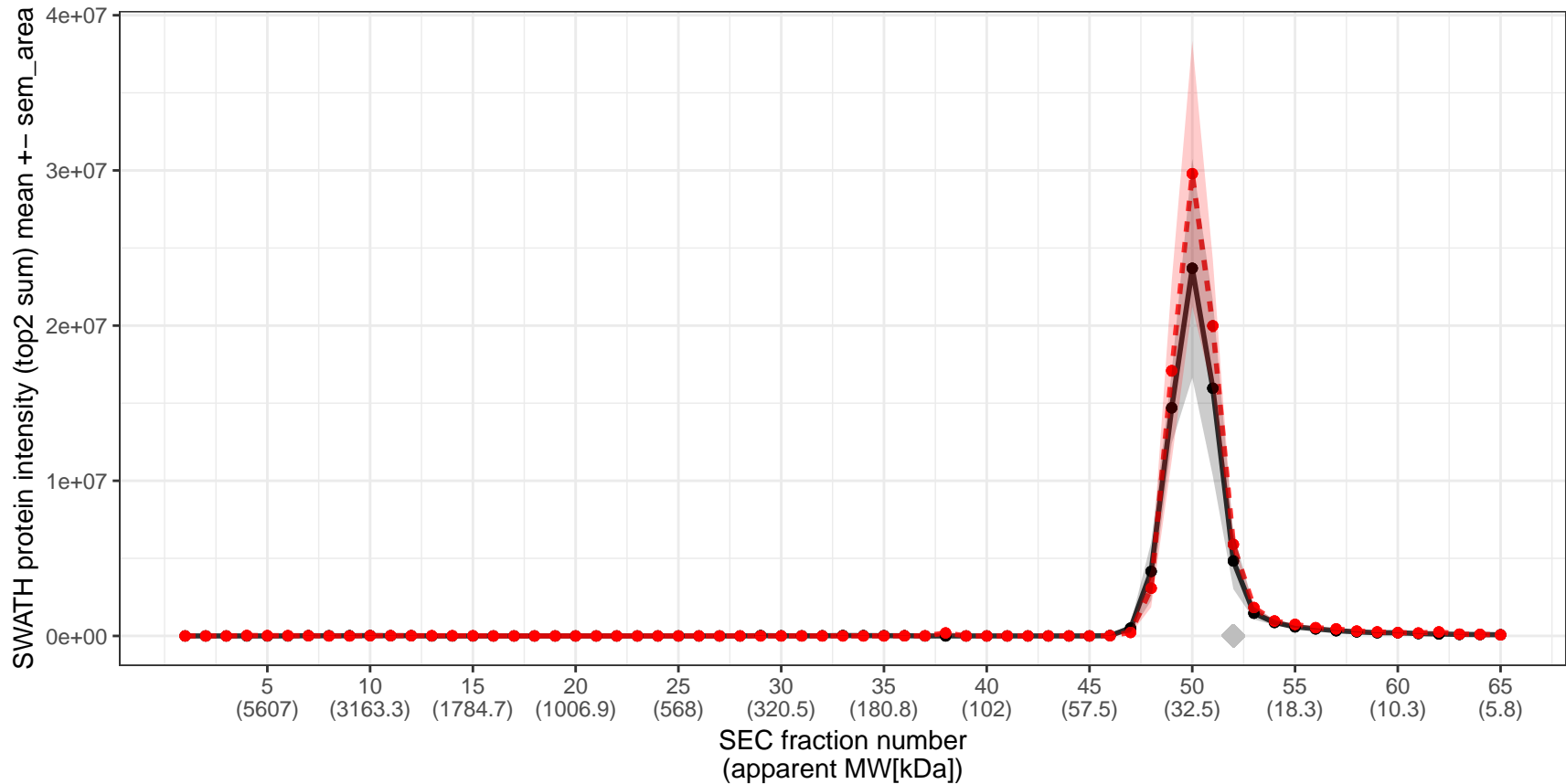

Supplement: Data S1. SEC-SWATH-MS Protein Chromatograms, Related to Figure 1 [file mmc6.zip › SECchrom_O00299_CLIC1_HUMAN_CLIC1_G6_NCC27.pdf]

O00303 | EIF3F\_HUMAN | EIF3F EIF3S5

Monomer MW [kDa]: 37.564 Monomer expected elution fraction: 49

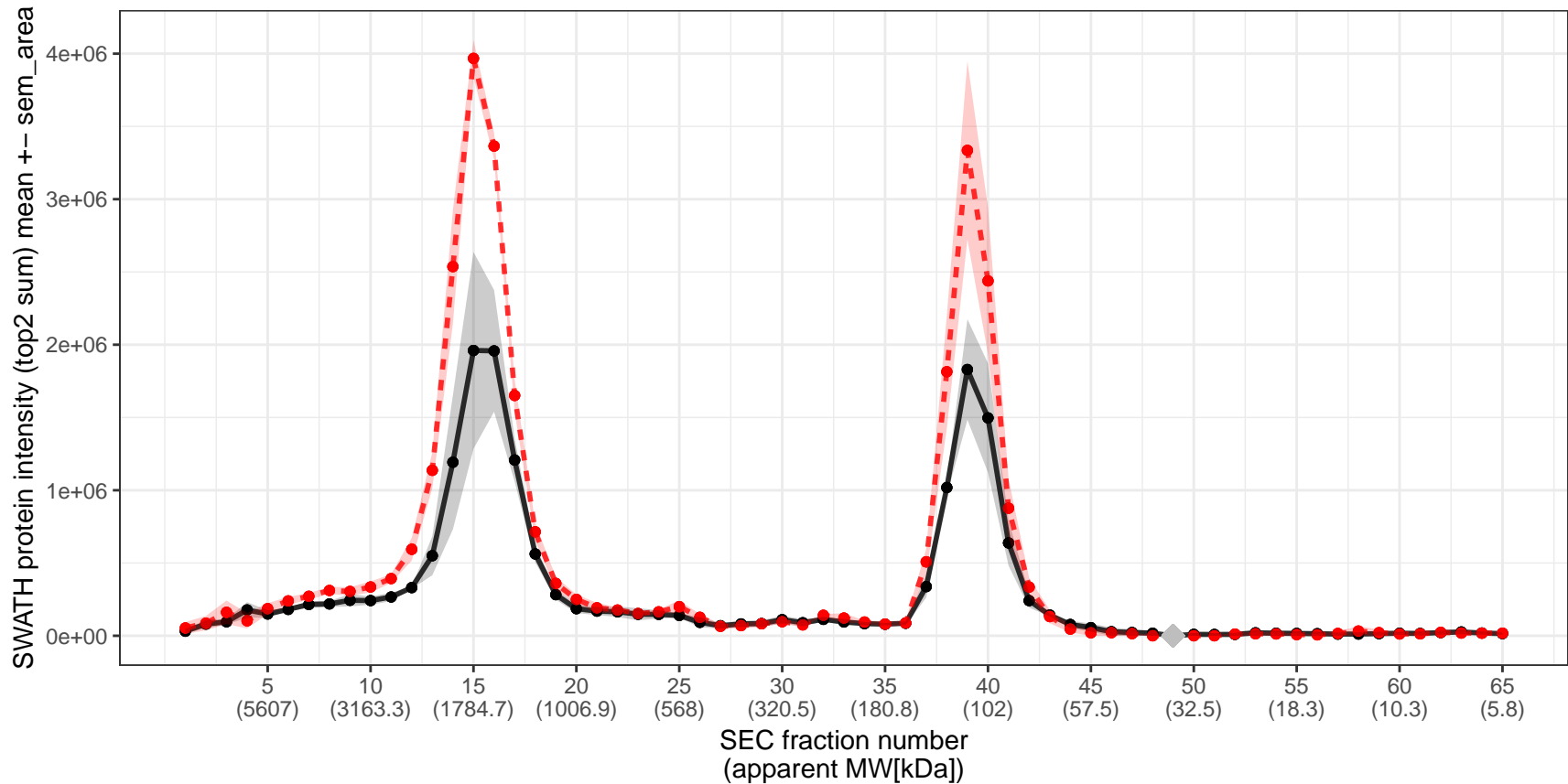

Supplement: Data S1. SEC-SWATH-MS Protein Chromatograms, Related to Figure 1 [file mmc6.zip › SECchrom_O00303_EIF3F_HUMAN_EIF3F_EIF3S5.pdf]

O00330 | ODPX\_HUMAN | PDHX PDX1

Monomer MW [kDa]: 54.122 Monomer expected elution fraction: 46

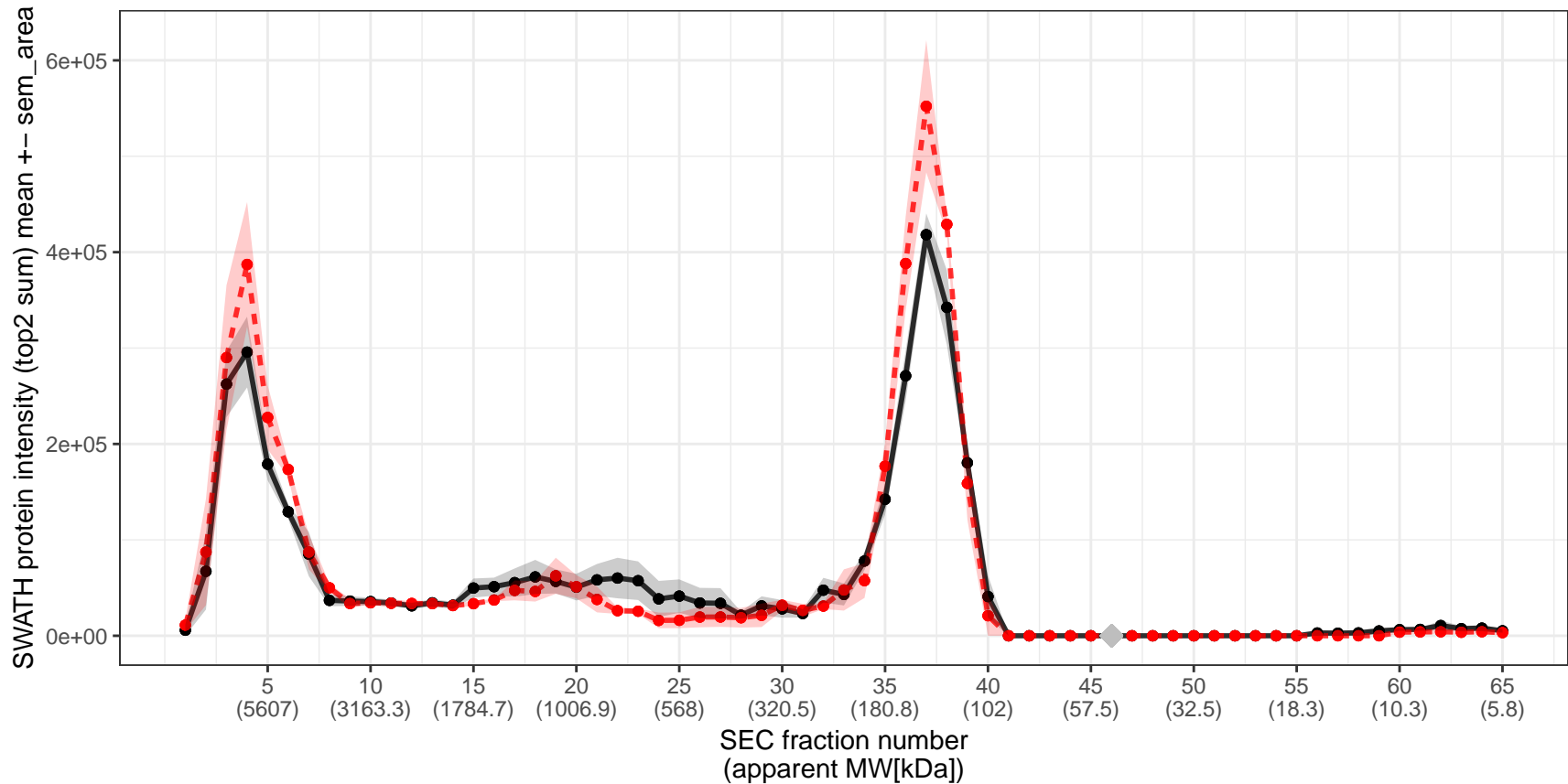

Supplement: Data S1. SEC-SWATH-MS Protein Chromatograms, Related to Figure 1 [file mmc6.zip › SECchrom_O00330_ODPX_HUMAN_PDHX_PDX1.pdf]

O00399 | DCTN6\_HUMAN | DCTN6 WS3

Monomer MW [kDa]: 20.747 Monomer expected elution fraction: 54

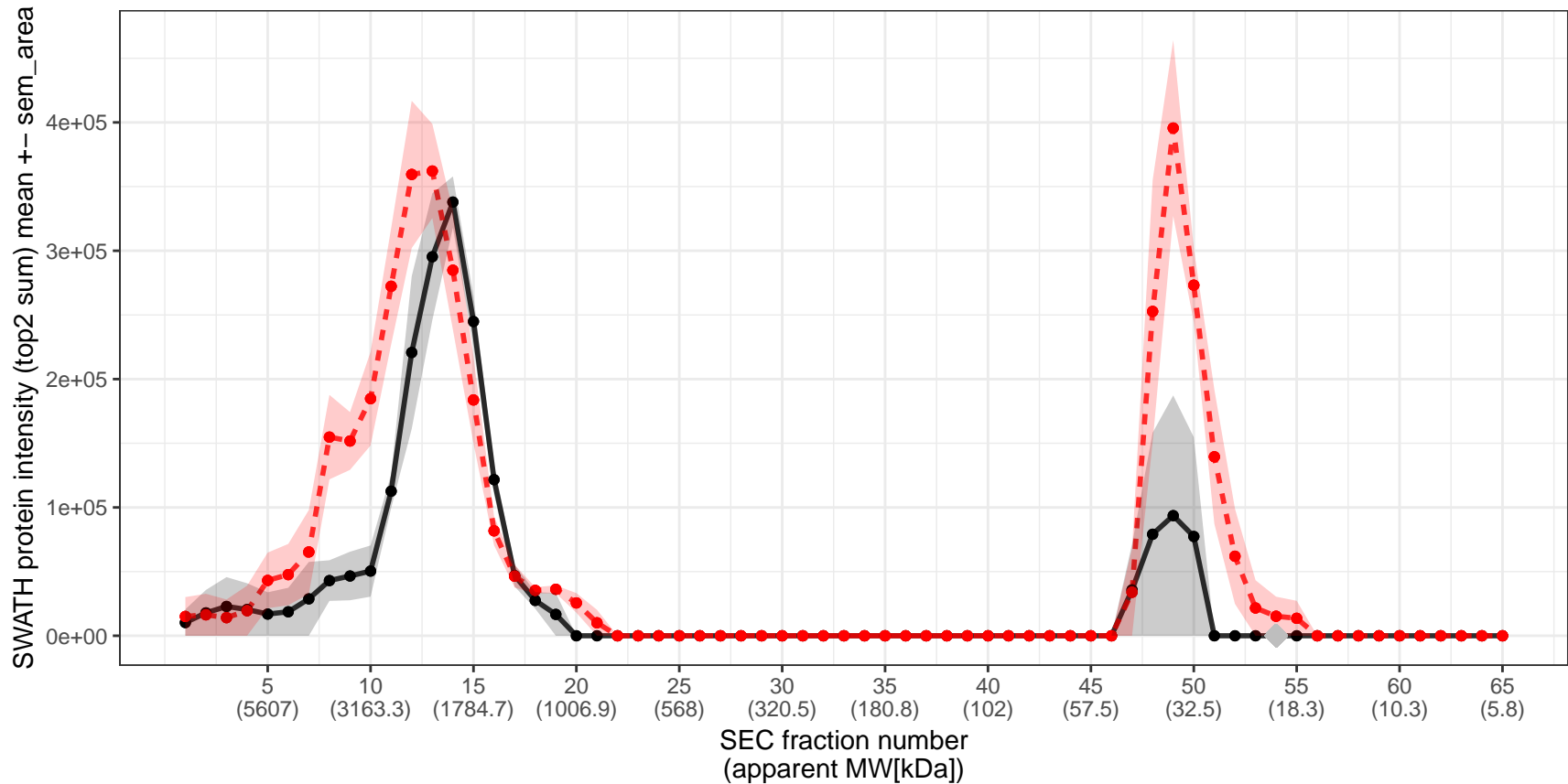

Supplement: Data S1. SEC-SWATH-MS Protein Chromatograms, Related to Figure 1 [file mmc6.zip › SECchrom_O00399_DCTN6_HUMAN_DCTN6_WS3.pdf]

O00400 | ACATN\_HUMAN | SLC33A1 ACATN AT1

Monomer MW [kDa]: 60.909 Monomer expected elution fraction: 45

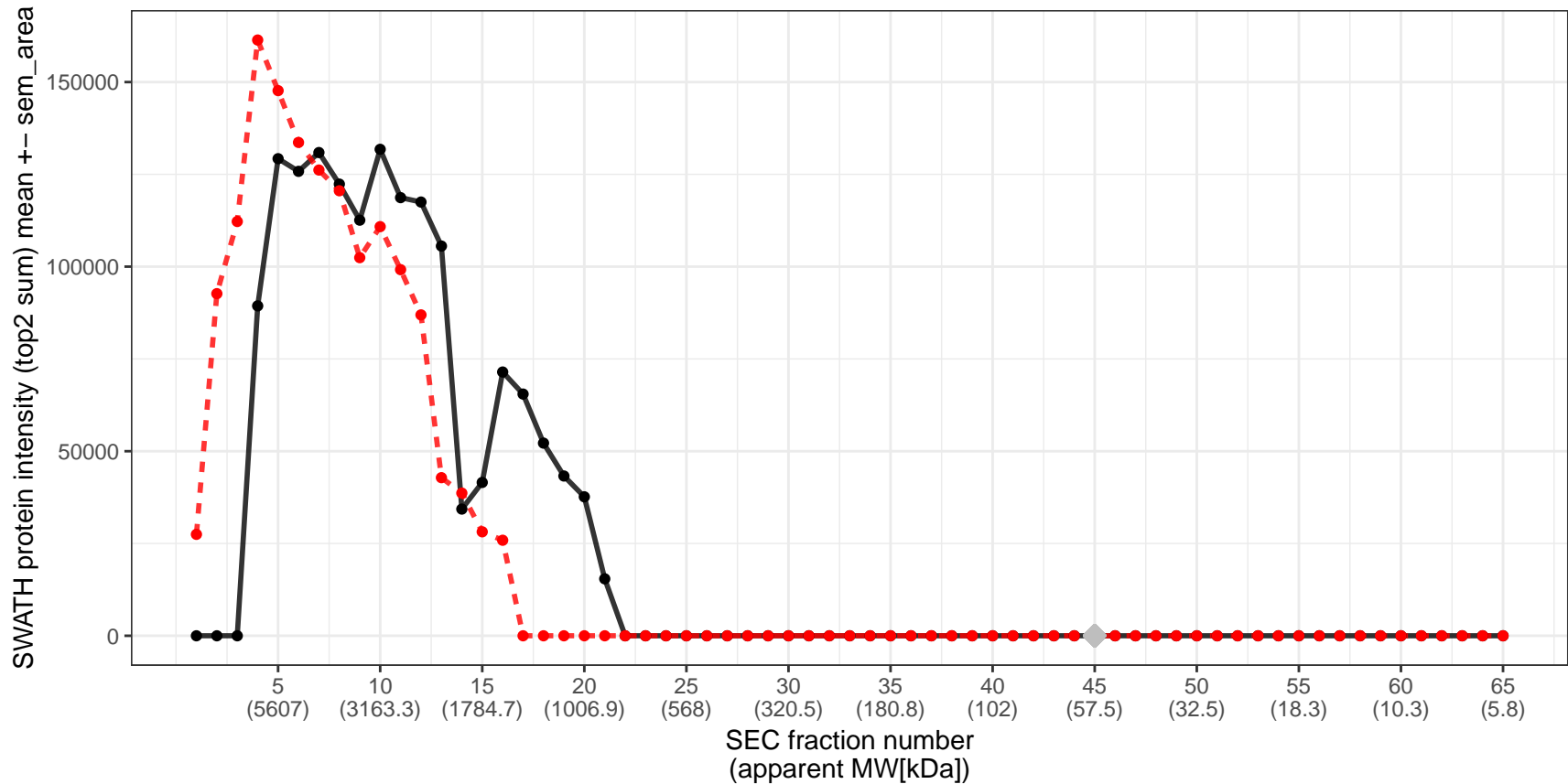

Supplement: Data S1. SEC-SWATH-MS Protein Chromatograms, Related to Figure 1 [file mmc6.zip › SECchrom_O00400_ACATN_HUMAN_SLC33A1_ACATN_AT1.pdf]

O00401 | WASL\_HUMAN | WASL

Monomer MW [kDa]: 54.827 Monomer expected elution fraction: 45

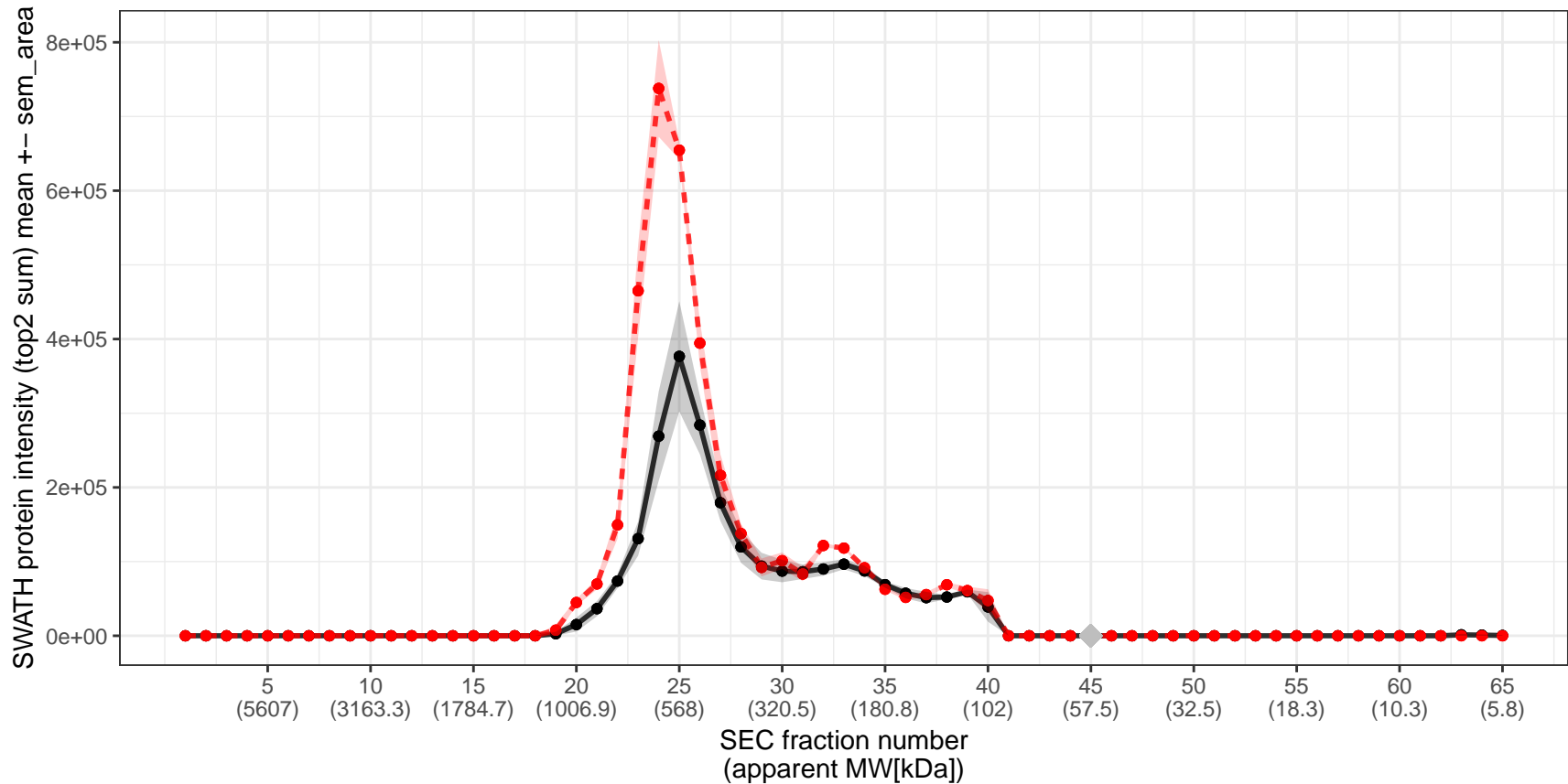

Supplement: Data S1. SEC-SWATH-MS Protein Chromatograms, Related to Figure 1 [file mmc6.zip › SECchrom_O00401_WASL_HUMAN_WASL.pdf]

O00411 | RPOM\_HUMAN | POLRMT

Monomer MW [kDa]: 138.62 Monomer expected elution fraction: 37

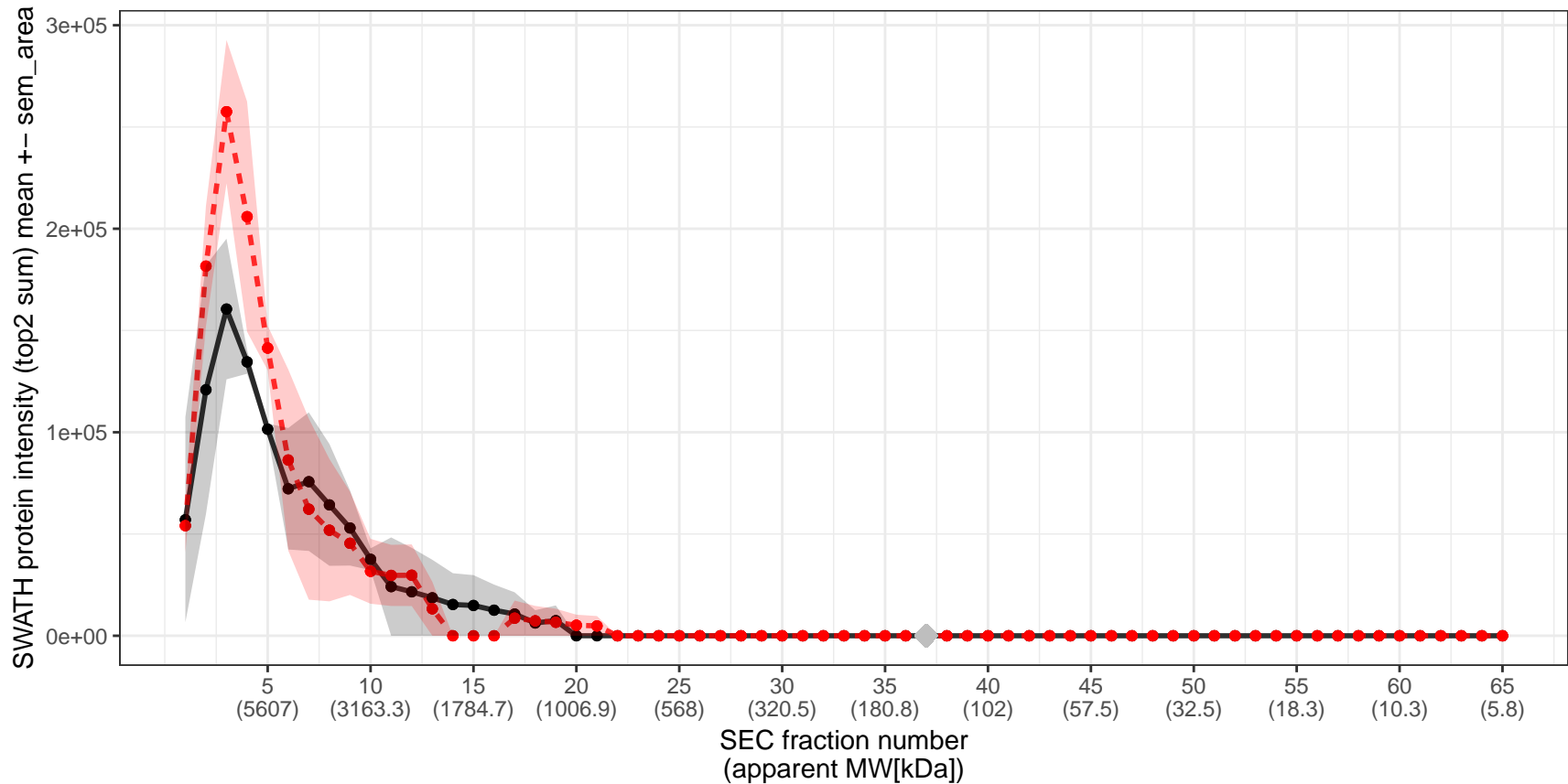

Supplement: Data S1. SEC-SWATH-MS Protein Chromatograms, Related to Figure 1 [file mmc6.zip › SECchrom_O00411_RPOM_HUMAN_POLRMT.pdf]

O00418 | EF2K\_HUMAN | EF2K

Monomer MW [kDa]: 82.144 Monomer expected elution fraction: 42

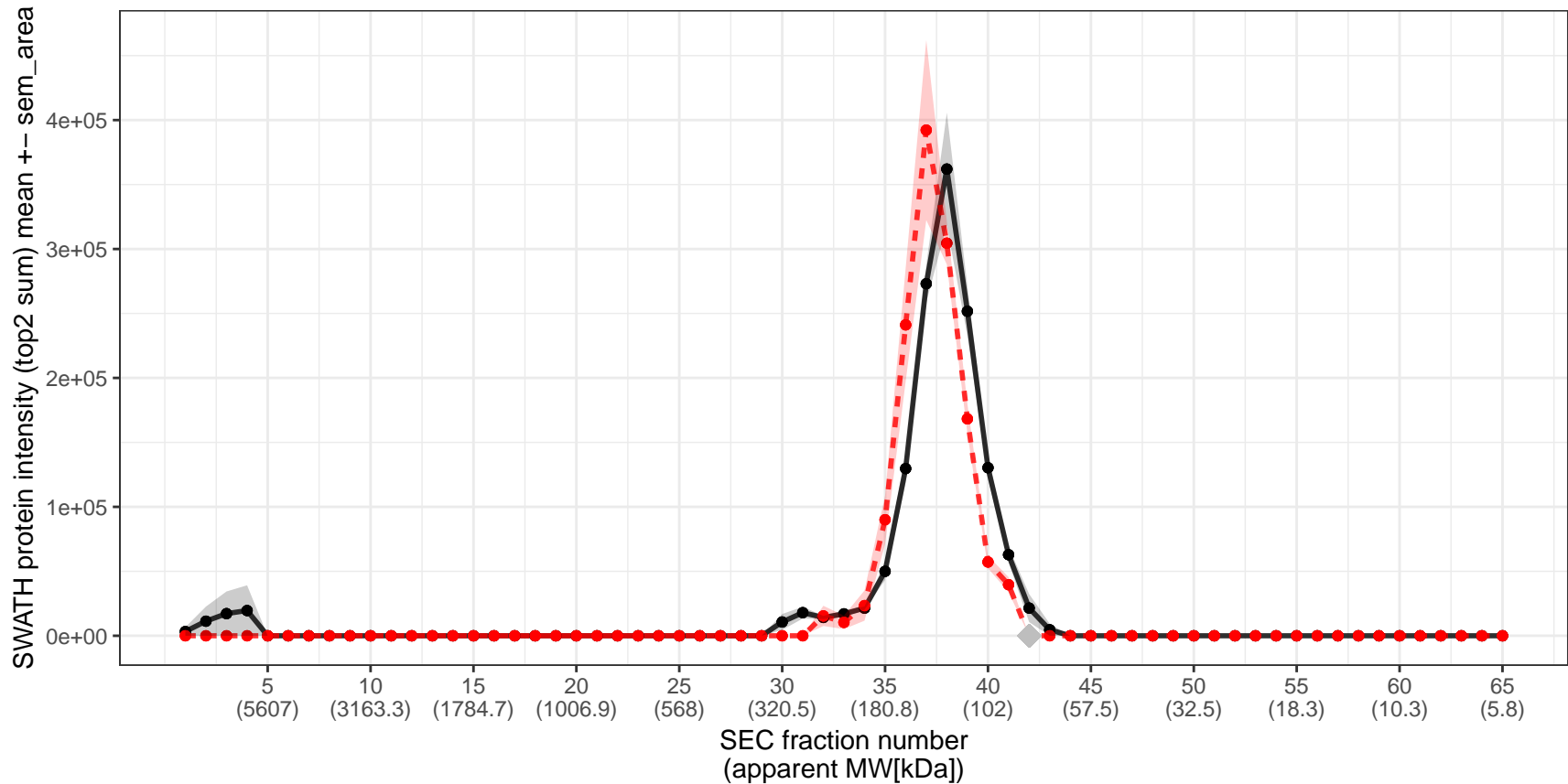

Supplement: Data S1. SEC-SWATH-MS Protein Chromatograms, Related to Figure 1 [file mmc6.zip › SECchrom_O00418_EF2K_HUMAN_EEF2K.pdf]

O00425 | IF2B3\_HUMAN | IGF2BP3 IMP3 KOC1 VICKZ3  
Monomer MW [kDa]: 63.705 Monomer expected elution fraction: 44

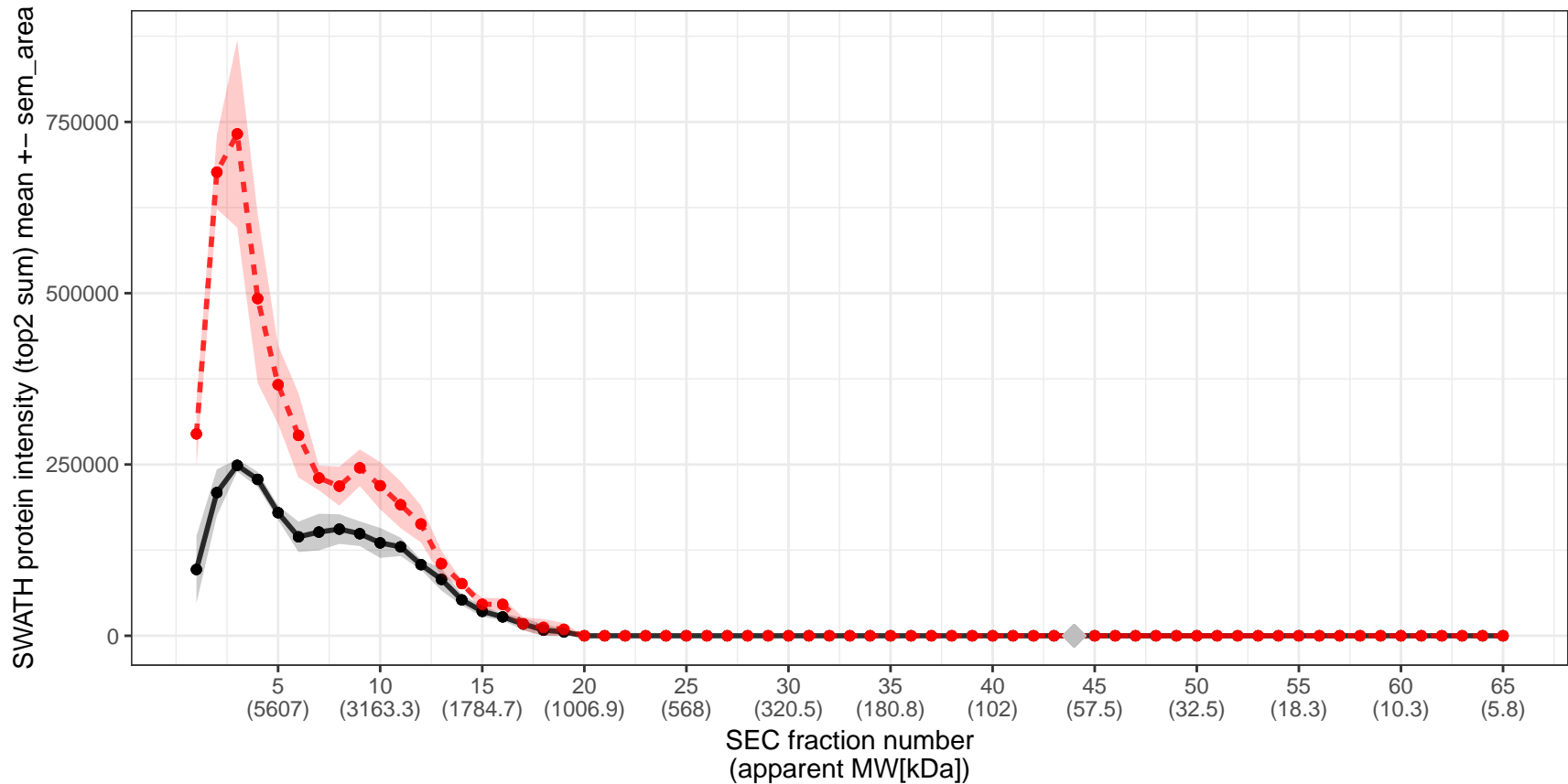

Supplement: Data S1. SEC-SWATH-MS Protein Chromatograms, Related to Figure 1 [file mmc6.zip › SECchrom_O00425_IF2B3_HUMAN_IGF2BP3_IMP3_KOC1_VICKZ3.pdf]

O00429 | DNM1L\_HUMAN | DNM1L DLP1 DRP1

Monomer MW [kDa]: 81.877 Monomer expected elution fraction: 42

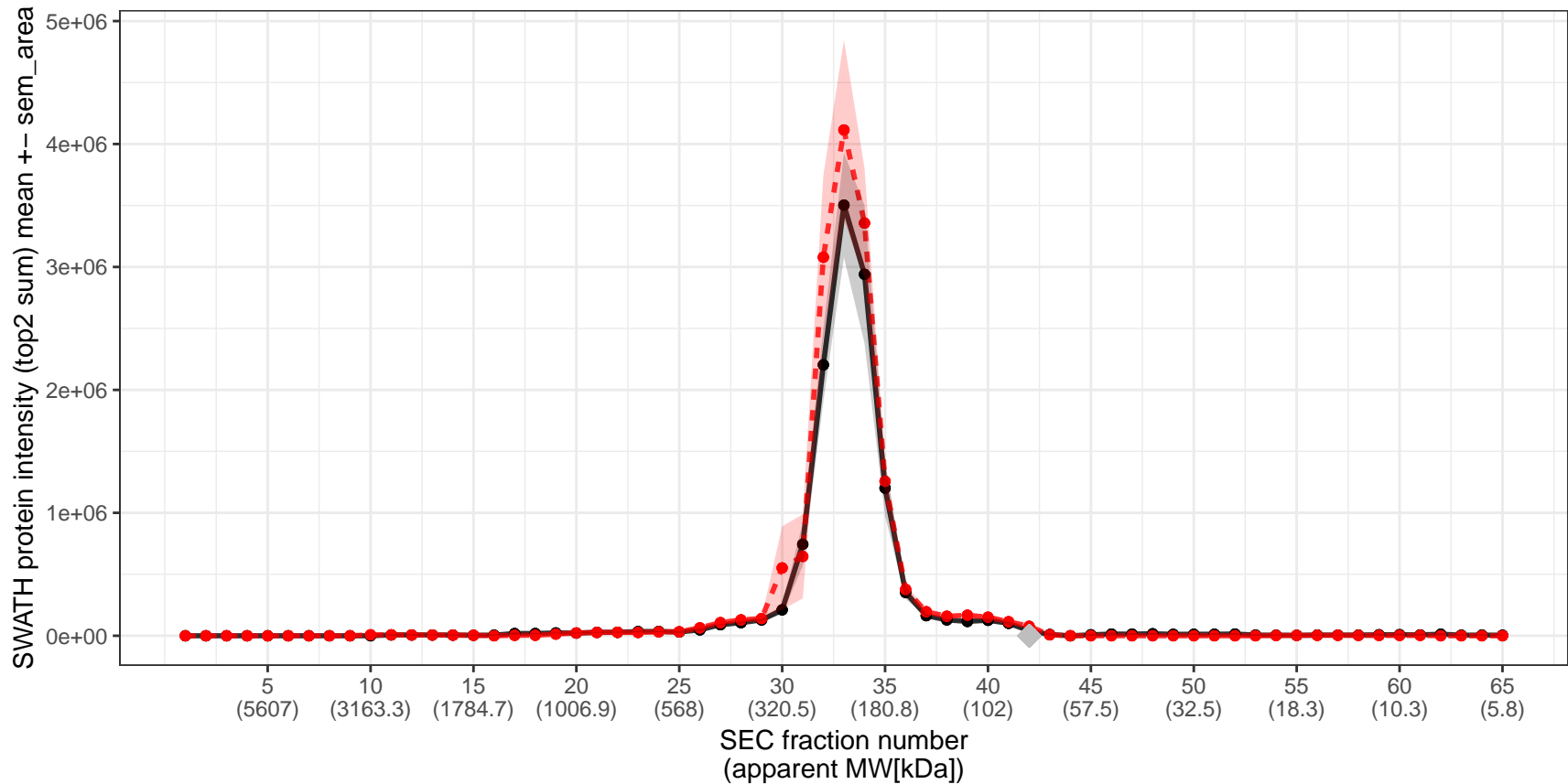

Supplement: Data S1. SEC-SWATH-MS Protein Chromatograms, Related to Figure 1 [file mmc6.zip › SECchrom_O00429_DNM1L_HUMAN_DNM1L_DLP1_DRP1.pdf]

O00442 | RTCA\_HUMAN | RTCA RPC RPC1 RTC1 RTCD1  
Monomer MW [kDa]: 39.337 Monomer expected elution fraction: 48

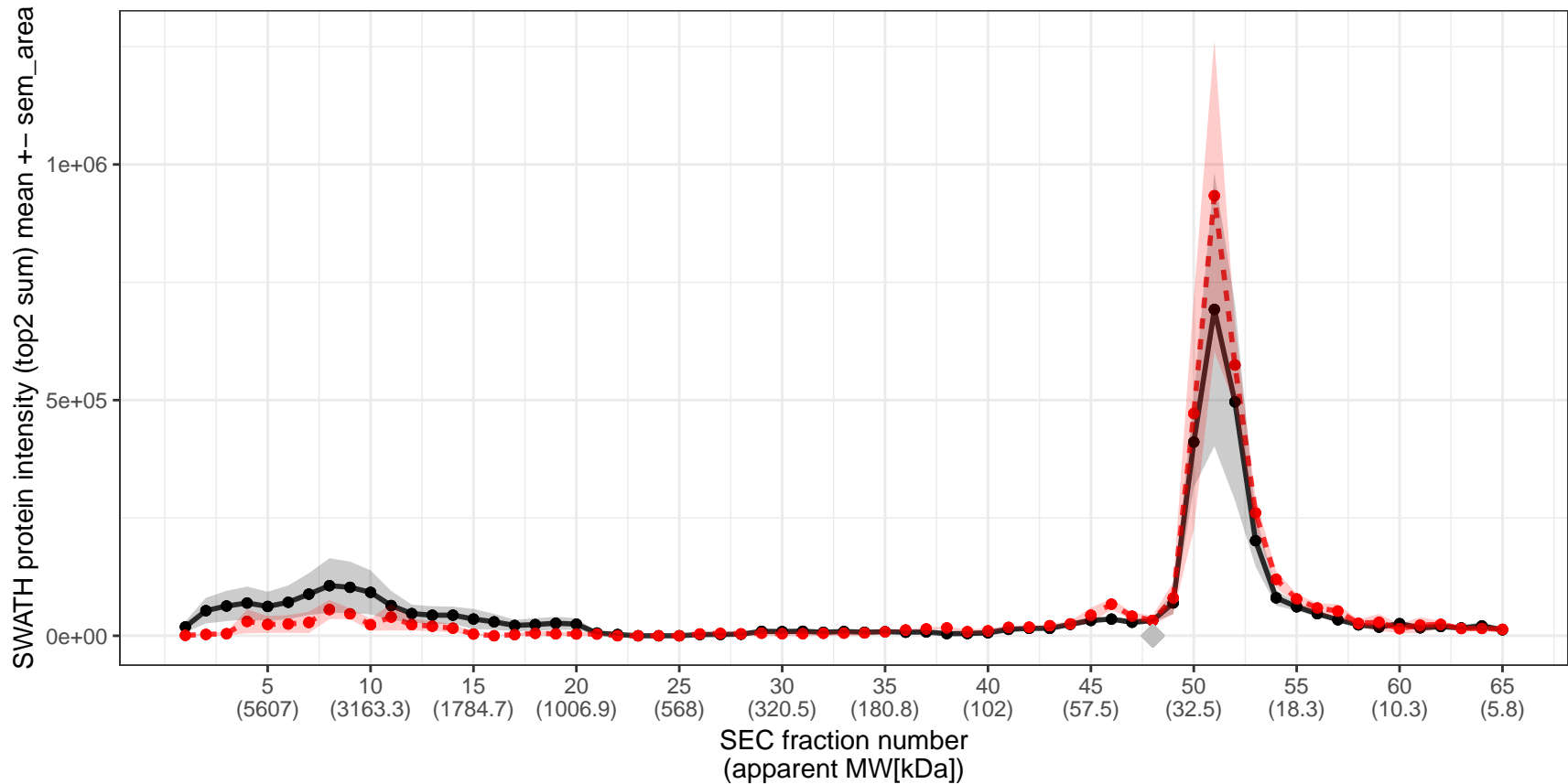

Supplement: Data S1. SEC-SWATH-MS Protein Chromatograms, Related to Figure 1 [file mmc6.zip › SECchrom_O00442_RTCA_HUMAN_RTCA_RPC_RPC1_RTC1_RTCD1.pdf]

O00461 | GOLI4\_HUMAN | GOLIM4 GIMPC GOLPH4 GPP130  
Monomer MW [kDa]: 81.88 Monomer expected elution fraction: 42

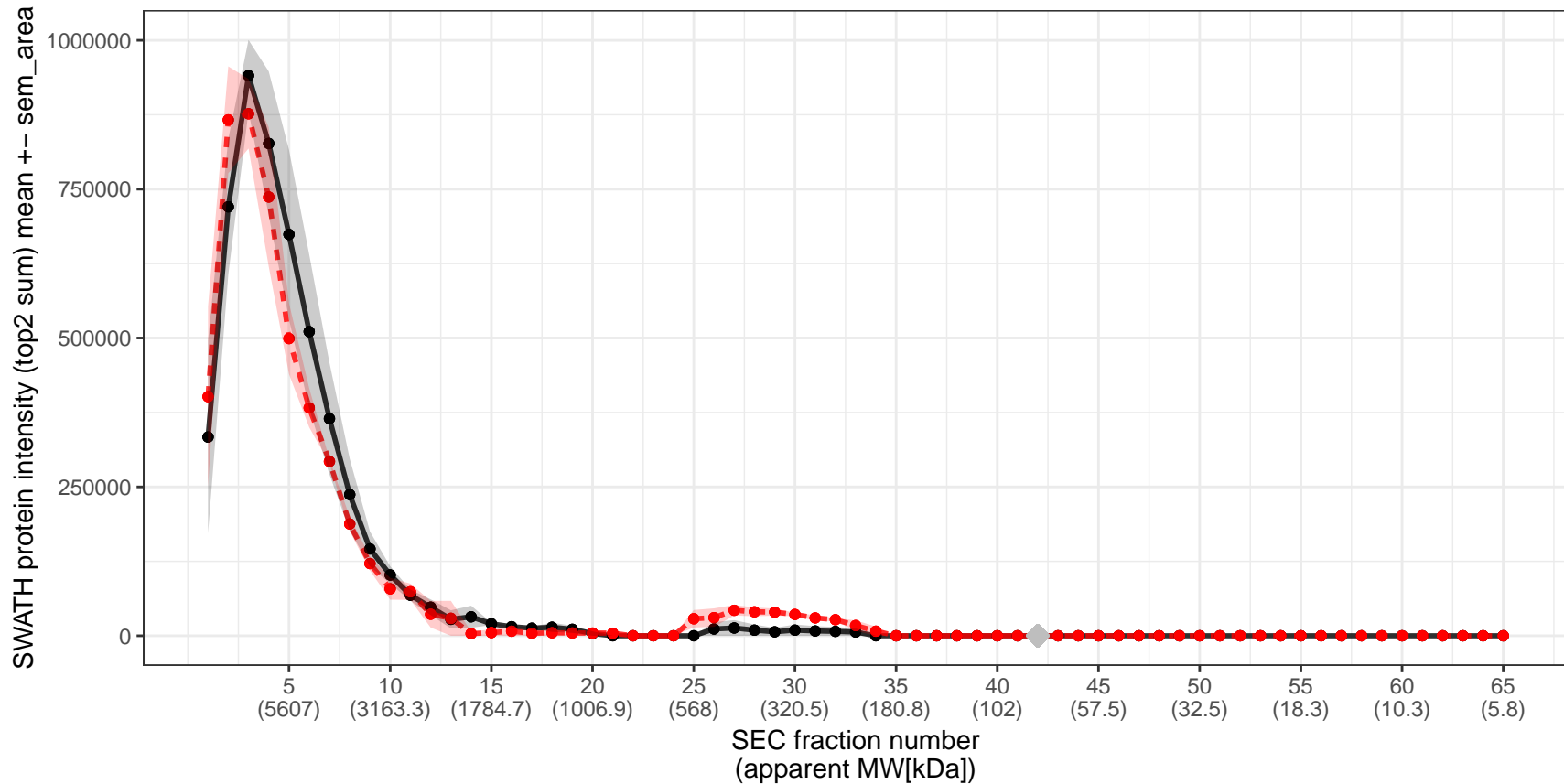

Supplement: Data S1. SEC-SWATH-MS Protein Chromatograms, Related to Figure 1 [file mmc6.zip › SECchrom_O00461_GOLI4_HUMAN_GOLIM4_GIMPC_GOLPH4_GPP130.pdf]

O00462 | MANBA\_HUMAN | MANBA MANB1

Monomer MW [kDa]: 100.895 Monomer expected elution fraction: 40

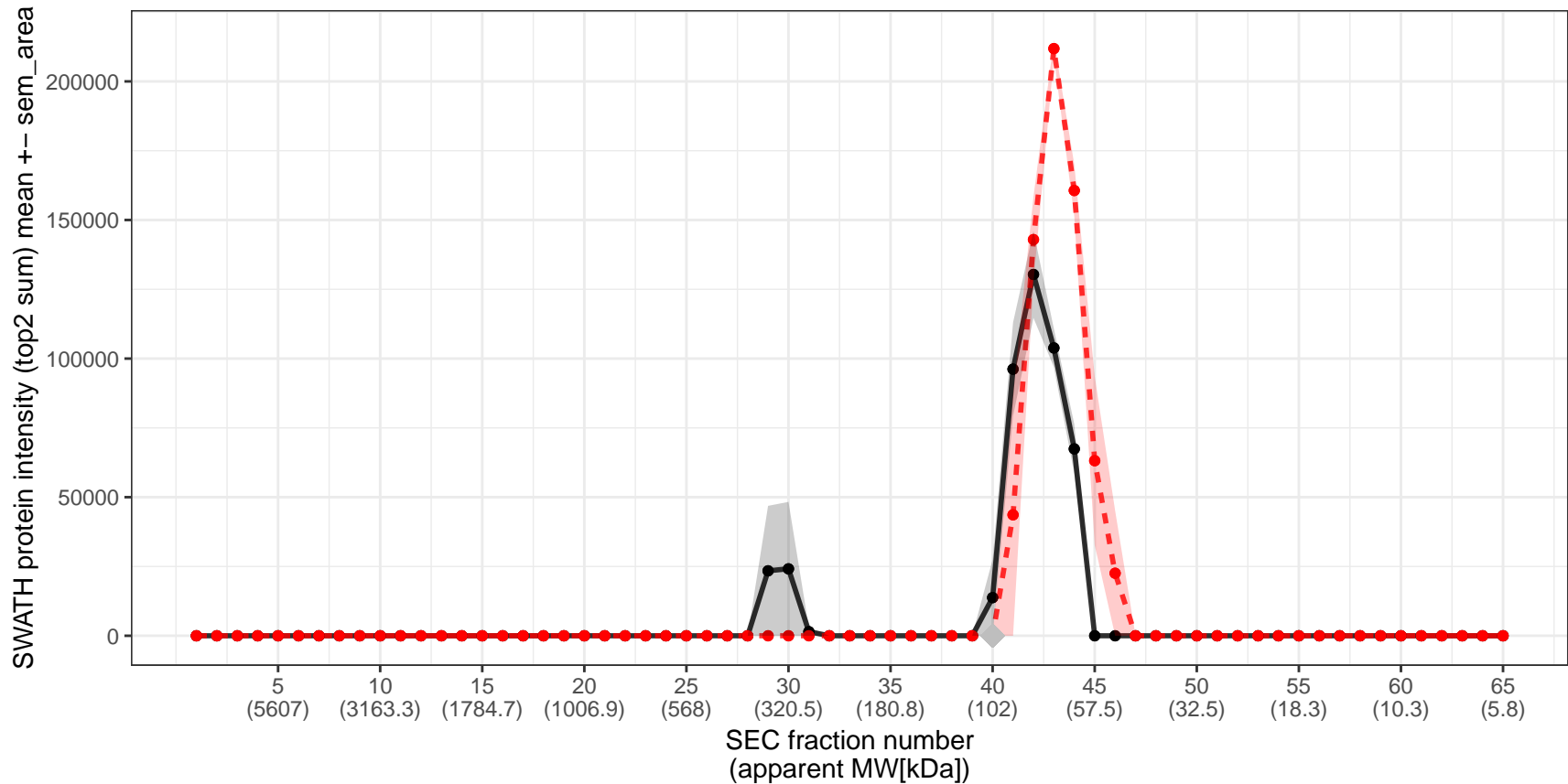

Supplement: Data S1. SEC-SWATH-MS Protein Chromatograms, Related to Figure 1 [file mmc6.zip › SECchrom_O00462_MANBA_HUMAN_MANBA_MANB1.pdf]

O00468 | AGRIN\_HUMAN | AGRN AGRIN

Monomer MW [kDa]: 217.232 Monomer expected elution fraction: 33

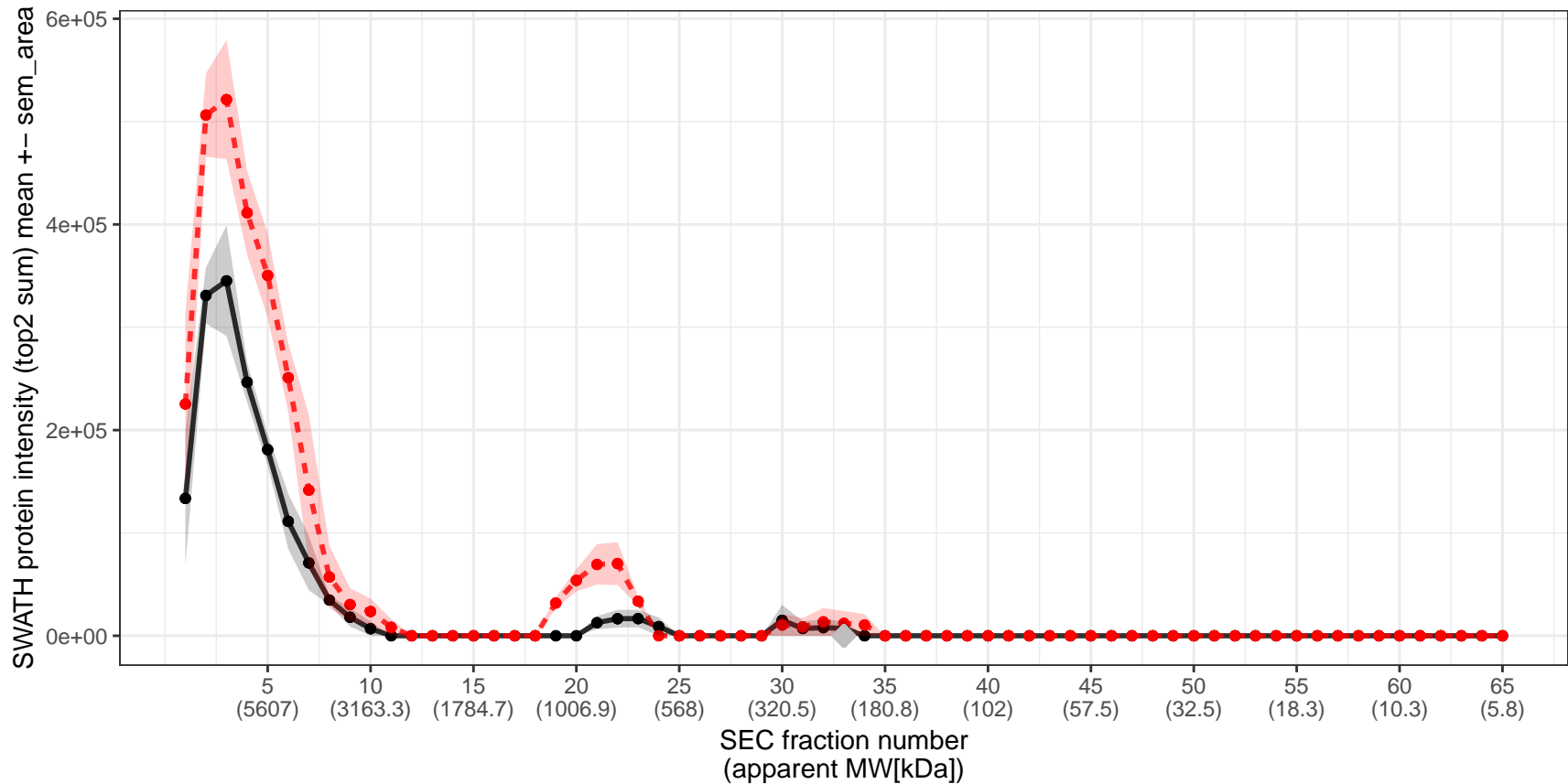

Supplement: Data S1. SEC-SWATH-MS Protein Chromatograms, Related to Figure 1 [file mmc6.zip › SECchrom_O00468_AGRIN_HUMAN_AGRN_AGRIN.pdf]

O00469 | PLOD2\_HUMAN | PLOD2

Monomer MW [kDa]: 84.686 Monomer expected elution fraction: 42

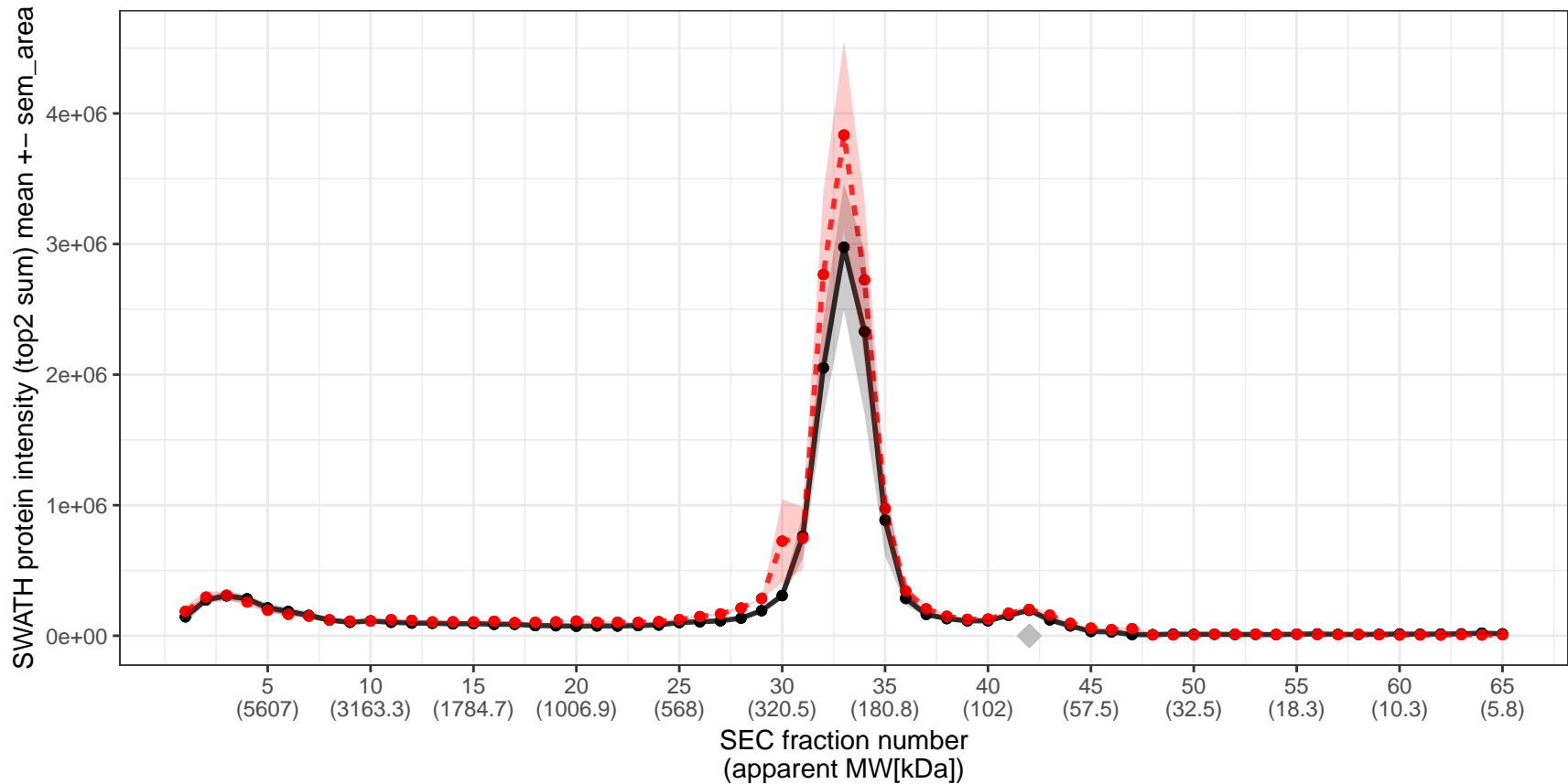

Supplement: Data S1. SEC-SWATH-MS Protein Chromatograms, Related to Figure 1 [file mmc6.zip › SECchrom_O00469_PLOD2_HUMAN_PLOD2.pdf]

O00470 | MEIS1\_HUMAN | MEIS1

Monomer MW [kDa]: 43.016 Monomer expected elution fraction: 48

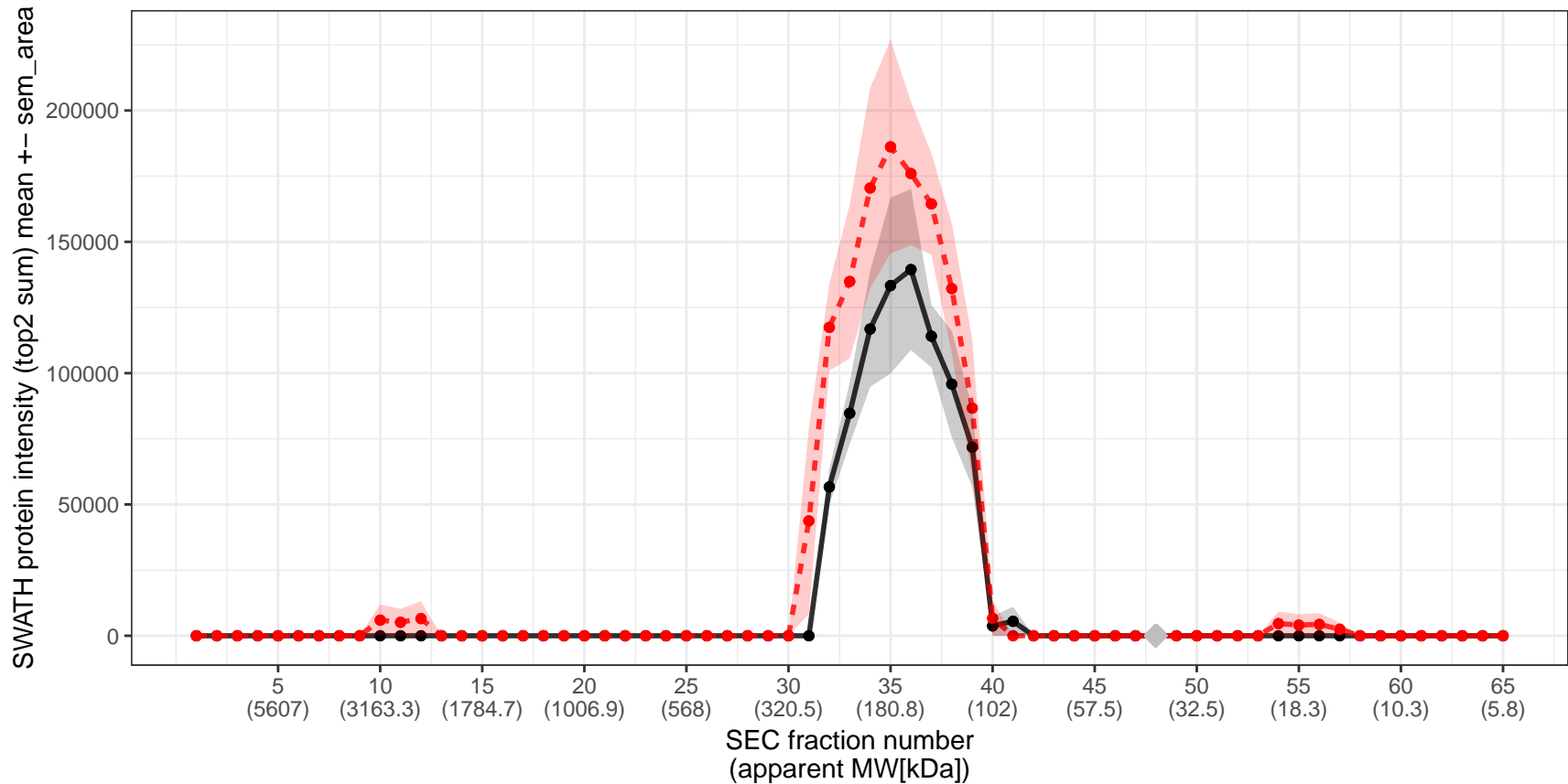

Supplement: Data S1. SEC-SWATH-MS Protein Chromatograms, Related to Figure 1 [file mmc6.zip › SECchrom_O00470_MEIS1_HUMAN_MEIS1.pdf]

O00505 | IMA4\_HUMAN | KPNA3 QIP2

Monomer MW [kDa]: 57.811 Monomer expected elution fraction: 45

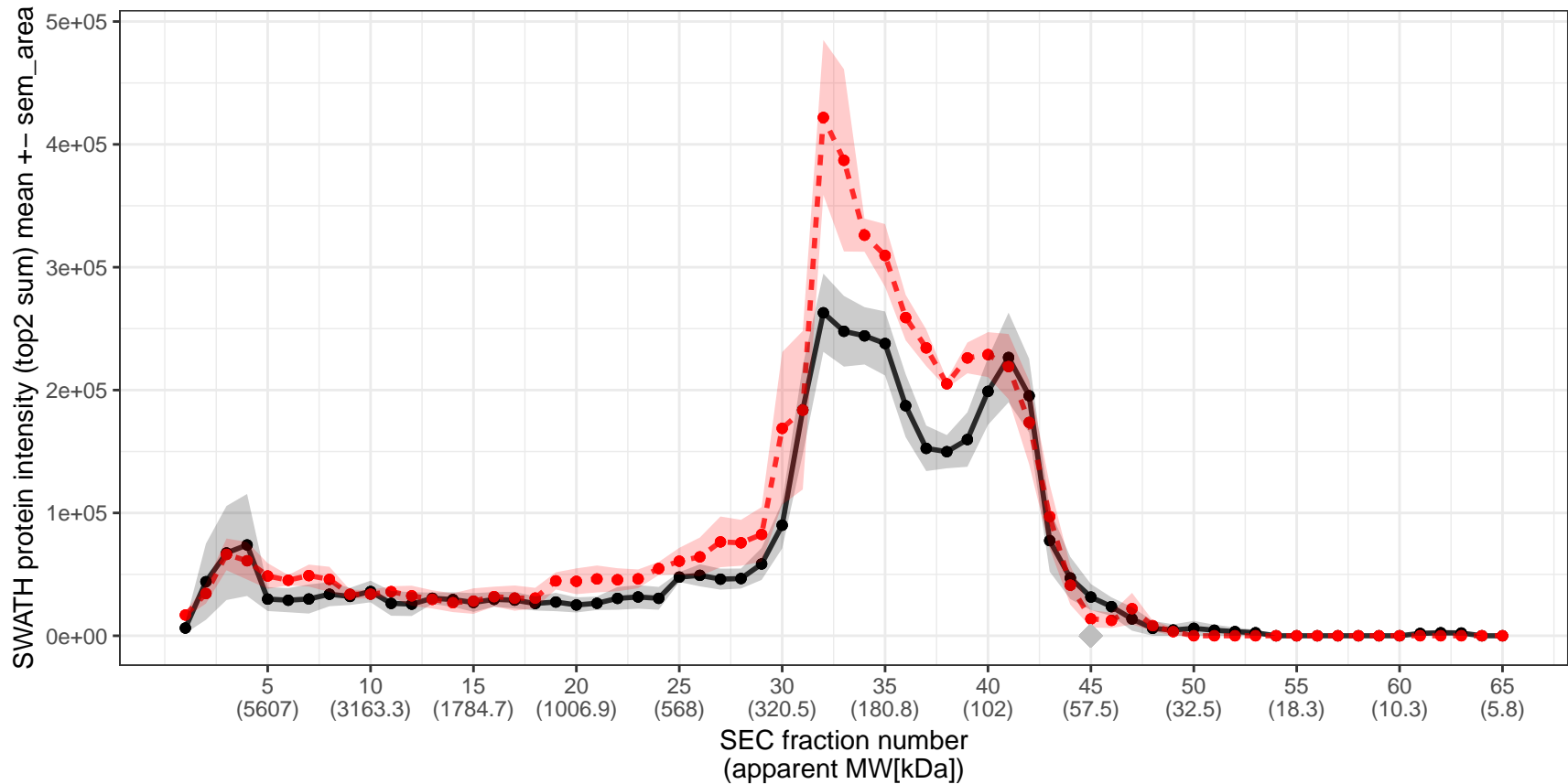

Supplement: Data S1. SEC-SWATH-MS Protein Chromatograms, Related to Figure 1 [file mmc6.zip › SECchrom_O00505_IMA4_HUMAN_KPNA3_QIP2.pdf]

O00560 | SDCB1\_HUMAN | SDCBP MDA9 SYCL

Monomer MW [kDa]: 32.444 Monomer expected elution fraction: 50

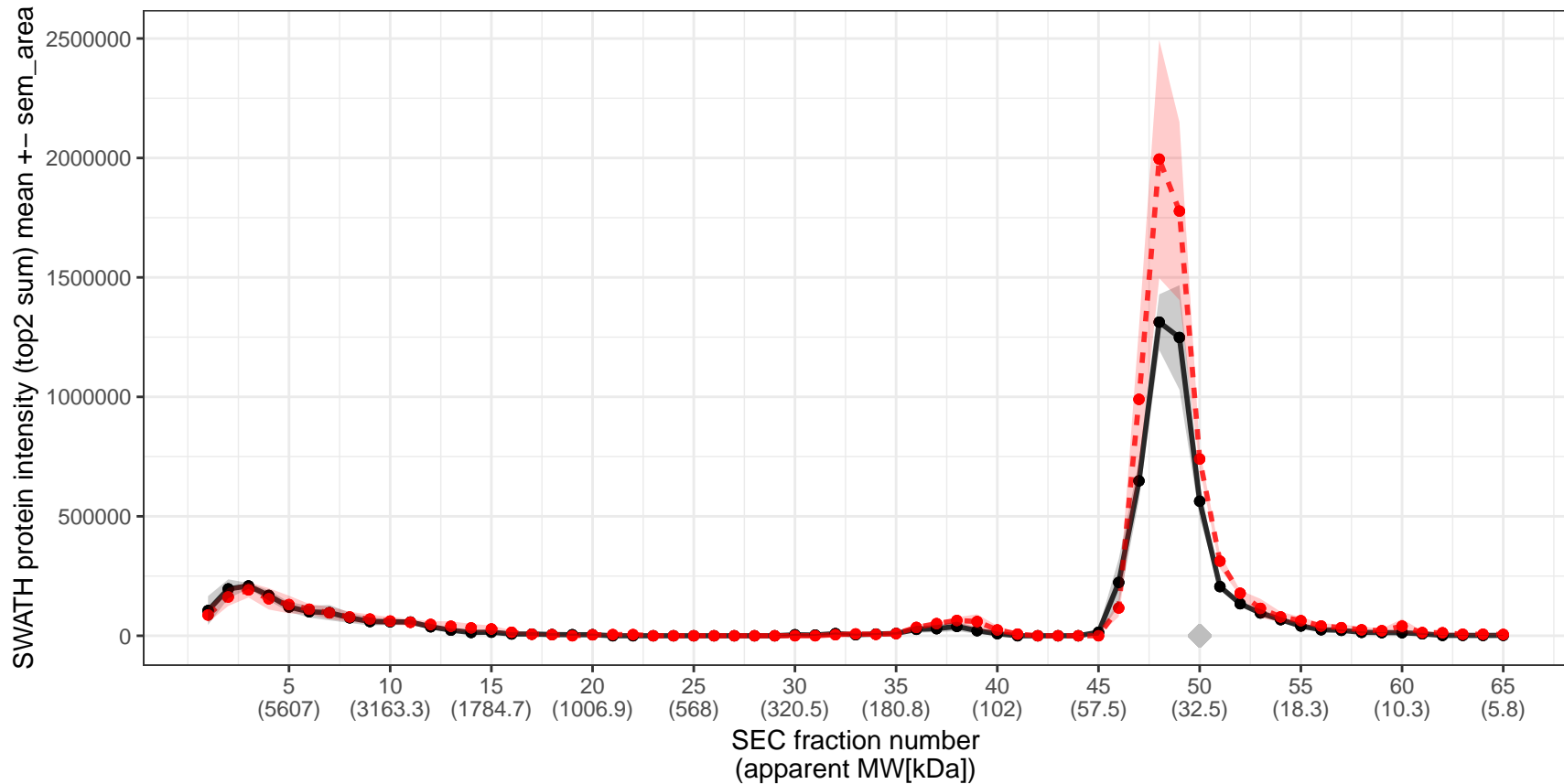

Supplement: Data S1. SEC-SWATH-MS Protein Chromatograms, Related to Figure 1 [file mmc6.zip › SECchrom_O00560_SDCB1_HUMAN_SDCBP_MDA9_SYCL.pdf]

O00567 | NOP56\_HUMAN | NOP56 NOL5A

Monomer MW [kDa]: 66.05 Monomer expected elution fraction: 44

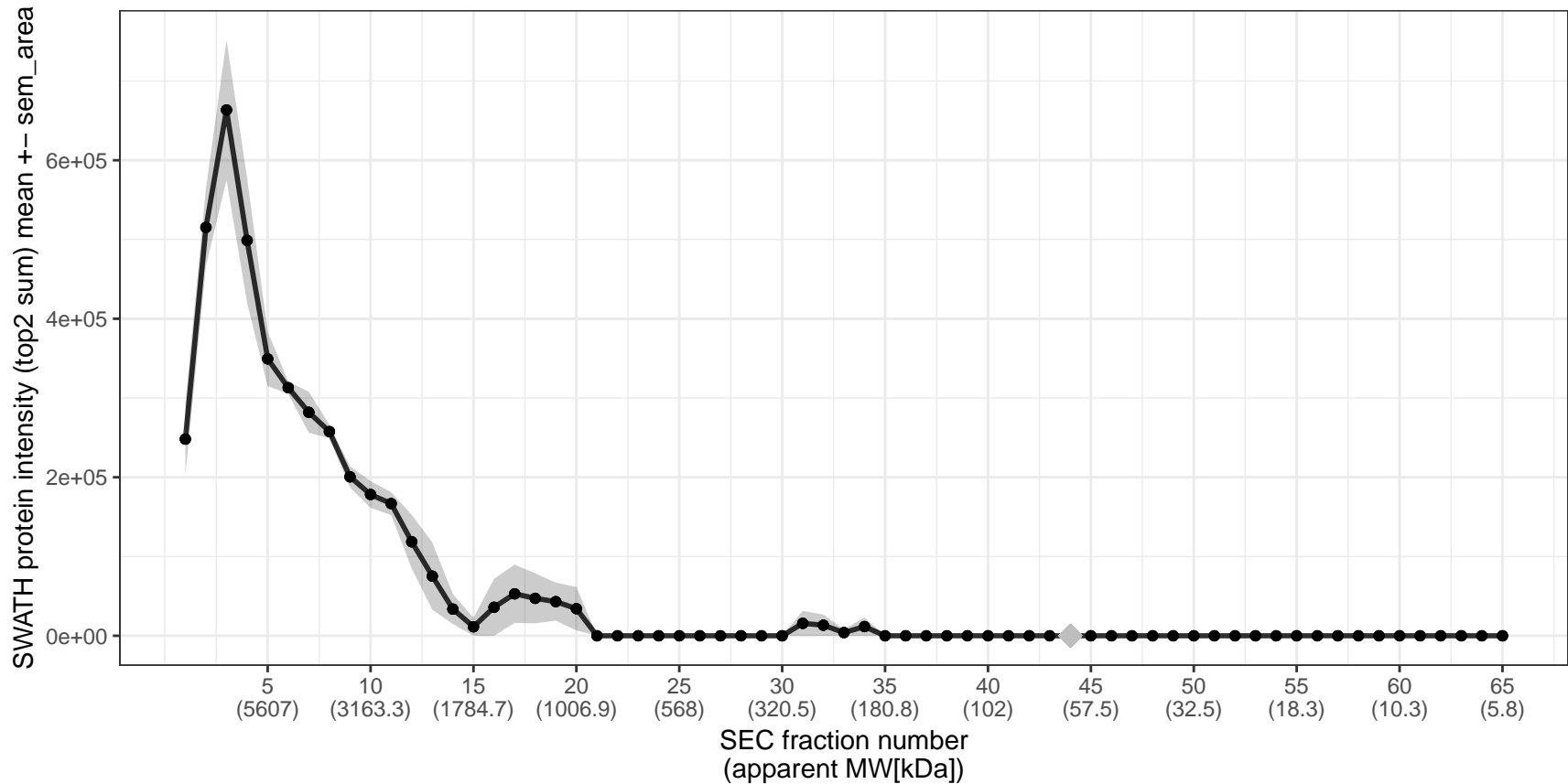

Supplement: Data S1. SEC-SWATH-MS Protein Chromatograms, Related to Figure 1 [file mmc6.zip › SECchrom_O00567_NOP56_HUMAN_NOP56_NOL5A.pdf]

O00584 | RNT2\_HUMAN | RNASET2 RNASE6PL

Monomer MW [kDa]: 29.481 Monomer expected elution fraction: 51

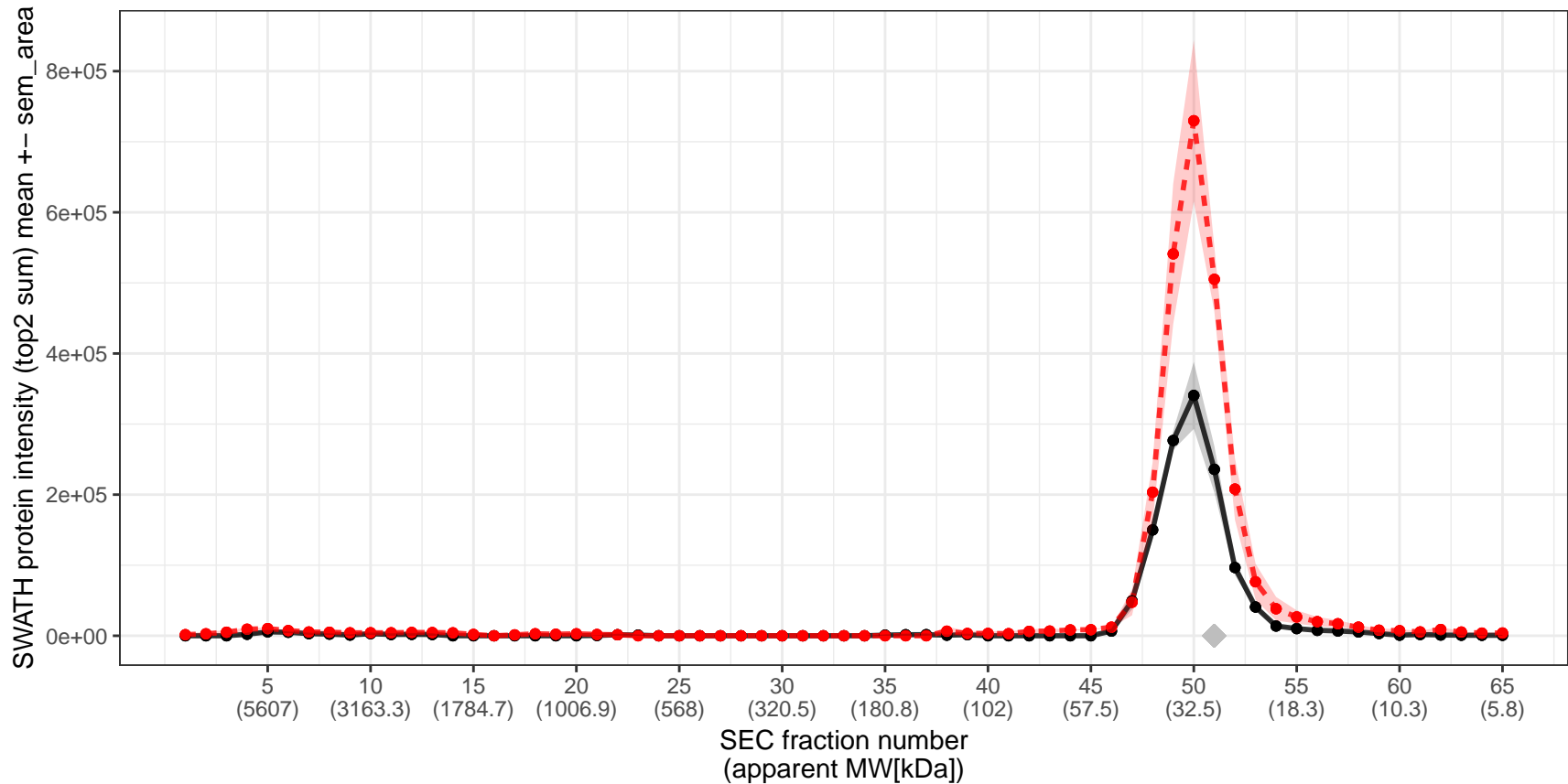

Supplement: Data S1. SEC-SWATH-MS Protein Chromatograms, Related to Figure 1 [file mmc6.zip › SECchrom_O00584_RNT2_HUMAN_RNASET2_RNASE6PL.pdf]

O00592 | PODXL\_HUMAN | PODXL PCLP PCLP1

Monomer MW [kDa]: 58.635 Monomer expected elution fraction: 45

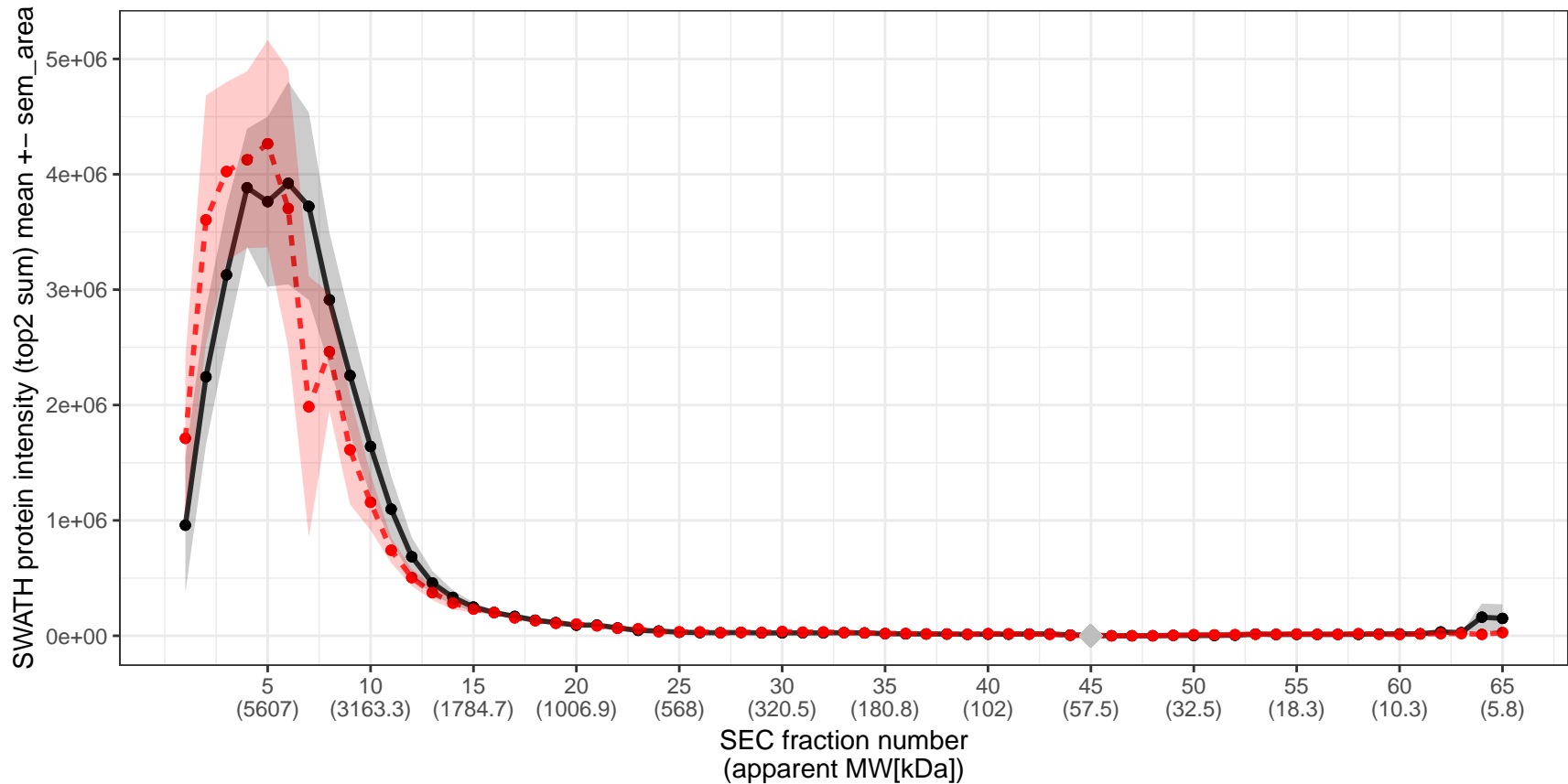

Supplement: Data S1. SEC-SWATH-MS Protein Chromatograms, Related to Figure 1 [file mmc6.zip › SECchrom_O00592_PODXL_HUMAN_PODXL_PCLP_PCLP1.pdf]

O00625 | PIR\_HUMAN | PIR

Monomer MW [kDa]: 32.113 Monomer expected elution fraction: 50

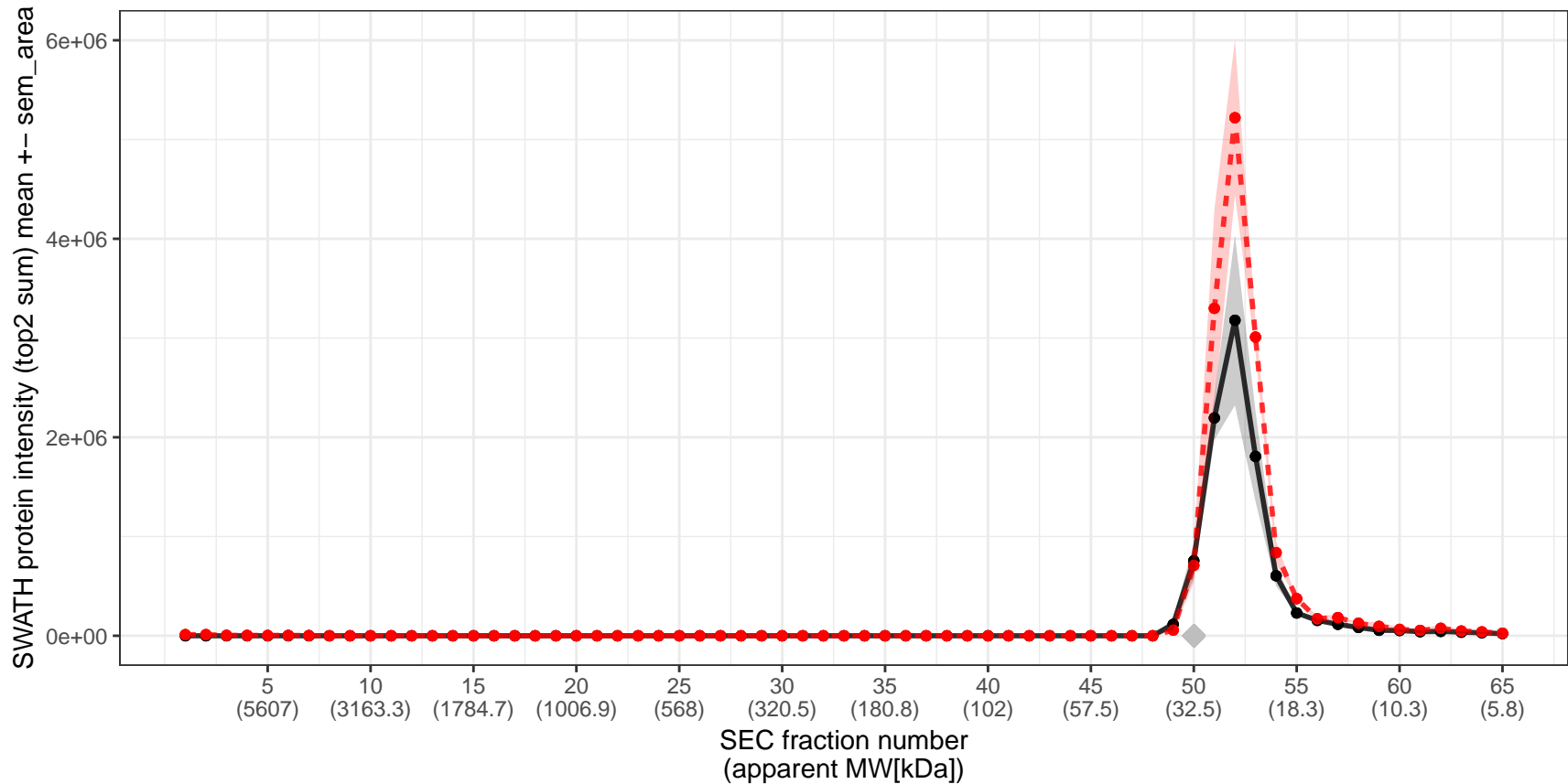

Supplement: Data S1. SEC-SWATH-MS Protein Chromatograms, Related to Figure 1 [file mmc6.zip › SECchrom_O00625_PIR_HUMAN_PIR.pdf]

O00629 | IMA3\_HUMAN | KPNA4 QIP1

Monomer MW [kDa]: 57.887 Monomer expected elution fraction: 45

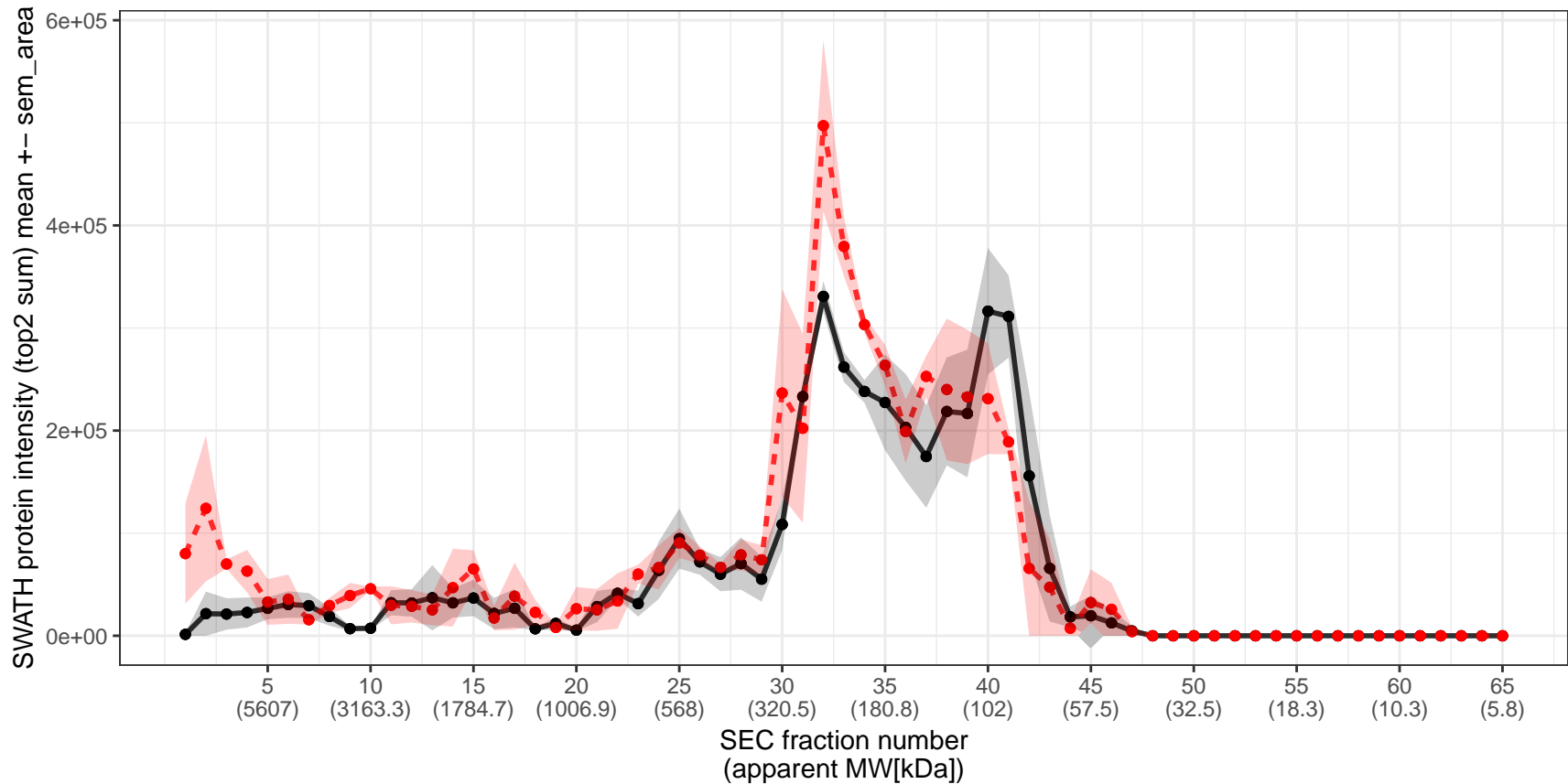

Supplement: Data S1. SEC-SWATH-MS Protein Chromatograms, Related to Figure 1 [file mmc6.zip › SECchrom_O00629_IMA3_HUMAN_KPNA4_QIP1.pdf]

O00635 | TRI38\_HUMAN | TRIM38 RNF15 RORET

Monomer MW [kDa]: 53.416 Monomer expected elution fraction: 46

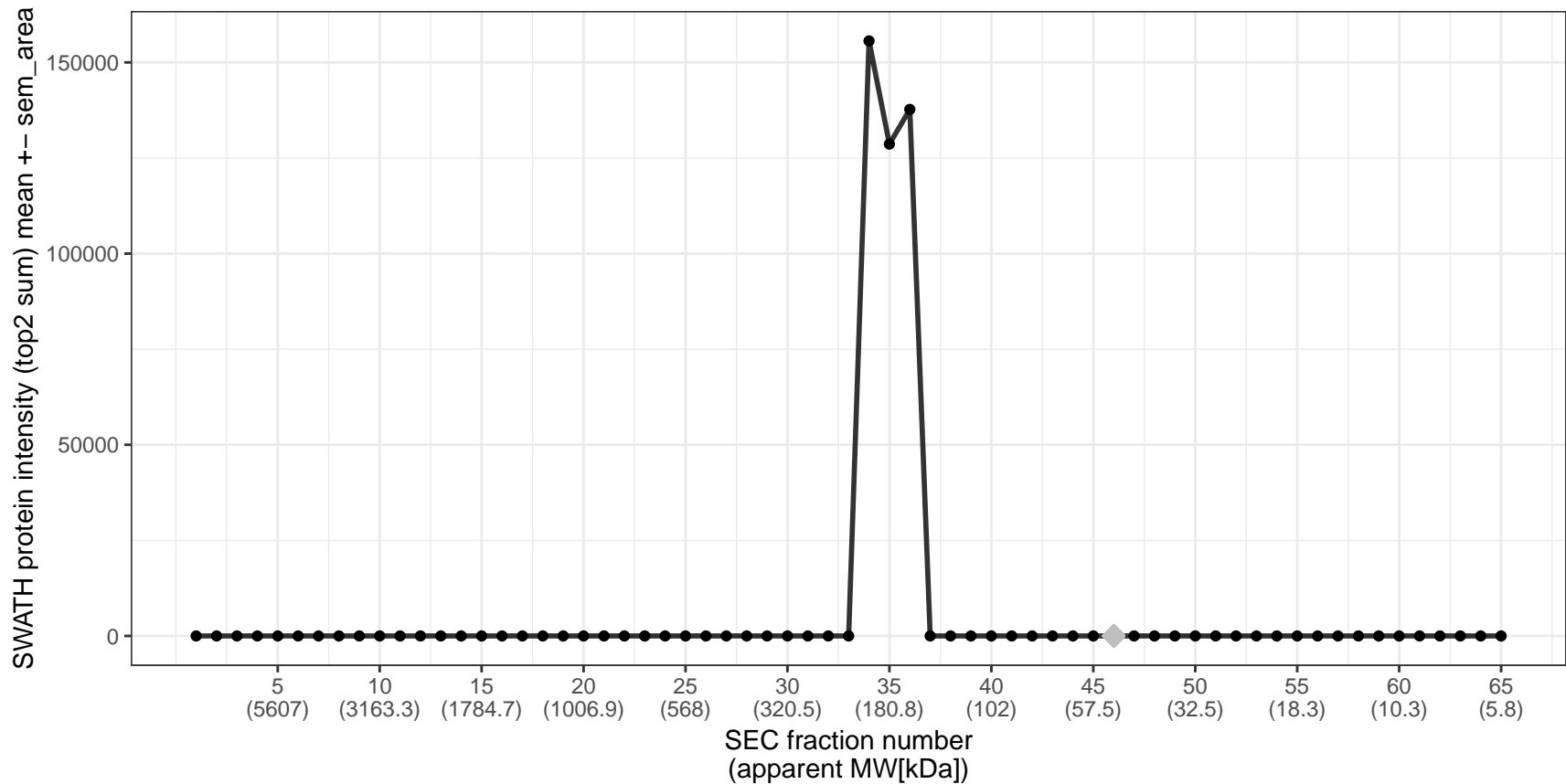

Supplement: Data S1. SEC-SWATH-MS Protein Chromatograms, Related to Figure 1 [file mmc6.zip › SECchrom_O00635_TRI38_HUMAN_TRIM38_RNF15_RORET.pdf]

O00712 | NFIB\_HUMAN | NFIB

Monomer MW [kDa]: 47.442 Monomer expected elution fraction: 47

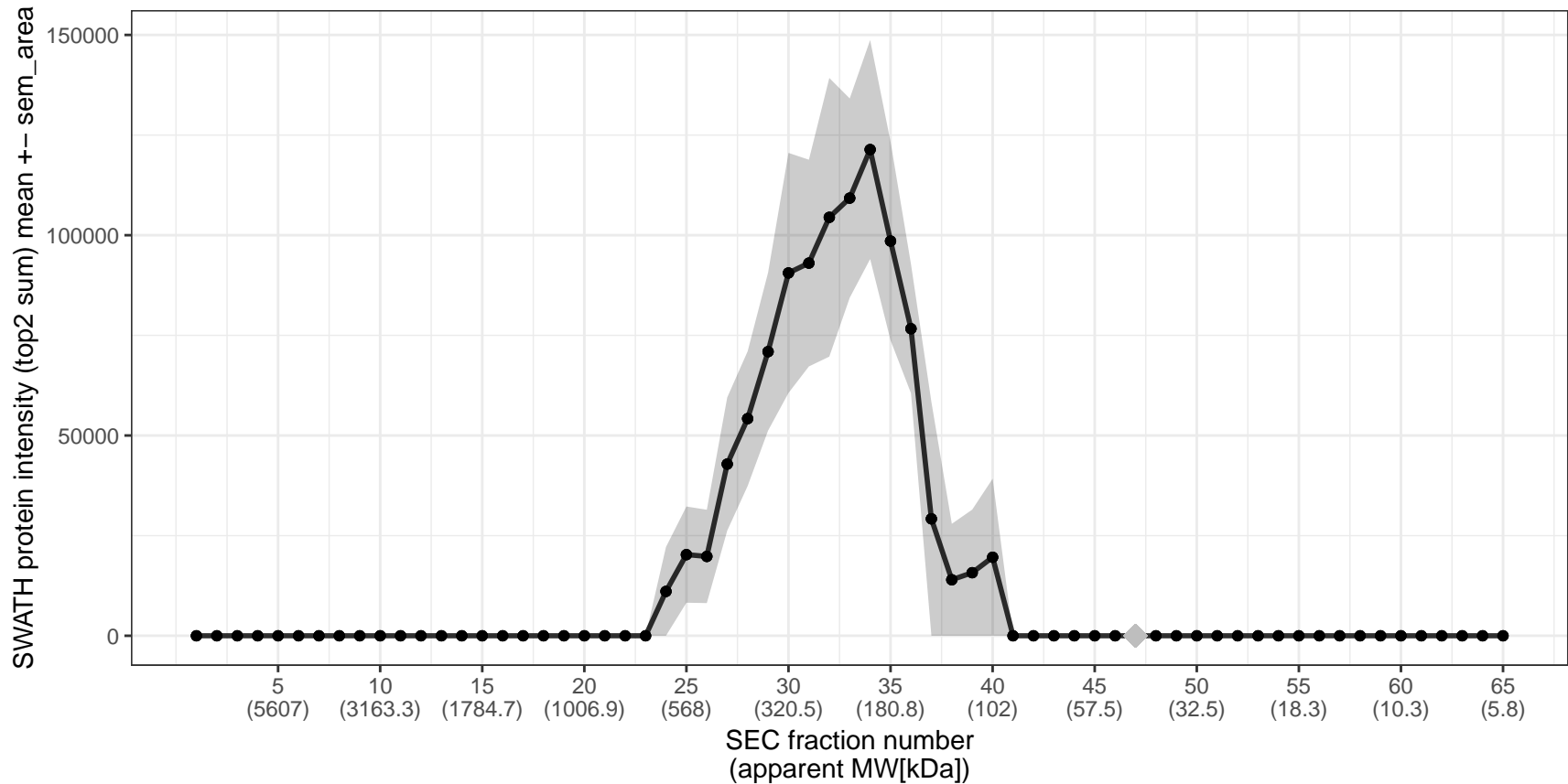

Supplement: Data S1. SEC-SWATH-MS Protein Chromatograms, Related to Figure 1 [file mmc6.zip › SECchrom_O00712_NFIB_HUMAN_NFIB.pdf]

O00743 | PPP6\_HUMAN | PPP6C PPP6

Monomer MW [kDa]: 35.144 Monomer expected elution fraction: 49

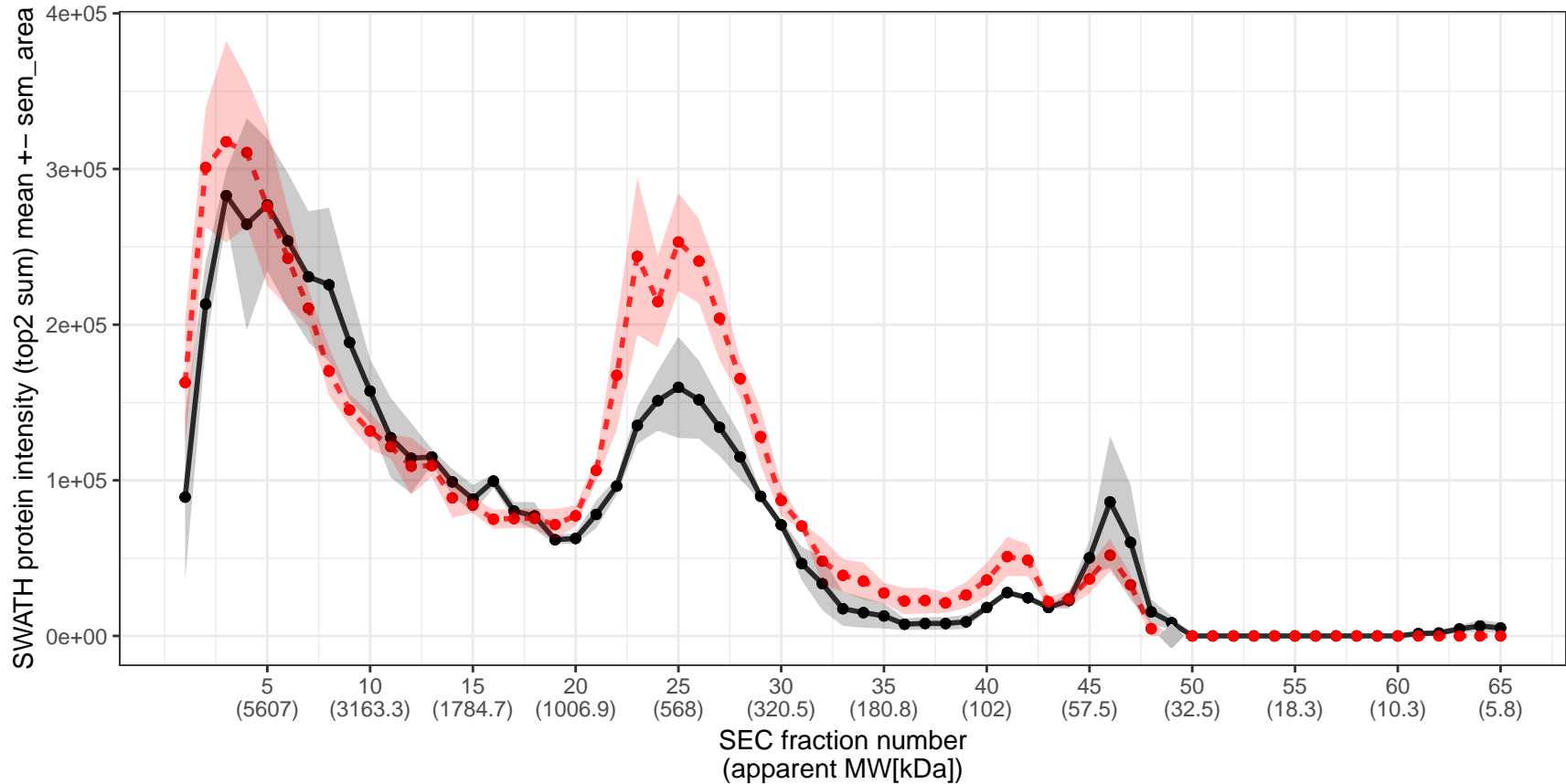

Supplement: Data S1. SEC-SWATH-MS Protein Chromatograms, Related to Figure 1 [file mmc6.zip › SECchrom_O00743_PPP6_HUMAN_PPP6C_PPP6.pdf]

O00750 | P3C2B\_HUMAN | PIK3C2B

Monomer MW [kDa]: 184.768 Monomer expected elution fraction: 35

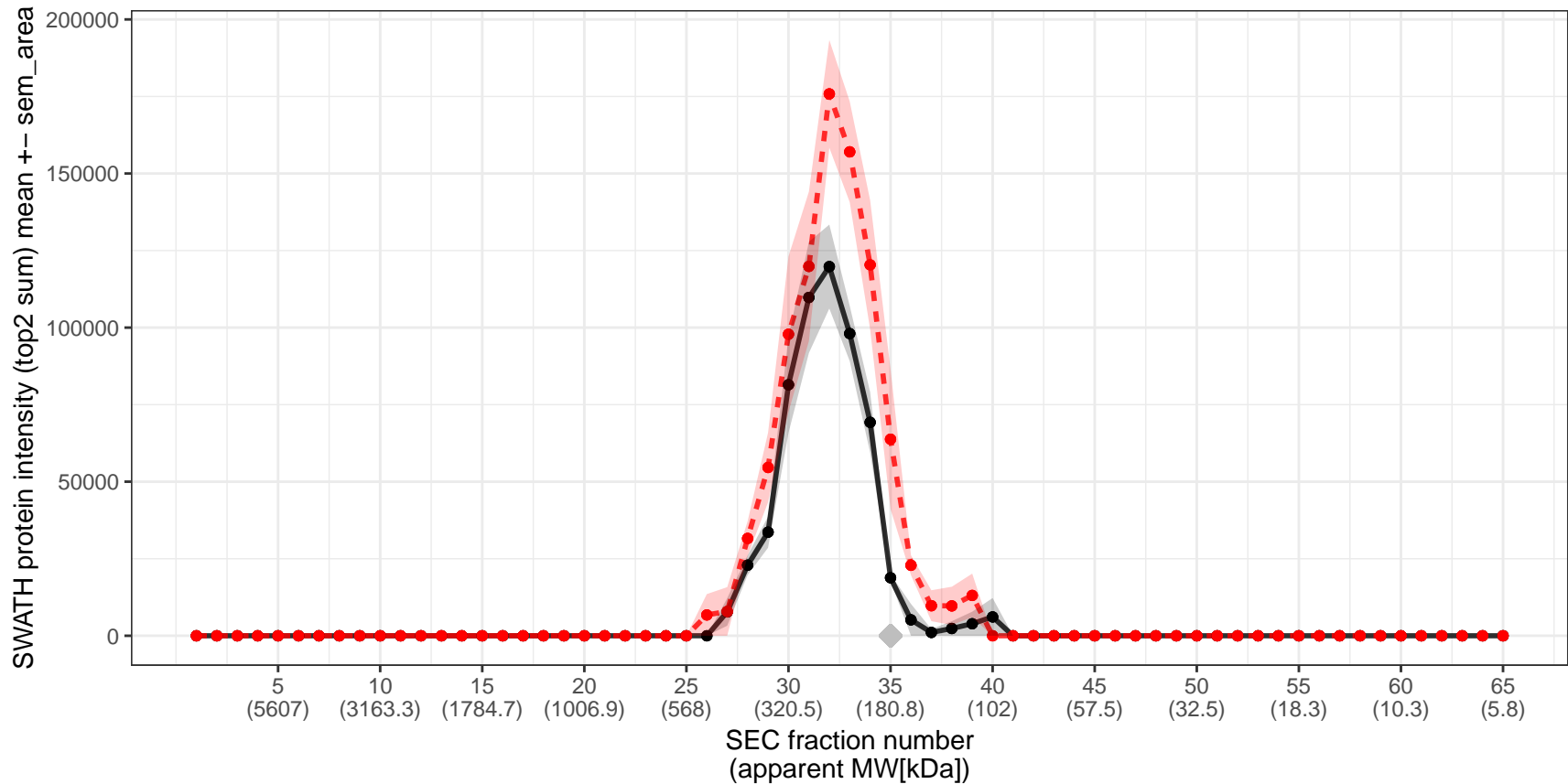

Supplement: Data S1. SEC-SWATH-MS Protein Chromatograms, Related to Figure 1 [file mmc6.zip › SECchrom_O00750_P3C2B_HUMAN_PIK3C2B.pdf]

O00754 | MA2B1\_HUMAN | MAN2B1 LAMAN MANB

Monomer MW [kDa]: 113.744 Monomer expected elution fraction: 39

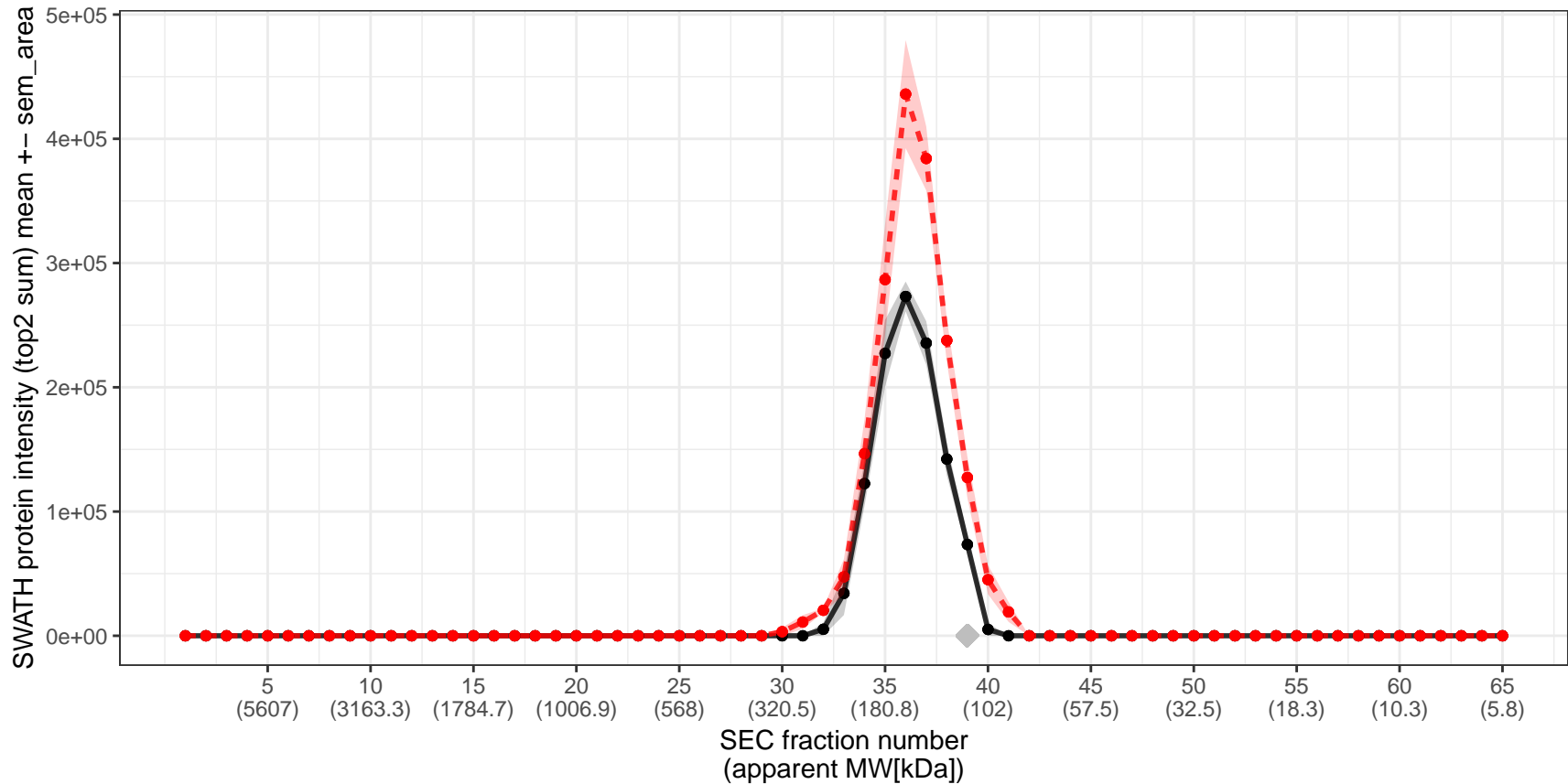

Supplement: Data S1. SEC-SWATH-MS Protein Chromatograms, Related to Figure 1 [file mmc6.zip › SECchrom_O00754_MA2B1_HUMAN_MAN2B1_LAMAN_MANB.pdf]

O00764 | PDXK\_HUMAN | PDXK C21orf124 C21orf97 PKH PNK PRED79

Monomer MW [kDa]: 35.102 Monomer expected elution fraction: 49

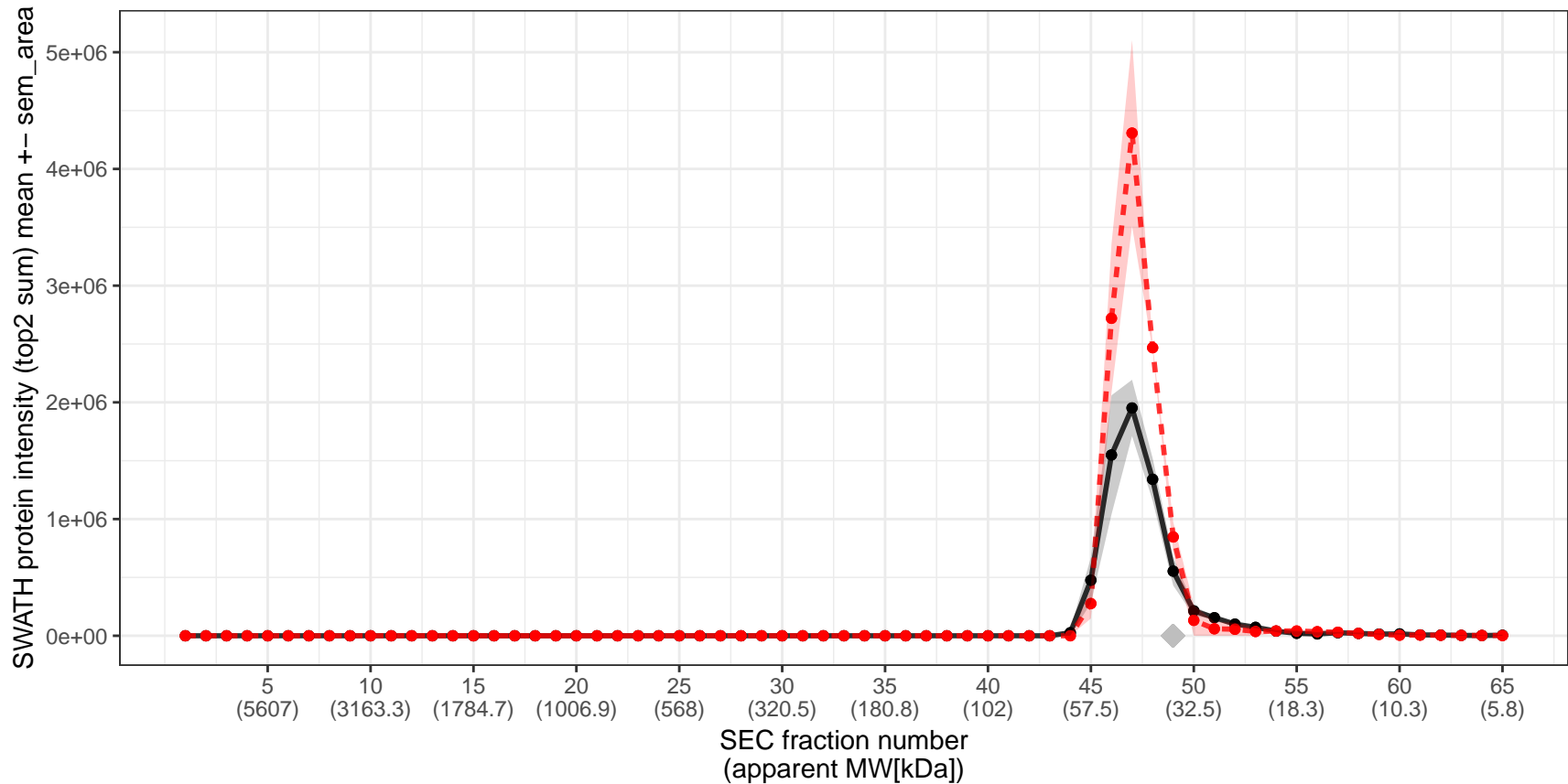

Supplement: Data S1. SEC-SWATH-MS Protein Chromatograms, Related to Figure 1 [file mmc6.zip › SECchrom_O00764_PDXK_HUMAN_PDXK_C21orf124_C21orf97_PKH_PNK_.pdf]

O14524 | NEMP1\_HUMAN | NEMP1 KIAA0286 TMEM194 TMEM194A

Monomer MW [kDa]: 50.64 Monomer expected elution fraction: 46

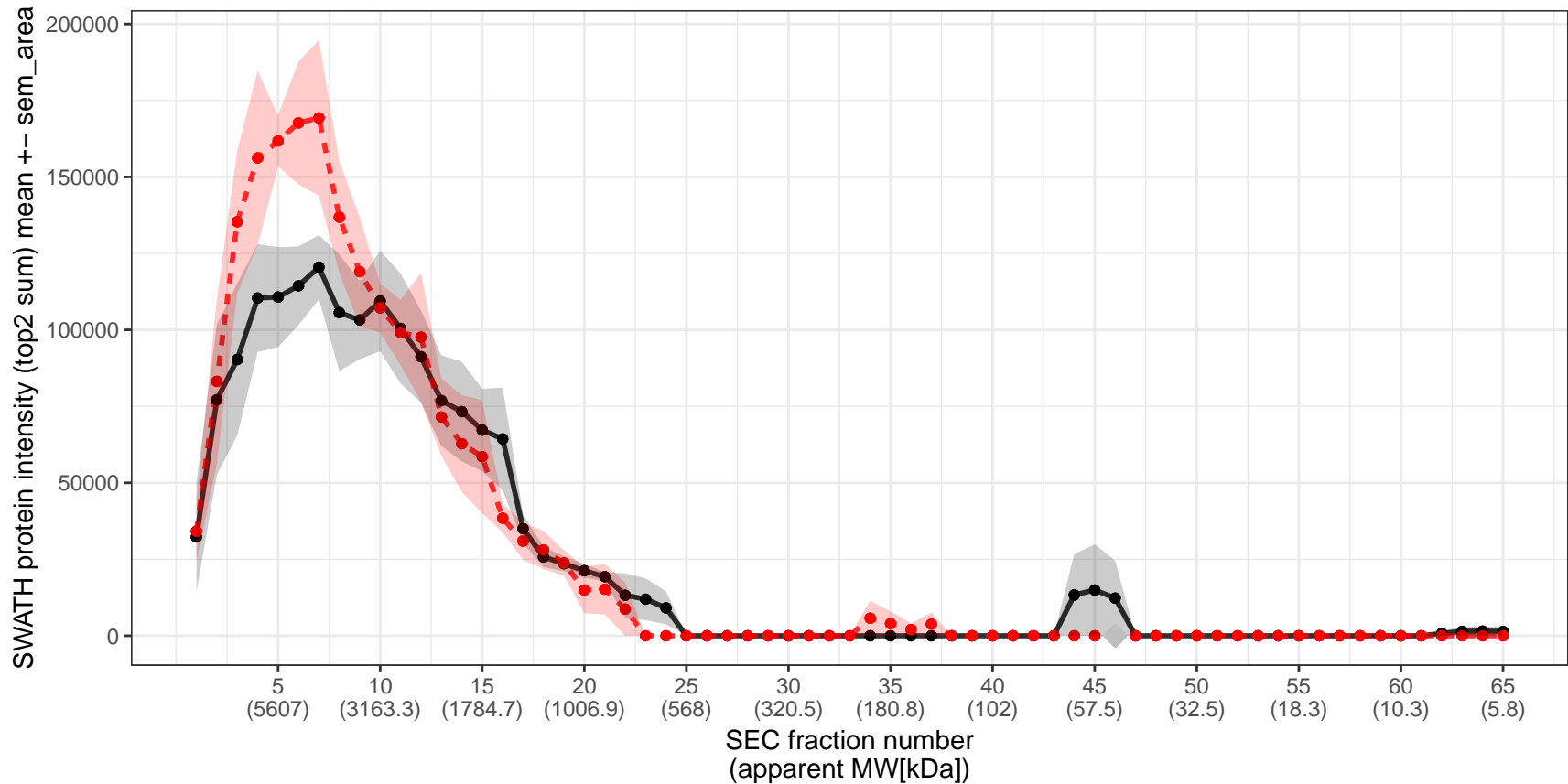

Supplement: Data S1. SEC-SWATH-MS Protein Chromatograms, Related to Figure 1 [file mmc6.zip › SECchrom_O14524_NEMP1_HUMAN_NEMP1_KIAA0286_TMEM194_TMEM194A.pdf]

O14530 | TXND9\_HUMAN | TXNDC9 APACD

Monomer MW [kDa]: 26.534 Monomer expected elution fraction: 52

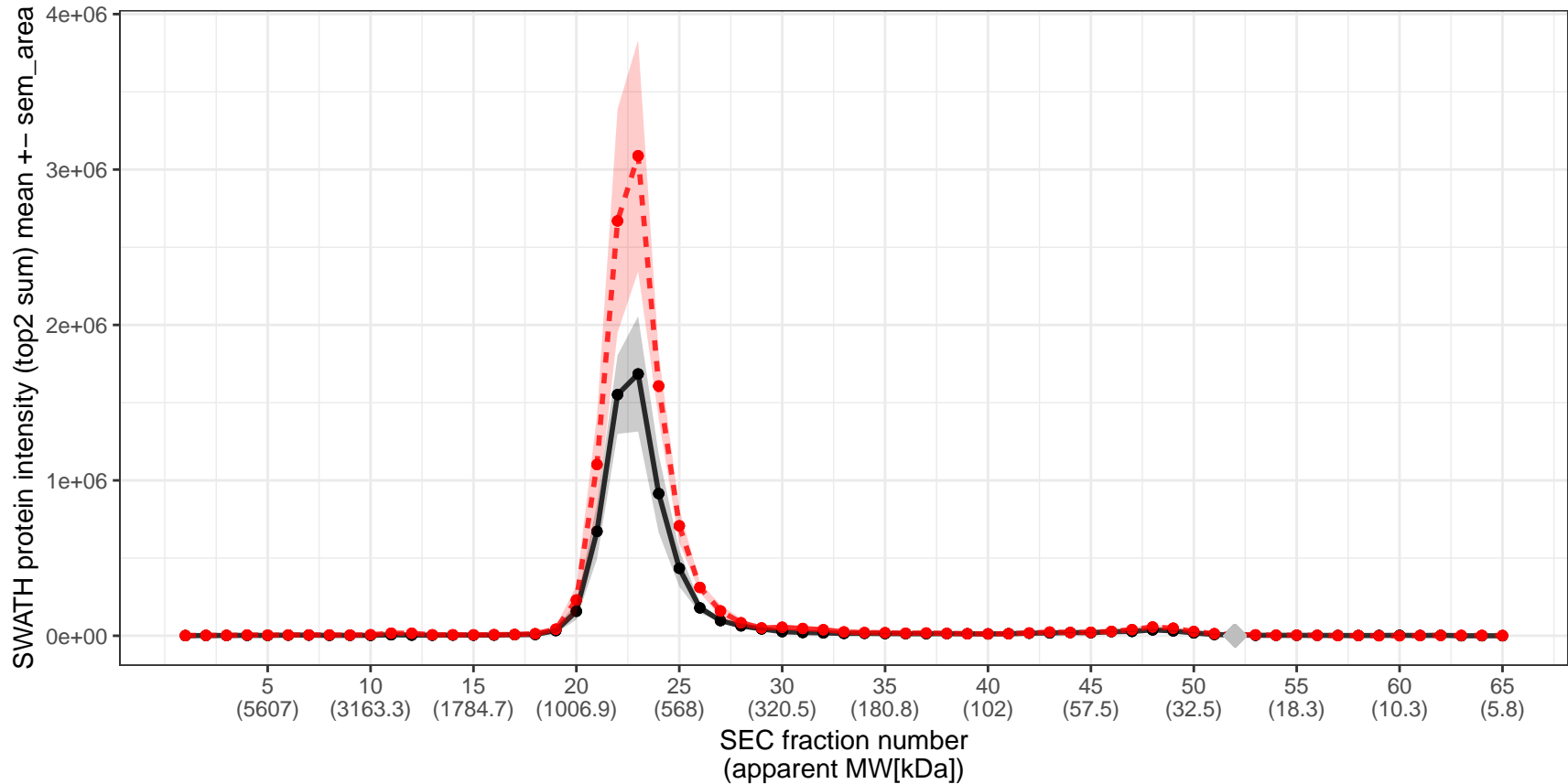

Supplement: Data S1. SEC-SWATH-MS Protein Chromatograms, Related to Figure 1 [file mmc6.zip › SECchrom_O14530_TXND9_HUMAN_TXNDC9_APACD.pdf]

O14618 | CCS\_HUMAN | CCS

Monomer MW [kDa]: 29.041 Monomer expected elution fraction: 51

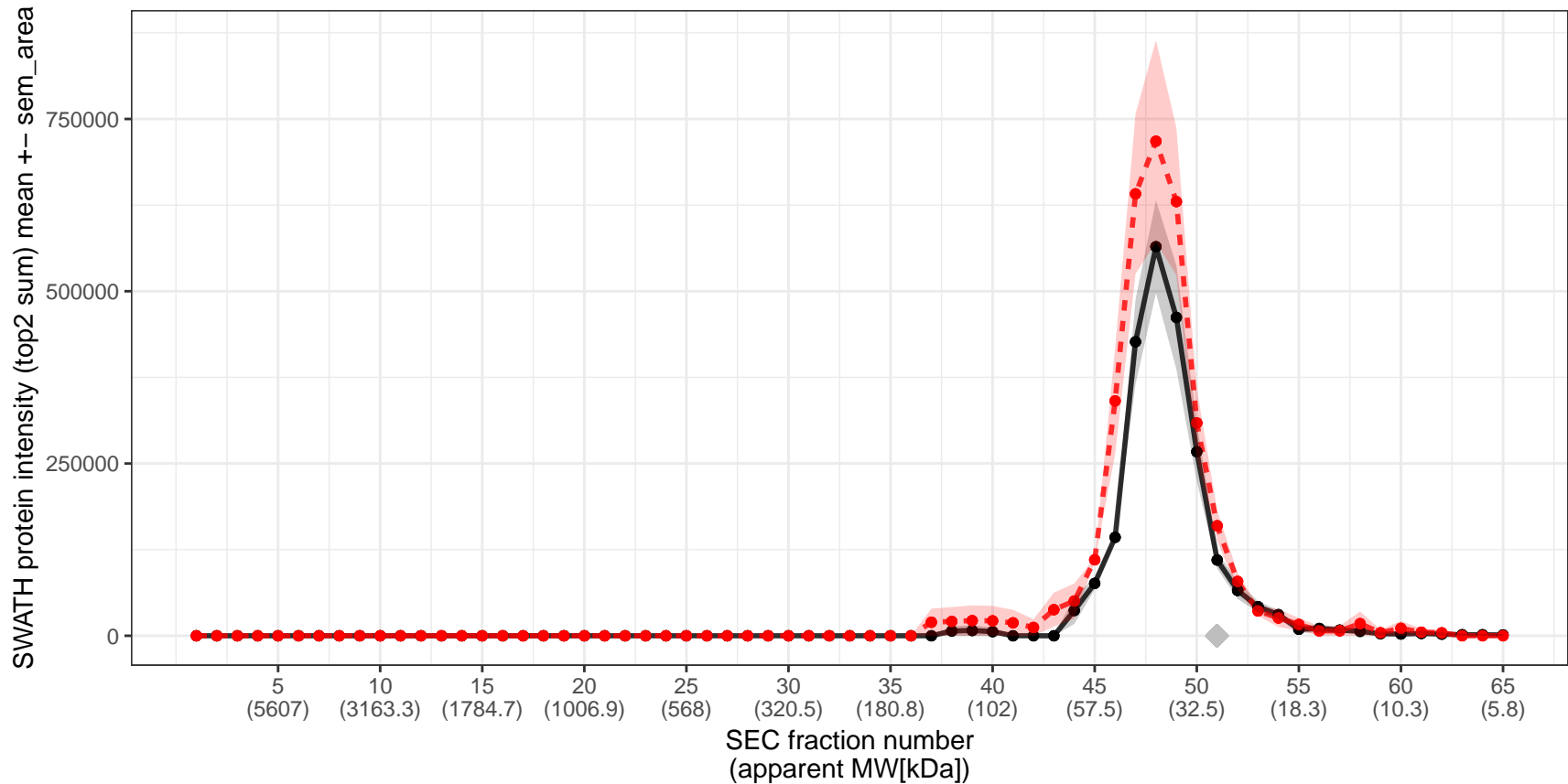

Supplement: Data S1. SEC-SWATH-MS Protein Chromatograms, Related to Figure 1 [file mmc6.zip › SECchrom_O14618_CCS_HUMAN_CCS.pdf]

O14639 | ABLM1\_HUMAN | ABLIM1 ABLIM KIAA0059 LIMAB1  
Monomer MW [kDa]: 87.688 Monomer expected elution fraction: 41

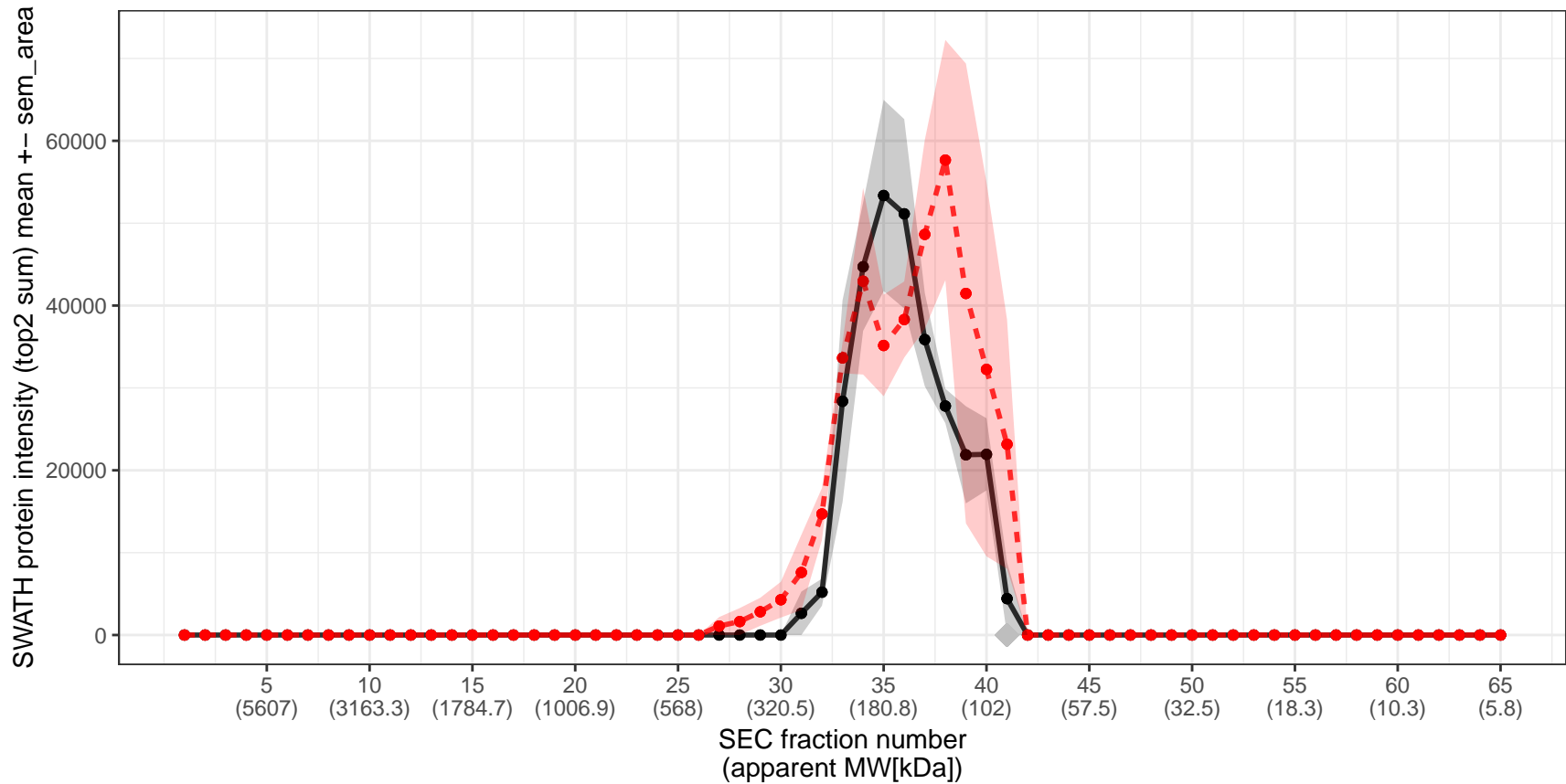

Supplement: Data S1. SEC-SWATH-MS Protein Chromatograms, Related to Figure 1 [file mmc6.zip › SECchrom_O14639_ABLM1_HUMAN_ABLIM1_ABLIM_KIAA0059_LIMAB1.pdf]

O14641 | DVL2\_HUMAN | DVL2

Monomer MW [kDa]: 78.948 Monomer expected elution fraction: 42

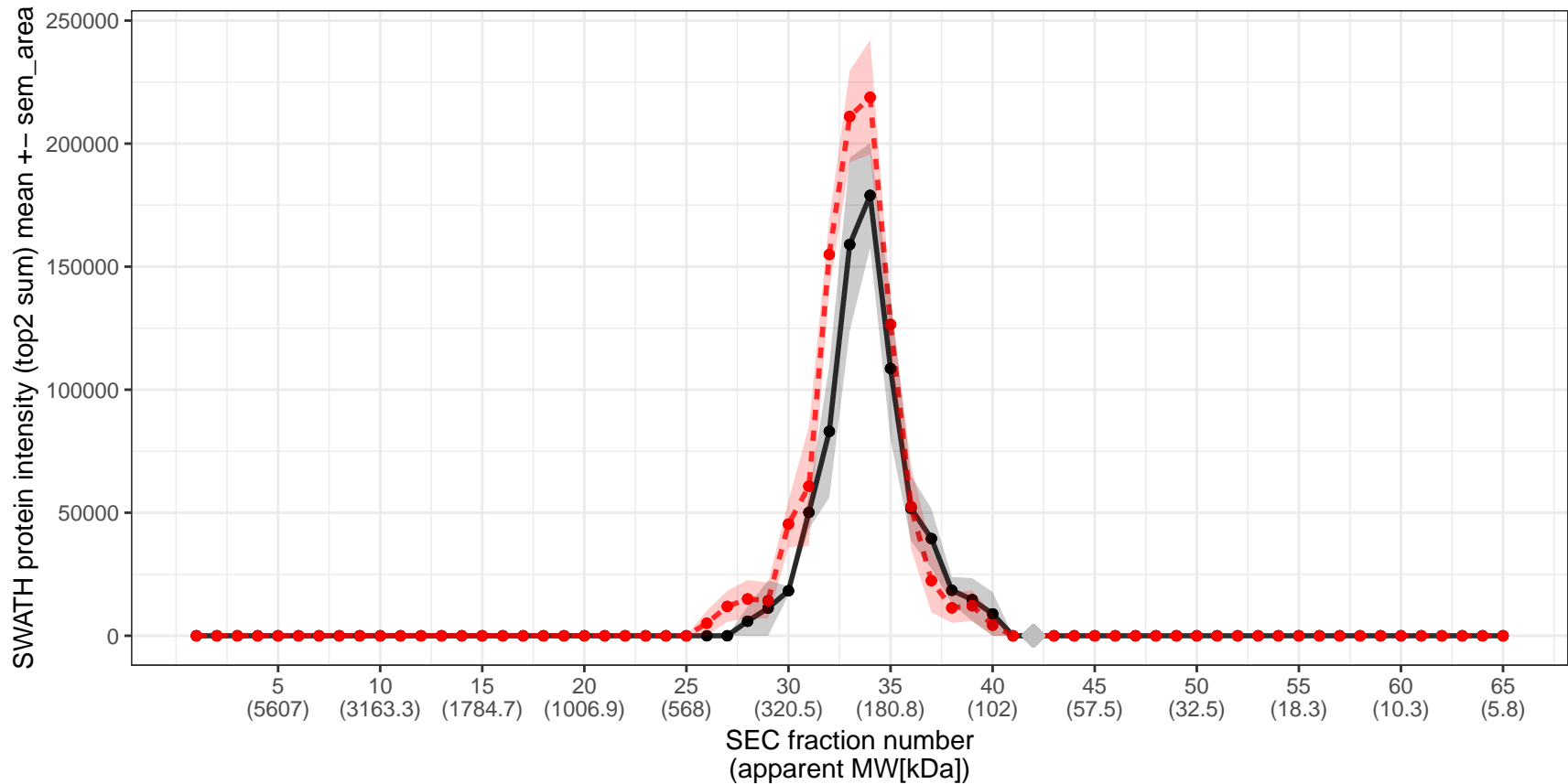

Supplement: Data S1. SEC-SWATH-MS Protein Chromatograms, Related to Figure 1 [file mmc6.zip › SECchrom_O14641_DVL2_HUMAN_DVL2.pdf]

O14656 | TOR1A\_HUMAN | TOR1A DQ2 DYT1 TA TORA  
Monomer MW [kDa]: 37.809 Monomer expected elution fraction: 49

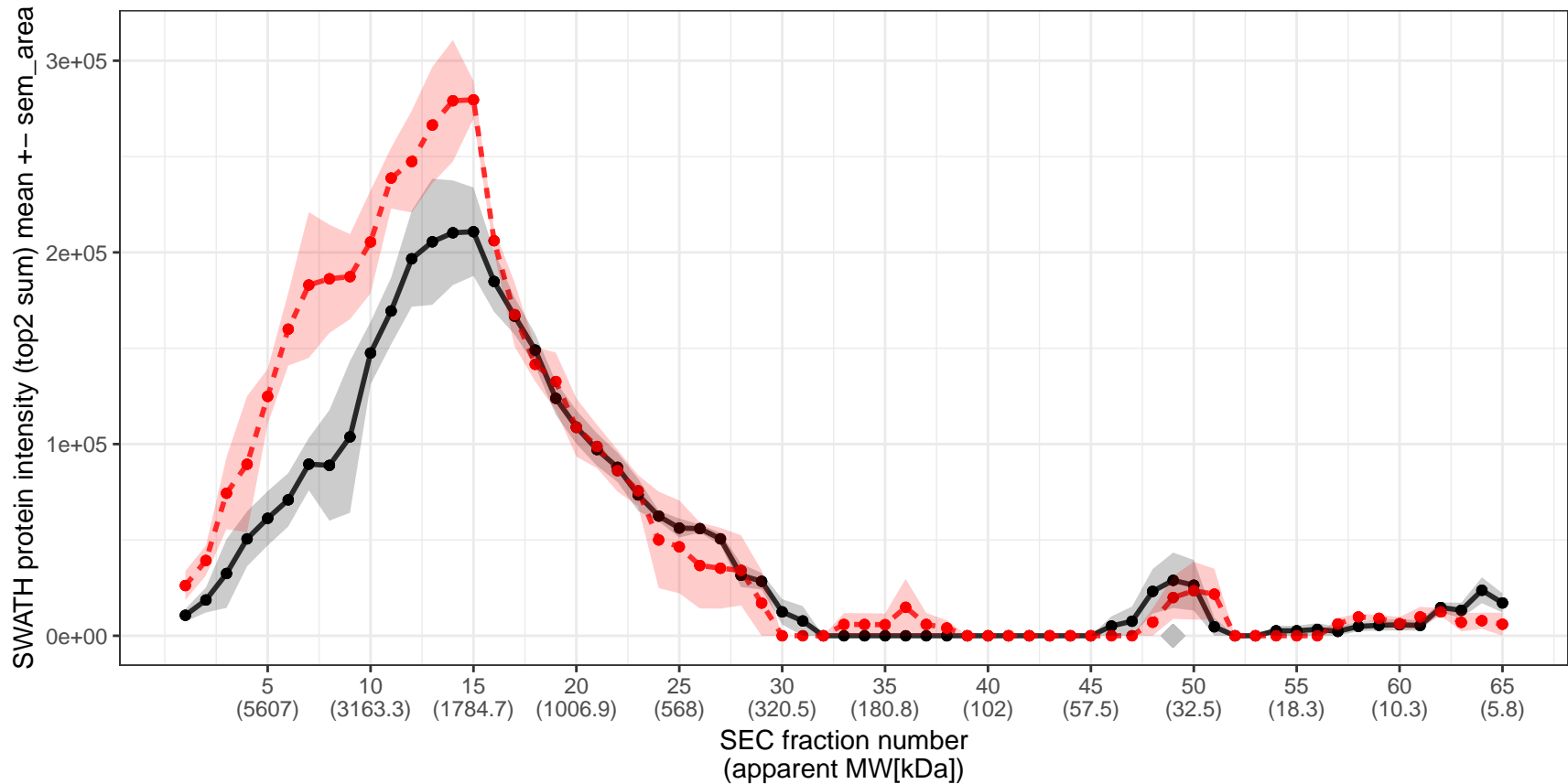

Supplement: Data S1. SEC-SWATH-MS Protein Chromatograms, Related to Figure 1 [file mmc6.zip › SECchrom_O14656_TOR1A_HUMAN_TOR1A_DQ2_DYT1_TA_TORA.pdf]

O14672 | ADA10\_HUMAN | ADAM10 KUZ MADM

Monomer MW [kDa]: 84.142 Monomer expected elution fraction: 42

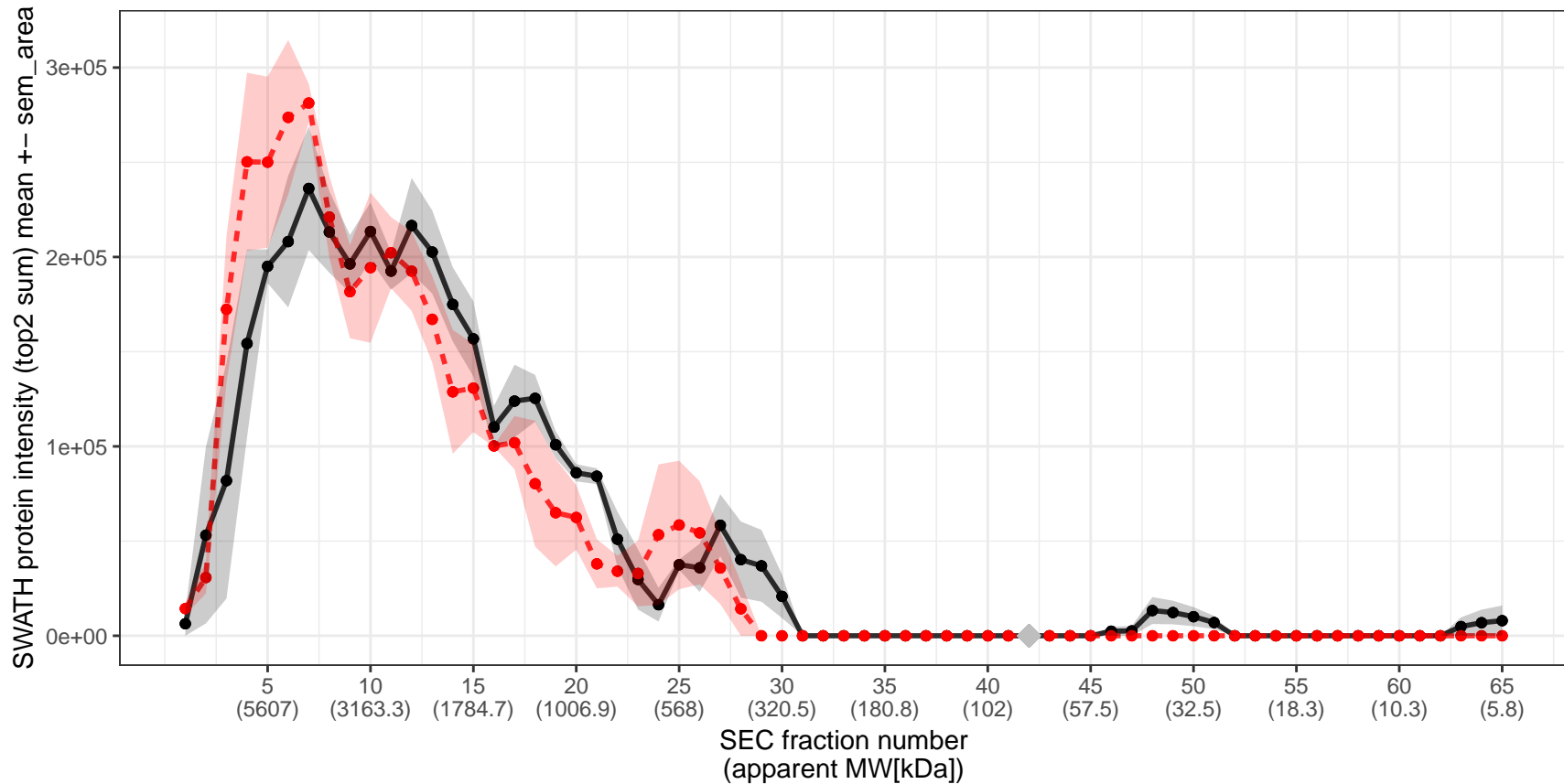

Supplement: Data S1. SEC-SWATH-MS Protein Chromatograms, Related to Figure 1 [file mmc6.zip › SECchrom_O14672_ADA10_HUMAN_ADAM10_KUZ_MADM.pdf]

O14727 | APAF\_HUMAN | APAF1 KIAA0413

Monomer MW [kDa]: 141.84 Monomer expected elution fraction: 37

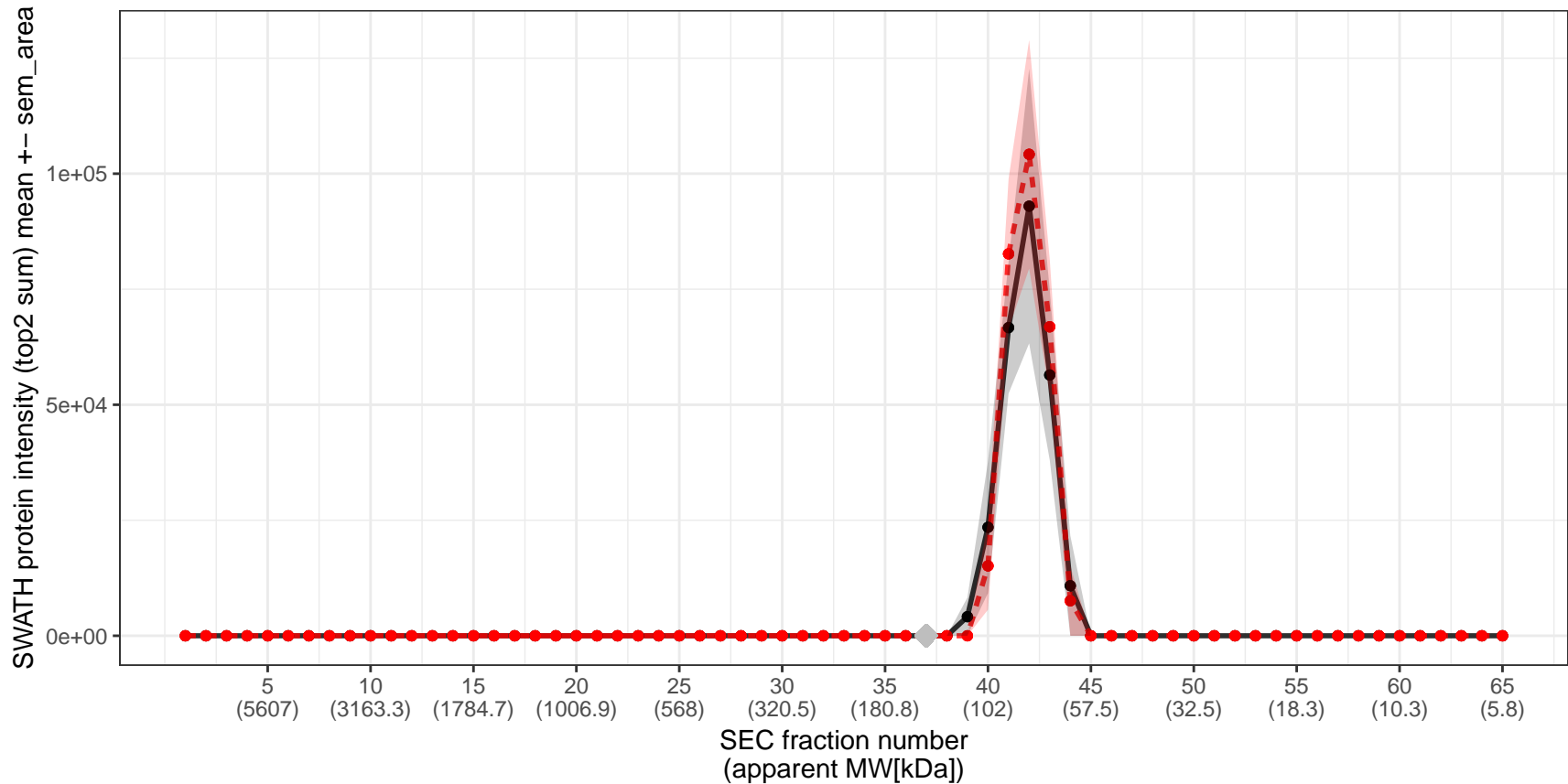

Supplement: Data S1. SEC-SWATH-MS Protein Chromatograms, Related to Figure 1 [file mmc6.zip › SECchrom_O14727_APAF_HUMAN_APAF1_KIAA0413.pdf]

O14732 | IMPA2\_HUMAN | IMPA2 IMP.18P

Monomer MW [kDa]: 31.321 Monomer expected elution fraction: 50

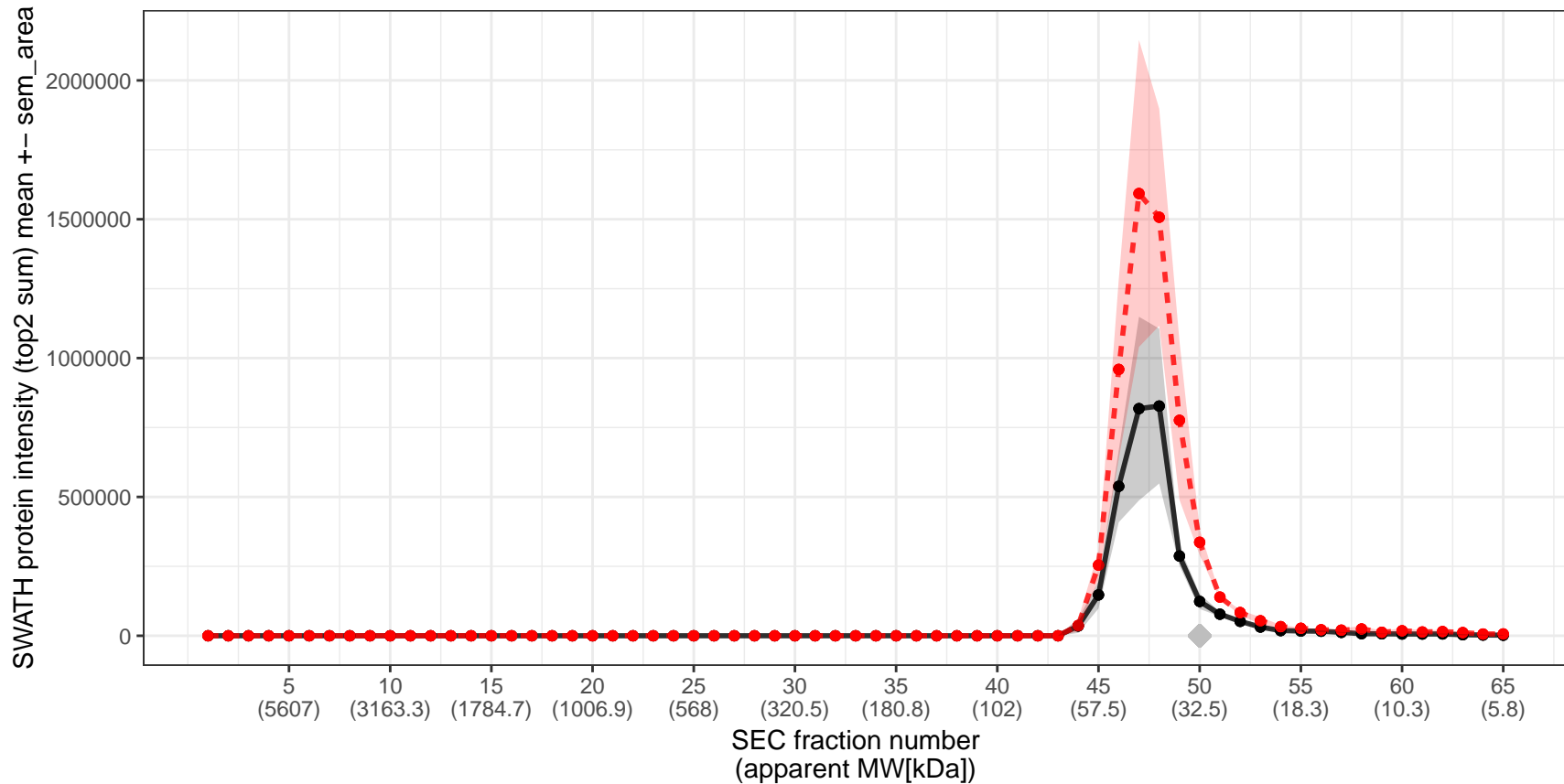

Supplement: Data S1. SEC-SWATH-MS Protein Chromatograms, Related to Figure 1 [file mmc6.zip › SECchrom_O14732_IMPA2_HUMAN_IMPA2_IMP.18P.pdf]

O14737 | PDCD5\_HUMAN | PDCD5 TFAR19

Monomer MW [kDa]: 14.285 Monomer expected elution fraction: 57

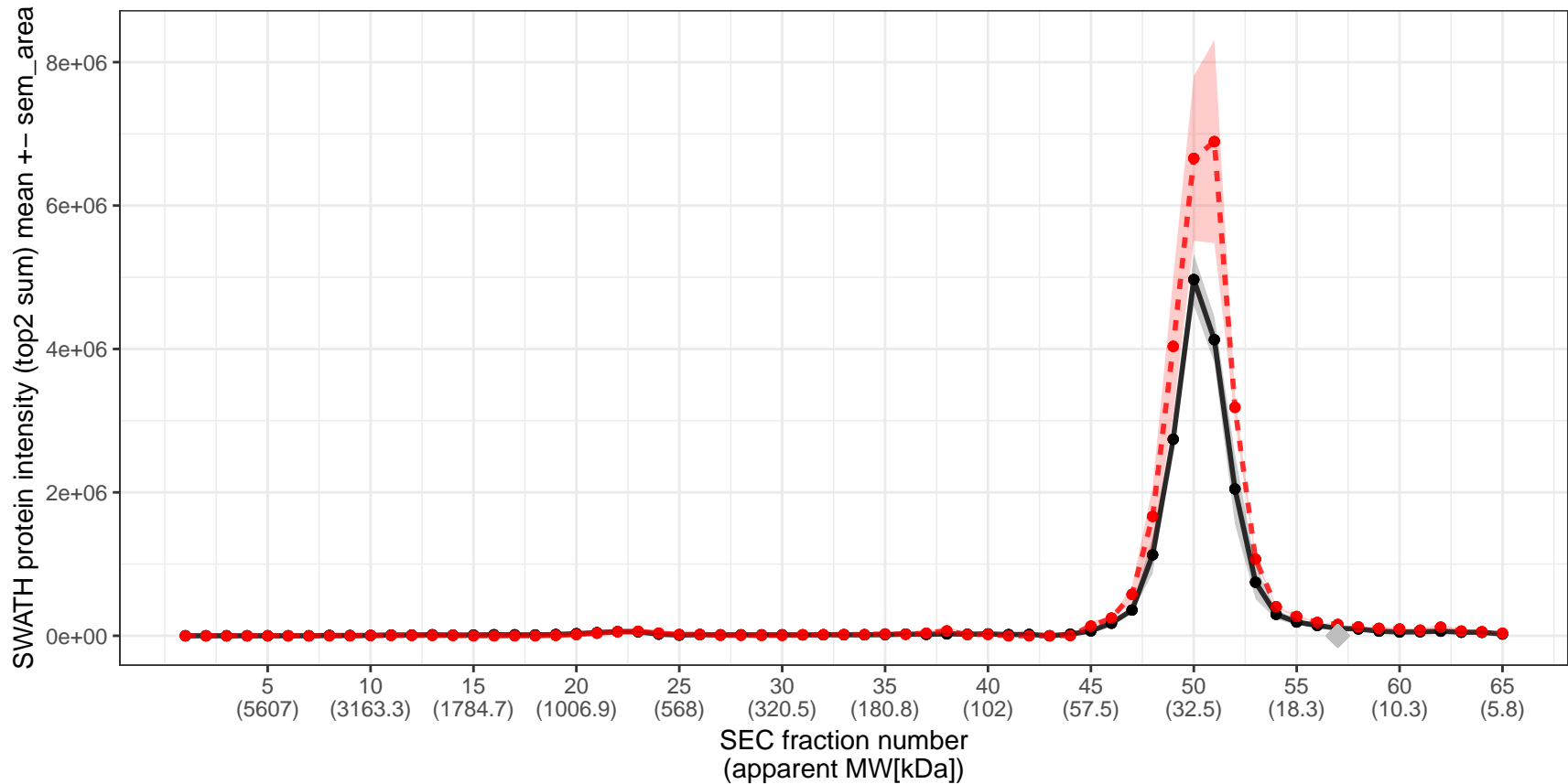

Supplement: Data S1. SEC-SWATH-MS Protein Chromatograms, Related to Figure 1 [file mmc6.zip › SECchrom_O14737_PDCD5_HUMAN_PDCD5_TFAR19.pdf]

O14745 | NHRF1\_HUMAN | SLC9A3R1 NHERF NHERF1  
Monomer MW [kDa]: 38.868 Monomer expected elution fraction: 48

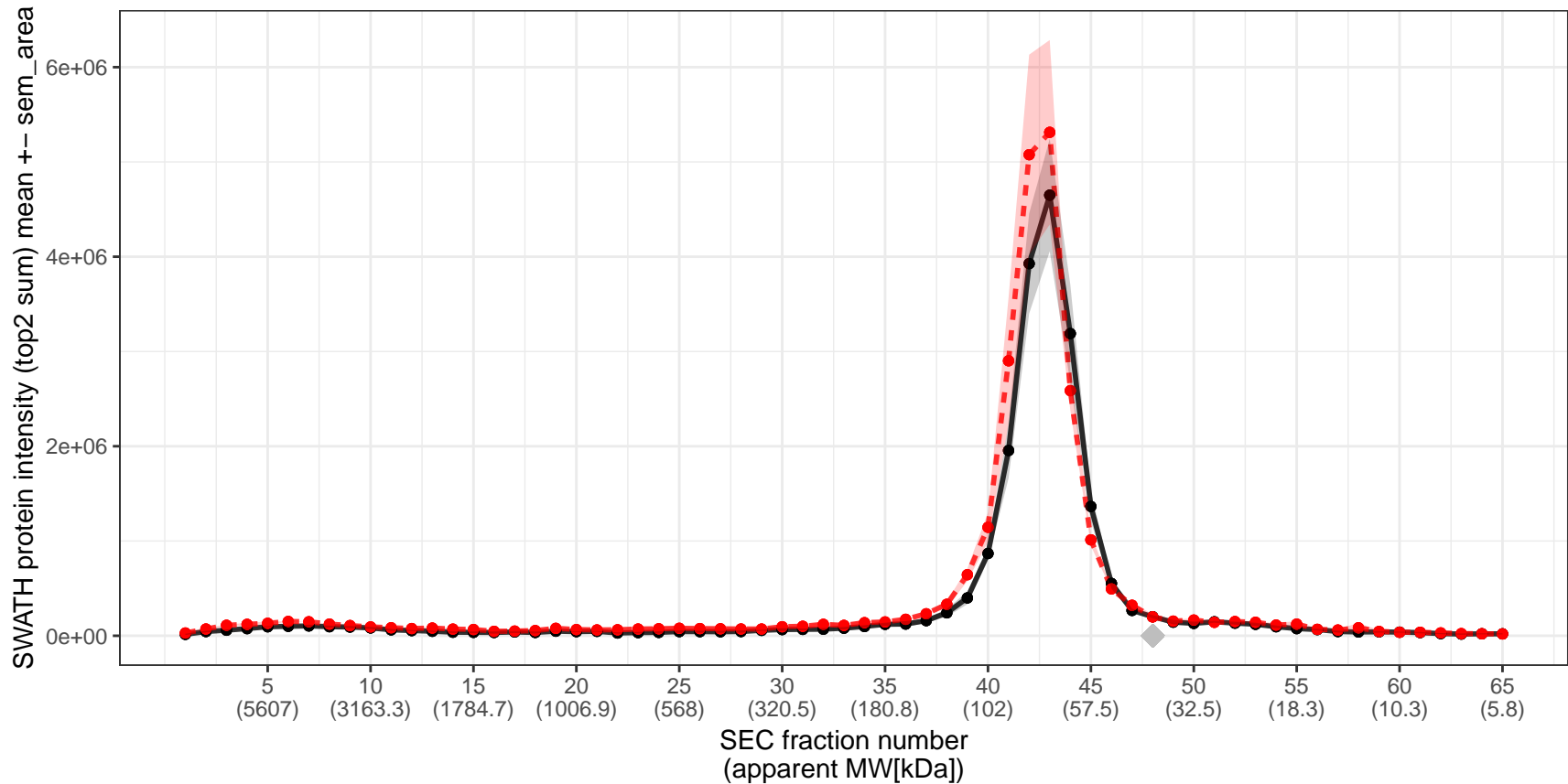

Supplement: Data S1. SEC-SWATH-MS Protein Chromatograms, Related to Figure 1 [file mmc6.zip › SECchrom_O14745_NHRF1_HUMAN_SLC9A3R1_NHERF_NHERF1.pdf]

O14757 | CHK1\_HUMAN | CHEK1

Monomer MW [kDa]: 54.434 Monomer expected elution fraction: 45

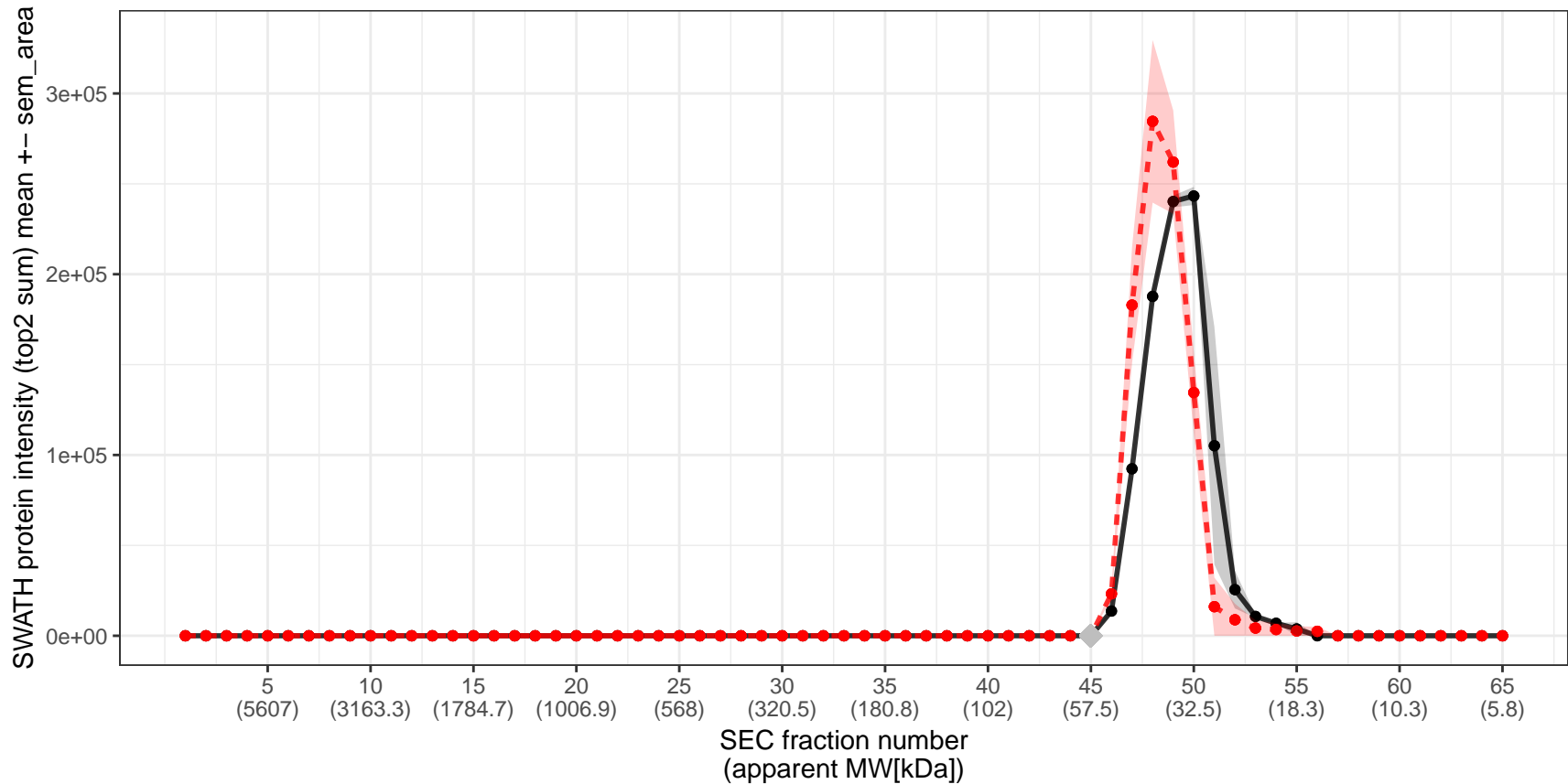

Supplement: Data S1. SEC-SWATH-MS Protein Chromatograms, Related to Figure 1 [file mmc6.zip › SECchrom_O14757_CHK1_HUMAN_CHEK1_CHK1.pdf]

O14777 | NDC80\_HUMAN | NDC80 HEC HEC1 KNTC2

Monomer MW [kDa]: 73.913 Monomer expected elution fraction: 43

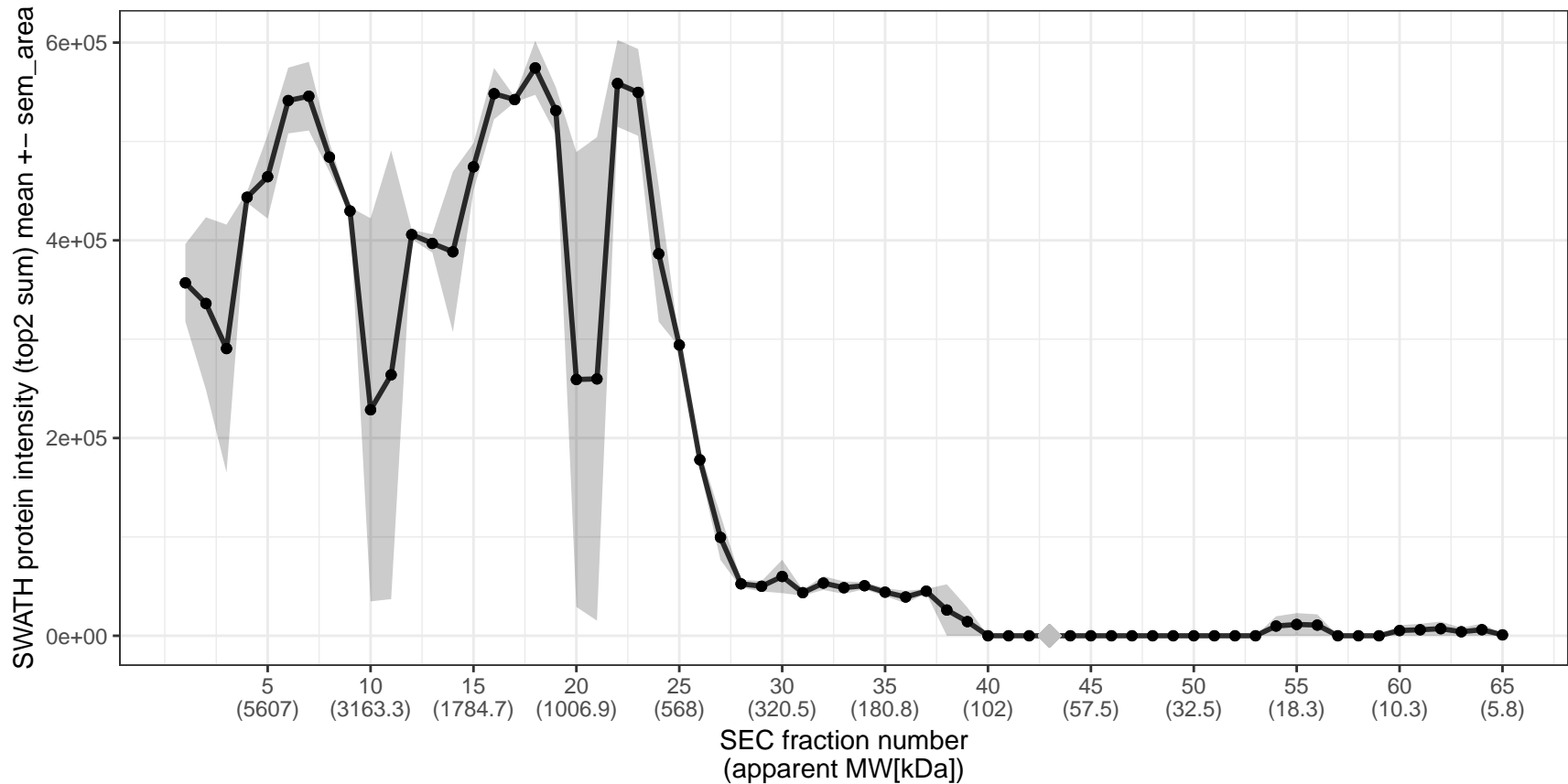

Supplement: Data S1. SEC-SWATH-MS Protein Chromatograms, Related to Figure 1 [file mmc6.zip › SECchrom_O14777_NDC80_HUMAN_NDC80_HEC_HEC1_KNTC2.pdf]

O14802 | RPC1\_HUMAN | POLR3A

Monomer MW [kDa]: 155.641 Monomer expected elution fraction: 36

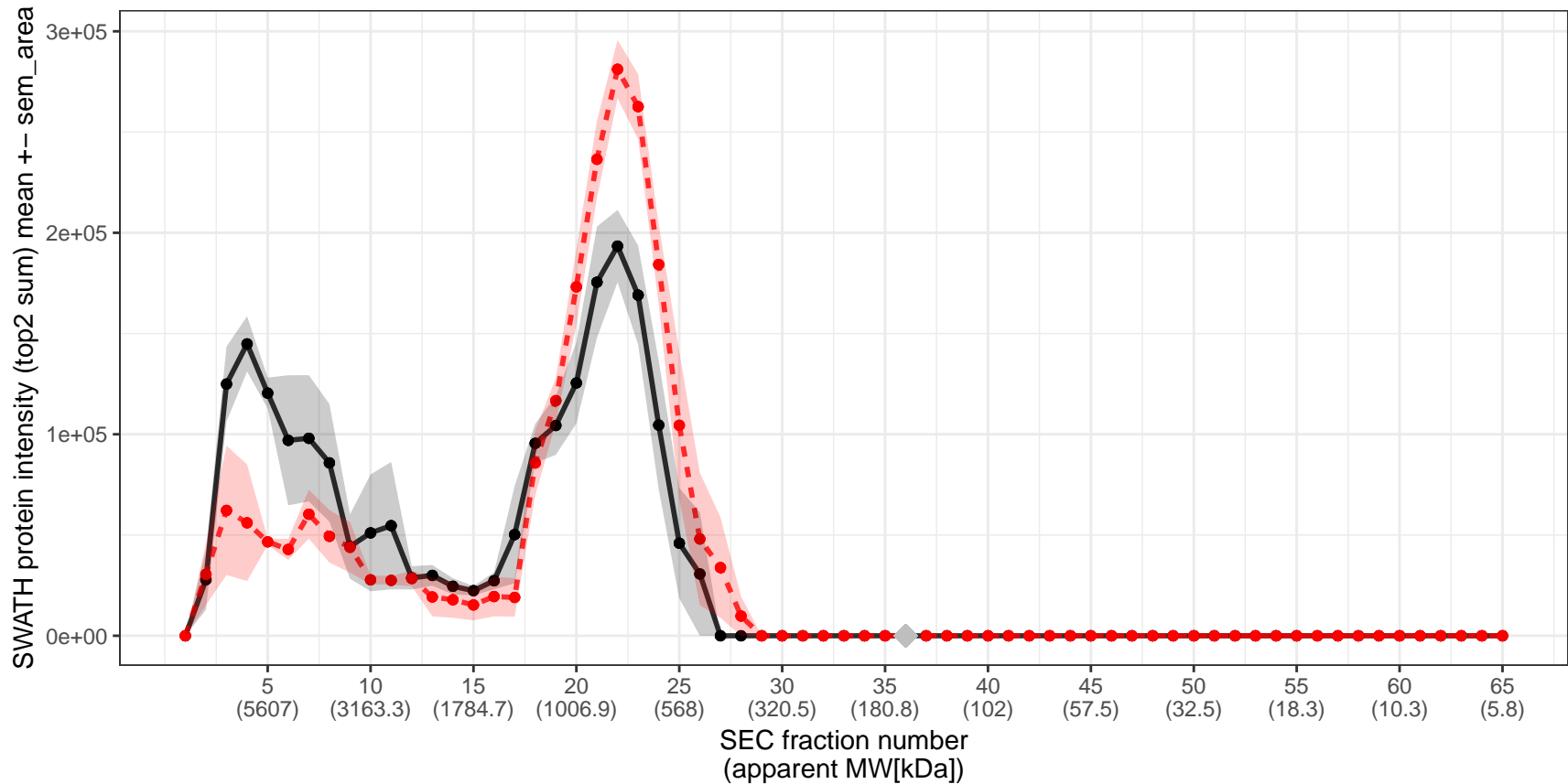

Supplement: Data S1. SEC-SWATH-MS Protein Chromatograms, Related to Figure 1 [file mmc6.zip › SECchrom_O14802_RPC1_HUMAN_POLR3A.pdf]

O14818 | PSA7\_HUMAN | PSMA7 HSPC

Monomer MW [kDa]: 27.887 Monomer expected elution fraction: 51

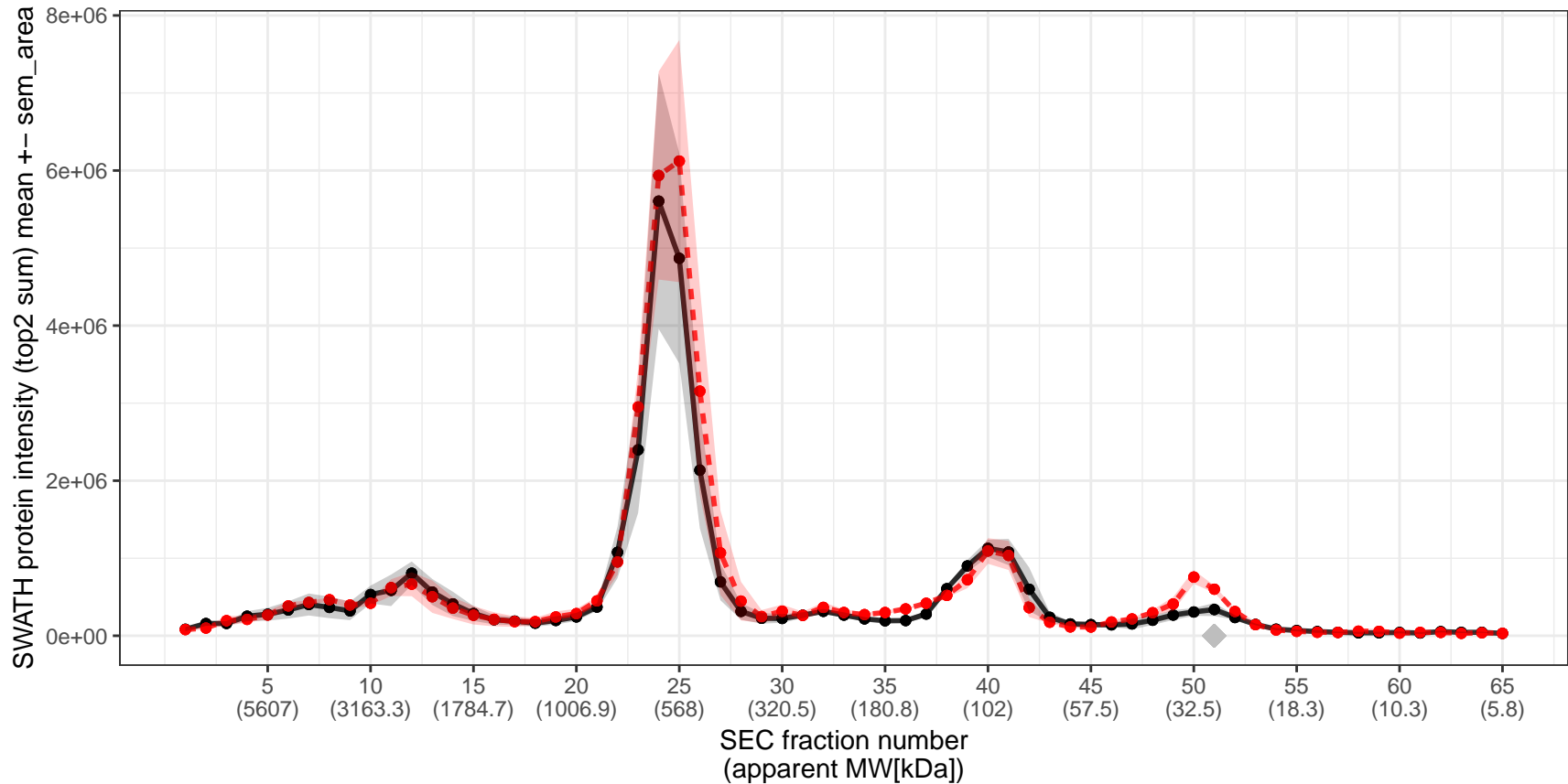

Supplement: Data S1. SEC-SWATH-MS Protein Chromatograms, Related to Figure 1 [file mmc6.zip › SECchrom_O14818_PSA7_HUMAN_PSMA7_HSPC.pdf]

O14828 | SCAM3\_HUMAN | SCAMP3 C1orf3 PROPIN1

Monomer MW [kDa]: 38.287 Monomer expected elution fraction: 49

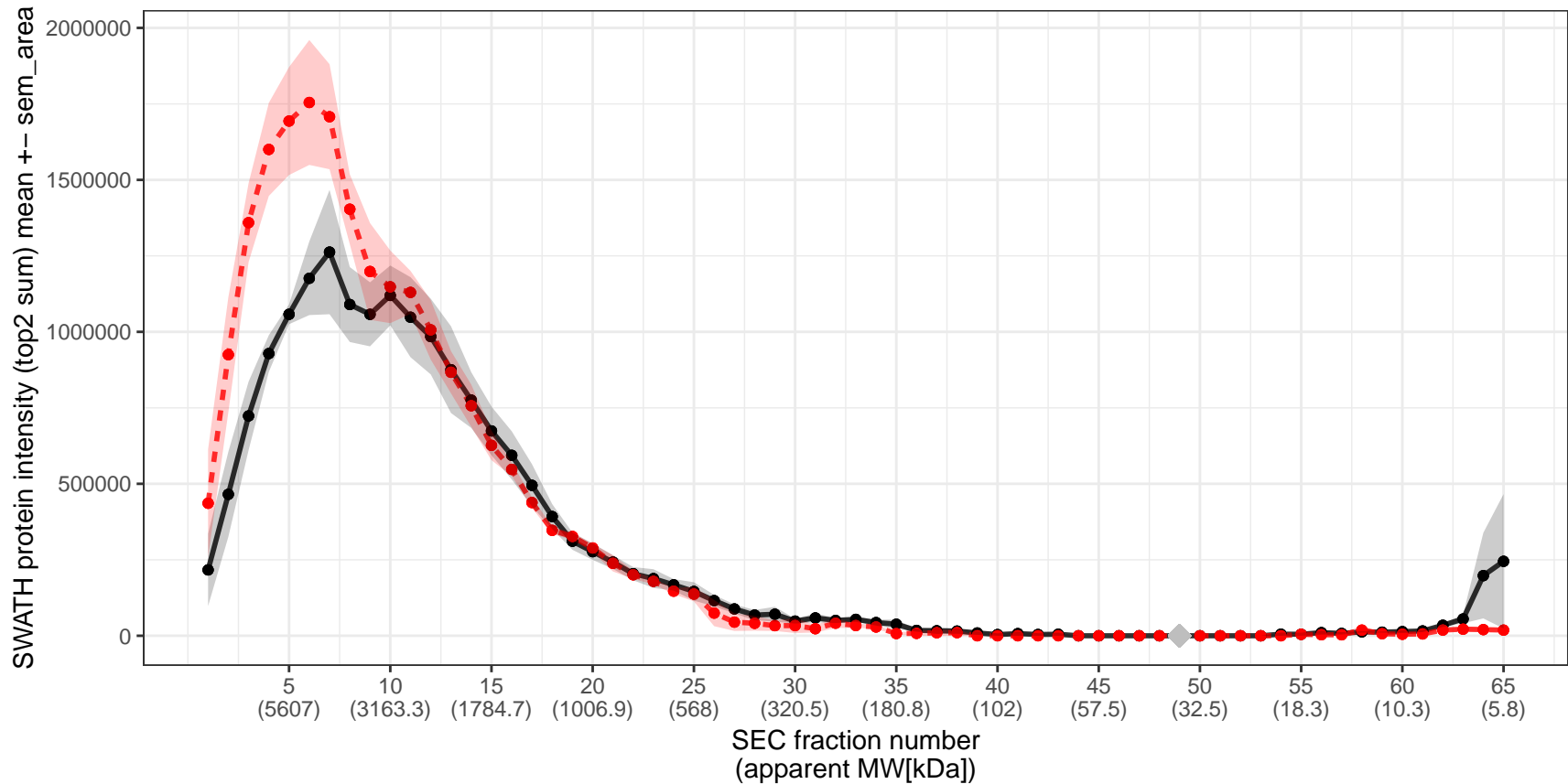

Supplement: Data S1. SEC-SWATH-MS Protein Chromatograms, Related to Figure 1 [file mmc6.zip › SECchrom_O14828_SCAM3_HUMAN_SCAMP3_C1orf3_PROPIN1.pdf]

O14832 | PAHX\_HUMAN | PHYH PAHX

Monomer MW [kDa]: 38.538 Monomer expected elution fraction: 49

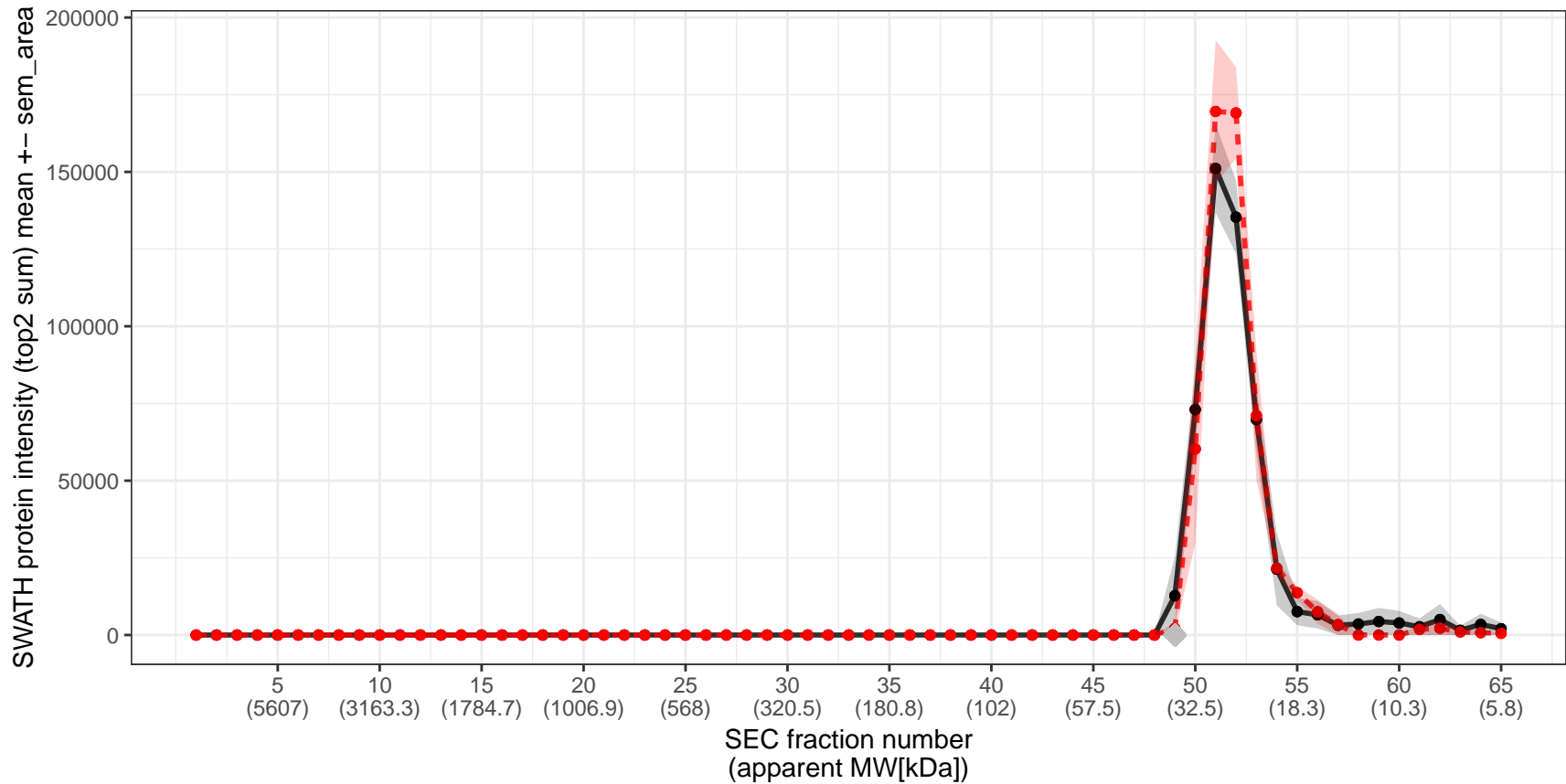

Supplement: Data S1. SEC-SWATH-MS Protein Chromatograms, Related to Figure 1 [file mmc6.zip › SECchrom_O14832_PAHX_HUMAN_PHYH_PAHX.pdf]

O14841 | OPLA\_HUMAN | OPLAH

Monomer MW [kDa]: 137.457 Monomer expected elution fraction: 37

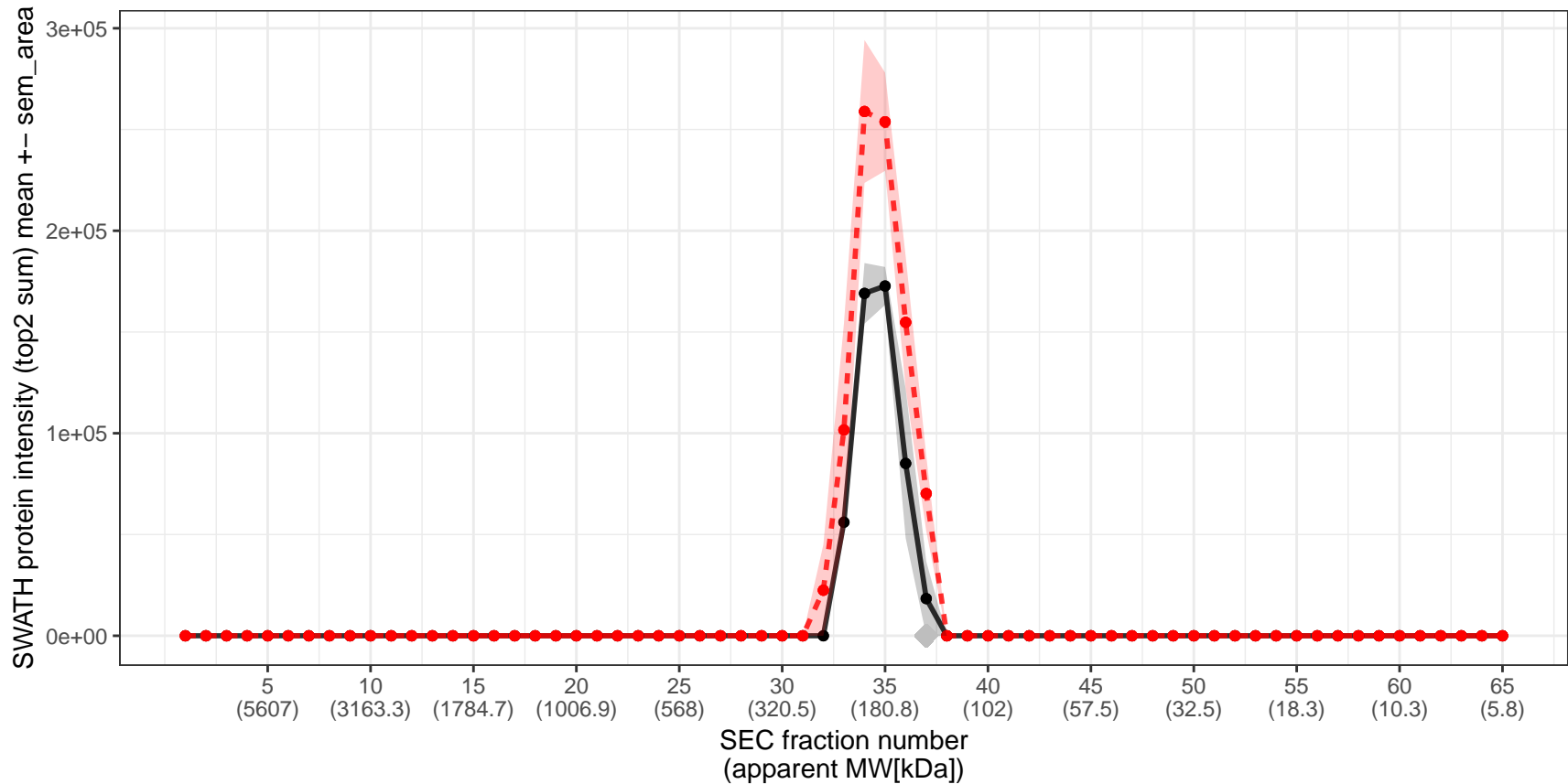

Supplement: Data S1. SEC-SWATH-MS Protein Chromatograms, Related to Figure 1 [file mmc6.zip › SECchrom_O14841_OPLA_HUMAN_OPLAH.pdf]

O14893 | GEMI2\_HUMAN | GEMIN2 SIP1

Monomer MW [kDa]: 31.585 Monomer expected elution fraction: 50

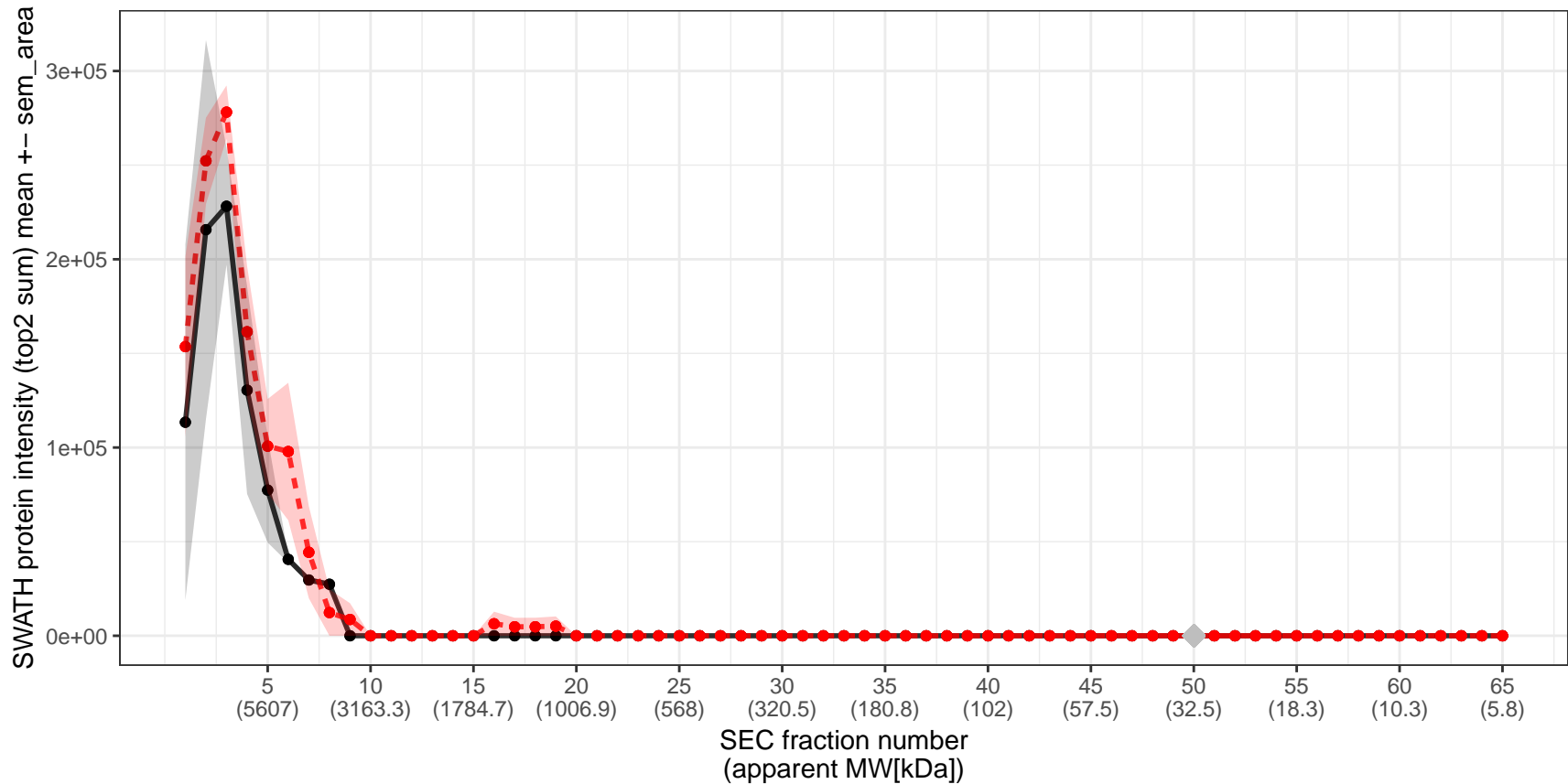

Supplement: Data S1. SEC-SWATH-MS Protein Chromatograms, Related to Figure 1 [file mmc6.zip › SECchrom_O14893_GEMI2_HUMAN_GEMIN2_SIP1.pdf]

O14907 | TX1B3\_HUMAN | TAX1BP3 TIP1

Monomer MW [kDa]: 13.735 Monomer expected elution fraction: 58

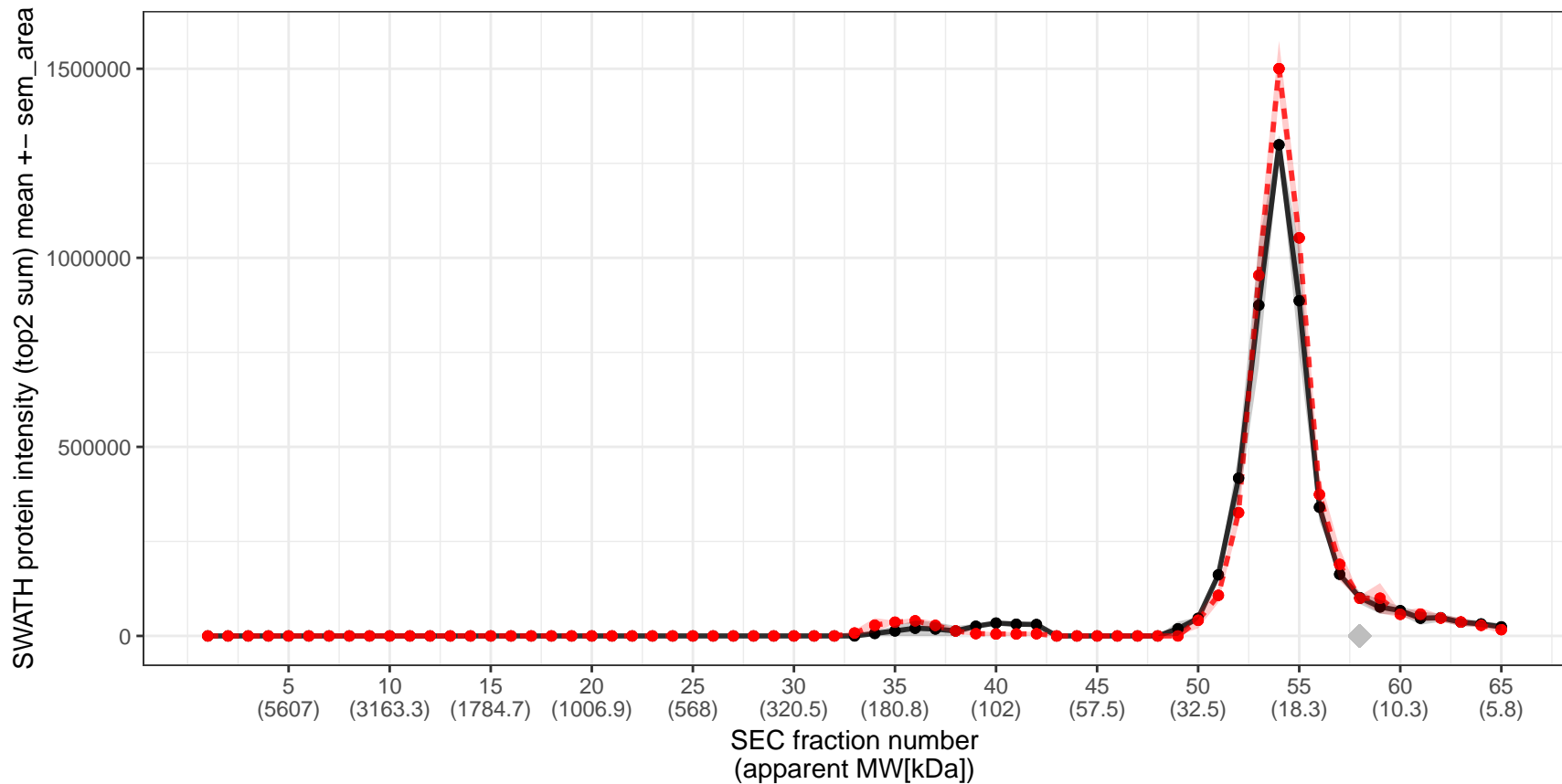

Supplement: Data S1. SEC-SWATH-MS Protein Chromatograms, Related to Figure 1 [file mmc6.zip › SECchrom_O14907_TX1B3_HUMAN_TAX1BP3_TIP1.pdf]

O14908 | GIPC1\_HUMAN | GIPC1 C19orf3 GIPC RGS19IP1  
Monomer MW [kDa]: 36.049 Monomer expected elution fraction: 49

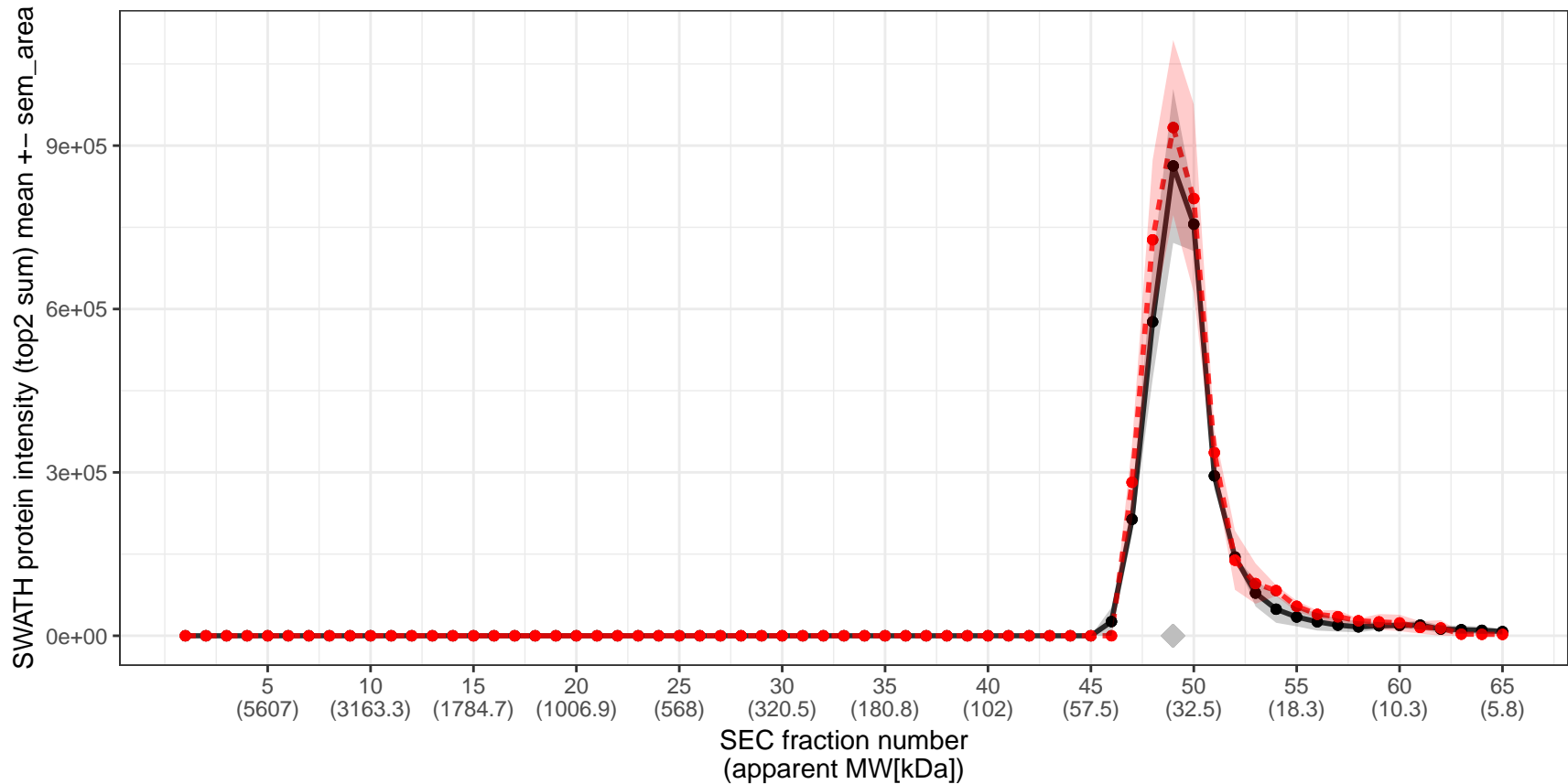

Supplement: Data S1. SEC-SWATH-MS Protein Chromatograms, Related to Figure 1 [file mmc6.zip › SECchrom_O14908_GIPC1_HUMAN_GIPC1_C19orf3_GIPC_RGS19IP1.pdf]

O14920 | IKKB\_HUMAN | IKKB

Monomer MW [kDa]: 86.564 Monomer expected elution fraction: 41

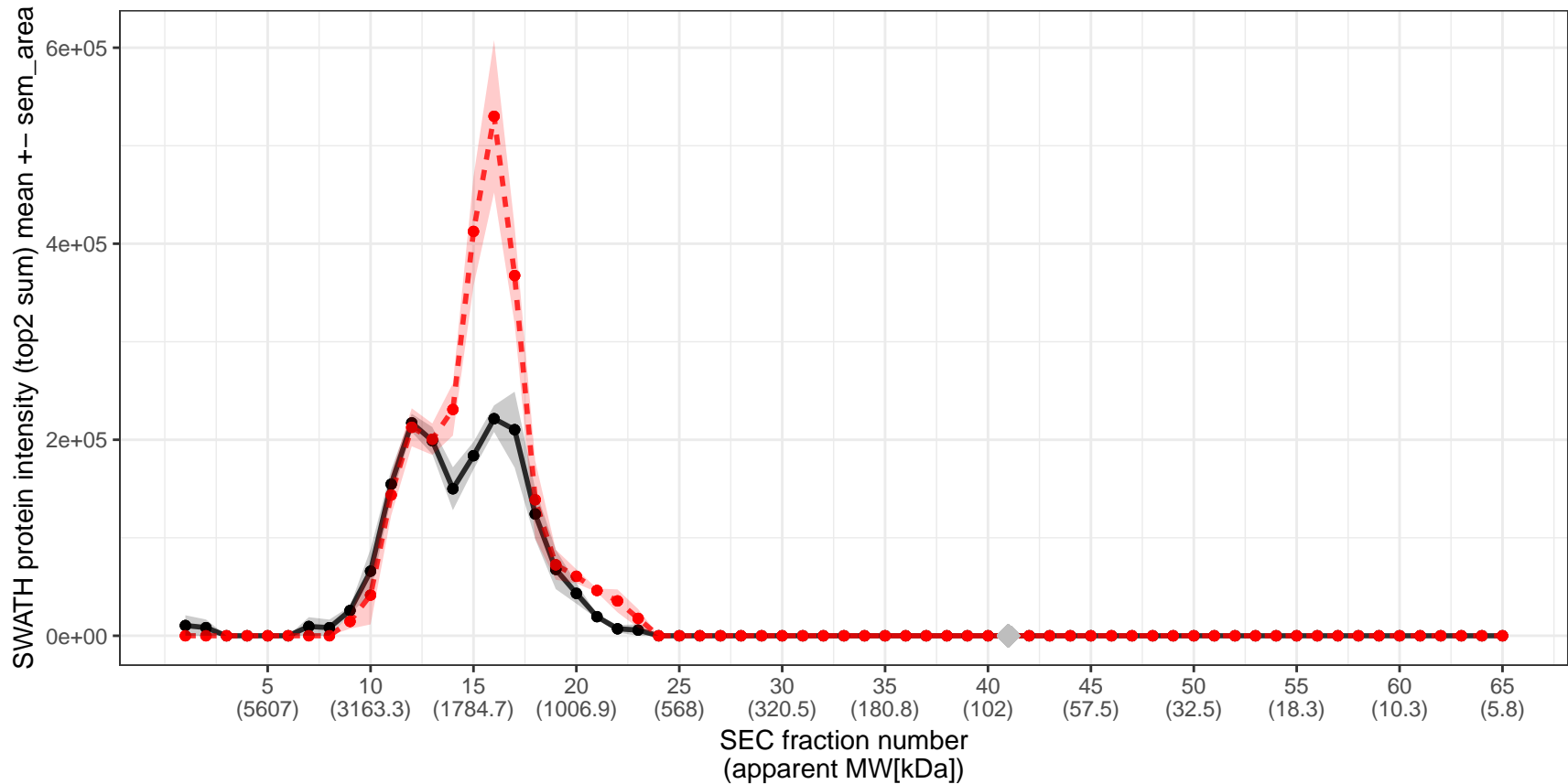

Supplement: Data S1. SEC-SWATH-MS Protein Chromatograms, Related to Figure 1 [file mmc6.zip › SECchrom_O14920_IKKB_HUMAN_IKBKB_IKKB.pdf]

O14929 | HAT1\_HUMAN | HAT1 KAT1

Monomer MW [kDa]: 49.513 Monomer expected elution fraction: 46

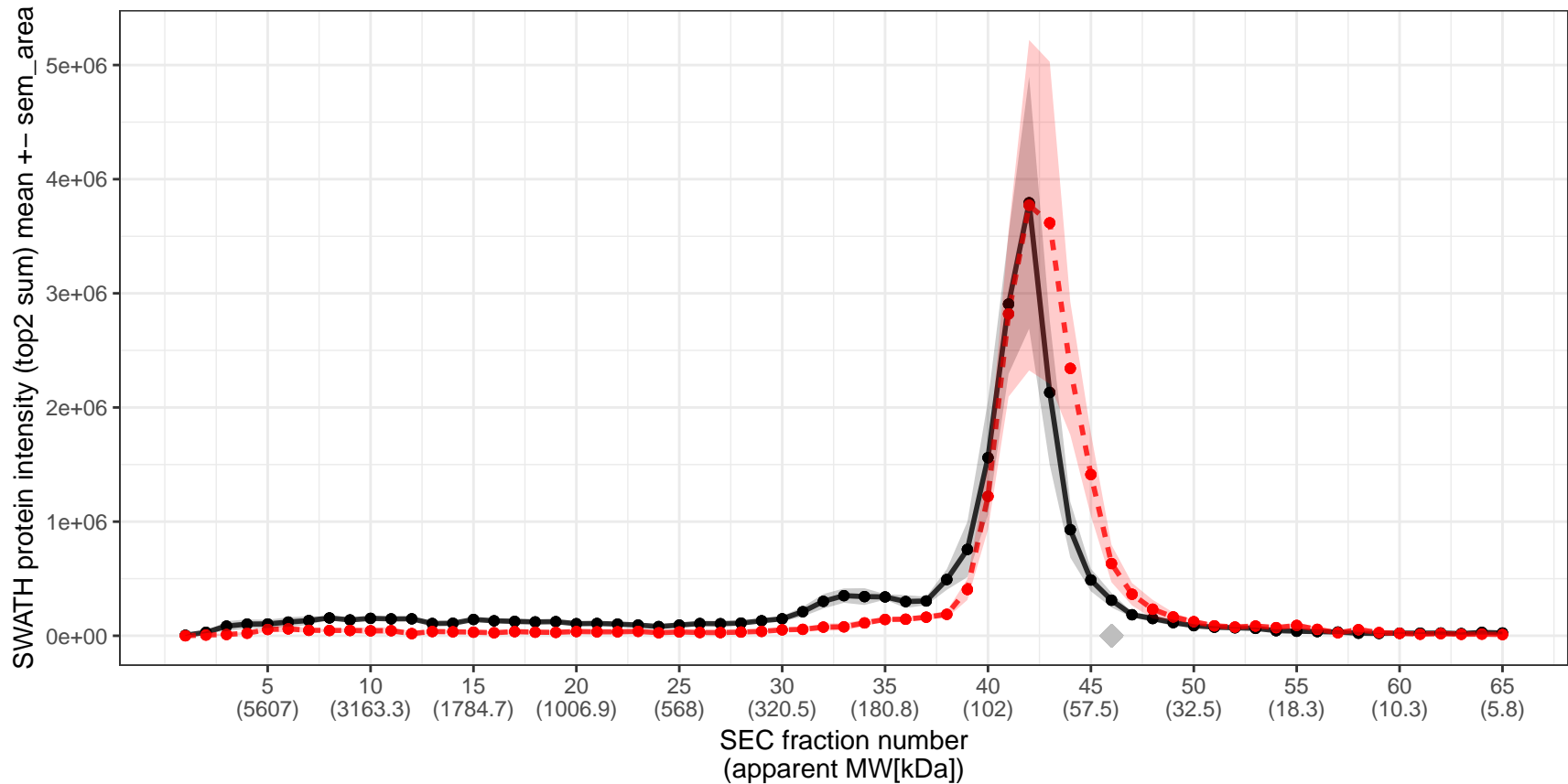

Supplement: Data S1. SEC-SWATH-MS Protein Chromatograms, Related to Figure 1 [file mmc6.zip › SECchrom_O14929_HAT1_HUMAN_HAT1_KAT1.pdf]

O14949 | QCR8\_HUMAN | UQCRQ

Monomer MW [kDa]: 9.906 Monomer expected elution fraction: 60

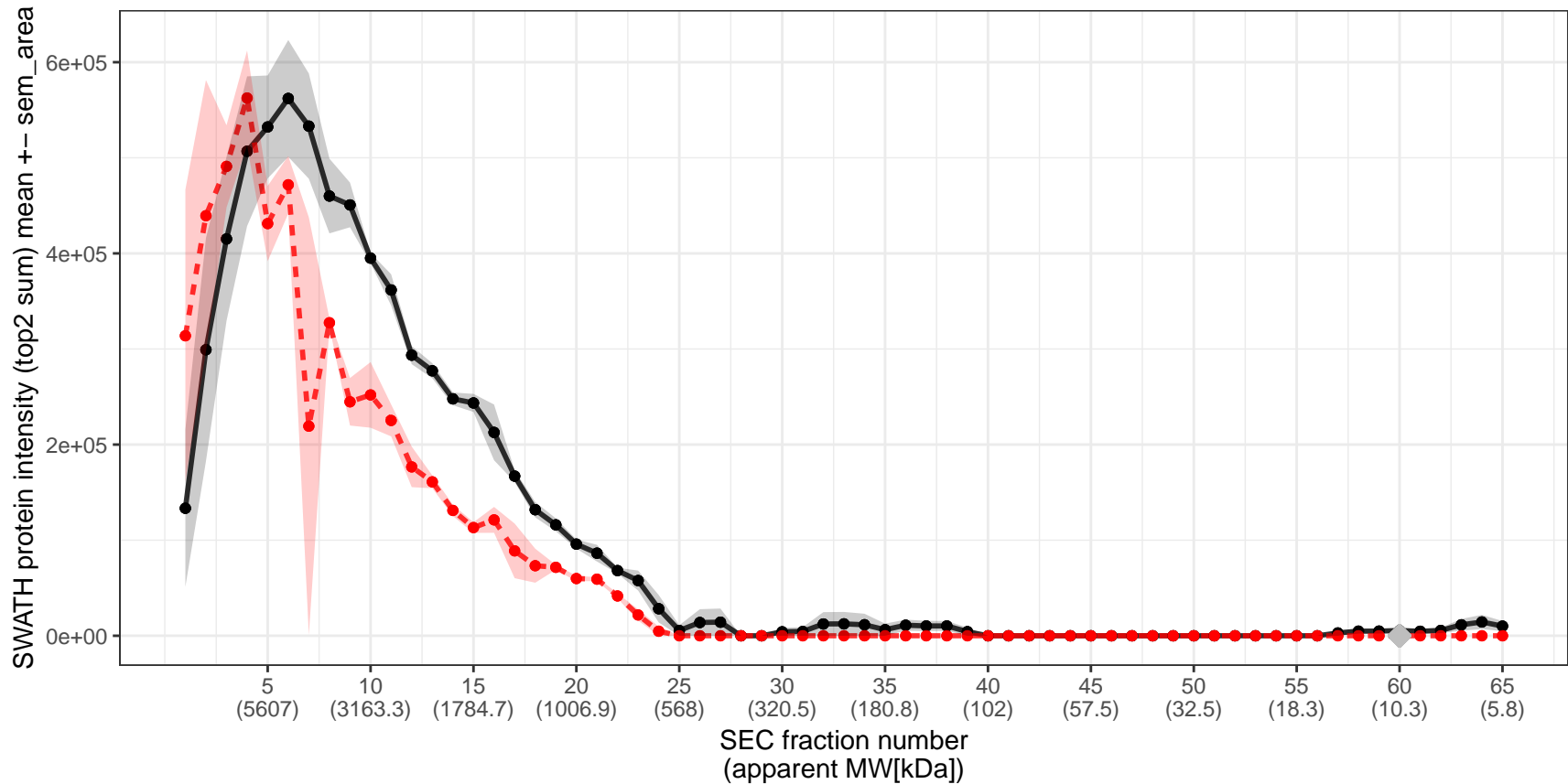

Supplement: Data S1. SEC-SWATH-MS Protein Chromatograms, Related to Figure 1 [file mmc6.zip › SECchrom_O14949_QCR8_HUMAN_UQCRQ.pdf]

O14972 | DSCR3\_HUMAN | DSCR3 DCRA DSCRA

Monomer MW [kDa]: 33.01 Monomer expected elution fraction: 50

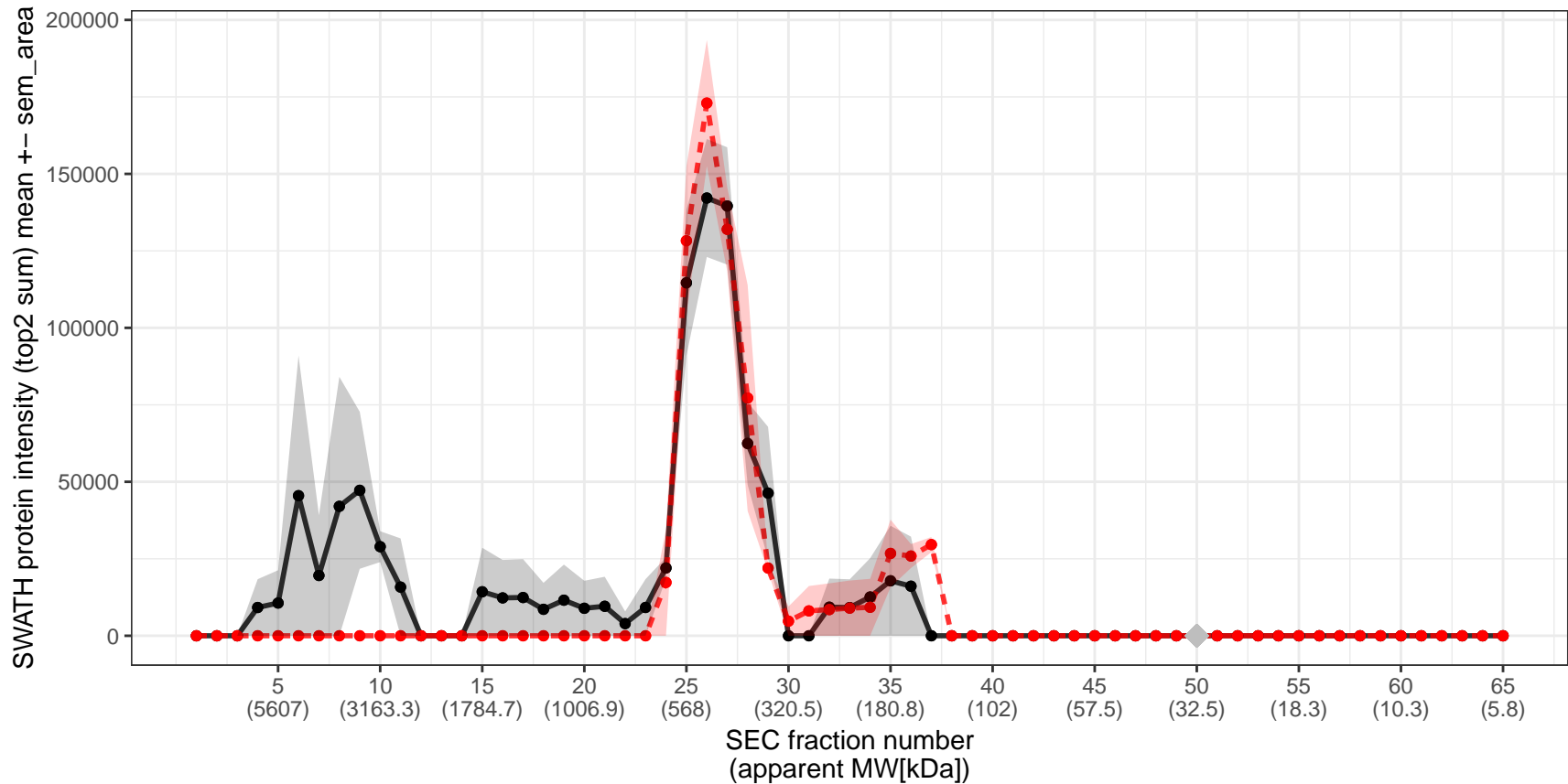

Supplement: Data S1. SEC-SWATH-MS Protein Chromatograms, Related to Figure 1 [file mmc6.zip › SECchrom_O14972_DSCR3_HUMAN_DSCR3_DCRA_DSCRA.pdf]

O14975 | S27A2\_HUMAN | SLC27A2 ACSVL1 FACVL1 FATP2 VLACS

Monomer MW [kDa]: 70.312 Monomer expected elution fraction: 43

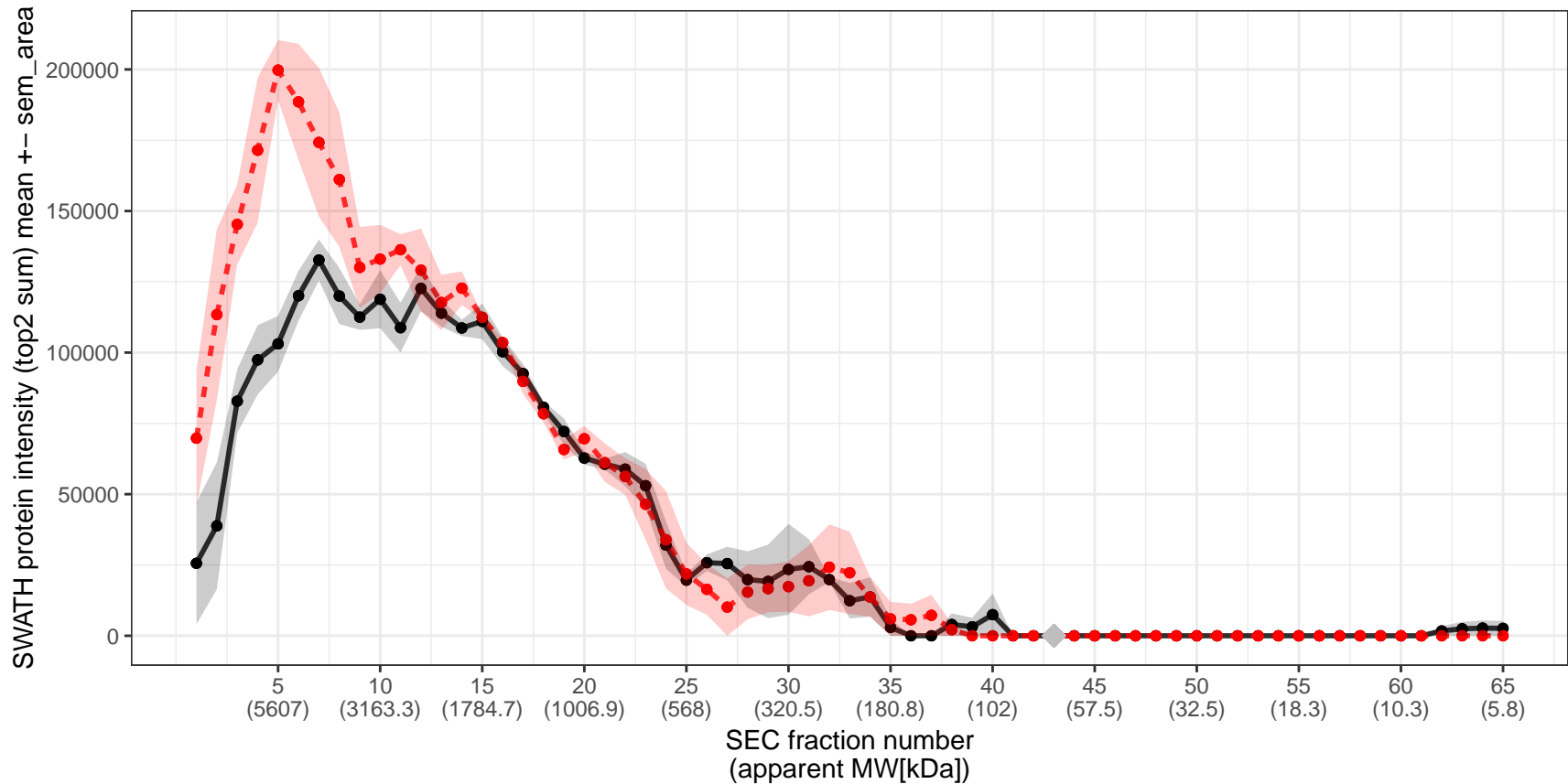

Supplement: Data S1. SEC-SWATH-MS Protein Chromatograms, Related to Figure 1 [file mmc6.zip › SECchrom_O14975_S27A2_HUMAN_SLC27A2_ACSVL1_FACVL1_FATP2_VLA.pdf]

O14976 | GAK\_HUMAN | GAK

Monomer MW [kDa]: 143.191 Monomer expected elution fraction: 37

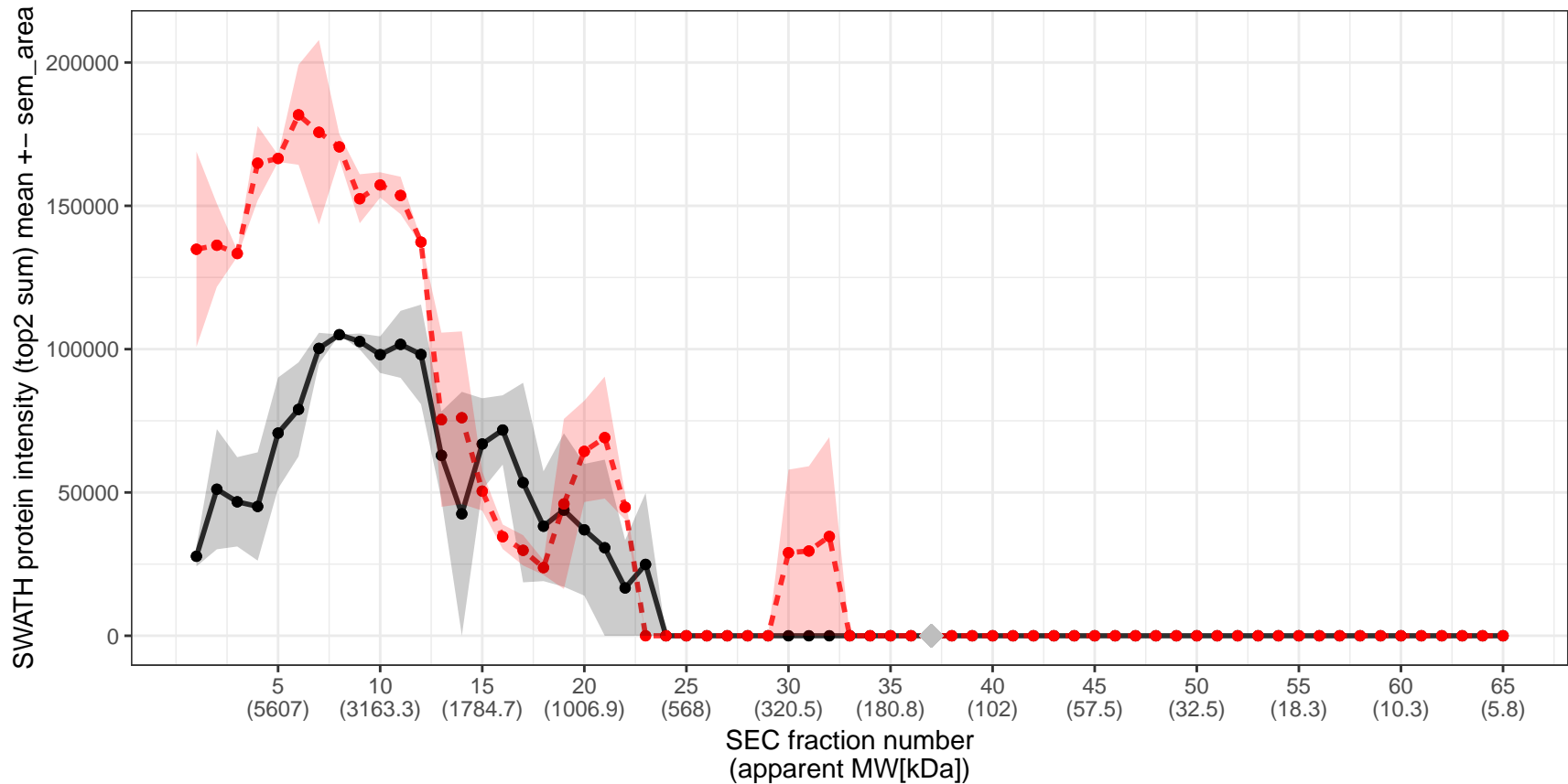

Supplement: Data S1. SEC-SWATH-MS Protein Chromatograms, Related to Figure 1 [file mmc6.zip › SECchrom_O14976_GAK_HUMAN_GAK.pdf]

O15013 | ARHGA\_HUMAN | ARHGEF10 KIAA0294

Monomer MW [kDa]: 151.612 Monomer expected elution fraction: 37

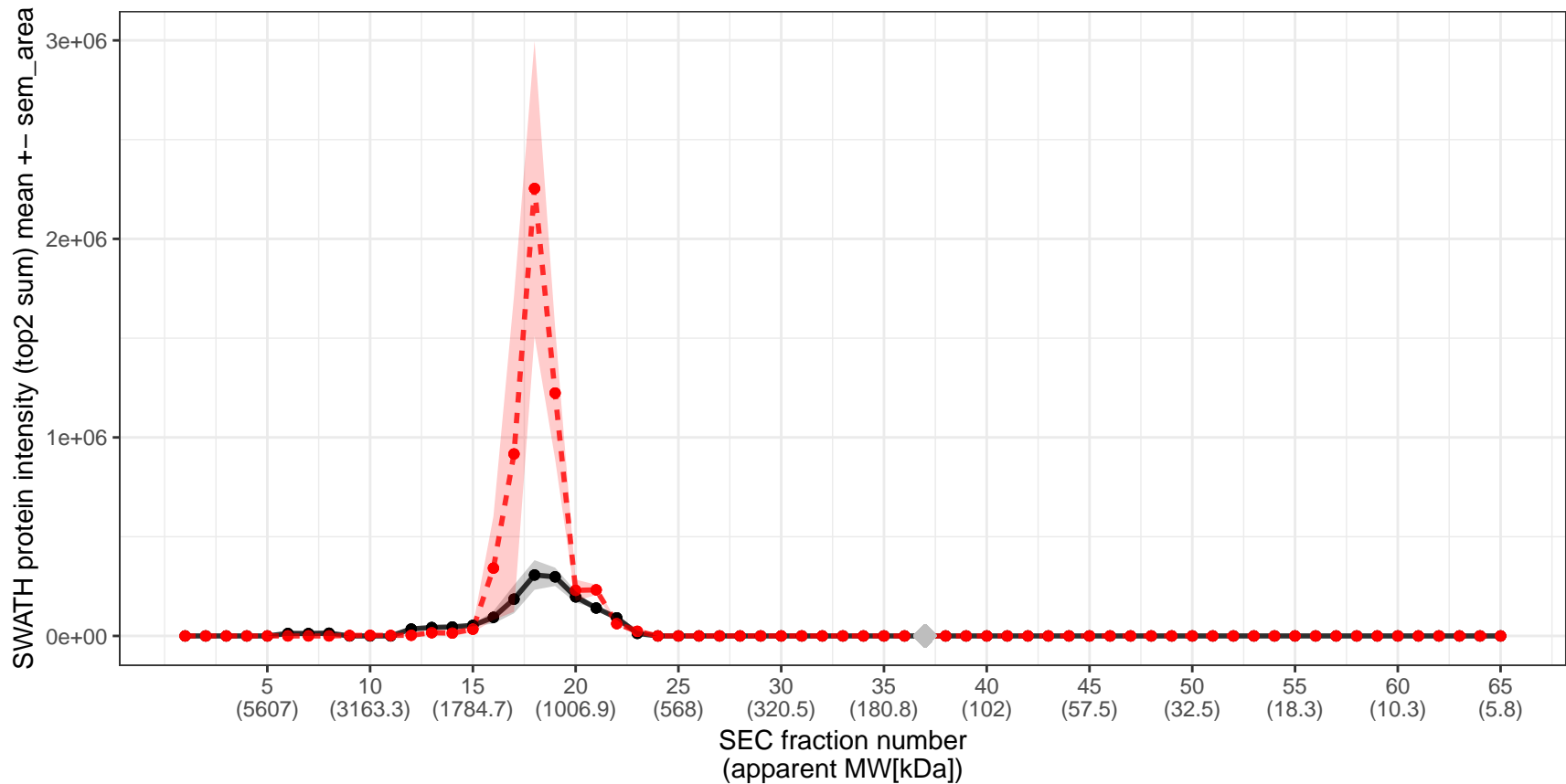

Supplement: Data S1. SEC-SWATH-MS Protein Chromatograms, Related to Figure 1 [file mmc6.zip › SECchrom_O15013_ARHGA_HUMAN_ARHGEF10_KIAA0294.pdf]

O15020 | SPTN2\_HUMAN | SPTBN2 KIAA0302 SCA5

Monomer MW [kDa]: 271.325 Monomer expected elution fraction: 31

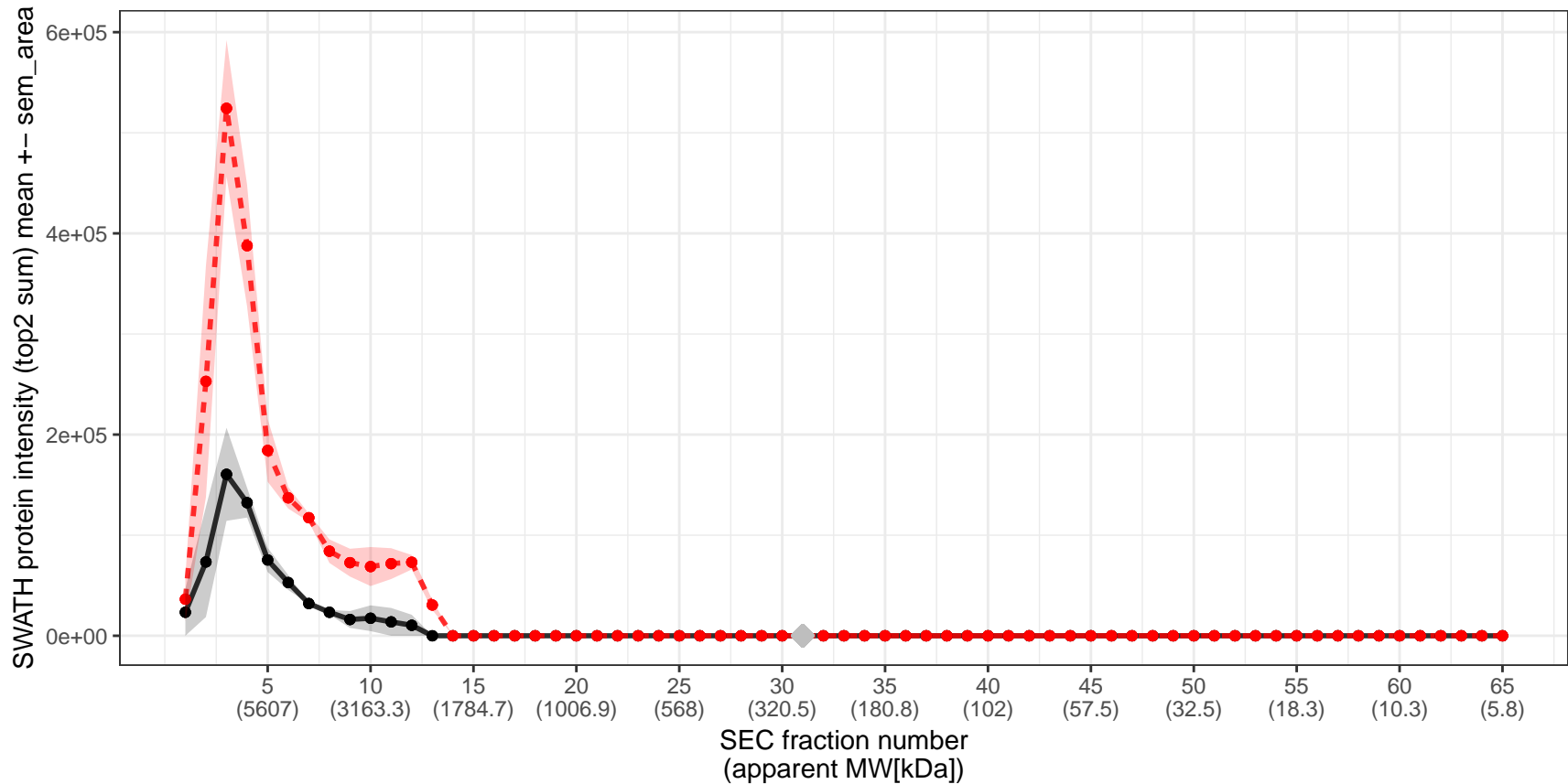

Supplement: Data S1. SEC-SWATH-MS Protein Chromatograms, Related to Figure 1 [file mmc6.zip › SECchrom_O15020_SPTN2_HUMAN_SPTBN2_KIAA0302_SCA5.pdf]

O15042 | SR140\_HUMAN | U2SURP KIAA0332 SR140

Monomer MW [kDa]: 118.292 Monomer expected elution fraction: 39

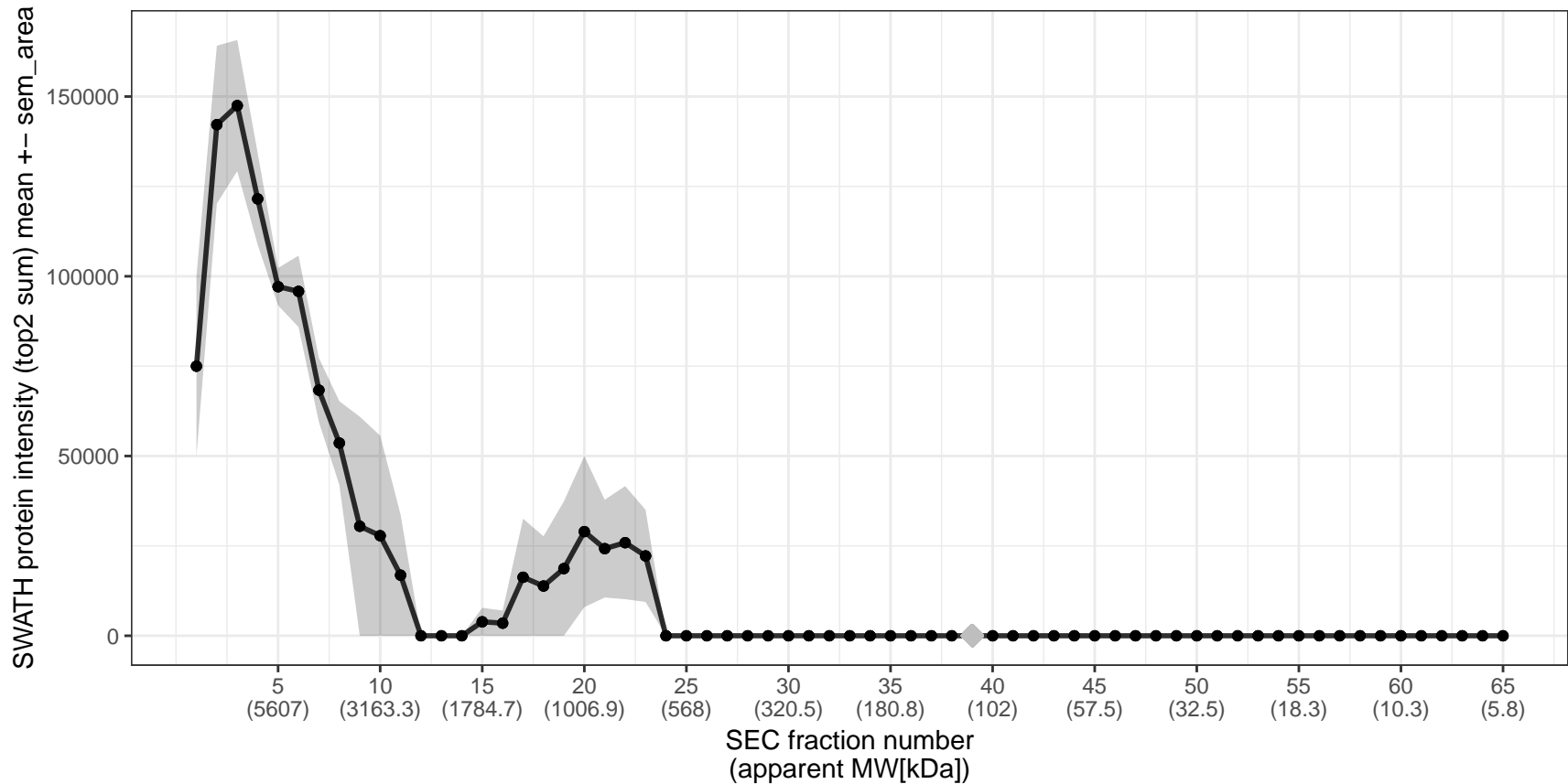

Supplement: Data S1. SEC-SWATH-MS Protein Chromatograms, Related to Figure 1 [file mmc6.zip › SECchrom_O15042_SR140_HUMAN_U2SURP_KIAA0332_SR140.pdf]

O15056 | SYNJ2\_HUMAN | SYNJ2 KIAA0348

Monomer MW [kDa]: 165.538 Monomer expected elution fraction: 36

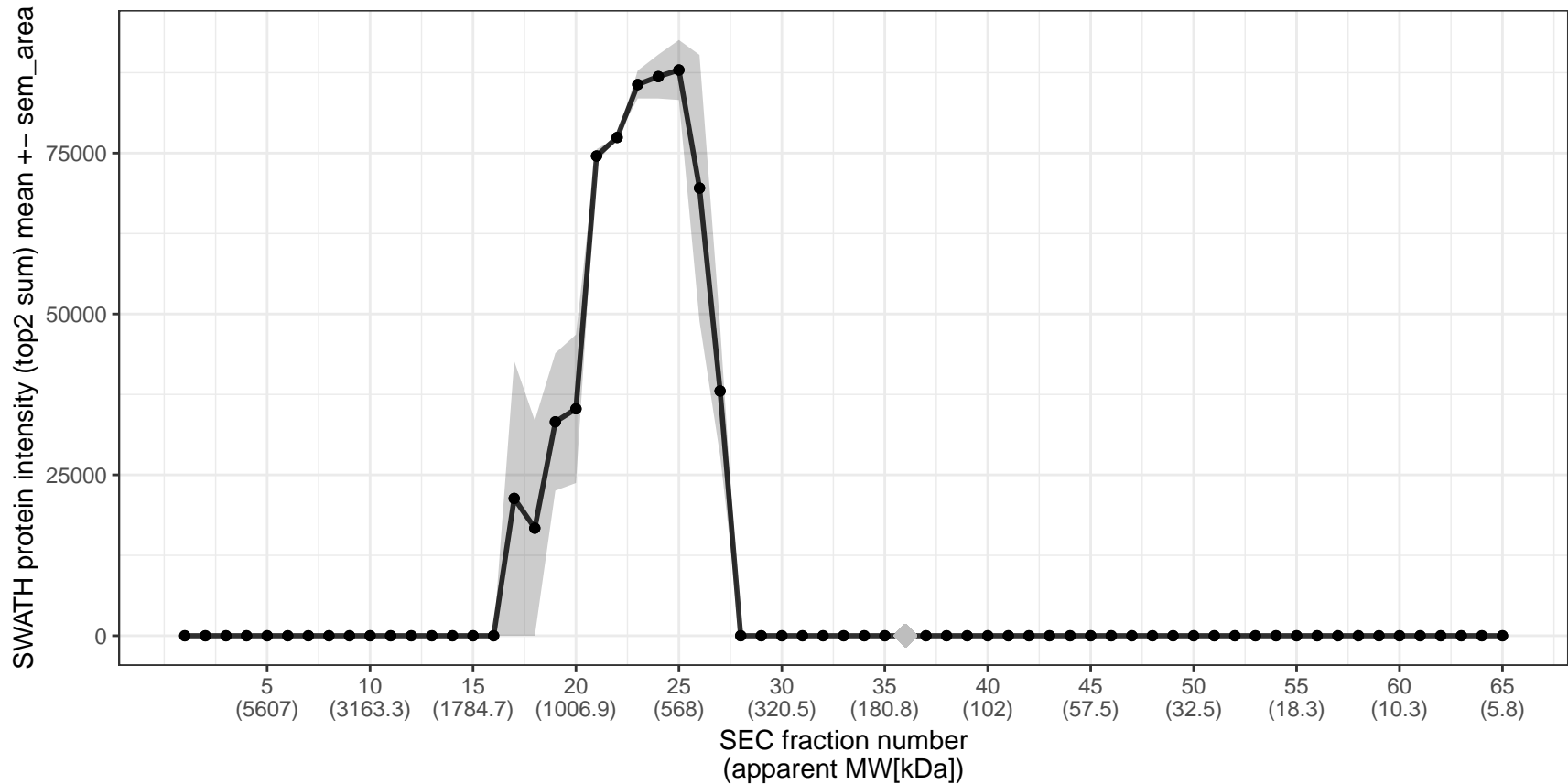

Supplement: Data S1. SEC-SWATH-MS Protein Chromatograms, Related to Figure 1 [file mmc6.zip › SECchrom_O15056_SYNJ2_HUMAN_SYNJ2_KIAA0348.pdf]
